# Unravelling Intratumoral Heterogeneity through High-Sensitivity Single-Cell Mutational Analysis and Parallel RNA Sequencing

**DOI:** 10.1016/j.molcel.2019.01.009

**Published:** 2019-03-21

**Authors:** Alba Rodriguez-Meira, Gemma Buck, Sally-Ann Clark, Benjamin J. Povinelli, Veronica Alcolea, Eleni Louka, Simon McGowan, Angela Hamblin, Nikolaos Sousos, Nikolaos Barkas, Alice Giustacchini, Bethan Psaila, Sten Eirik W. Jacobsen, Supat Thongjuea, Adam J. Mead

**Affiliations:** 1 Haematopoietic Stem Cell Biology Laboratory, Medical Research Council Weatherall Institute of Molecular Medicine, University of Oxford, Oxford OX3 9DS, UK; 2 Medical Research Council Molecular Haematology Unit, Weatherall Institute of Molecular Medicine, University of Oxford, Oxford OX3 9DS, UK; 3 Flow Cytometry Facility, Medical Research Council, Weatherall Institute of Molecular Medicine, University of Oxford, Oxford OX3 9DS, UK; 4 Medical Research Council Centre for Computational Biology, Weatherall Institute of Molecular Medicine, University of Oxford, Oxford OX3 9DS, UK; 5 National Institute for Health Research Biomedical Research Centre, University of Oxford, Oxford, UK; 6 Department of Cell and Molecular Biology, Wallenberg Institute for Regenerative Medicine, Karolinska Institutet, Stockholm, Sweden; 7 Karolinska University Hospital, Stockholm, Sweden; 8 Department of Medicine Huddinge, Center for Hematology and Regenerative Medicine, Karolinska Institutet, Stockholm, Sweden

**Keywords:** Heterogeneity, single-cell, cancer, hematopoiesis, transcriptomics, myeloproliferative neoplasm, leukemia, mutations, TARGET-seq, sequencing

## Abstract

Single-cell RNA sequencing (scRNA-seq) has emerged as a powerful tool for resolving transcriptional heterogeneity. However, its application to studying cancerous tissues is currently hampered by the lack of coverage across key mutation hotspots in the vast majority of cells; this lack of coverage prevents the correlation of genetic and transcriptional readouts from the same single cell. To overcome this, we developed TARGET-seq, a method for the high-sensitivity detection of multiple mutations within single cells from both genomic and coding DNA, in parallel with unbiased whole-transcriptome analysis. Applying TARGET-seq to 4,559 single cells, we demonstrate how this technique uniquely resolves transcriptional and genetic tumor heterogeneity in myeloproliferative neoplasms (MPN) stem and progenitor cells, providing insights into deregulated pathways of mutant and non-mutant cells. TARGET-seq is a powerful tool for resolving the molecular signatures of genetically distinct subclones of cancer cells.

## Highlights

• Conventional scRNA-seq protocols do not allow reliable mutational analysis
• TARGET-seq combines high-sensitivity genomic DNA and cDNA genotyping with scRNA-seq
• TARGET-seq resolves the distinct transcriptional signatures of tumor genetic subclones
• Non-mutant cells from patients show aberrant, inflammation-associated gene expression

## Graphical Abstract

**Figure:**
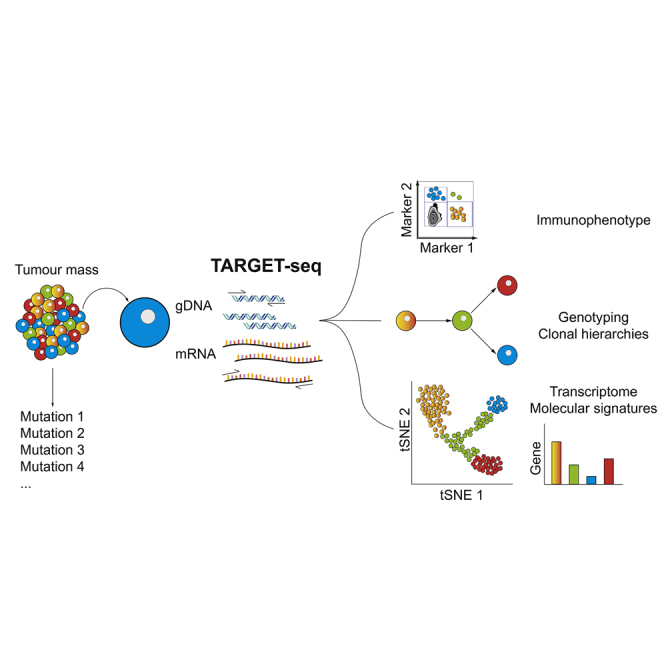

## Teaser

Rodriguez-Meira et al. developed TARGET-seq, a method for high-sensitivity mutational analysis and parallel RNA sequencing from the same single cell. Applied to 4,559 single cells, TARGET-seq unraveled transcriptional and genetic tumor heterogeneity in myeloproliferative neoplasm (MPN) stem and progenitor cells. TARGET-seq is a powerful tool for resolving the molecular signatures of genetically distinct tumor subclones.

## Introduction

Resolving intratumoral heterogeneity (ITH) is critical for our understanding of tumor evolution and resistance to therapies; this understanding, in turn, is required for the development of effective cancer treatments and biomarkers for precision medicine (McGranahan and Swanton, 2017). The best-characterized source of ITH has been at the genetic level; this heterogeneity has been identified through advances in next-generation sequencing (NGS) techniques at the bulk and single-cell levels (Vogelstein et al., 2013). However, certain factors beyond somatic mutations contribute to ITH. For example, some tumors are hierarchically organized and contain cancer stem cells (CSCs), which propagate disease relapse. The genetic events underlying tumor evolution originate in CSCs, which in some tumors are rare within the total tumor bulk population (Clevers, 2011, Magee et al., 2012, Woll et al., 2014). Furthermore, the CSCs’ normal cellular counterparts, which lack genetic mutations, can be difficult to distinguish from malignant cells because they might share phenotypic features, but these cells can nevertheless be informative for disease biology (Giustacchini et al., 2017). Consequently, resolving ITH requires methods that allow these multiple layers of heterogeneity to be teased apart.

A potentially powerful approach for gaining a better understanding of the functional consequences of ITH is to link genetic ITH with the transcriptional signatures of distinct subpopulations of tumor cells. A number of studies have begun to apply single-cell RNA sequencing (scRNA-seq) to characterize different malignancies, demonstrating the power of scRNA-seq to identify the different cell types that are encompassed within a tumor, including cells with “stemness” signatures and characterization of developmental hierarchies of tumor cells (Patel et al., 2014, Tirosh et al., 2016a, Tirosh et al., 2016b, Venteicher et al., 2017). However, although scRNA-seq approaches can readily resolve such transcriptional heterogeneity, current techniques do not allow parallel mutational analysis because of a lack of coverage across mutation hotspots (Kiselev et al., 2017, Patel et al., 2014, Tirosh et al., 2016b). This integration of mutational and transcriptional information is crucial for linking genetic evolution events to the cell of origin; this is of considerable importance because serial mutation acquisition might occur within distinct and developmentally ordered stem and progenitor cell types, as described in acute leukemia (Jan et al., 2012). Furthermore, mutation analysis is also important for unravelling disrupted gene expression in non-mutant cells; this disruption of gene expression might be cell-extrinsically mediated and of clinical importance (Giustacchini et al., 2017). In order to overcome this current limitation in single-cell genomic techniques, we set out to develop a method that combines full-length scRNA-seq or 3′-end-counting, high-throughput scRNA-seq with high-sensitivity mutation analysis.

## Design

The limitation of applying current scRNA-sequencing techniques to the detection of mutations in single cells partly relates to the fact that commonly used “end-counting” scRNA-seq techniques only detect the 3′ or 5′ region of transcripts (Hedlund and Deng, 2018). Consequently, most mutations within the body of a gene are not covered by sequencing reads. However, scRNA-seq techniques that amplify full-length transcripts, such as Smart-seq2 (Picelli et al., 2013), also have very poor sensitivity with regard to detecting the expression of most genes in most cells (Figure S1), and this difficulty precludes high-sensitivity mutational analysis. Furthermore, the vast majority of mutations identified in cancer are single-nucleotide variants (SNVs) and small indels (Vogelstein et al., 2013); these might be either heterozygous or associated with loss of heterozygosity (LOH) and have important functional consequences (Kharazi et al., 2011). Therefore, a key challenge in the field is to minimize allelic dropouts (ADOs) in order to ensure the detection of both alleles from a single cell.

It remains unclear whether the high ADO rates and lack of coverage across mutation hotspots in scRNA-seq data is primarily due to technical dropouts related to inefficient reverse transcription (RT) and/or PCR amplification or whether they are the result of true biological heterogeneity in the expression of mutant transcripts across single cells. We therefore first optimized the Smart-seq2 RT and PCR enzymatic conditions (SMART-seq+; Table S1A); this resulted in a significant reduction in dropout rates (Figure S1A), particularly for genes expressed at a low level (Figures S1B and C); a 25% increase in the number of genes detected per cell (Figure S1D); and a reduction in library bias (Figure S1E). However, despite improved sensitivity for the detection of gene expression with SMART-seq+, ADO rates remained exceedingly high for most genes (Figures S1F–H), a fact that currently precludes reliable mutational analysis using scRNA-seq (Povinelli et al., 2018). We therefore concluded that, because of the stochastic nature of gene expression in single cells, improving sensitivity for the analysis of coding DNA (cDNA) alone is unlikely to provide sufficient sensitivity for the detection of most cancer-associated mutations at the single-cell level.

Overcoming this problem requires the detection of mutations from genomic DNA (gDNA) in parallel with cDNA. Techniques for studying gDNA and mRNA from the same single cell have been previously described. However, these techniques either require both types of molecules to be physically separated (Han et al., 2018, Hou et al., 2016, Macaulay et al., 2015), which inevitably results in some loss of genetic material and consequently limits the techniques’ sensitivity, or they rely on the parallel amplification of total gDNA and mRNA followed by the masking of coding regions (Dey et al., 2015). These technical constraints restrict the sensitivity of such techniques for the confident detection of specific point mutations. Whole-genome amplification also introduces significant expense to the method and has inherently high ADO and false-positive rates (Hosokawa et al., 2017, Wang et al., 2014). As a result, up to now, these techniques have not been widely used for parallel mutation or scRNA-seq analysis in cancer. Methods that combine targeted single-cell gene expression and mutation analysis have also been reported (Cheow et al., 2016, Wang et al., 2017), but these approaches have the limitation that only the expression of a limited number of pre-selected genes can be analyzed per cell.

Recently, we have described a method for the high-sensitivity detection of BCR-ABL1 (breakpoint cluster region and Abelson murine leukemia viral oncogene homolog 1 fusion protein) transcripts in parallel with scRNA-seq in chronic myeloid leukemia stem cells (Giustacchini et al., 2017). Although this study highlights the power of linking mutation and transcriptome information in single cells, the method is dependent on the expression of the targeted gene and/or allele in all mutated cells. This approach was effective in the specific case of the *BCR-ABL* fusion gene. However, for many autosomal genes, expression is undetectable or highly allelic-biased in the majority of transcriptionally active and highly proliferative K562 cells (Figure S1F) and also in quiescent Lin^−^CD34^+^CD38^−^ primary human hematopoietic stem and progenitor cells (HSPCs; Figures S1G and H); this makes this method unsuitable to profile most mutations found in cancer. Moreover, this approach precludes analysis of non-coding mutations with key roles in tumorigenesis (Khurana et al., 2016). We therefore developed a method named TARGET-seq, which dramatically reduces ADO and also enables the efficient detection of non-coding mutations from the same single cell by allowing parallel, targeted mutation analysis of gDNA and cDNA alongside scRNA-seq.

## Results

### TARGET-Seq Dramatically Increases the Sensitivity of Mutation Detection in Single Cells

**Figure 1:**
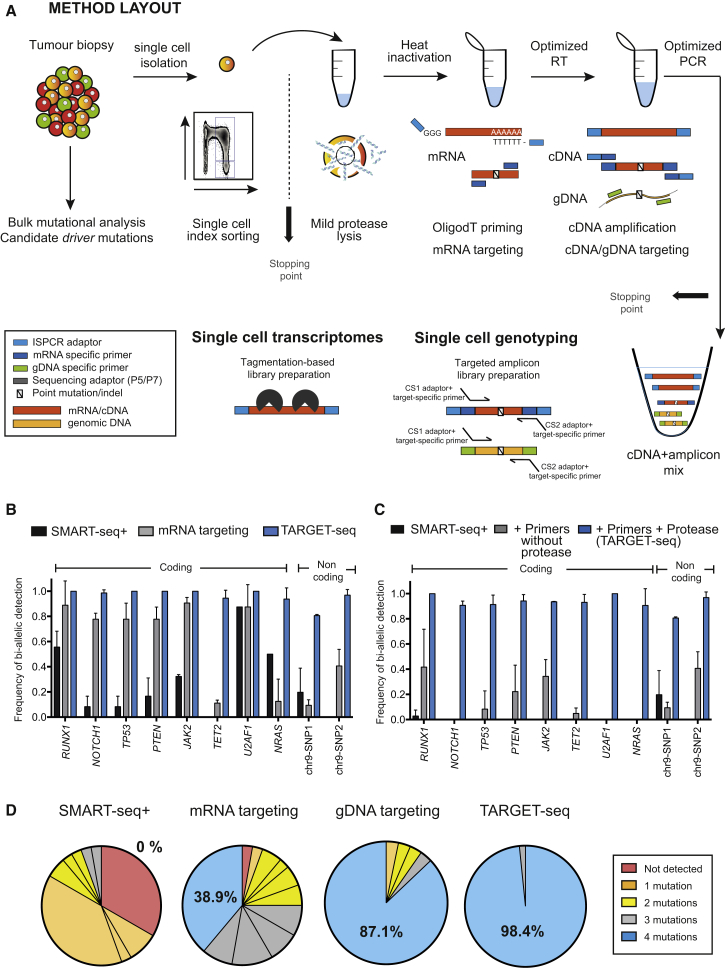
TARGET-Seq: A Method for High-Sensitivity Mutation Detection and Parallel Whole-Transcriptome Analysis from the Same Single Cell (A) Schematic representation of the method (full details are available in STAR Methods and Supplemental Experimental Procedures). In brief, cells were sorted into plates containing TARGET-seq lysis buffer; after lysis, protease was heat inactivated. RT mix was then added. OligodT-ISPCR primed polyadenylated mRNA and target-specific primers primed mRNA molecules of interest. During subsequent PCR, we used ISPCR adaptors to amplify polyA-cDNA, and we used target-specific cDNA and gDNA primers to amplify amplicons of interest. An aliquot of the resulting cDNA+amplicon mix was used for preparing the genotyping library and another aliquot for preparing the transcriptome library for scRNA-seq. (B) Frequency with which TARGET-seq detected heterozygous mutations in ten coding and non-coding regions in cell lines; this approach is compared to SMART-seq+ and mRNA targeting approaches (n = 376 cells, 2–3 independent experiments per amplicon; the bar graph represents mean ± SD). (C) Frequency of detection of heterozygous mutations for the same amplicons as in (B), showing exclusively results from targeted genomic DNA sequencing. The bar graph represents mean ± SD. (D) Frequency of detection of heterozygous mutations in JURKAT cells with SMART-seq+ (n = 36 cells), mRNA targeting (n = 36 cells), gDNA targeting (n = 62 cells), and TARGET-seq (n = 62 cells) when four different mutations (*RUNX1*, *NOTCH1*, *PTEN*, and *TP53*) in the same single cell were profiled in three independent experiments. Each slice of the pie chart represents a different combination of mutations, and each color represents the number of mutations detected per single cell.

In order to improve the detection of specific mRNA and gDNA amplicons, we extensively modified previously published template-switching protocols (Hedlund and Deng, 2018, Picelli et al., 2013, Zheng et al., 2018). To improve the release of gDNA, we modified the lysis procedure to include a mild protease digestion (Figure 1A and Table S1); we subsequently heat-inactivated the protease to avoid inhibition of the RT and PCR steps. Target-specific primers for cDNA and gDNA were added to the RT and PCR-amplification steps (Table S2), which also used modified enzymes (Table S1) that provided more efficient amplification (Figure 1A). We used an aliquot of the pre-amplified gDNA and cDNA libraries for targeted NGS of specific cDNA and gDNA amplicons and another aliquot for whole-transcriptome library preparation. The libraries used for targeted mutation analysis and those used for scRNA-seq were sequenced and analyzed independently.

In clonal cell lines, TARGET-seq dramatically improved the detection of ten mutation hotspots, including SNVs and small indels across both coding and non-coding regions (Figure 1B). Notably, gDNA amplicons alone achieved a mean 93% bi-allelic mutation and/or SNV detection (Figure 1C; the variant-calling pipeline and specific examples of variant calling can be found in Figures S2A and S2B, respectively). Importantly, mutational analysis from raw RNA-sequencing reads was impossible in almost all cells because of a lack of coverage (Figure S2C), despite the fact that the mean sequencing depth reached 2.93 million reads/cell.

We next tested whether TARGET-seq would improve the detection of combinations of mutations in single cells. We profiled four different mutations in a clonal T cell leukemia diploid cell line (JURKAT) carrying heterozygous mutations in *NOTCH1*, *RUNX1*, *TP53*, and *PTEN*. When we used SMART-seq+, detection of all of the four mutations within the same single cell was not achieved in any of the cells analyzed. mRNA targeting detected the four mutations in 38.9% of cells, gDNA targeting in 87.1% of cells, and TARGET-seq (combined mRNA+gDNA targeting) in 98.4% of cells (Figure 1D). Therefore, TARGET-seq provides extremely high sensitivity for the detection of multiple mutations in the same single cell, and this high sensitivity is essential for reliable reconstruction of tumor phylogenetic trees.

### TARGET-Seq Produces Unbiased Transcriptomic Readouts from Single Cells

**Figure 2:**
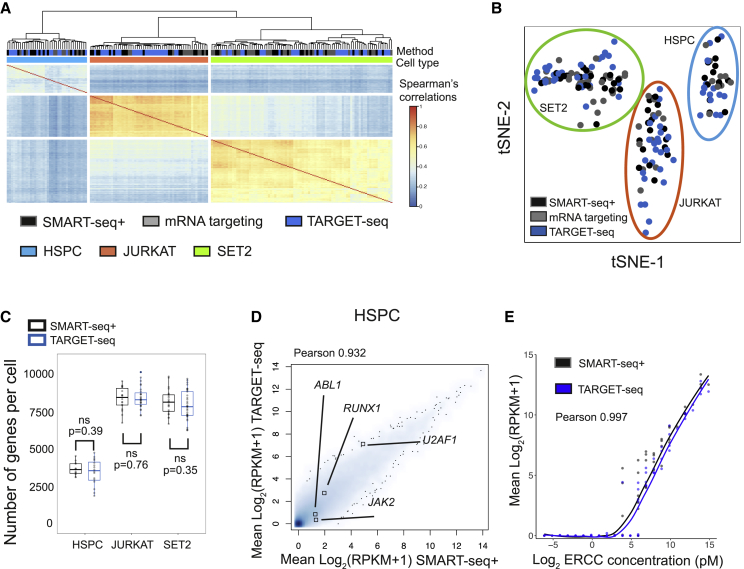
Unbiased Whole-Transcriptome Analysis of Single Cells with TARGET-Seq (A) Unsupervised hierarchical clustering of Spearman’s correlations from 180 single cells (JURKAT, n = 56; SET2, n = 86; and HSPC, n = 38); 4,088 highly variable genes were used. scRNA-seq libraries were generated with SMART-seq+, mRNA targeting, or TARGET-seq as indicated. (B) tSNE representation of HSPCs, SET2 cells, and JURKAT cells from (A); the same 4,088 highly variable genes as in (A) were used. (C) Number of detected genes per cell (RPKM ≥ 1) in HSPCs, SET2, and JURKAT cell lines from SMART-seq+ or TARGET-seq. “p” indicates the Student’s-t-test p value, and “ns” = non-significance. The boxes represent median and quartiles, and the dots represent the value for each individual cell. (D) Whole-transcriptome Pearson’s correlation between SMART-seq+ and TARGET-seq ensembles (mean RPKM values per condition) in HSPCs. The expression values for the genes targeted are highlighted. (E) Pearson’s correlation between mean ERCC spike-in expression values from SMART-seq+ and TARGET-seq in HSPCs per ERCC spike-in concentration.

To determine whether TARGET-seq introduces a bias in the single-cell whole-transcriptome data, we evaluated its performance in two cell lines (JURKAT and SET2) and in primary human HSPCs. Cells clustered by cell type and not by method (Figures 2A and 2B), and there were no significant differences in the number of genes detected between methods (Figure 2C). The sequencing quality controls (QCs; Figure S3A), numbers of cells passing QC (Figure S3B), and transcript coverage (Figure S3C) were comparable between SMART-seq+ and TARGET-seq, and there were good correlations of gene expression, including for genes selected for targeted amplification (Figures 2D, S3D, and S3E). Similarly, ERCC spike-in controls revealed high correlations between methods (Figures 2E, S3F, and S3G), and cDNA traces were comparable (Figures S3H–J). These results demonstrate that TARGET-seq allows accurate mutation detection with parallel, unbiased, and full-length (Figure S3C) scRNA-seq of the same single cell.

### The Stem Cell Compartment of Patients with MPN is Genetically and Transcriptionally Heterogeneous

**Figure 3:**
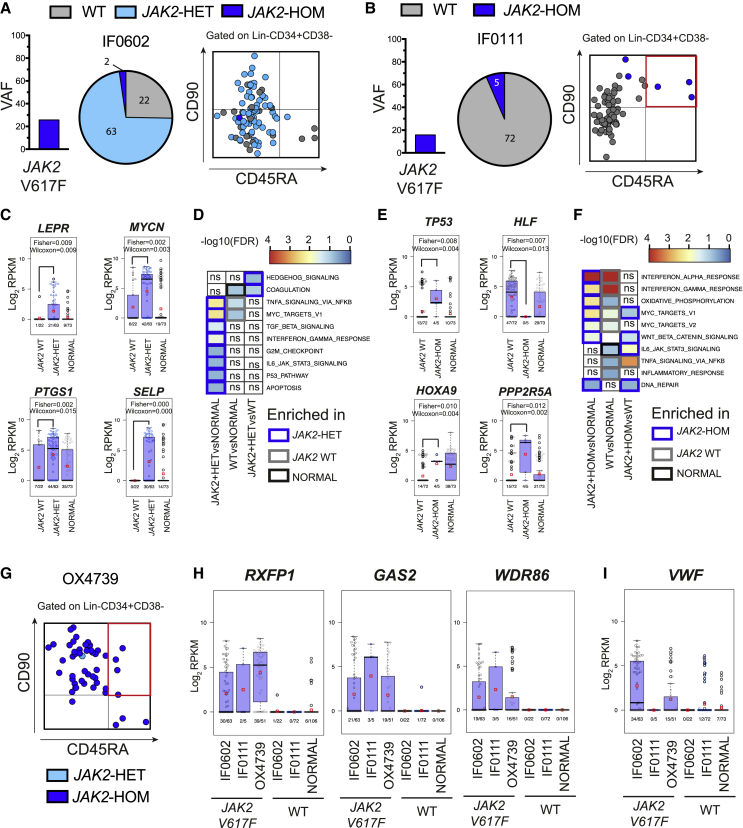
TARGET-Seq Reveals Genetic and Transcriptional Heterogeneity in the Stem-Cell Compartment of Patients with MPN (A and B) Variant allele frequency of *JAK2V617F* mutation (left), as identified by bulk sequencing of total MNCs; proportion of single cells that carry the mutation (including zygosity) in the Lin-CD34+CD38- compartment (center); and integration of index sorting with mutational information (right) for patients IF0602 (A) and IF0111 (B). (C–F) Analysis of disrupted gene expression associated with *JAK2V617F* mutation in HSPCs. Beeswarm plots show selected differentially expressed genes between (C) *JAK2* wild-type (WT) and *JAK2V617F*-heterozygous mutant cells from patient IF0602 or (E) *JAK2* WT and *JAK2V617F*-homozygous mutant cells from patient IF0111. Expression values for single cells from two normal donors (NORMAL) are also shown. Each dot represents the expression value for each single cell; red squares represent mean expression values for each group, and boxes represent median and quartiles. Fisher’s test and Wilcoxon test p values are shown on the top of each graph; expressing cell frequencies are shown on the bottom of each bar for each group. Table S4A (patient IF0602) and Table S4C (patient IF0111) show all significant, differentially expressed genes. (D) GSEA analysis of *JAK2* WT and *JAK2V617F*-heterozygous mutant cells from patient IF0602 or (F) *JAK2* WT and *JAK2V617F*-homozygous mutant cells from patient IF0111, as well as cells from normal donors (NORMAL). The heatmap represents –log10(FDR q-values) for each comparison, for which a FDR q-value cut-off < 0.25 was used; a white color with “ns” represents non-significance. The borders of each square of the heatmap are colored according to the group in which a particular pathway is enriched. Table S4B (patient IF0602) and Table S4D (patient IF0111) show results for all significant genesets tested. (G) Integration of index sorting with mutational information for patient OX4739. (H) Beeswarm plots of selected genes identified as biomarkers of *JAK2* mutant cells independently of the patient analyzed. Expression values across HSPCs from patients IF0602, IF0111, OX4739 (*JAK2* WT and *JAK2V617F* mutant cells shown separately), and two normal donors (NORMAL) are shown; expression frequencies are provided at the bottom of each graph for each group. (I) A Beeswarm plot of *VWF* expression values across HSPCs for the same patients and normal donors as in (H). Each dot represents the expression value for each single cell; red squares represent mean expression values for each group, and boxes represent the median and quartiles. Fisher’s test and Wilcoxon test p values are shown on the top of each graph; expressing cell frequencies are shown on the bottom of each bar for each group.

We next applied TARGET-seq to analyze 458 HSPCs in samples from five patients with myeloproliferative neoplasms (MPN); the samples carried different combinations of *JAK2V617F*, *EZH2*, and *TET2* mutations (Tables 1 and S3). Two normal donors were also included as controls. We isolated Lin^−^CD34^+^ cells via fluorescence-activated cell sorting (FACS) (Figure S4) and indexed the cells for CD38, CD90, CD45RA, and CD123 to allow assessment of clonal involvement in different stem and progenitor cell compartments (Majeti et al., 2007). All mutations identified in total mononuclear cells were also detected in single cells within the Lin^−^CD34^+^ compartment with TARGET-seq (Table S3), revealing subclonal mutations with striking inter-patient heterogeneity. This allowed us to determine the mutation acquisition order (Table S3B), which is of importance for MPN biology (Ortmann et al., 2015). For example, in patient SMD32316 (a patient with essential thrombocythemia; Tables 1 andS3), we could determine that a *TET2* mutation was acquired after the *JAK2V617F* mutation, whereas in patient OX2123 (a patient with myelodysplastic syndrome [MDS]/MPN overlap; Tables 1 and S3), a *TET2* mutation was acquired before a *JAK2V617F* mutation. In two patients with a similar *JAK2V617F* variant allele frequency (VAF) in bulk mononuclear cells (MNCs), the low percentage of ADO that was achieved by TARGET-seq analysis of single cells revealed that *JAK2V617F* was heterozygous in most Lin^−^CD34^+^CD38^−^ cells in patient IF0602 (a patient who had myelofibrosis [MF] and was receiving treatment with a JAK1/2 inhibitor; Table 1), and there was a normal distribution within the different Lin^−^CD34^+^CD38^−^ stem and progenitor fractions (Figure 3A). In contrast, in patient IF0111 (a patient who had polycythemia vera and was receiving interferon; Table 1), a lower fraction of clonally involved Lin^−^CD34^+^CD38^−^ cells were homozygous for *JAK2V617F* and predominantly had a CD90^+^CD45RA^+^ aberrant phenotype (Figure 3B) that has also been reported in other myeloid malignancies (Dimitriou et al., 2016). The ability to reliably distinguish heterozygous versus homozygous *JAK2V617F* mutations is of considerable importance for MPN biology (Li et al., 2014) and also, more broadly, in cancer because a mutant-allele-specific imbalance is common during disease progression (Soh et al., 2009).

**Table 1:** Summary of Donors in the Study, Mutation Status, and Clinical Characteristics

Sample Code	Mutation(s)	Donor Type	Diagnosis	Treatment	Figures
HD7643	–	normal donor	–	NA	Figures 3C–F, 3H, 3I, 4A–E, and S4A
HD7650	–	normal donor	–	NA	Figures 3C–F, 3H, 3I, and 4A–E
Aph1	–	normal donor	–	NA	Figures 5A–G and 5I–K
HD85	–	normal donor	–	NA	Figures 5A–G and 5I–K
SMD32316	JAK2 p.Val617Phe, TET2 p.Gln958Ter	patient	ET	aspirin	Figures 4A–C and 4E
IF0111	JAK2 p.Val617Phe	patient	PV	pegylated IFN alpha-2a	Figures 3B, 3E, 3F, 3H, 3I, 4A–C, and 4E
OX4739	JAK2 p.Val617Phe	patient	myelofibrosis (PMF)	ruxolitinib (JAK1 and JAK2 inhibitor)	Figures 3G–I, 4C, and 4E
OX2123	JAK2 p.Val617Phe, EZH2 p.Glu249AsnfsTer16, TET2 c.3409+1G>C	patient	MDS/MPN overlap with grade 3 bone marrow fibrosis	none	Figures 4C, 4D and S4B
IF0602	JAK2 p.Val617Phe	patient	myelofibrosis (PMF)	momelotinib (JAK1 and JAK2 inhibitor)	Figures 3A, 3C, 3D, 3H, 3I, 4A–C, and 4E (full length TARGET-seq); and Figures 5A–K (3'-TARGET-seq)
IF0155	JAK2 p.Val617Phe	patient	myelofibrosis (post-ET)	anagrelide	Figures 5A–K
IF0157	JAK2 p.Val617Phe	patient	myelofibrosis (post-PV)	ruxolitinib 10 mg BD (JAK1 and JAK2 inhibitor)	Figures 5A–K
IF0140	JAK2 p.Val617Phe, TET2 p.Ser1612LeufsTer4	patient	myelofibrosis (post-PV)	ruxolitinib 20 mg BD (JAK1 and JAK2 inhibitor)	Figures 5A–C
IF0101	JAK2 p.Val617Phe, CBL p.Cys404Tyr, SRSF2 p.Pro95His	patient	myelofibrosis (PMF)	ruxolitinib 10 mg BD (JAK1 and JAK2 inhibitor)	Figures 5A–C, 6E, 6F, S6, S7C, S7F, S7I, S7L, and S7O
IF0123	JAK2 p.Val617Phe, SF3B1p.Lys666Asn	patient	myelofibrosis (PMF)	ruxolitinib 5 mg BD (JAK1 and JAK2 inhibitor)	Figures 5A–G and S6
IF0138	JAK2 p.Val617Phe, ASXL1 p.Gly646TrpfsTer12, ASXL1 p.Gly644TrpfsTer12	patient	myelofibrosis (post-PV)	hydroxycarbamide	Figures 5A–K, 6C, 6D, S6, S7B, S7E, S7H, S7K, and S7N
IF0137	JAK2 p.Val617Phe, U2AF1 p.Gln157Arg, TET2 p.Ile1105MetfsTer8, ASXL1 p.Gln910AlafsTer13, ASXL1 p.Trp898ArgfsTer5	patient	myelofibrosis (PMF)	none	Figures 5A–G, 6A, 6B, S6, S7A, S7D, S7G, S7J, and S7M

Additional clinical details are shown in Table S3. PMF, primary myelofibrosis; MDS, myelodysplastic syndrome; MPN, myeloproliferative neoplasm; ET, essential thrombocythemia; PV, polycythemia vera.

TARGET-seq analysis uniquely allowed wild-type (WT) HSPCs to be reliably distinguished from *JAK2V617F* mutant cells in the same samples. The analysis revealed the aberrant expression of biologically relevant genes such as *LEPR* (Jiang et al., 2008) and oncogenes such as *MYCN*, *TP53*, or *PPP2R5A,* as well as biologically relevant pathways, including upregulation of hedgehog (Figure 3D) and Wnt β-catenin (Figure 3F) pathway-associated transcription (Table S4), in heterozygous (Figures 3C and 3D) and homozygous (Figures 3E and 3F) *JAK2V617F*-mutated HSPCs. HSPCs from patient IF0111 also showed dysregulation of interferon-associated gene expression, consistent with the patient receiving treatment with interferon (Figure 3F and Table 1). The CD90^+^CD45RA^+^ aberrant phenotype was also present at a similar low frequency in an additional patient with a homozygous *JAK2* mutation (Figure 3G; patient OX4739, an MF patient receiving JAK1/2 inhibitor treatment). Cells from patient OX4739 also showed disrupted expression of a number of the same genes identified in patient IF0111 (Table S4E).

Importantly, this analysis allowed us to identify candidate biomarkers for *JAK2V617F* mutations in HSPCs from patients with an isolated *JAK2* mutation (Figure 3H; *RXFP1*, *GAS2*, and *WDR86*). Interestingly, *VWF*, a marker of platelet-biased stem cells (Sanjuan-Pla et al., 2013), was specifically upregulated in *JAK2V617F* mutant cells from patients IF0602 and OX4739, whose disease was characterized by abnormal megakaryocytic differentiation and MF, but it was not upregulated in *JAK2V617F* mutant cells from patient IF0111, who had a polycythemia phenotype (Figure 3I). These data support the notion that transcriptional lineage priming in the HSPC compartment might be linked to the disease phenotype in MPN.

### Distinct Genetic Subclones Present Unique Transcriptional Signatures

**Figure 4:**
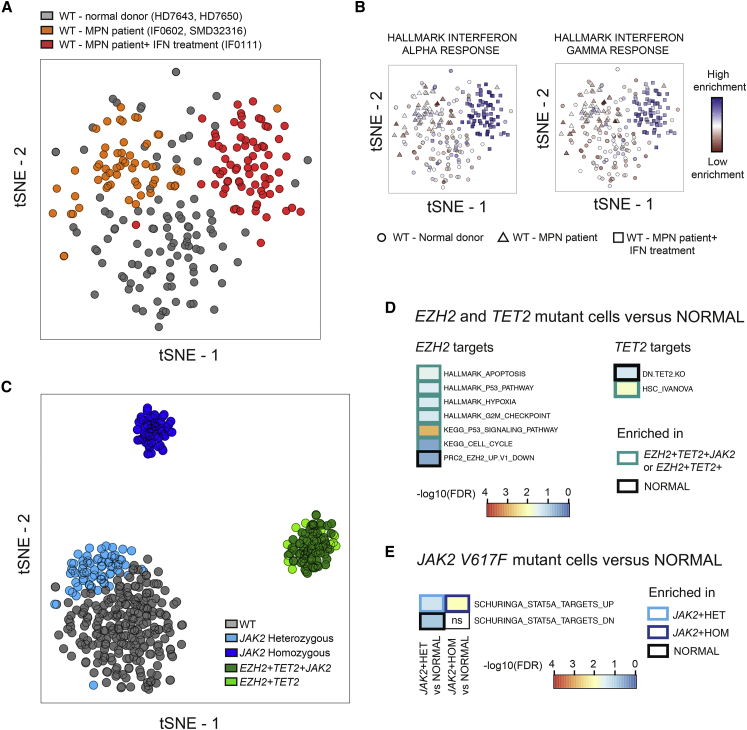
TARGET-Seq Reveals Distinct Transcriptional Signatures Associated with the Presence or Absence of Somatic Mutations in Single HSPCs (A) tSNE representation of 236 wild-type (WT) HSPCs from the three samples (from patients IF0602, SMD32316, and IF0111) in which WT cells are present, and cells from two normal donors (donors HD7650 and HD7643); 5,365 highly variable genes were used. Cells from normal donors are colored in gray, and cells from patients with MPN are colored in orange (patients SMD32316 and IF0602) or red (patient IF0111; patient treated with interferon). (B) Enrichment of IFN-α (left) or IFN-γ (right) signaling gene signatures as a projection of ssGSEA results at the same tSNE coordinates from the cells of the specific donors or patients shown in (A). Each shape represents a group of donors. (C) tSNE representation of 448 HSPCs from five patients and two normal controls; the top 2,000 genes as measured by the Gini index from the random forest analysis were used. Only genotypes present in at least five cells were analyzed. The gene expression matrix was batch- and donor-corrected, and genotypes were preserved. (D and E) Enrichment of *EZH2*-related pathways, *TET2*-related pathways (D), or the JAK/STAT pathway (E) in cells carrying mutations in these genes compared to (n = 106) cells from two normal donors. The heatmap represents –log10(FDR q-values) for each comparison, using a FDR q-value cut-off < 0.25. A complete list of all significant genesets tested can be found in Tables S4F and S4G, and a summary list of all genesets can be found in Table S4H.

TARGET-seq also uniquely allowed comparison of WT cells from patients’ samples and normal controls. Intriguingly, this analysis established that WT HSPCs from patients with MPN were transcriptionally distinct from normal donor HSPCs (Figure 4A) and showed enrichment of inflammatory pathways associated with tumor necrosis factor α (TNFα) and interferon (IFN) signaling (Figures 3D, 3F, and 4B). These results might indicate the MPN microenvironment’s effects on the wild-type cells from the same patient; a similar finding was demonstrated to have clinically predictive value in chronic myeloid leukemia (Giustacchini et al., 2017). Interestingly, WT HSPCs from patient IF0111, who was receiving interferon treatment, also showed strong IFN signaling signatures, thus providing an additional layer of validation for the transcriptional signatures obtained (Figures 3F and 4B).

Using the top 2,000 genes identified by random forest analysis (Figure 4C), we analyzed combinations of mutations and showed striking clustering of HSPCs of the same genotype from multiple different patients. HSPCs carrying mutations in epigenetic modifiers had a highly distinct transcriptomic signature, whereas the signature of cells carrying only heterozygous *JAK2V617F* mutations more closely resembled the transcriptome of WT cells (Figure 4C). *EZH2* mutant cells showed enrichment in pathways such as apoptosis, P53 signaling, hypoxia, and the cell cycle (Figure 4D and Table S4F) previously identified to be correlated with loss of PRC2 function (Xie et al., 2014) and negative enrichment in genes downregulated upon *EZH2* knockdown (Table S4F). *TET2* mutant cells also showed enrichment in HSC-related genes and a negative enrichment in genes downregulated upon *TET2* knockout (Zhang et al., 2016) (Figure 4D and Table S4F). Moreover, *JAK2V617F* cells showed dysregulation of *STAT5A* targets (Figure 4E and Table S4G). Taken together, these data demonstrate that TARGET-seq reveals distinct and biologically relevant molecular signatures of HSPC subclones in MPN and represents a powerful tool for biomarker and therapeutic target discovery.

### High-Throughput 3′-TARGET-Seq Resolves Complex Clonal Hierarchies in *JAK2* Mutant Myelofibrosis

To increase the throughput of the technique, we adapted TARGET-seq to allow barcoding and pooling of scRNA-seq libraries in a 384-well format in reduced reaction volumes, generating 3′-biased libraries (Table S1C and Figure S5A). Barcodes could be reliably detected (Figure S5B), sequencing quality metrics were in line with other 3′-biased scRNA-seq methods (Paul et al., 2015, Velten et al., 2017) (Figure S5C), and transcript coverage was 3′ biased (Figure S5D). We then analyzed 2,798 cells from a cohort of eight patients with MF and two age-matched normal donors (Tables 1 and S3). TARGET-seq genotyping provided very low dropout rates, in stark contrast to cDNA genotyping alone (Figures S6A and S6B). This allowed reconstruction of clonal hierarchies in these patients at unprecedented scale and resolution (Figures S6B and S6C and Table S3). Considerable inter-patient heterogeneity was observed, and there were both linear and branching patterns of clonal evolution (Figure S6C). Spliceosome mutations were an early event in these patients; in contrast, *ASXL1* mutations were acquired late, and there were also multiple *ASXL1* mutations acquired independently in patient IF0137 (Figures S6B and S6C and Table S3).

**Figure 5:**
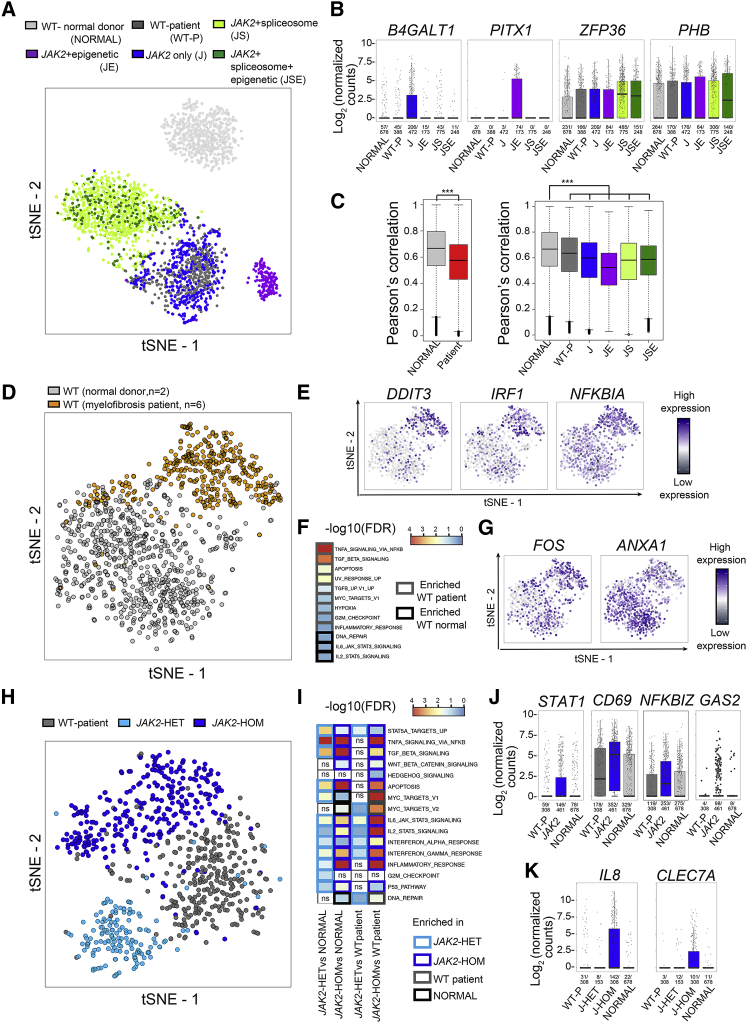
High-Throughput TARGET-Seq Identifies Molecular Signatures of Genetic Subclones in HSPCs from *JAK2-V617F* Mutant Myelofibrosis (A) tSNE representation of 2,734 HSPCs from eight patients and two age-matched normal donors; the samples were processed with 3′-TARGET-seq, and 3,286 highly variable genes were used for the analysis. Cells from age-matched normal donors are colored in light gray (NORMAL). Wild-type (WT) cells from patients with MF are colored in dark gray (“WT-P”). Cells carrying mutations exclusively in *JAK2* are colored in blue (“J”); those carrying mutations in *JAK2* and epigenetic modifiers (*TET2* and *ASXL1*) are colored in purple (“JE”); those carrying mutations in *JAK2* and spliceosome components (*SF3B1*, *SRSF2*, and *U2AF1*) are colored in light green (“JS”); and those carrying mutations in *JAK2*, spliceosome components, and epigenetic modifiers are colored in dark green (“JSE”). The gene expression matrix was batch- and donor-corrected, and genotypes were preserved. (B) Boxplots of representative differentially expressed genes from *JAK2* only (*B4GALT1*), *JAK2*+epigenetic (*PITX1*), *JAK2*+spliceosome (*ZFP36*), or *JAK2*+spliceosome+epigenetic (*PHB* and *ZFP36*) genetic subclones. Each dot represents the expression value for each single cell; boxes represent median and quartiles, and the central line represents the median for each group. Expression frequencies are shown on the bottom of each bar for each group. (C) Boxplot of overall Pearson’s correlation of cells from normal donors and cells from MF-patient samples; the cells are grouped per donor type (normal donor or patient sample; left panel) or by the genotype groups presented in (A) (right panel). A Kolmogorov-Smirnov test provided the significance level for each comparison (^∗∗∗^; p value < 0.001). (D) tSNE representation of 1,066 WT cells from six patients and two normal donors; 3,436 highly variable genes were used. The gene expression matrix was batch-corrected, and the donor effect was preserved. (E) tSNE projection (from the same cells as in [D]) representing relative gene expression levels from selected differentially expressed inflammation-associated genes in WT cells from patients and normal donors. (F) Enrichment of selected pathways in the same WT cells from the same samples as in (D) and (E) from normal donors and patients. A complete list of all significant genesets tested can be found in Table S5A. (G) tSNE projection representing relative gene expression levels from selected differentially expressed oncogenes (*FOS*) and tumor suppressors (*ANXA1*) between the same WT cells from patients and normal donors as in (D). (H) tSNE representation of 769 WT and *JAK2*-only mutant HSPCs from four patients with MF (patients IF0138, IF0155, IF0157, and IF0602); we used the top 2,000 genes as identified by the Gini index from random forest analysis. (I) Enrichment of selected HALLMARK and STAT5A pathways from the same cells as in (H), as well as cells from normal donors (NORMAL). A complete list of all significant genesets tested can be found in Tables S5B and S5C, and specific comparisons for subclones within patients can be found in Table S5D. (J and K) Analysis of disrupted gene expression associated with *JAK2V617F* mutation in HSPCs. Boxplots show selected differentially expressed genes specifically upregulated in *JAK2* mutant cells independently of zygosity (J) or exclusively in *JAK2*-homozygous cells (K). Each dot represents the expression value for each single cell; boxes represent median and quartiles, and the central line represents the median for each group. Expressing-cell frequencies are shown on the bottom of each bar for each group. A complete list of all significant differentially expressed genes and associated p values can be found in Table S5E. The heatmaps are colored according to –log10(FDR q-values) for each comparison, for which an FDR q-value cut-off < 0.25 was used. The borders of each square of the heatmap are colored according to the group in which a particular pathway is enriched; a white color with “ns” represents non-significance.

T-SNE analysis using 3,286 highly variable genes showed distinct clusters of MF HSPCs according to their genotype (Figure 5A). HSPCs carrying mutations in spliceosome components or epigenetic modifiers in addition to *JAK2* clustered separately from WT HSPCs, including WT cells from the same patients, and were also distinct from cells carrying a *JAK2* mutation alone. TARGET-seq allowed the identification of specific gene expression associated with certain genetic subclones of HSPCs. For example, cells carrying mutations exclusively in *JAK2* specifically upregulated *B4GALT1* (Figure 5B), which is associated with acquisition of drug resistance in leukemia (Zhou et al., 2013), and cells with mutations in epigenetic modifiers specifically upregulated *PITX1*, which has been previously implicated in leukemogenesis (Nagel et al., 2011). *ZFP36* (also known as *TTP*), which modulates the interferon-induced inflammatory response (Sauer et al., 2006), was upregulated in cells carrying mutations in spliceosome components. Cells carrying mutations in spliceosome and epigenetic genes upregulated *PHB*, a proposed therapeutic target in leukemia (Pomares et al., 2016). MF HSPCs also showed more transcriptional diversity, including within genetically defined subclones, than WT counterparts (Figure 5C), suggesting that this transcriptional heterogeneity is not driven by genetic heterogeneity alone (Figure 5C). Normal donor HSPCs also clustered separately from WT HSPCs from MF patients (Figure 5D), an observation similar to that made by full-length TARGET-seq. Differences between normal donor and MF WT HSPCs included dysregulation of specific genes and gene signatures associated with inflammation, as well as TNFα and TGFβ signaling (Figures 5E and 5F and Table S5). Furthermore, a number of oncogenes and tumor suppressors were aberrantly expressed in WT HSPCs from MF patients (Figure 5G), raising the possibility that these cells might be more susceptible to malignant transformation and the development of secondary hematopoietic malignancy.

Specific analysis that compared only *JAK2* mutant and WT cells and used the top 2,000 genes identified by random forest analysis showed specific clustering of WT, *JAK2V617F*-heterozygous, and *JAK2V617F*-homozygous cells (Figure 5H). *JAK2*V617F-heterozygous cells showed enrichment in inflammation-related signatures such as TNFα, TGF β, and IFN signaling; the G2M checkpoint; and the P53 pathway (Figure 5I), further validating the pathways previously identified by full-length TARGET-seq in specific patients (Figure 3). *JAK2*V617F-homozygous mutant cells showed enrichment in WNT β-catenin, hedgehog signaling, and apoptosis, as well as in inflammation-related signatures (Figure 5I). The distinct clustering we observed was driven by a number of the same genes identified by full-length TARGET-seq, e.g., *GAS2* and *RXFP1* (Figure 5J and Table S5); we also identified a number of additional genes (*STAT1*, *CD69*, and *NFKBIZ* [Figure 5J and Table S5]), some of which were specifically upregulated in *JAK2*-homozygous but not *JAK2*-heterozygous mutant cells (*IL8* and *CLEC7A* [Figure 5K]).

### Transcriptional Differences between Genetic Subclones within Individual Patients Are Identified with TARGET-Seq

**Figure 6:**
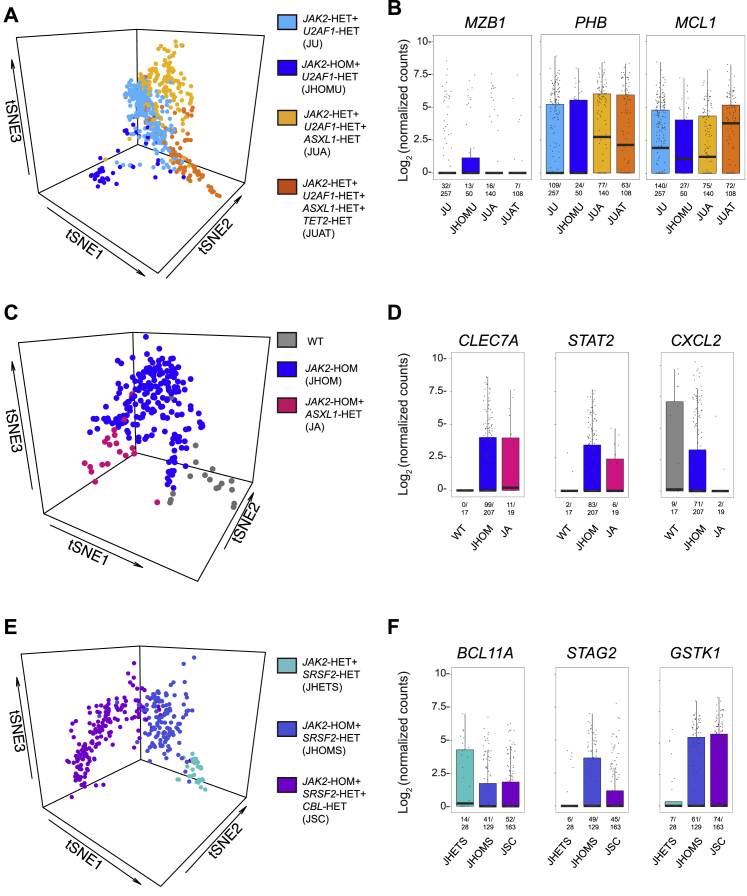
TARGET-Seq Resolves Genetic and Transcriptional Heterogeneity of HSPCs within Individual Myelofibrosis Patients (A and B) Distinct transcriptional signatures of genetic subclones identified by TARGET-seq in patient IF0137. (A) tSNE representation of 555 cells; 633 differentially expressed genes identified with ANOVA were used and (B) boxplots of selected differentially expressed genes between each genetic subclone from the same cells as in (A). Genetic subclones carrying *JAK2*, *U2AF1*, and *ASXL1* (p897/p910) mutations from patient IF0137 are labeled JAK2-*HET*+U2AF1-*HET*+ASXL1-*HET* and were analyzed together as indicated. Each genetic subclone is colored and labeled according to the legend provided in (A). (C and D) Distinct transcriptional signatures of genetic subclones from patient IF0138. (C) tSNE representation of 243 cells; 418 differentially expressed genes identified with ANOVA were used. (D) Boxplots of selected differentially expressed genes between distinct genetic subclones. Each genetic subclone is colored according to the legend provided in (C). (E and F) Distinct transcriptional signatures of genetic subclones from patient IF0101. (E) tSNE representation of 320 cells; 500 differentially expressed genes identified with ANOVA were used. (F) Boxplots of selected differentially expressed genes between distinct genetic subclones. Each genetic subclone is colored according to the legend provided in (E). Each dot represents the expression value for each single cell; boxes represent median and quartiles, and the central line represents the median for each group. Expressing cell frequencies are shown on the bottom of each bar for each group. The list of differentially expressed genes identified in each patient and associated p values for each comparison can be found in Table S6. Only genetic subclones representing at least 5% of the total cells for each patient are included in the analysis.

Finally, we explored whether distinct genetic subclones of HSPCs in individual patients could be identified with TARGET-seq. We analyzed three patients with complex clonal hierarchies (at least three genetic subclones [Figure S6]): patients IF0137 (Figures 6A and 6B), IF0138 (Figures 6C and 6D), and IF0101 (Figures 6E and 6F). Each genetic subclone clustered separately (Figures 6A, 6C, and 6E) and showed transcriptional differences driven by pro-apoptotic genes (*MCL1* [Figure 6B and Table S6]), JAK2-STAT signaling (*STAT2* [Figure 6D and Table S6]), chemokines (*CXCL2* [Figure 6D and Table S6), and genes previously implicated in leukemogenesis (*PHB*, *BCL11A*, and *STAG2* [Figures 6B and 6F and Table S6) or drug resistance (*GSTK1* [Figure 6F and Table S6).

We then explored whether the same genetic subclones could have been identified by common dimensionality reduction or clustering methods. Dimensionality reduction using highly variable genes (Figures S7A–C) did not identify distinct clustering patterns associated with genetic subclones in patients IF0137, IF0138, or IF0101 either when we regressed out the effect of the cell-cycle phase (Figures S7D–F) or when we specifically modeled zero inflation (Figures S7G–I) (Pierson and Yau, 2015). Furthermore, genetic subclones could not be identified with a recently published single-cell K-means clustering method (SC3) (Kiselev et al., 2017) previously reported to specifically distinguish genetically distinct subclones of cells (Figures S7J–L); they also could not be identified with the KNN-based clustering implemented in the PAGODA2 package (Figures S7M–O). Distinct genetic subclones from the same patient were, however, robustly identified by dimensionality reduction when we used genes that were differentially expressed between different genetic subclones, the identification of which was made possible by TARGET-seq (Figure 6).

## Discussion

With the advent of molecularly targeted therapy in cancer (Longo, 2017), clinical remissions and clonal responses can be readily achieved in many patients. However, relapse frequently occurs, and it is often associated with evidence of clonal evolution, most likely reflecting ITH already present at diagnosis (Smith et al., 2017) and a differential response to the targeted therapy in distinct tumor subclones. Therefore, it is crucial to resolve the clonal heterogeneity of tumors and dissect the transcriptional heterogeneity associated with the responsive and resistant subclones of cancer cells. Although scRNA-seq offers great potential to resolve the transcriptomic signatures of tumor subclones, up to now it has not been possible to correlate scRNA-seq data with mutation analysis because of the lack of coverage for small indels or point mutations in the scRNA-seq reads, although large chromosomal aberrations can be detected (Tirosh et al., 2016a). For example, in a recent study of gliomas, from 22 mutations analyzed, reads spanning the position of the mutations were detected in 0.4% to 8.7% of the cells (Tirosh et al., 2016b). Although methods for the parallel sequencing of the whole-transcriptome and whole-genome of single cells have previously been reported, these methods are not well suited for high-sensitivity mutation detection because of high ADO rates (Dey et al., 2015, Macaulay et al., 2015). Furthermore, these approaches are relatively costly because of the requirement for whole-genome amplification. Consequently, up to now, such techniques have not been widely used for the analysis of cancerous tissues.

We herein report a single-cell RNA sequencing and genotyping method that provides a simple, easily implementable, and customizable protocol for high-sensitivity mutation detection with parallel, unbiased whole-transcriptome analysis. TARGET-seq has clear advantages above other available scRNA-seq methodologies and provides improved complexity of scRNA-seq libraries and a dramatically improved ability to detect multiple mutations in the same single cell, primarily attributable to the detection of gDNA variants through modified cell lysis and high-sensitivity, targeted amplification. The high sensitivity for bi-allelic detection of mutations provided by our technique is also of considerable importance as loss of heterozygosity of a number of different mutations is an important driver of disease phenotype as well as therapy response (Kharazi et al., 2011). This is also demonstrated in our analysis of patients with MPN; this analysis shows clear transcriptional differences between *JAK2*-heterozygous and homozygous HSPCs in multiple patients. TARGET-seq also allowed analysis of the order of acquisition of mutations, which is of importance in cancer biology (Ortmann et al., 2015). Moreover, TARGET-seq has the advantage of combining scRNA-seq data and mutational analysis with index sorting, allowing cells to be traced back to canonical stem and progenitor cell hierarchies. This revealed an aberrant HSPC phenotype associated with the presence of a *JAK2*-homozygous mutation in patients with MPN. Furthermore, the reliable identification of WT cells by TARGET-seq allows analysis of aberrant gene expression in normal tissue-residing cells; such aberrant expression might reflect cell-extrinsic phenomena. Such microenvironmental factors might underlie many aspects of tumor biology and therapy response.

TARGET-seq is adapted to allow both full-length and 3′-biased scRNA-seq approaches. The throughput of the full-length technique would typically enable the preparation of approximately 400 cells per week and thousands of cells within a few months; this amount is in line with the numbers of cells analyzed in published scRNA-seq tumor datasets (Giustacchini et al., 2017, Tirosh et al., 2016a, Tirosh et al., 2016b). This version of the protocol generates scRNA-seq libraries of high complexity and sensitivity for detecting low-level expressed genes. Moreover, it allows analysis of alternative splicing patterns; this is of importance in cancer biology (David and Manley, 2010), as well as in many other diseases (Cooper et al., 2009), particularly because components of the spliceosome machinery are recurrently mutated in cancer (Kandoth et al., 2013).

Higher-throughput scRNA-seq techniques are available (Macosko et al., 2015, Zheng et al., 2017); these typically provide shallow coverage of only the 3′ or 5′ region of transcripts and lower molecular capture rates but enable the analysis of larger numbers of cells. Therefore, we also developed 3′-biased TARGET-seq to allow higher-throughput analysis. 3′-TARGET-seq is associated with shallower coverage than full-length TARGET-seq, reducing sequencing costs, but it retains high-sensitivity mutation analysis at the single-cell level. 3′-TARGET-seq is mostly automated, and the process would typically allow 1,000 cells to be processed per week and tens of thousands to be processed within a few months, considerably increasing the throughput of the technique. In a cohort of patients with MF, this approach revealed complex clonal hierarchies and marked inter-patient variability that was not apparent from bulk genetic analysis. This allowed distinct transcriptional signatures of specific genetic subclones and non-clonally involved WT HSPCs to be characterized, which was not possible with other computational approaches.

In summary, TARGET-seq is a powerful tool for resolving both genetic and transcriptional intratumoral heterogeneity. TARGET-seq also uniquely allows the identification of specific molecular signatures within genetically distinct subclones of tumor cells. We expect that this will pave the way for the application of scRNA sequencing for the definitive analysis of intratumoral heterogeneity and the identification and characterization of therapy-resistant tumor subclones.

### Limitations

A potential limitation of TARGET-seq is that this approach does not support mutation discovery and relies on the analysis of known driver mutations or mutations previously identified by other discovery-type methods. However, because the lysate is initially frozen and stored, this will routinely allow for mutational analysis of the same sample before the subsequent processing of single cells. Up to now, we have multiplexed primers to detect a total of 12 different mutations per single cell. Although this will be adequate for analyzing key driver mutations in many tumors, for more genetically complex malignancies, a more complex multiplexing strategy might be required. For very genetically complex tumors where potentially hundreds of different mutations need to be tracked, a whole-genome and whole-transcriptome approach might be more appropriate (Dey et al., 2015, Macaulay et al., 2015), albeit at the cost of reduced sensitivity for the detection of those mutations (Hosokawa et al., 2017, Wang et al., 2014). In the current study, we have applied this technique to analyze hematopoietic tumors; however, this method could be broadly applied to the analysis of a range of cancers and is a powerful tool for linking transcriptional signatures with genetic tumor heterogeneity.

## STAR★Methods

### Key Resources Table

**Table:** 

REAGENT or RESOURCE	SOURCE	IDENTIFIER
**Antibodies**		

CD8-FITC (Lineage)	BioLegend	Clone: RPA-T8 Cat#: 301006 RRID: AB_314124
CD20-FITC (Lineage)	BioLegend	Clone: 2H7 Cat#: 302304 RRID: AB_314252
CD66b-FITC (Lineage)	BioLegend	Clone: G10F5 Cat#: 305104 RRID: AB_314496
CD10-FITC (Lineage)	BioLegend	Clone: HI10a Cat#: 312208 RRID: AB_314919
CD127-FITC (Lineage)	eBioscience	Clone eBioRDR5; Cat#: 11-1278-42 RRID: AB_1907343
Human Hematopoietic Lineage Cocktail – FITC (Lineage)	eBioscience	Cat**#** 22-7778-72; RRID: AB_1311229
CD123-PECy7	BioLegend	Clone: 6H6 Cat#: 306010 RRID: AB_493576
CD38-PETxRed	Invitrogen	Clone: HIT2 Cat#: MHCD3817 RRID: AB_10392545
CD90-BV421	BioLegend	Clone: 5E10 Cat#: 328122 RRID: AB_2561420
CD45RA-PE	eBioscience	Clone: HI100 Cat#: 12-0458-41 RRID: AB_10717397
CD34-APC-eF780	eBioscience	Clone: 4H11 Cat#: 47-0349-42 RRID: AB_2573956
CD34-PerCP/Cy5.5	BioLegend	Clone: 562 Cat# 343611, RRID:AB_2566787
CD90-PE	BioLegend	Clone: 5E10 Cat# 328109 RRID: AB_893442
CD45RA-FITC	Invitrogen	Clone: MEM56 Cat# MHCD45RA01 RRID: AB_10373858
CD2-PE/Cy5 (Lineage)	BioLegend	Clone: RPA-2.10 Cat# 300209 RRID:AB_314033
CD3-PE/Cy5 (Lineage)	BioLegend	Clone: HIT3a Cat# 300310 RRID: AB_314046
CD4-PE/Cy5 (Lineage)	BioLegend	Clone: RPA-T4 Cat# 300510 RRID: AB_314078
CD7-PE/Cy5 (Lineage)	BioLegend	Clone: 6B7 Cat# 343110 RRID: AB_2075096
CD8-PE/Cy5 (Lineage)	BioLegend	Clone: RPA-T8 Cat# 301010 RRID: AB_314128
CD10-PE/Cy5 (Lineage)	BioLegend	Clone: HI10a Cat# 312206 RRID: AB_314917
CD11b-PE/Cy5 (Lineage)	BioLegend	Clone: ICRF44 Cat# 301308 RRID: AB_314160
CD14-PE/Cy5 (Lineage)	Invitrogen	Clone: 61D3 Cat# 15-0149-41 RRID: AB_2573057
CD19-PE/Cy5 (Lineage)	BioLegend	Clone: HIB19 Cat# AB_314240 RRID: 302210
CD20-PE/Cy5 (Lineage)	BioLegend	Clone: 2H7 Cat# AB_314256 RRID: 302308
CD56-PE/Cy5 (Lineage)	BD Biosciences	Clone: B159 Cat# 555517 RRID: AB_395907
CD235a,b-PE/Cy5 (Lineage)	BioLegend	Clone: HIR2 Cat# 306606 RRID: AB_314624

**Biological Samples**		

Healthy Donors (HD7643; HD7650; Aph1; HD85) and MPN patient samples (OX2123; IF0602; IF0111; SMD32316; OX4739; IF0101; IF0123; IF0137; IF0138; IF0140; IF0155; IF0157; See Table 1 and Table S3)	INForMeD Study (REC:199833, University of Oxford)	https://www.hra.nhs.uk/planning-and-improving-research/application-summaries/research-summaries/the-informed-study/

**Chemicals, Peptides, and Recombinant Proteins**		

Protease	QIAGEN	Cat# 19155
RNase Inhibitor	Takara (Clontech)	Cat# 2313A
SMARTScribe	Takara (Clontech)	Cat# 639537
SeqAMP	Takara (Clontech)	Cat# 638509

**Critical Commercial Assays**		

Nextera XT DNA Library Preparation Kit	Illumina	Cat# FC-131-1096
Nextera XT Index Kit v2 Set A	Illumina	Cat# FC-131-2001
Nextera XT Index Kit v2 Set C	Illumina	Cat# FC-131-2003
KAPA 2G Robust HS PCR Kit	Kapa Biosystems	Cat# KK5517
FastStart High Fidelity PCR System, dNTPack - Sigma-Aldrich	Roche	Cat# 04-738-292 001
Access Array™ Barcode Library for Illumina® Sequencers-384, Single Direction	Fluidigm	Cat# 100-4876

**Deposited Data**		

Single-cell RNA sequencing	this paper	GEO: GSE105454
Targeted genotyping sequencing (validation; Figure 1)	this paper	SRA: PRJNA503734
Targeted genotyping sequencing (patients processed using full-length TARGET-seq; Figure 3, Figure 4)	this paper	SRA: PRJNA503736
Targeted genotyping sequencing (patients processed using 3′-TARGET-seq; Figures 5 and 6; Figures S6 and S7)	this paper	SRA: PRJNA503628

**Experimental Models: Cell Lines**		

K562	ATCC	RRID:CVCL_0004
MOLT4	ATCC	RRID:CVCL_0013
NALM6	DSMZ	RRID:CVCL_0092
SET2	Laboratory of Dr. Jacqueline Boultwood	RRID:CVCL_2187
JURKAT	ATCC	RRID:CVCL_0367

**Oligonucleotides**		

OligodT-ISPCR (HPLC purification): aagcagtggtatcaacgcagagtacttttttttttttttttttttttttttttttvn	Picelli et al., 2013	N/A
TSO-LNA (RNase Free HPLC purification): AAGCAGTGGTATCAACGCAGAGTACATrGrG+G	Picelli et al., 2013	N/A
ISPCR (HPLC purification): AAGCAGTGGTATCAACGCAGAGT	Picelli et al., 2013	N/A
P5_index (HPLC purification): AATGATACGGCGACCACCGAGATCTACACGCCTGTC CGCGGAAGCAGTGGTATCAACGCAGAGT^∗^T^∗^G	this paper; adapted from Zheng et al., 2018	N/A
P5_SEQ (PAGE purification): GCCTGTCCGCGGAAGCAGTGG TATCAACGCAGAGTTGC^∗^T	this paper; adapted from Zheng et al., 2018	N/A
CS1-seq sequencing primer (HPLC purification): A+CA+CTG+ACGACATGGTTCTACA	N/A	N/A
CS2-seq sequencing primer (HPLC purification): T+AC+GGT+AGCAGAGACTTGGTCT	N/A	N/A
CS1rc-seq sequencing primer (HPLC purification): T+GT+AG+AACCATGTCGTCAGTGT	N/A	N/A
CS2rc-seq sequencing primer (HPLC purification): A+GAC+CA+AGTCTCTGCTACCGTA	N/A	N/A
See Table S2 for pre-amplification, barcoding PCR1 target-specific primer sequences and barcoded oligodT-ISPCR primers	this paper and adaptor from Zheng et al., 2018	N/A

**Software and Algorithms**		

bcl2fastq (version 2.20)	Illumina	RRID:SCR_015058
STAR (version 2.4.2a)	Dobin et al., 2013	https://github.com/alexdobin/STAR RRID: SCR_015899
TrimGalore (version 0.4.1)	Felix Krueger, The Babraham Institute	https://www.bioinformatics.babraham.ac.uk/projects/trim_galore/
FeatureCounts (version 1.4.5-p1)	Liao et al., 2014	http://subread.sourceforge.net/ RRID: SCR_012919
Samtools (version 1.1)	Li et al., 2009	http://samtools.sourceforge.net/ RRID:SCR_002105
R (version 3.4.3)	CRAN	RRID:SCR_001905
Flowjo	Tree Star	RRID:SCR_008520
Gene set enrichment analysis (GSEA)	Broad Institute	RRID:SCR_003199
MSigDB	Broad Institute	RRID:SCR_003199
Graphpad Prism (version 7)	Graphpad	RRID:SCR_002798

**Other**		

Full-length TARGET-seq, 3′TARGETseq detailed protocols and primer design and validation technical note	This Paper	Methods S1

### Contact for Reagent and Resource Sharing

Further information and requests for resources and reagents should be directed to and will be fulfilled by the Lead Contact, Adam Mead (adam.mead@imm.ox.ac.uk).

### Method Details

#### Cell Lines

K562, MOLT4 and JURKAT cells were obtained from the American Type Culture Collection (ATCC). NALM6 cells were obtained from the German Collection of Microorganisms and Cell Cultures (DSMZ). SET2 cells were kindly provided by Dr. Jacqueline Boultwood and Dr. Andrea Pellagatti (Radcliffe Department of Medicine, University of Oxford). All cell lines were maintained in culture in RPMI-1640 supplemented with 10% Fetal Calf Serum (FCS) and antibiotics. Cell lines were authenticated by targeted sequencing of known mutations.

#### Banking and Processing of Human Samples

Patients and normal donors provided written informed consent in accordance with the Declaration of Helsinki for sample collection and use in research under the INForMeD Study (REC:199833, University of Oxford). Cryopreserved peripheral blood and bone marrow mononuclear cells (MNCs) were thawed and processed for flow cytometry analysis as previously described (Woll et al., 2014). Briefly, cryopreserved cells were thawed and 1 mL of FCS was immediately added to each sample. Samples were further diluted with 8mL IMDM (Iscove’s Modified Dulbecco’s Medium) supplemented with 20% FCS and 10% DNase I (Merck). Samples were spun down for 10 min at 350 g, washed and spun down again for 10 min at 350 g. A summary of patients and normal donors’ samples used for analysis can be found in Table 1 and Table S3.

#### Bulk Sequencing of Mononuclear Cells

Bulk genomic DNA from patient samples’ mononuclear cells was isolated using DNeasy Blood & Tissue Kit (QIAGEN) as per manufacturer’s instructions. Targeted sequencing was performed using a TruSeq Custom Amplicon panel (Illumina) consisting of 341 amplicons (∼56 kb) designed around exons of 32 genes frequently mutated in myeloid malignancies (Hamblin et al., 2014). Library preparation was performed as per manufacturer’s instructions using 50-250 ng genomic DNA.

Targets were chosen based on the genes/exons most frequently mutated and/or likely to alter clinical practice (diagnostic, prognostic, predictive or monitoring capacity) across a range of myeloid malignancies (e.g., MDS/AML/ MPN), and can be found in the table below:

**Table:** 

Gene	Exons Covered	Gene	Exons Covered
*ASXL1*	12	*KRAS*	2, 3
*ATRX*	8, 9, 10, 17-31	*MPL*	10
*CBL*	8-9	*NPM1*	11
*CBLB*	9-10	*NRAS*	2,3
*CBLC*	9-10	*PDGFRA*	12, 14, 18
*CEBPA*	1	*PHF6*	2-10
*CSF3R*	14-17	*PTEN*	5-7
*DNMT3A*	23	*RUNX1*	3-8
*ETV6*	1-8	*SETBP1*	4
*EZH2*	2-20	*SF3B1*	14,15
*FLT3*	14, 15, 20	*SRSF2*	1
*HRAS*	2,3	*TET2*	3-11
*IDH1*	4	*TP53*	4-9
*IDH2*	4	*U2AF1*	2, 6
*JAK2*	12,14	*WT1*	7, 9
*KIT*	2, 8-11, 13, 17	*ZRSR2*	1-11

Alignment and variant calling were performed in Basespace (utilizing BWA and GATK/Somatic variant caller; Illumina, or SVC) while filtering and annotation were performed using Variant Studio (Illumina).

Every variant was individually assessed against COSMIC, dbSNP, gnomAD and published literature for frequency in the germline and acquired state and whether any data (*in* vitro or *in vivo*) suggests its likely pathogenicity. Variants with a population frequency of > 1% were considered polymorphisms. Variants with a population frequency of < 1% but with ethnicity bias and a variant allele frequency close to 50% were also considered polymorphisms.

Any variant passing these criteria and a variant allele frequency cut-off of 5% of the reads (point mutations) or 2% of reads (insertions/deletions longer than 5 bp) was reported as mutated in Table S3 and analyzed for each patient.

#### Fluorescent Activated Cell Sorting (FACS) Staining and Single-Cell Isolation

Single cell FACS-sorting was performed as previously described (Giustacchini et al., 2017), using BD Aria III or BD Fusion I instruments (Becton Dickinson) for 96-well plate experiments and SH800S (SONY) for 384-well plate experiments. Full details are provided in Supplemental Experimental Procedures. Experiments involving isolation of human hematopoietic stem and progenitor cells (HSPCs) included single color stained controls (CompBeads, BD Biosciences) and Fluorescence Minus One controls (FMOs). Lineage-CD34+ cells were sorted and indexed for CD38, CD90, CD45RA and CD123 markers, which allowed us to record the fluorescence levels of each marker for each single cell. For samples processed using full-length TARGET-seq in 96 well-plates (Table S3), HSPCs were stained with the following the antibody cocktail: Lineage-FITC, CD34-APC-e780, CD38-PE-TxRed, CD90-BV421, CD45RA-PE and CD123-PECy7. For samples processed using 3′-TARGET-seq in 384-well plates (Table S3), HSPCs were stained with the following antibody cocktail: Lineage-PE/Cy5, CD34-PerCp/Cy5.5, CD38-PE-TxRed, CD90-PE, CD45RA-FITC, CD123-PECy7. The full list of antibodies used for HSPCs immunophenotyping and isolation can be found in Key Resources; 7- aminoactinomycin D (7-AAD) was used for dead cell exclusion. Briefly, single cells directly sorted into 96-well PCR plates containing 4.1-4.2 μL of lysis buffer or into 384-well plates containing 2.07 μL of lysis buffer. K562 cells were sorted into the lysis buffer described in Table S1A. JURKAT, MOLT4, NALM6, SET2 and HSPCs (processed using full length TARGET-seq) were sorted into lysis buffers described in Table S1B. HSPCs processed using 3′-TARGET-seq were sorted into the lysis buffer described in Table S1C, using the barcoded oligodT-ISPCR primers listed in Table S2C (adapted from (Zheng et al., 2018)). Flow cytometry profiles of the HSPC compartment (Figure S4) were analyzed using FlowJo software (version 10.1).

#### cDNA Synthesis (RT-PCR)

For K562 cells, RT and PCR steps were performed as described in Table S1A, using 18 cycles of PCR amplification. For JURKAT, MOLT4, NALM6, SET2 cells and HSPCs (full length TARGET-seq), RT and PCR steps were performed as described in Table S1B, using 20 cycles of PCR amplification for cell lines and 22 cycles of amplification for HSPCs. For HSPCs processed using 3′-TARGET-seq, RT and PCR steps were performed as described in Table S1C, using 24 cycles of PCR amplification. The sequences of the primers used in the RT and PCR steps, for whole transcriptome and targeted retrotranscription and cDNA amplification, can be found in Table S2A and Key Resources Table. Primers were designed to amplify amplicons 250-700 bp long and specificity was checked against RefSeq and human genome assembly databases using PrimerBlast. mRNA and cDNA primers were designed to amplify coding regions whereas gDNA primers were designed to bind at least to one intronic region. More information regarding primer design and validation can be found in the Supplemental Experimental Procedures “Technical Note: Primer Design and Validation.” After PCR, an aliquot of the cDNA-amplicon mix was used for whole transcriptome library preparation and another aliquot, for single-cell genotyping library preparation. For full length TARGET-seq, 15 μL from a total of 25 μL of cDNA-amplicon mix were diluted with 11 μL of water, purified using 16 μL of Ampure XP Beads (0.6:1 beads to cDNA ratio; Beckman Coulter), and resuspended in a final volume of 8 μL of EB buffer (QIAGEN). For high throughput 3′-TARGET-seq, 1 μL from each quadrant of a 384-well plate was pooled to generate a cDNA pool of barcoded libraries; each cDNA pool was purified twice using Ampure XP beads (0.6:1 beads to cDNA ratio). The quality of cDNA traces was checked using a High Sensitivity DNA Kit in a Bioanalyzer instrument (Agilent Technologies). The remaining of the cDNA-amplicon mix was used for subsequent single-cell genotyping or stored at −20 C.

#### Targeted NGS Single-Cell Genotyping

After RT-PCR, 1.5 μL aliquot from each single cell derived cDNA+amplicon mix was used as input to generate a targeted and Illumina-compatible library for single cell genotyping. The preparation of single cell genotyping libraries involves 2 PCR steps (See Supplemental Experimental Procedures). In the first PCR step, target specific primers (Table S2B) attached to universal CS1 / CS2 adaptors (Figure 1, Forward adaptor, CS1: ACACTGACGACATGGTTCTACA; Reverse adaptor, CS2: TACGGTAGCAGAGACTTGGTCT) are used to amplify the target regions of interest. Target-specific primers were designed to specifically amplify cDNA or gDNA, amplifying annotated coding regions in the case of cDNA amplicons and at least one intronic region in the case of genomic DNA amplicons. In the second PCR step (See Detailed Protocol), Illumina compatible adaptors (PE1/PE2) containing 10 bp single-direction indexes (Access Array Barcode Library for Illumina® Sequencers-384, Single Direction, Fluidigm) are attached to pre-amplified amplicons from the first PCR through CS1/CS2 regions, to generate single-cell barcoded libraries. Amplicons were pooled using a Mosquito HTS liquid handling platform (TTP Labtech) and pooled amplicons were purified with Ampure XP beads (0.8:1 ratio beads to product; Beckman Coulter). Purified pools were quantified using Quant-iT Picogreen (Thermo Fisher Scientific) and each pool was diluted to a final concentration of 4 nM. Pools were further diluted to 10 pM in HT1 buffer prior sequencing.

Up to 384 single cells were sequenced on a MiSeq (Illumina) instrument, with the following sequencing configuration: 151 bp R1, 10 bp index read, 151 bp R2. We used custom sequencing primers for Read1 and Read 2 (500 nM CS1-seq and 500 nM CS2-seq; See Key Resources) and Index Read (500 nM CS1rc-seq and 500 nM CS2rc-seq; See Key Resources) diluted in 700 μL of HT1 buffer. Reads were aligned to GRCh37/hg19 using STAR with default settings (version 2.4.2a) and cDNA/gDNA amplicons were separated into different bam files using a custom pipeline, extracting reads matching the different primer sequences used for targeted PCR barcoding. This allowed us to obtain independent mutational information from cDNA and gDNA. Variant calling was performed using mpileup (samtools version 1.1, options–minBQ 30,–count-orphans,–ignore overlaps) and results were summarized with a custom pipeline (https://github.com/albarmeira/TARGET-seq; Figure S2A). Thresholds for the detection of each amplicon were set based on non-template controls and thresholds for mutation calling were based on WT controls and customized for each amplicon (1.5%–4% of the reads, representative examples can be found in Figure S2B). Both non-template and WT controls were routinely processed in parallel to test samples. Importantly, none of the tested mutations were detected in any control cells (n = 874) or blanks (n = 114) in any of the experiments using the mutational pipeline and cut-offs described, implying that the false positive rate of variant calling is effectively zero. For experiments involving isolation of HSPCs, QC genotyping was performed as follows: single cells where one of the targeted amplified genes tested failed to be detected by either gDNA or mRNA were excluded from analysis. Cells for which cDNA/gDNA mutation analysis showed discrepant readouts were considered heterozygous if one of the molecules (cDNA or gDNA) gave a heterozygous readout. When one of the molecules gave a homozygous readout and the other gave a WT readout, cells were also considered heterozygous, although this was a rare event occurring in 0.18% of the amplicons. We considered a cell homozygous when only the mutant allele was detected at the genomic DNA level and we considered a cell WT when only the WT allele was detected at the genomic DNA level. We excluded cells in which only the WT or mutant allele were detected at the mRNA level, but the same gene was not detected at the gDNA level, a rare event occurring in 0.57% of amplicons. Specifically for *JAK2* mutation, where we carried out extensive analysis of the data for zygosity, we included an additional “not determined” category for cells with mRNA and gDNA *JAK2* amplicons in which allele frequency was 0.03 < AF < 0.1 for gDNA (full-length TARGET-seq dataset), 0.04 < AF < 0.1 for gDNA (3′-TARGET-seq dataset) and 0.03 < AF < 0.1 for mRNA (3′-TARGET-seq dataset). Not determined amplicons were excluded from analysis: 36 of 3900 amplicons detected for gJAK2 and 51 out of 1295 amplicons detected for mJAK2. We required a minimum coverage of 30 reads per amplicon to obtain mutational readouts; the mean coverage per amplicon is 2641 reads.

#### Nextera XT Library Preparation for Full-Length Whole-Transcriptome Sequencing

Bead-purified cDNA libraries were used for tagmentation with Nextera XT DNA Kit (Illumina) using one fourth of the original volume as previously described (Giustacchini et al., 2017). 4nM libraries were diluted to 1.8 pM in HT1 buffer and sequenced on a NextSeq instrument with 75 bp single-end reads using a NextSeq 500/550 High Output v2 kit (Illumina). HSPCs were sequenced to a mean sequencing depth of 2.4 M reads.

#### Nextera XT Library Preparation for 3′-Biased Whole-Transcriptome Sequencing

Bead-purified and pooled cDNA libraries were used for tagmentation-based library preparation with Nextera XT DNA Kit (Illumina) using a custom PCR amplification strategy. Briefly, 1 ng of each barcoded cDNA pool was tagmented as per manufacturer’s instructions. Subsequently, reaction was stopped and PCR was performed as per manufacturer’s instructions, with the exception of P5 adaptor, for which 200 nM of a custom P5 adaptor was used (P5_index; See Key Resources). Each indexed pool was bead purified twice with Ampure XP beads (0.7:1 beads to cDNA ratio). 4nM libraries were diluted to 3 pM in a total volume of 1.3 mL of HT1 buffer and were sequenced on a NextSeq instrument, using a NextSeq 500/550 High Output v2 kit (Illumina) with a custom sequencing primer for read1 (P5_SEQ, 900 nM in a total volume of 3 mL of HT1 buffer; See Key Resources) and the following sequencing configuration: 20 bp R1; 8 bp index read; 64 bp R2. HSPCs were sequenced to a mean sequencing depth of 152,552 reads.

#### Single Cell Full-Length RNA-Sequencing Data Pre-Processing

RNA-sequencing reads were trimmed for Nextera adaptors with TrimGalore (version 0.4.1) and aligned to the human genome (hg19) using STAR with default settings (version 2.4.2a). RefSeq gene model was used as the reference for gene expression quantification. Counts for each RefSeq gene were obtained with FeatureCounts (version 1.4.5-p1; options–primary) and were normalized to reads per kilobase per million mapped reads (RPKM). Genes with RPKM values less than 1 were considered non-detected (Giustacchini et al., 2017) and expression values for these genes were converted to zero. We further normalized RPKM expression values into the log2 scale. QC filtering was performed using the following parameters: percentage of reads mapping in exons > 50%, percentage of mapped reads > 50% and number of detected genes per cell (RPKM > = 1) > 6000 for JURKAT and SET2 cells, > 5000 for K562 cells and > 1500 for primary HSPCs. For cell lines, we excluded 8 cells after applying these QC filters (5.3%) and for HSPCs, 33 cells (6.1%).

#### Single Cell 3′-Biased RNA-Sequencing Data Pre-Processing

FASTQ files were generated using bcl2fastq (version 2.20) with default parameters and the following read configuration: Y12N^∗^, I8, Y64N^∗^, in which read1 corresponds to an 8bp cell-specific barcode, index read corresponds to i7 index from each cDNA pool and read2 corresponds to cDNA sequence. Demultiplexed FASTQ files were trimmed for polyA tails using TrimGalore (version 0.4.1); files from different lanes were merged together using samtools (version 1.1) and aligned to the human genome using STAR (version 2.4.2a). RefSeq gene model was used as the reference for gene expression quantification. Counts for each RefSeq gene were obtained with FeatureCounts (version 1.4.5-p1; options–primary). Counts were normalized as follows: counts for each single cell were divided by the total library size for that cell and multiplied by the mean library size of all cells processed (68,412). Genes with normalized count values less than 1 were considered non-detected and expression values for these genes were converted to zero. We further normalized counts into the log2 scale. QC filtering was performed using the following parameters: library size > 2000 reads; percentage of reads mapping to the mitochondrial chromosome < 10%; percentage of ERCC < 50% and number of detected genes per cell (normalized counts > = 1) > 500. We retained 2851 cells after applying these QC filters (81.6%).

#### Whole-Transcriptome Variant Calling from Single Cells

Bam files from 48 single K562 cells (Figure S1F) or 38 single HSPCs (Figure S1H) were merged using samtools to computationally create a single cell ensemble. LoFreq software (Wilm et al., 2012) was used for variant calling in the single cell ensemble. Heterozygous regions across the transcriptome (AF > 0.05 of the minor allele, Allele Frequency) were used for variant calling in each individual cell, requiring a minimum coverage of 10 reads and minimum base quality of 30. A SNV was considered heterozygous if 0.05 < AF < 0.95 and homozygous if AF < 0.05 or AF < 0.95.

#### Mutational Analysis from RNA-Sequencing Reads

Variant calling from raw RNA-sequencing reads was performed using mpileup (samtools version 1.1, options–minBQ 30,–count-orphans,–ignore overlaps) and results were summarized with a custom script (https://github.com/albarmeira/TARGET-seq). Thresholds for the detection of amplicons were set at 30 reads per position (Figure S2C), in line with variant calling guidelines (Sims et al., 2014).

#### Dropout Frequency and Library Bias Calculation

The frequency of dropout for a given gene was calculated as the percentage of cells from a specific condition (SMART-seq2 or SMART-seq+) in which the gene is not detected (RPKM < 1), as compared to the average expression of that gene in K562 bulk samples (6 replicates of 100 cells each; 3 replicates per chemistry). Library bias was calculated as the ratio between the mean RPKM of the top 10% expressed genes in the library and the mean RPKM of all genes.

#### Transcript Coverage

Normalized transcript coverage was calculated using “geneBody_coverage.py” script from RSeQC package (Wang et al., 2012), using a list of 4040 housekeeping genes obtained from http://rseqc.sourceforge.net/.

#### Differential Expression Analysis

Differentially expressed genes were identified using a combination of non-parametric Wilcoxon test, to compare the expression values for each group, and Fisher’s exact test, to compare the frequency of expression for each group, as previously described (Giustacchini et al., 2017). We used log2(RPKM) and log2(normalized counts) matrices, including genes expressed in at least two cells (when analyzing less than 200 cells; Table S4) or in at least five cells (when analyzing over 200 cells; Tables S5, and S6). P values were combined using Fisher’s method and adjusted p values were derived using Benjamini & Hochberg procedure. Significant genes were selected on the basis of adjusted *P* value < 0.1 and absolute log2(fold change)>0.5. Differentially expressed genes in between several distinct genetic subclones (Figure 6, and Table S6) were identified using the “genefilter” package in R with analysis of variance (p value < 0.05). Beeswarm plots from selected genes were generated using “beeswarm” package in R and boxplots from selected genes were generated using “ggplot2” package in R.

#### Identification of Highly Variable Genes

We identified variable genes above technical noise by fitting a lowess model of the log2(mean expression level) and coefficient of variation for each gene. We selected genes with a coefficient of variation above the fitted model and log2(mean expression) > = 0.

#### Single Cell Clustering and Dimensionality Reduction

T-distributed stochastic neighbor embedding (tSNE) was performed using ‘Rtsne’ package, the implementation of the method in R, with “perplexity” = 15 for Figures 4A and 4B “perplexity=20” for Figures 2B and 4C. For the analysis of 3′-TARGET-seq, similarly to other high-throughput 3′-biased techniques, we first computed a PCA reduction using 50 dimensions, and then used the top thirty (Figures 5A, 5D, 5E, and 5G), top twenty (Figure 5H) or top five dimensions (Figures 6A, 6C, 6E, and S7A–F) with higher variance to generate the tSNE plots in Figures 5, 6, and S7, using “perplexity=20” for Figure 5H, “perplexity=25” for Figures 6A, 6C, 6E, and S7A–F, and “perplexity=30” for Figures 5A, 5D, 5E, and 5G. The number of genes used for each analysis is specified in the legend for each figure. Zero Inflated Factor Analysis (ZIFA) (Pierson and Yau, 2015) was used to assess transcriptional heterogeneity associated with the subclonal composition of patients IF0137, IF0138 and IF0101 (Figures S7G–I), performed using highly variable genes with default parameters. SC3 software (Kiselev et al., 2017) was used to analyze the subclonal composition of patients IF0137, IF0138 and IF0101, using default parameters and k = 4 for patient IF0137 (as there are four genetically-distinct subclones; Figure S7J) or k = 3 for patients IF0138 and IF0101 (as there are three genetically-distinct subclones; Figures S7K and S7L) with default parameters. K-Nearest Neighbors clustering integrated in the PAGODA2 package (https://github.com/hms-dbmi/pagoda2) was used to analyze the subclonal composition of patients IF0137, IF0138 and IF0101 (Figures S7M–O). We calculated a PCA reduction of the batch-corrected gene expression matrix using 50 principal components and 3000 overdispersed genes, computed nearest neighbors using “cosine” distance (k = 15) and identified clusters using “multilevel community” method. We then plotted the tSNE graphs presented in Figures S7M–O with “perplexity=25.” We observed that transcriptional heterogeneity between genetic subclones within individual patients was better captured with higher-dimensionality representations, and we therefore represent three tSNE dimensions in Figures 6 and S7.

#### Cell to Cell Correlation Measurements

Pearson’s correlation between single cells for each genetic subgroup was calculated using the log2(normalized counts), including genes expressed in at least five cells (Figure 5C).

#### Batch Correction

Batch correction was performed using “limma” package in R (Figures 4, 5, 6, and S7). Gene expression matrix was batch and donor corrected in Figures 4C, 5A, and 5H, while preserving genotypes. Gene expression matrix was batch corrected in Figures 5D, 5E, and 5G, while preserving donor effect. Gene expression matrix was batch corrected in Figures S7A and S7D and plate corrected in Figures S7C and S7F. We used batchNorm function from PAGODA2 package (method = ”glm”) to perform batch correction in Figures S7M and S7O.

#### Cell Cycle Phase Assignment and Correction

An S-phase and G2M-phase cell cycle score was calculated as the mean expression value of a set of S-phase and G2M-phase genes (Tirosh et al., 2016a) for each cell. S-phase and G2M-phase scores were used to fit a linear model on the normalized and logged gene expression matrices using “limma” package in R, in order to remove the effect of cell cycle. Cell-cycle corrected matrices were used as an input for the analysis presented in Figures 5A, 5H, and S7D–F.

#### Random Forest Analysis

Random forest analysis was performed using ‘randomForest’ package in R (ntree = 2000), trained on the genotypes of single cells. Only genotypes with at least five cells were included in this analysis. Expression matrix was batch and donor-corrected, and genotypes were preserved. The top 2000 genes identified by the random forest analysis (MeanDecreaseGini > 0.041 in Figure 4C; MeanDecreaseGini > 0.045 in Figure 5H) were used for the tSNE representation in Figures 4C and 5H (perplexity = 20). Clustering of cells was stable when selecting from 500 to 5000 top genes from the random forest analysis.

#### GeneSet Enrichment Analysis

GSEA was performed using GSEA software (http://software.broadinstitute.org/gsea) with default parameters and 1000 permutations on the phenotype. Gene sets used for the analysis were downloaded from MSigDB or relevant studies (Table S4H). Single Sample GSEA (ssGSEA) was performed using ssGSEA Projection Module (https://genepattern.broadinstitute.org) with default settings and combine mode ‘combine.off’. A projection of ssGSEA results is shown in Figure 4B.

### Quantification and Statistical Analysis

Unpaired Student t test with Welch’s correction was used for the comparisons in Figures S1A, S1B, S1D, S1E, 2C, and S3A. Kolmogorov-Smirnov test was used for the comparison of Pearson’s correlations distributions in Figure 5C.

#### Computational Reconstruction of Clonal Hierarchies

Phylogenetic tree reconstruction for patients with more than one driver mutation was performed using SCITE (Jahn et al., 2016) with default parameters and “-r 1 -l 900000 -fd 0.001 -ad 0.01 0.01 -cc 0.” We accounted for Loss of Heterozygosity in *JAK2* by introducing the mutational status of each *JAK2* allele as separate components of the mutational matrix.

#### Code Availability

R, Perl and Python scripts used for the analysis are available upon request or accessible at https://github.com/albarmeira/TARGET-seq. Genotyping pipeline used for analysis of targeted-sequencing data generated by TARGET-seq (SCpipeline) can be downloaded from https://github.com/albarmeira/TARGET-seq.

### Data and Software Availability

Single cell RNA-sequencing data is available at GEO: GSE105454. Single cell targeted sequencing data is available at the NCBI’s SRA data repository with project number SRA: PRJNA503734 (validation experiments), SRA: PRJNA503736 (full-length TARGETseq patients’ dataset) and SRA: PRJNA503628 (3′-TARGETseq patients’ dataset).

### Additional Resources

Detailed protocols and primer design technical note: a detailed full-length TARGET-seq, 3′-TARGET-seq protocol and a Technical Note describing primer design and validation is provided as Supplemental Experimental Procedures.

## Supplemental Information

Document S1. Figures S1–S7 and Supplemental Experimental Procedures

Table S1. Reagents and Conditions Used for cDNA Synthesis of SMART-Seq2, SMART-Seq+, mRNA Targeting, and TARGET-Seq Libraries

Table S2. Primers Used for the Amplification of mRNA and gDNA Amplicons during RT, PCR, and Barcoding PCR Steps and Barcoded OligodT-ISPCR Primers for 3'-TARGET-Seq

Table S3. Samples from Patients and Normal Donors Included in the Study; Clinical Characteristics and Genetic Subclones Identified in Each Patient

Table S4. Differentially Expressed Genes and GSEA Analysis of JAK2V617F, EZH2, and TET2 Mutant Cells, Wild-Type Cells from Patients, and Cells from Normal Donors; Included Is a Full List of Genesets Used for the Study

Table S5. GSEA Analysis and Differential-Expression Analysis of *JAK2*-Heterozygous, *JAK2*-Homozygous, and Wild-Type Cells from Patient Samples compared to Wild-Type Cells from Normal Donors and Wild-Type Cells from Patient Samples

Table S6. Differentially Expressed Genes between Different Genetic Subclones from Patients IF0137, IF0138, and IF0101

Document S2. Article plus Supplemental Information
