# Supplementary material for: Unravelling Intratumoral Heterogeneity through High-Sensitivity Single-Cell Mutational Analysis and Parallel RNA Sequencing
Source: Mol Cell. 2019 Mar 21;73(6):1292–1305.e8. doi: 10.1016/j.molcel.2019.01.009 (PMC6436961; doi:10.1016/j.molcel.2019.01.009)
Supplement: Document S1. Figures S1–S7 and Supplemental Experimental Procedures [file mmc1.pdf]

Molecular Cell, Volume *73*

Supplemental Information

Unravelling Intratumoral Heterogeneity through High-Sensitivity Single-Cell Mutational Analysis and Parallel RNA Sequencing

Alba Rodriguez-Meira, Gemma Buck, Sally-Ann Clark, Benjamin J. Povinelli, Veronica Alcolea, Eleni Louka, Simon McGowan, Angela Hamblin, Nikolaos Sousos, Nikolaos Barkas, Alice Giustacchini, Bethan Psaila, Sten Eirik W. Jacobsen, Supat Thongjuea, and Adam J. Mead

Figure S1

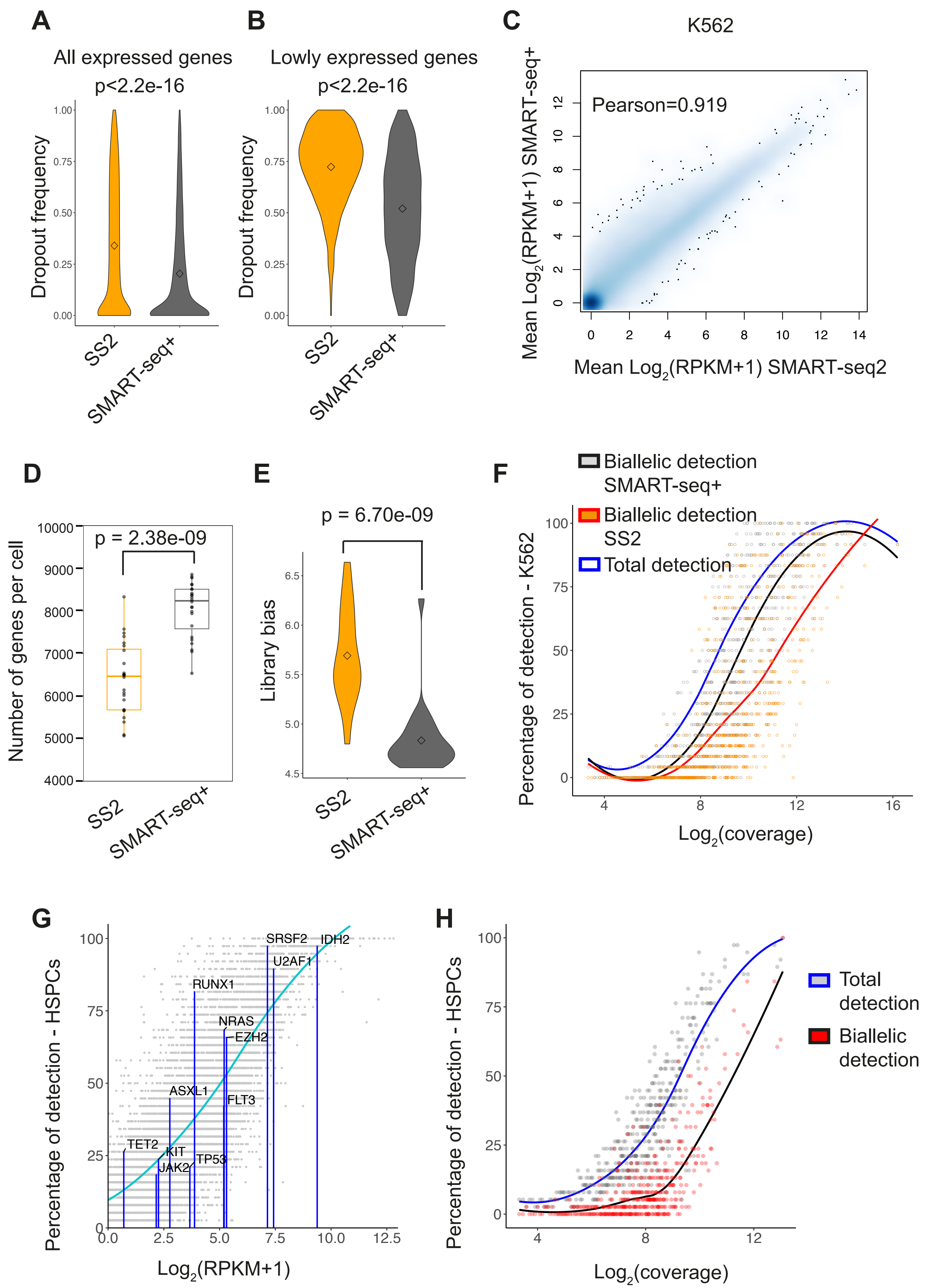
A
All expressed genes
p<2.2e-16
Dropout frequency
SS2
SMART-seq+
B
Lowly expressed genes
p<2.2e-16
Dropout frequency
SS2
SMART-seq+
C
K562
Pearson=0.919
Mean Log_2(RPKM+1) SMART-seq+
Mean Log_2(RPKM+1) SMART-seq2
D
p = 2.38e-09
Number of genes per cell
SS2
SMART-seq+
E
p = 6.70e-09
Library bias
SS2
SMART-seq+
F
Biallelic detection SMART-seq+
Biallelic detection SS2
Total detection
Percentage of detection - K562
Log_2(coverage)
G
SRSF2
IDH2
U2AF1
RUNX1
NRAS
EZH2
ASXL1
FLT3
TET2
KIT
JAK2
TP53
Percentage of detection - HSPCs
Log_2(RPKM+1)
H
Total detection
Biallelic detection
Percentage of detection - HSPCs
Log_2(coverage)

Figure S1, related to Figure 1. Single-cell RNA-sequencing is associated with high levels of allelic dropout. (a) Comparison of dropout frequency between SMART-seq2 (SS2) and SMART-seq+ methods (n=48 single K562 cells; 24 cells per chemistry from three independent experiments) for all genes expressed in K562 bulk samples (RPKM>1; 9096 genes). P-value from two-tailed unpaired Student's t-test is shown on the top of the graph. Points represent the mean for each group. **(b)** Comparison of dropout frequency for lowly expressed genes (2>RPKM>1; 1347 genes) between SMART-seq2 (SS2) and SMART-seq+ methods for the same cells as in (a). P-value from two-tailed unpaired Student's t-test is shown on the top of the graph. Points represent the mean for each group. **(c)** Pearson's correlation between mean log2(RPKM+1) values for SMART-seq2 and SMART-seq+ chemistries (n=48). **(d)** Number of detected genes per cell in K562 cells processed using Smart-seq2 or optimized SMART-seq+ chemistry for the same cells as in (a-c). P-value from two-tailed unpaired Student's t-test is shown on the top of the graph. Boxes represent median and quartiles and points represent the value for each single cell. **(e)** Library bias per chemistry, calculated as the ratio between the mean RPKM values of the top 10% expressed genes and the mean RPKM for all genes expressed in the library, using the same 48 cells as in (a-d). P-value from two-tailed unpaired Student's t-test is shown on the top of the graph. Points represent the mean for each group. **(f)** Percentage of total (dark blue line) or bi-allelic detection in heterozygous SNVs for Smart-seq2 (orange dots and red line) or optimized SMART-seq+ (grey dots and black line) chemistries (n=48 single K562 cells). Lines represent the mean percentage of detection (y-axis) with respect to log2(coverage; x-axis) and points represent individual SNVs. **(g)** Total percentage of detection of selected myeloid genes in Lin-CD34+CD38- hematopoietic stem/progenitor cells (HSPC; n=38; y-axis) with respect to the average level of expression for each gene (log2(RPKM+1); x-axis). Blue bars represent detection of specific gene transcripts that are frequently mutated in myeloid malignancies. The light blue line represents the average percentage of detection for a certain expression value (number of cells that express that gene divided by the total number of cells), and each grey dot represents an individual transcript. **(h)** Total versus bi-allelic percentage of detection of heterozygous SNVs in the same single cells as in (g) with respect to the total number of reads spanning that position (log2(coverage); x-axis). The blue line and grey points represent the total percentage of detection for a certain heterozygous position. The black line and red points indicate the detection of both alleles (at least 5% of reads mapping to either of the alleles).

Figure S2

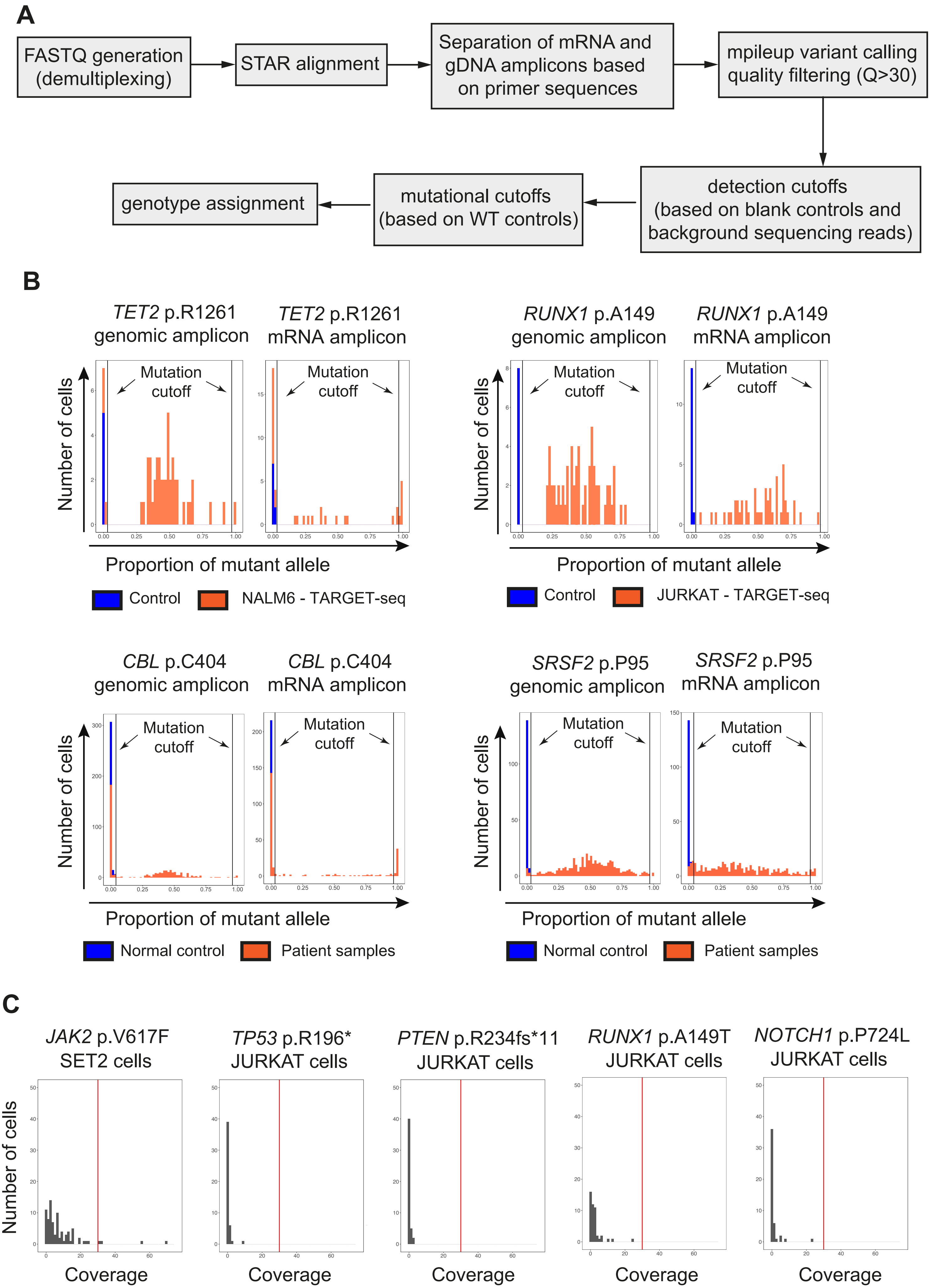
A
FASTQ generation (demultiplexing)
STAR alignment
Separation of mRNA and gDNA amplicons based on primer sequences
mpileup variant calling quality filtering (Q>30)
detection cutoffs (based on blank controls and background sequencing reads)
mutational cutoffs (based on WT controls)
genotype assignment
B
TET2 p.R1261 genomic amplicon
TET2 p.R1261 mRNA amplicon
RUNX1 p.A149 genomic amplicon
RUNX1 p.A149 mRNA amplicon
Mutation cutoff
Number of cells
Proportion of mutant allele
Control
NALM6 - TARGET-seq
JURKAT - TARGET-seq
CBL p.C404 genomic amplicon
CBL p.C404 mRNA amplicon
SRSF2 p.P95 genomic amplicon
SRSF2 p.P95 mRNA amplicon
Normal control
Patient samples
C
JAK2 p.V617F SET2 cells
TP53 p.R196* JURKAT cells
PTEN p.R234fs*11 JURKAT cells
RUNX1 p.A149T JURKAT cells
NOTCH1 p.P724L JURKAT cells
Number of cells
Coverage

Figure S2, related to Figure 1. Targeted pre-amplification and sequencing of mRNA and gDNA amplicons dramatically increases the sensitivity of mutation detection. (a) Schematic representation of the pipeline used for variant calling of targeted next generation sequencing. **(b)** Representative examples of variant allele frequencies and mutational cutoffs for gDNA and cDNA amplicons in *TET2* p.R1261 mutation in NALM6 cell line, *RUNX1* p.A149T mutation in JURKAT cell line, and *CBL* p.C404 and *SRSF2* p.P95 mutations in patient samples and normal donors. Black lines represent mutation cut-offs for each amplicon. **(c)** RNA-sequencing coverage of *JAK2* mutation in SET2 cells and *TP53, NOTCH1, RUNX1* and *PTEN* mutations in JURKAT cells. The y-axis represents the number of cells against their coverage for each mutation in the x-axis. Red line represents a coverage threshold of 30, used as minimum coverage for targeted sequencing experiments.

Figure S3

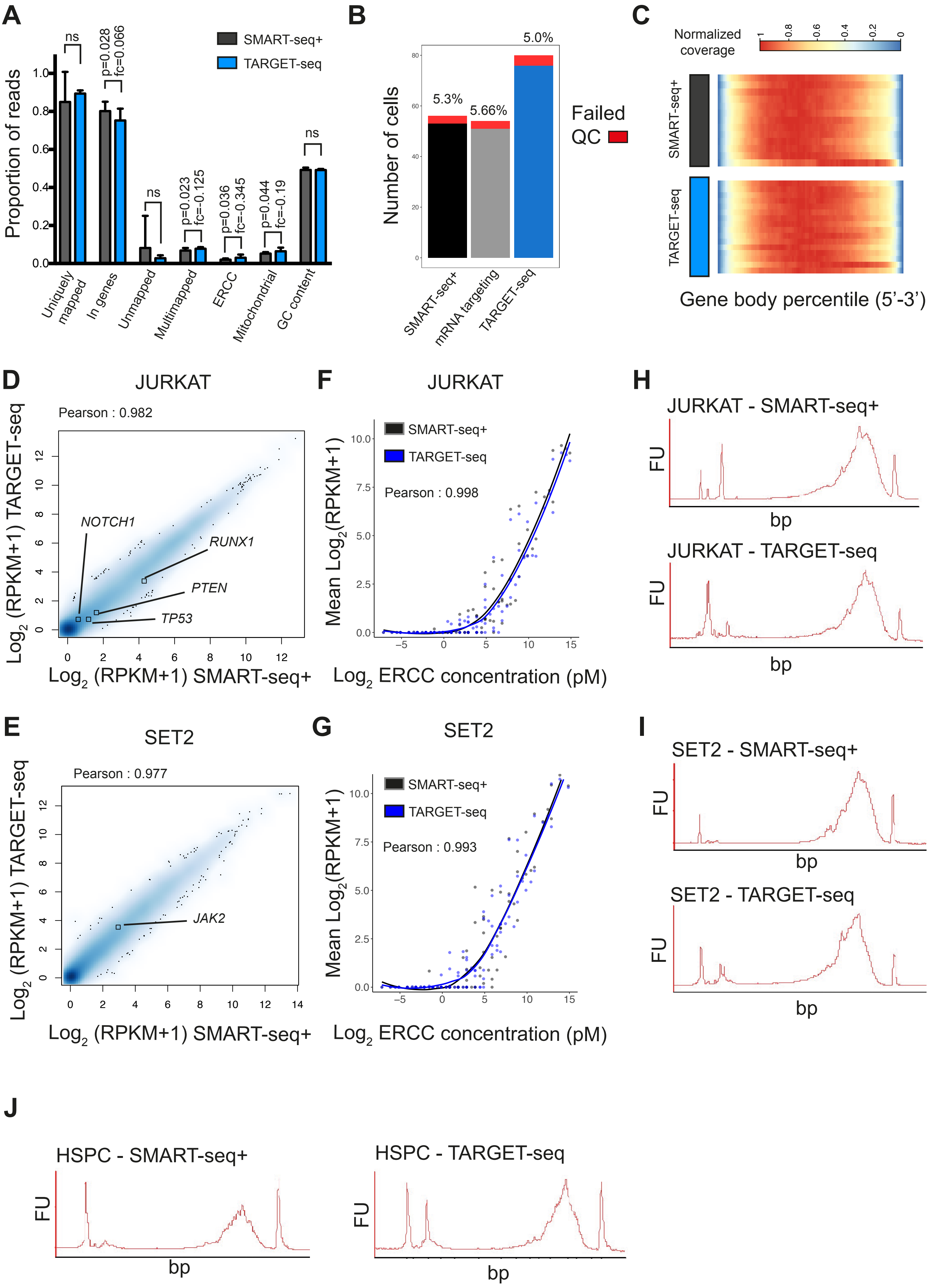
A
SMART-seq+
TARGET-seq
Proportion of reads
ns
p=0.028 fc=0.066
ns
p=0.023 fc=-0.125
p=0.036 fc=-0.345
p=0.044 fc=-0.19
ns
Uniquely mapped
In genes
Unmapped
Multimapped
ERCC
Mitochondrial
GC content
B
Number of cells
5.3%
5.66%
5.0%
Failed QC
SMART-seq+
mRNA targeting
TARGET-seq
C
Normalized coverage
SMART-seq+
TARGET-seq
Gene body percentile (5'-3')
D
JURKAT
Pearson : 0.982
NOTCH1
RUNX1
PTEN
TP53
Log2 (RPKM+1) TARGET-seq
Log2 (RPKM+1) SMART-seq+
F
JURKAT
SMART-seq+
TARGET-seq
Pearson : 0.998
Mean Log2(RPKM+1)
Log2 ERCC concentration (pM)
H
JURKAT - SMART-seq+
JURKAT - TARGET-seq
FU
bp
E
SET2
Pearson : 0.977
JAK2
Log2 (RPKM+1) TARGET-seq
Log2 (RPKM+1) SMART-seq+
G
SET2
SMART-seq+
TARGET-seq
Pearson : 0.993
Mean Log2(RPKM+1)
Log2 ERCC concentration (pM)
I
SET2 - SMART-seq+
SET2 - TARGET-seq
FU
bp
J
HSPC - SMART-seq+
HSPC - TARGET-seq
FU
bp

Figure S3, related to Figure 2. Unbiased whole transcriptome analysis of single cells using TARGET-seq. **(a)** Sequencing statistics of single cell libraries from HSPCs processed using SMART-seq+ or TARGET-seq. The bar graph represents the proportion of reads for each sequencing statistic and condition, and error bars represent standard deviation of the mean. P-values from two-tailed Student's t-test and fold change values for each sequencing statistic are shown on the top of each pair of bars. **(b)** Number of cells passing or failing QC (Quality Control) per method. The percentage of cells failing QC for each method is shown on the top of each bar. **(c)** Normalized transcript coverage from single HSPCs processed using SMART-seq+ or TARGET-seq methods, using 4040 housekeeping genes. **(d,e)** Whole transcriptome Pearson's correlation between SMART-seq+ and TARGET-seq ensembles (mean RPKM values per condition) in JURKAT **(d)** and SET2 cells **(e)**. The expression values for the genes targeted are highlighted in each cell type. **(f,g)** Pearson's correlation between mean ERCC spike-in expression values from SMART-seq+ and TARGET-seq in JURKAT cells **(f)** and SET2 cells **(g)** per each ERCC spike-in concentration. **(h-j)** Bioanalyzer traces of representative cDNA libraries synthetized using SMART-seq+ or TARGET-seq in JURKAT **(h)**, SET2 **(i)** or HSPCs **(j)**.

Figure S4

A HD7643

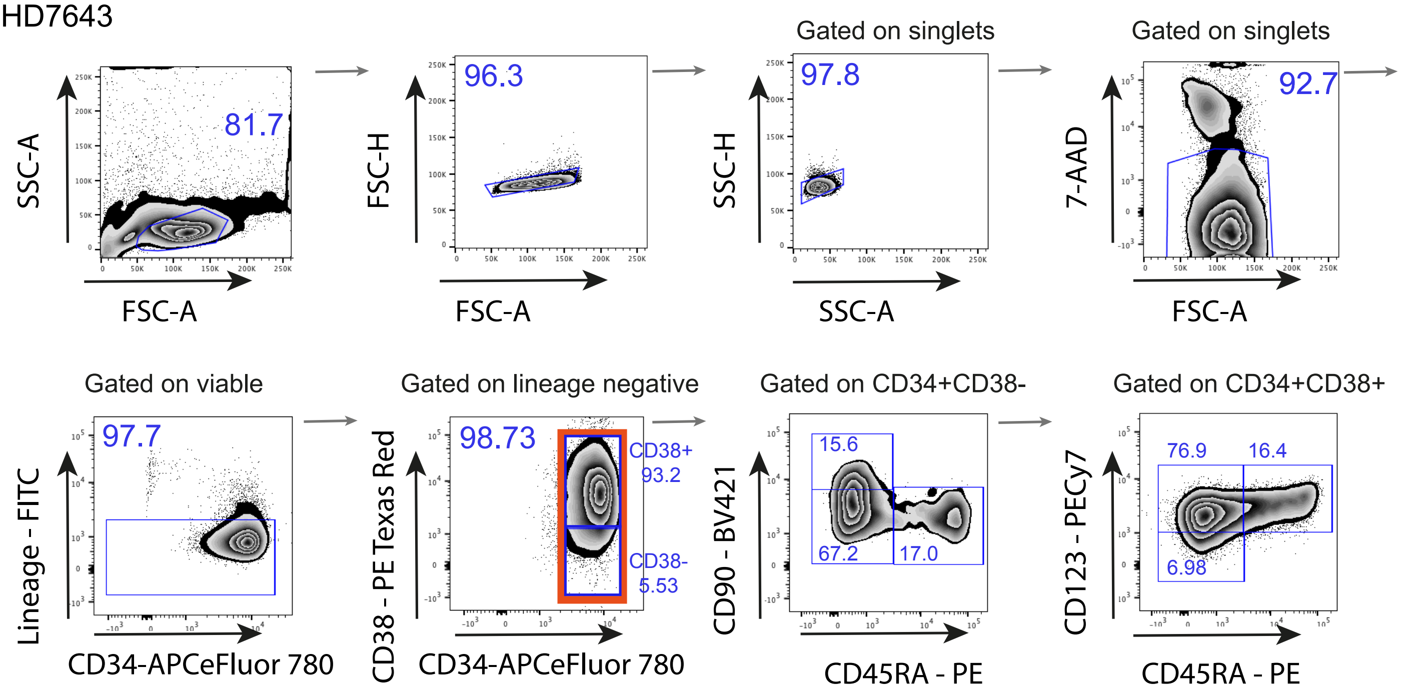

B OX2123

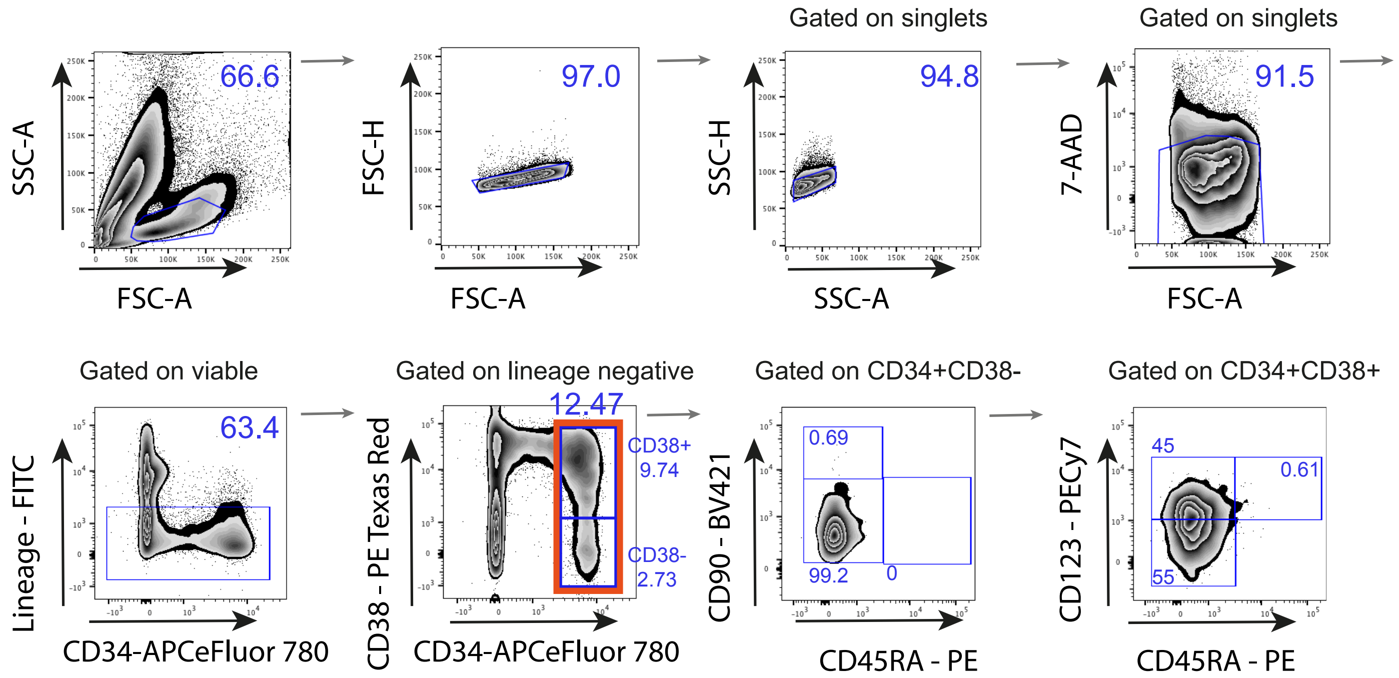

Figure S4, related to Figures 3 and 4. Schematic representation of gating and sorting strategy. (a-b) Schematic representation of gating and sorting strategy for a CD34+ selected healthy donor sample (a; HD7643) or patient sample (b; OX2123). Orange square represents sorting gate. Numbers represent percentage of gated cells. Antibodies used for HSPC isolation are listed in Key Resources Table and STAR Methods.

Figure S5

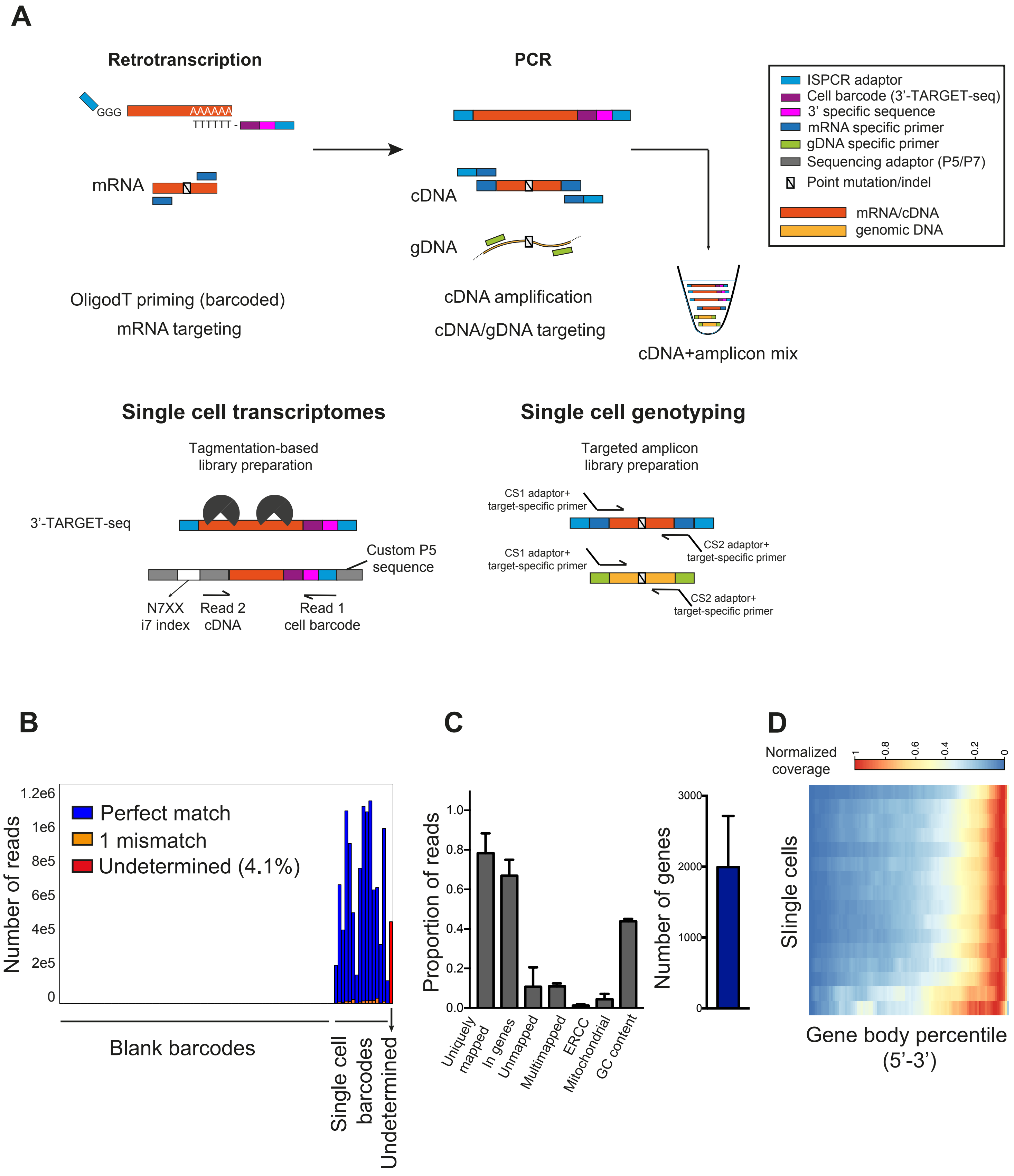
A
Retrotranscription
GGG
AAAAAA
TTTTTT -
mRNA
OligodT priming (barcoded)
mRNA targeting
PCR
cDNA
gDNA
cDNA amplification
cDNA/gDNA targeting
cDNA+amplicon mix
ISPCR adaptor
Cell barcode (3'-TARGET-seq)
3' specific sequence
mRNA specific primer
gDNA specific primer
Sequencing adaptor (P5/P7)
Point mutation/indel
mRNA/cDNA
genomic DNA
Single cell transcriptomes
Tagmentation-based
library preparation
3'-TARGET-seq
Custom P5
sequence
N7XX
i7 index
Read 2
cDNA
Read 1
cell barcode
Single cell genotyping
Targeted amplicon
library preparation
CS1 adaptor+
target-specific primer
CS2 adaptor+
target-specific primer
CS1 adaptor+
target-specific primer
CS2 adaptor+
target-specific primer
B
Number of reads
1.2e6
1e6
8e5
6e5
4e5
2e5
0
Perfect match
1 mismatch
Undetermined (4.1%)
Blank barcodes
Single cell
barcodes
Undetermined
C
Proportion of reads
1.0
0.8
0.6
0.4
0.2
0.0
Uniquely
mapped
In genes
Unmapped
Multimapped
ERCC
Mitochondrial
GC content
Number of genes
3000
2000
1000
0
D
Normalized
coverage
1
0.8
0.6
0.4
0.2
0
Slingle cells
Gene body percentile
(5'-3')

Figure S5, related to Figures 5 and 6. Validation of high throughput 3'-TARGET-seq. (a) Schematic representation of 3'-TARGET-seq method. Briefly, a barcoded oligodT-ISPCR primer was used to prime polyadenylated mRNA molecules from each single cell; a 3'-specific sequence is also added to preferentially enrich for fragments containing the 3'-end of the molecule in tagmentation-based library preparation and 3'-biased sequencing. **(b)** Detection of cellular barcodes using 3'-TARGETseq in 16 HSPCs. Blue bars represent total number of reads mapping to cellular barcodes used for cDNA synthesis of HSPCs (16 barcodes); blank barcodes represent those not used for cDNA synthesis (80 barcodes); red bar represents the total number of reads from cell barcodes that do not match any of the 96 available cell barcodes. **(c)** Sequencing statistics of 3'-TARGET-seq libraries from the same 16 HSPCs as in (b) and number of genes detected per cell. Bars represents the proportion of reads for each sequencing statistic (left panel) or number of genes detected per cell (normalized counts≥1, right panel); error bars represent standard deviation of the mean. "GC" refers to guanine-cytosine content. **(d)** Normalized transcript coverage across 4040 housekeeping genes for 16 single HSPCs processed using 3'-TARGET-seq, showing expected 3' bias.

Figure S6

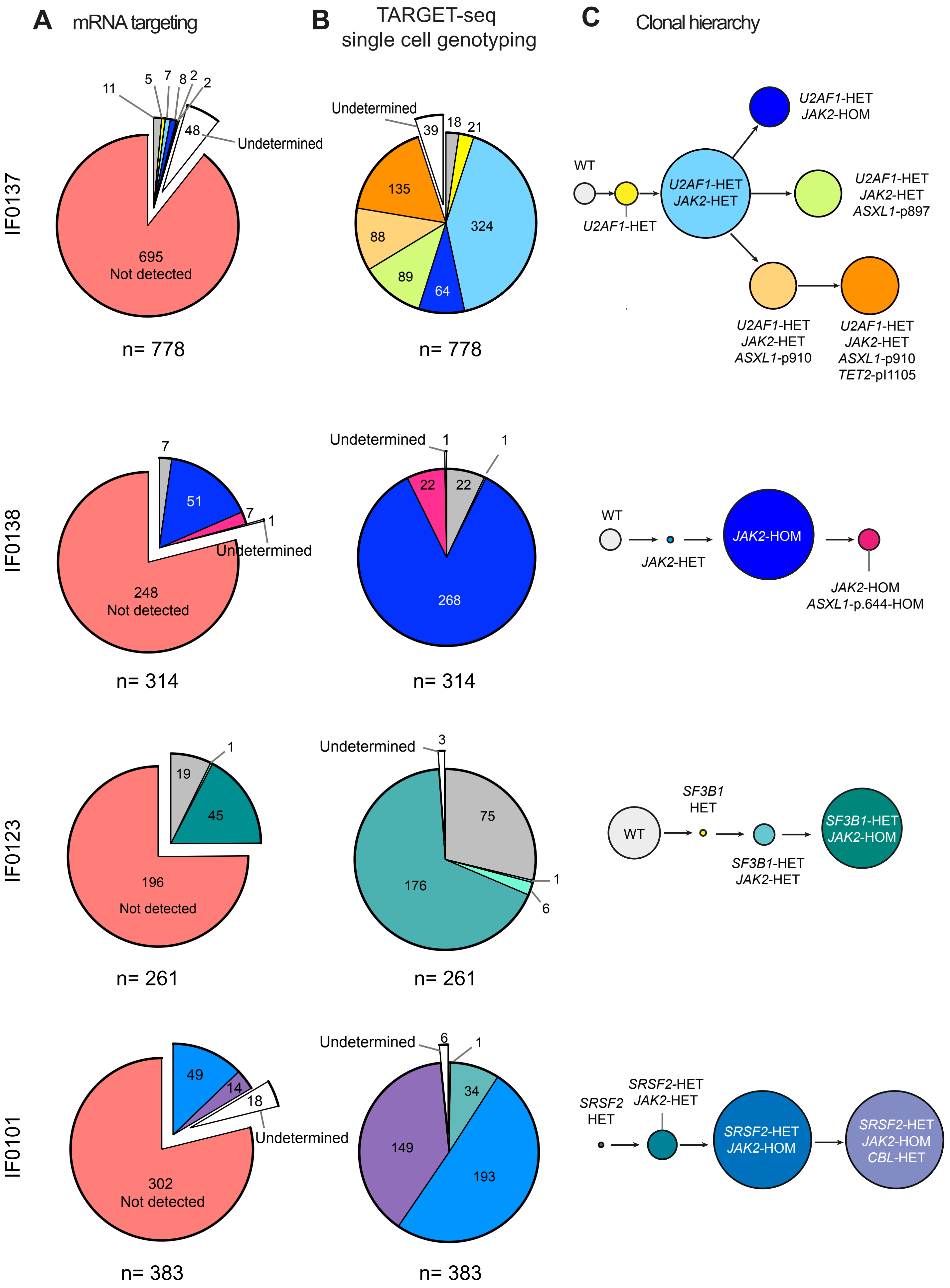

A
mRNA targeting
B
TARGET-seq
single cell genotyping
C
Clonal hierarchy
IF0137
11
5
7
8
2
2
48
Undetermined
695
Not detected
n= 778
Undetermined
39
18
21
135
324
88
89
64
n= 778
WT
U2AF1-HET
U2AF1-HET
JAK2-HET
U2AF1-HET
JAK2-HOM
U2AF1-HET
JAK2-HET
ASXL1-p897
U2AF1-HET
JAK2-HET
ASXL1-p910
U2AF1-HET
JAK2-HET
ASXL1-p910
TET2-pI1105
IF0138
7
51
7
1
Undetermined
248
Not detected
n= 314
Undetermined
1
1
22
22
268
n= 314
WT
JAK2-HET
JAK2-HOM
JAK2-HOM
ASXL1-p.644-HOM
IF0123
19
1
45
196
Not detected
n= 261
Undetermined
3
75
176
1
6
n= 261
WT
SF3B1
HET
SF3B1-HET
JAK2-HET
SF3B1-HET
JAK2-HOM
IF0101
49
14
18
Undetermined
302
Not detected
n= 383
Undetermined
6
1
34
149
193
n= 383
SRSF2
HET
SRSF2-HET
JAK2-HET
SRSF2-HET
JAK2-HOM
SRSF2-HET
JAK2-HOM
CBL-HET

Figure S6, related to Figures 5 and 6. TARGET-seq reveals genetic subclones in the HSPC compartment from myelofibrosis patients that could not be inferred through bulk sequencing or by mRNA targeting alone. (a-b) Subclonal composition of indicated patients' HSPC compartment identified by single cell genotyping using (a) mutational information from targeted mRNA amplicons (mRNA targeting) or (b) TARGET-seq. Total number of cells identified per subclone is shown in each slice of the pie chart, and the total number of cells passing QC genotyping for each patient is shown below each chart. "Undetermined" cells (those not fitting in the clonal hierarchy determined by SCITE) are coloured in white; "ND" (Not Detected; coloured in red) represents cells in which at least one of the amplicons was not detected. Each patient is labelled according to the code provided in Table S3. **(c)** Clonal hierarchies identified by SCITE for each patient. Each subclone in (a-b) is color-coded according to the clonal tree presented in (c). A full list of genetic subclones identified for all patients can be found in Table S3b. The size of circles in the clonal tree represents the relative fraction of detected cells according to (b).

Figure S7

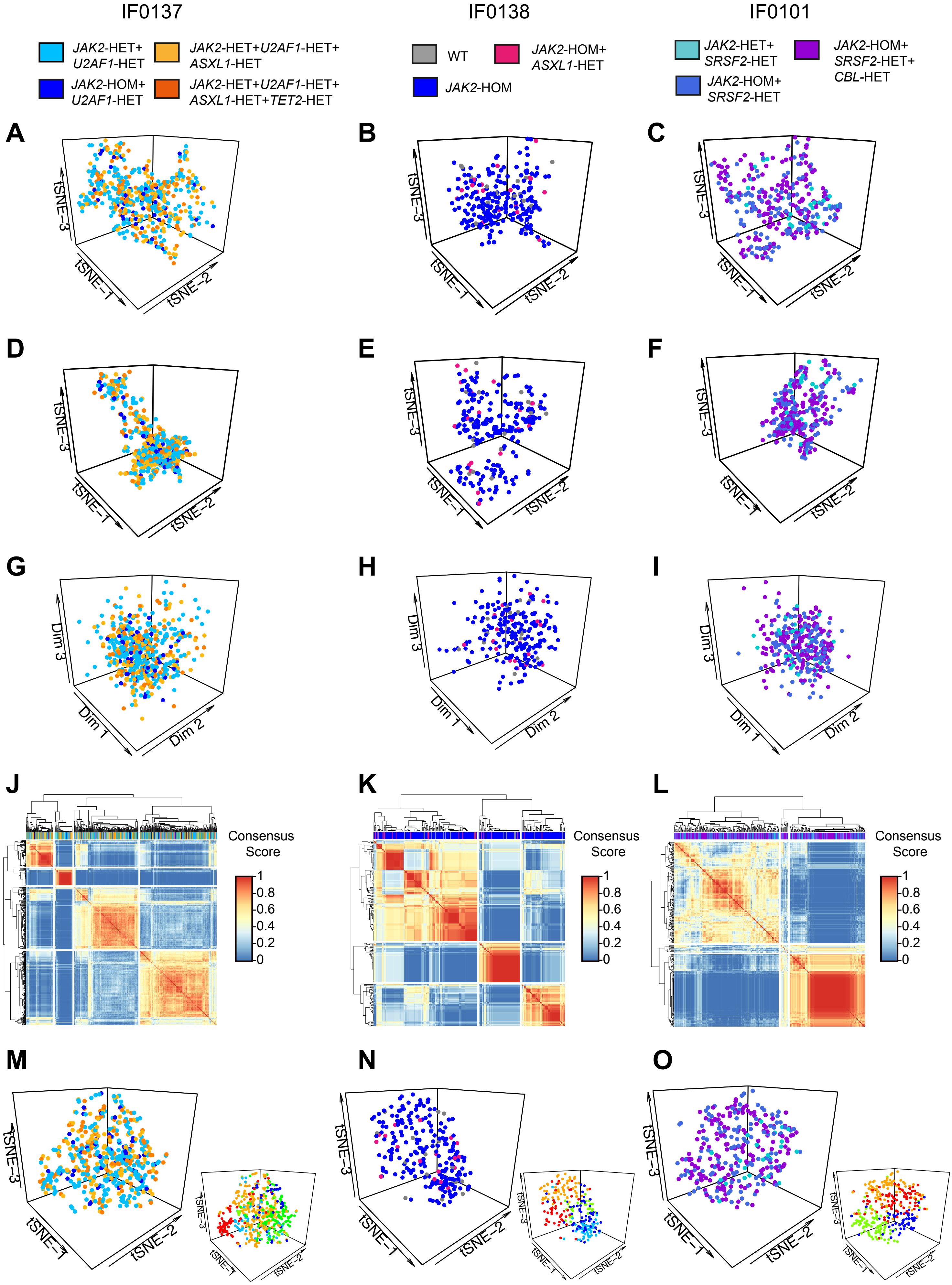
IF0137
IF0138
IF0101
JAK2-HET+ U2AF1-HET
JAK2-HET+U2AF1-HET+ ASXL1-HET
JAK2-HOM+ U2AF1-HET
JAK2-HET+U2AF1-HET+ ASXL1-HET+TET2-HET
WT
JAK2-HOM+ ASXL1-HET
JAK2-HOM
JAK2-HET+ SRSF2-HET
JAK2-HOM+ SRSF2-HET+ CBL-HET
JAK2-HOM+ SRSF2-HET
A
B
C
D
E
F
G
H
I
J
K
L
M
N
O
tSNE-1
tSNE-2
tSNE-3
Dim 1
Dim 2
Dim 3
Consensus Score
1
0.8
0.6
0.4
0.2
0

Figure S7, related to Figure 6. **Computational analysis of scRNA-seq data does not distinguish genetically distinct subclones of HSPCs within individual myelofibrosis patients**. **(a-c)** tSNE representation of 555 cells from patient IF0137 (a), 243 cells from patient IF0138 (b) and 320 cells from patient IF0101 (c) using 3031 (a), 2605 (b) and 3023 (c) highly variable genes. Gene expression matrices were batch corrected and genotypes were preserved. Cells are colored according to each genotype group for each patient. **(d-f)** tSNE representation from the same cells and patients as in (a-c), using highly variable genes and regressing out the effect of the cell cycle. Gene expression matrices were batch corrected and genotypes were preserved. **(g-i)** ZIFA dimensionality reduction from the same single cells and patients as in (a-c) using 3031 (g), 2605 (h) or 3023 (i) highly variable genes. **(j-l)** SC3 K-means clustering clustering from the same single cells and patients as in (a-c) using k=4 for patient IF0137 (j), k=3 for patient IF0138 (k) and k=3 for patient IF0101 (l). Heatmaps are coloured according to the consensus score computed by SC3. **(m-o)** tSNE representation of clusters identified by PAGODA2 from the same single cells and patients as in (a-c), coloured by genotype (left panel) or by clusters identified by PAGODA2 (right panel). We identified 7 clusters in patient IF0137 (m), 5 clusters in patient IF0138 (n) and 4 clusters in patient IF0101 (o). Gene expression matrices were batch corrected using the batchNorm function (method='glm').

SUPPLEMENTAL EXPERIMENTAL PROCEDURES

Detailed step by step TARGET-seq protocols and primer design and validation technical note, related to Figure 1 and Figure S5.

Full-length TARGET-seq protocol in 96 well plates

Materials

- 96-well PCR plates (Thermo Fisher Scientific, AB-0900)
- 384 well PCR plates (FrameStar, 4titude, 4ti-0384/C)
- Corning® 96 Well TC-Treated Microplates (Cat. No. CLS3595-50EA)
- RNase Free Microfuge Tubes (Invitrogen, AM12400)
- V-shaped 96 well plate (AXYGEN, P-96-450V-C, 500 uL 96 well "V" bottom clear; Ref. 391-02-501)
- PCR film (Thermo Fisher Scientific, MicroAMp Clear Adhesive Film, Cat. No. 4306311)
- Aluminium Sealing Film (StarLab, E2796-0792)
- 96-well magnetic stand (Invitrogen, AM10027)
- Protease (Qiagen, Cat#19155)
- Triton X-100 (Sigma-Aldrich, Cat#T8787)
- RNase inhibitor (TAKARA, Cat#2313A)
- dNTPs (Life Technologies, Cat#R0192)
- UltraPure DNAse/RNAse-Free Distilled Water (Life Technologies, Cat#10977035)
- EB Buffer (Qiagen, Cat No./ID: 19086)
- Ethanol
- RNAse-free TE buffer (Invitrogen, Cat#AM9849)
- ERCC aliquot (Ambion, Cat#4456740)
- SMARTScribe enzyme (Clontech - Cat. No. 639537).
- SeqAMP enzyme (Clontech, Cat#638509).
- RT-PCR Grade Water (Life Technologies, Cat#AM9935).
- Ampure XP beads (Beckman Coulter, Cat#A63881)
- Pre-amplification primers: oligodT-ISPCR primer, mRNA target-specific primers, TSO-LNA; gDNA target-specific primers, cDNA target-specific primers and ISPCR primers. Keep in dedicated “pre-amplification” area only. Custom primers from Biomers.net (oligodT-ISPCR, mRNA/gDNA/cDNA target-specific primers, ISPCR primers; HPLC purification) and Qiagen (TSO-LNA; RNAse free HPLC purification).
- High Sensitivity NGS Fragment Analysis Kit (1bp - 6,000 bp; Agilent; Cat# DNF-474) or similar kit for capillary system (High Sensitivity D5000 ScreenTape System; Agilent, Cat# 5067- 5592 and Cat# 5067- 5593; or Agilent High Sensitivity DNA Kit to use with Agilent 2100 Bioanalyzer System, Cat#5067-4627 and Cat#5067-4626).
- PCR1 primers: CS1/CS2-target specific primers for gDNA and cDNA PCR1 barcoding (custom primers from Invitrogen; desalted).
- Sequencing primers for targeted genotyping libraries: CS1-seq, CS2-seq, CS1rc-seq, CS2rc-seq (custom LNA primers from Qiagen; HPLC purification).

- Nextera XT Kit Library Preparation Kit (Illumina , Cat#15032354) including i7 indexes and i5 indexes (Nextera XT Index Kit, Illumina , Cat#FC-131-1001).
- KAPA 2G Robust HS PCR Kit (Sigma Aldrich,Cat#KK5517)
- FastStart High Fidelity PCR System (Roche, REF:04738292001)
- Access Array™ Barcode Library for Illumina® Sequencers-384, Single Direction (Fluidigm, Cat#100-4876).
- Qubit (ThermoFisher, Cat. No. 32854)

Sorting and lysis – Timing: variable

1. First, prepare sufficient lysis buffer for the required number of cells for each experiment, plus 10% dead volume. Aliquot the lysis buffer (containing oligodT-ISPCR primer) into each well of a 96-well PCR plate (Thermo Scientific #AB-0900) in a clean environment dedicated to 'pre-amplification' work only. Cover with a PCR film and keep on ice/in the fridge until use. Lysis buffer should be prepared fresh on the day of sorting, maximum a few hours before use.

Lysis	1 cell	Storage	Cat. No./Supplier
Triton 0.4%	1.9 µL	-20 °C	Sigma-Aldrich # T8787, resuspended in DNAse/RNAse-Free water
RNAse Inhibitor	0.1 µL	-20 °C	TAKARA #2313A
dNTPs (10 mM)	1 µL	-20 °C	Life Technologies #R0192
Oligo-dT-ISPCR (10 µM)	1 µL	-20 °C	Biomers (custom oligo, HPLC purified)
Protease (1.09 AU/mL in water)	0.1 µL	+4 °C	Qiagen #19155; resuspend in UltraPure DNAse/RNAse-Free Distilled Water (Life Tech, #10977035)
ERCC RNA spike-in mix (1:2e6)	0.1 µL	-80 °C(single use aliquot)	Ambion #4456740
**TOTAL**	4.2 µL		

2. Prepare the sorter for single-cell sorting. Use single cell purity mode and keep the event rate low (less than 1000/s).

3. Check sorter alignment: use a 96 well tissue culture flat-bottom plate (Corning) and sort one fluorescent bead per well. Check under a fluorescent microscope that there is only one bead per well and that the position of the bead is centered. Note: don't add any media into the plate so that the bead stays in the place it was deposited by the sorter.

4. Use a 96-well PCR plate (Thermo Scientific #AB-0900, same model as the one in which cells will be sorted) covered with a PCR film, and sort 50 cells in positions 1A, 1H, 12A and 12H. Droplets should be positioned in the centre of the wells if the sorter is correctly aligned; if not, make necessary adjustments until drops are falling perfectly into each well.

5. After this initial check, remove the PCR film from the 96 PCR plate and sort 50 cells into columns 1 and 12 (wells 1A, 1B, 1C, 1D, 1E, 1F, 1G, 1H and 12A, 12B, 12C, 12D, 12E, 12F, 12G, 12H): drops should now be deposited at the very bottom of each well with no traces of liquid been left in the sides of each well. If correct, alignment checks are now complete.

6. Perform a purity sort of desired populations.

7. Sort cells directly into a 96 well PCR plate (Thermo Scientific #AB-0900) containing the lysis buffer, cover the plate with an aluminium PCR film (StarLab), spin down the plate and incubate 5 minutes at room temperature to allow for protease digestion. If sorting time is longer than 10 minutes, there is no need to incubate the plate further.

8. Put the plate directly into dry ice and store at -80 °C up to 1-2 months. (Processing plates after 3 months of -80 °C storage has shown decreased yield and/or signs of RNA degradation).

Heat inactivation, cDNA synthesis and amplification (RTPCR) – Timing: 6.5 hours; 1.5 hours hands-on time

9. Transport the plate(s) and TSO-LNA aliquot from -80 C storage on dry ice to a ‘pre-amplification’ dedicated workspace/clean room.

10. Thaw the 5X Buffer, RT-PCR Grade Water and any mRNA targeting primers that you might add to the mix. These can be thawed at room temperature. Aliquot them into an RNase free tube to prepare a master mix for the retrotranscription (RT) step as per the table below. RNase inhibitor, TSO-LNA and SMARTScribe enzyme will be added to the mix during heat inactivation step.

RT	1 cell (µL)	Storage	Cat. No.
**Buffer 5X**	2.00 µL	-20 °C	Clontech - Cat. No. 639537 (delivered with enzyme)
**RNase Inhibitor** (wait until the 72C step to add it)	0.25 µL	-20 °C	TAKARA – Cat#2313A
**TSO-LNA (100 µM)** (wait until 72C step to add it)	0.10 µL	-80 °C (aliquot into single use aliquots to avoid freeze/thaw cycles)	Custom TSO-LNA oligo from Exiqon-Qiagen (same as Picelli et al., 2013)
**RT-PCR Grade Water**	Variable	-20 °C	Life Tech - AM9935
**mRNA primers** (0.035 µL of each primer from a 200 uM stock)	Variable	-20 °C	Custom HPLC purified primers from biomers.net; resuspend in RNAse Free TE/water
**SMARTScribe** (wait until 72C step to add it)	1.00 µL	-20 °C	Clontech - Cat. No. 639537

**TOTAL**	5.60 µL		
**TOTAL (cumulative)**	9.80 µL		

11. Incubate the sample plate 15 minutes at 72 °C in a thermocycler (no RT mix has been added at this point). This step will inactivate the protease included in the lysis buffer so it doesn't interfere with any subsequent enzymatic steps.

12. During the heat inactivation time, add the RNAse Inhibitor, TSO-LNA and RT enzyme to the RT master mix on ice/cold block. Vortex and spin down.

13. Once the heat inactivation step is finished, take the plate out of the thermocycler, spin down and place into ice/cold rack. Aliquot 5.6 µL of RT mix into each well and carefully seal the plate with a PCR film (MicroAMp Clear Adhesive Film, Cat. No. 4306311). Note: it is essential that this step is performed within 5-7 minutes to avoid RNA degradation.

14. Spin down and run the following program in a thermocycler:

**Temperature**	**Time**	**Cycles**
42 C	90 min	1
50 C	2 min	10 cycles
42 C	2 min	
70 C	15 min	1
4 C	HOLD	-

15. Fifteen minutes before the RT program finishes, start thawing reagents to prepare the PCR master mix.

**PCR**	**1 cell (µL)**	**Storage**	**Cat. No.**
**2X Buffer**	12.50 µL	-20 °C	638509 - Clontech (delivered with enzyme
**ISPCR (10 µM)**	0.125 µL	-20 °C	Custom HPLC oligo from biomers.net (same as Picelli et al., 2013)
**RT-PCR Water**	Variable	-20 °C	Life Tech - AM9935
**SeqAMP Enzyme -** wait until RT is about to finish to add	0.50 µL	-20 °C	638509 - Clontech
**cDNA primers -** (0.035 µL from each primer from 20 µM stock)	Variable	-20 °C	Custom HPLC purified primers from biomers.net; resuspend in RNAse Free TE/water
**Genomic primers** (0.1 µL from each primer from a 200 µM stock)	Variable	-20 °C	
**TOTAL**	15.00 µL		
**TOTAL (cumulative)**	24.80 µL		

16. Once the RT program is finished, spin down the plate and add PCR master mix on ice/cold rack. Spin down the plate at 1000 g for 15 seconds. Take outside of the clean room workspace, place in a thermocycler and run the following program:

Temperature	Time	Cycles
98 C	3 min	
98 C	00:15	22 cycles (single HSPCs)
67 C	00:20	
72 C	6 min	
72 C	5 min	
4 C	HOLD	

Bead clean-up – Timing: 45 minutes

17. Add 16 µL of beads (Ampure XP Beads, Beckman Coulter, Cat. No. 391-02-501) into a V-shaped 96 well plate (AXYGEN, P-96-450V-C, 500 uL 96 well "V" bottom clear; Ref. 391-02-501).

18. Aliquot 11 µL of clean water (PCR grade) into the same V-shaped 96 well plate.

19. Aliquot 14 µL of each cDNA+amplicon mix into each well of the same plate and pipette up and down to mix the cDNA+amplicon mix with beads (0.6:1 beads to cDNA ratio). Incubate for 5 minutes at room temperature.

20. Incubate mixture on a 96-well magnetic stand for 2 minutes. Once the liquid is clear of beads, remove the liquid.

21. Wash the beads twice with 80 % EtOH (freshly prepared, dilute EtOH in PCR grade water). Add 100 µL of ethanol to each well, incubate for 30 seconds and remove. Repeat once more (2 times in total) and use P10 tips to remove any remaining ethanol.

22. Leave the beads to air-dry for 3 minutes. Be careful not to overdry the beads at this point or it will be difficult to resuspend them.

23. Resuspend the beads into 8 µL of EB Buffer (Qiagen Cat No./ID: 19086) with the plate off the magnet. Incubate for 30 seconds and put the plate back onto the magnet. Incubate into the magnet until the liquid is clear, then transfer 7.5 µL of purified cDNA library to a new plate for -20 C storage or further processing.

24. Check cDNA traces quality and size distributions using Bioanalyzer (Agilent), Fragment Analyzer Automated CE System (Advanced Analytical) or similar capillary system. Representative good quality cDNA traces are shown below (Figure MS1).

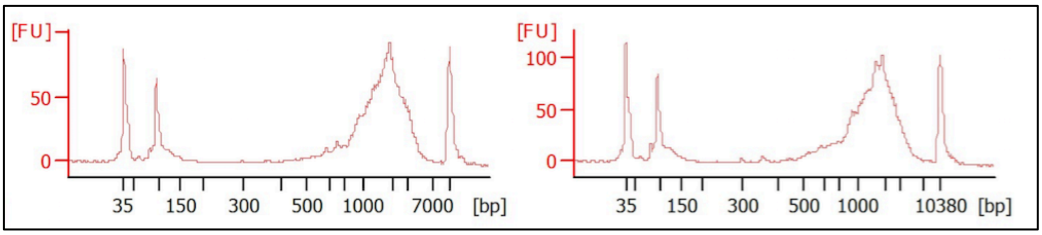

Figure MS1. Representative bead-purified cDNA traces from single HSPCs synthesized using full-length TARGET-seq in 96-well plates.

Whole transcriptome library preparation – Timing: 2 hours – 1.5 hours hands on time

25. Library preparation is performed using a commercially available Nextera XT Kit (FC-131-1096, Illumina) and commercially available i5 and i7 indeces (Nextera XT Index Kit, FC-131-1001, Illumina) using one fourth of the recommended volume. First, add 2.5 µL of Tagmentation Buffer into the required number of wells in a 96-well or 384-well plate (one well will be used for each cell).

26. Add 700 pg of bead-purified cDNA from each pool in a total volume of 1.25 µL and 1.25 µL of Amplicon Tagmentation Mix (ATM). Incubate 6 minutes at 55 C (total volume 5 µL).

**Reagents**	**1 reaction (µL)**
Tagmentation Buffer	2.5 µL
Bead purified cDNA (560 pg/µL)	1.25 µL
ATM (Amplicon Tagment Mix)	1.25 µL
**TOTAL**	5 µL

27. Once the incubation is finished, add 1.25 µL of NT (Neutralization) buffer to neutralize the tagmentation reaction.

28. Prepare PCR master mix as outlined below.

**Reagents**	**1 reaction (µL)**
i7 index (2 µM)	1.25 µL
i5 index (2 µM)	1.25 µL
NPM (PCR master mix)	3.75 µL
**TOTAL**	6.25 µL
**TOTAL (cumulative)**	12.5 µL

29. Incubate in a thermocycler and run the following PCR program:

**Temperature**	**Time**	**Cycles**
72 C	3 minutes	1
95 C	30 seconds	1
95 C	10 seconds	14 cycles

55 C	30 seconds	
72 C	30 seconds	
72 C	5 minutes	1
4 C	HOLD	1

30. Bead-purify barcoded and tagmented Nextera XT libraries using Ampure XP beads. First, dilute the product 1:1 with 12.5 µL of PCR-grade water. Aliquot each barcoded and tagmented library into a V-shaped 96 well plate (Cat. No. P-96-450V-C, Axygen) and aliquot 16 µL of pre-warmed (room temperature) Ampure XP beads (Beckman Coulter; Cat. No. A63881) into each well (0.6:1 beads to cDNA ratio). Incubate for 5 minutes at room temperature.

31. Incubate mixture into a 96-well magnetic stand for 2 minutes. Once the liquid is clear of beads, remove the liquid.

32. Wash the beads twice with 80 % EtOH (freshly prepared, dilute EtOH in PCR grade water). Add 100 µL of ethanol to each well, incubate for 30 seconds and remove. Repeat once more (2 times in total) and use P10 tips to remove any remaining ethanol.

33. Leave the beads to air-dry for 3 minutes. Be careful not to overdry the beads at this point or it will be difficult to resuspend them.

34. Resuspend the beads into 21 µL of EB Buffer (Qiagen Cat No./ID: 19086) with the plate off the magnet. Incubate for 30 seconds and put the plate back onto the magnet.

35. Incubate on the magnet until the liquid is clear of beads, then transfer 20 µL of purified tagmented/barcoded library to a new plate for -20 °C storage or further processing.

36. Run libraries on D5000 TapeStation or similar capillary array. Library fragments should be from 300 bp to 800 bp on average (Figure MS2):

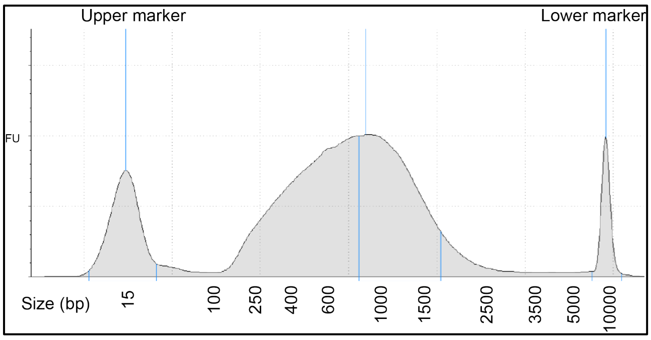

Figure MS2. Representative traces of tagmented, amplified and bead-purified full-length Nextera XT libraries.

37. Quantify tagmented and barcoded libraries using Qubit (ThermoFisher, Cat. No. 32854) and pool equimolar concentrations of each library. Quantify the final pool and sequence on a NextSeq/HiSeq platform.

Single cell genotyping library preparation for NGS – Timing: 5 hours – 2 hours hands on time

38. Take one aliquot of the unpurified cDNA+amplicon mix, dilute 1:2 with PCR Grade water and use as an input for the first barcoding PCR (PCR1). Perform an individual PCR reaction for each sample in a 384 well-plate (FrameStar 384, Cat. No. 4ti-0384/C). During this PCR reaction, target-specific primers attached to universal tags (CS1/CS2 adaptors) will be added to each amplicon from each sample, in order to prepare a targeted sequencing library. Targets with similar amplification efficiencies might be amplified simultaneously in the same reaction for the same single cell. Note: gDNA and cDNA pre-amplified amplicons don't have a cell-specific barcode at this stage; therefore, amplicons corresponding to each cell should be kept in individual wells of the 384 well-plate, taking precautions to avoid cross-well contamination.

39. Prepare PCR1 Mix and aliquot in the 384 well-plate using a Biomek FxP Liquid Handler (Beckman Coulter) of similar liquid handling platform:

**PCR1 BARCODING with target-specific primers**	**1 Reaction**	**Storage**	**Cat. No.**
KAPA 2G Ready Mix	3.125 µL	-20 °C	KAPA 2G Robust HS PCR Kit #KK5517
Primer F1+R1 (20 µM)	0.375 µL	-20 °C	Custom primers (Invitrogen) desalted, resuspend in TE
Primer F2+R2 (20 µM)	0.375 µL	-20 °C	
Primer F3+R3 (20 µM)	0.375 µL	-20 °C	
Primer FX+RX…	…	…	
RT-PCR Grade Water	Variable	-20 °C	UltraPure DNAse/RNAse-Free Distilled Water, (Life Technologies, #10977035)
cDNA aliquot	1.5 µL	-20 °C	
**TOTAL**	6.25 µL		

40. Incubate in a thermocycler and run the following PCR program:

**PCR1 PROGRAM**		
Temperature	Time (min:sec)	Cycles
95 C	03:00	1
95 C	00:15	20
60 C	00:20	
72 C	01:00	
72 C	05:00	1
4 C	HOLD	

41. Use 2.5 µL of PCR1 product as an input for the next reaction (PCR2). During this step, sample-specific barcodes are attached to previously tagged amplicons using the

Access Array™ Barcode Library for Illumina® Sequencers (384, Single Direction, Fluidigm). Barcode each sample in individual reactions.

42. Aliquot the barcodes (Access Array™ Barcode Library for Illumina® Sequencers) into a 384 well plate, and aliquot the PCR1 product into the same plate using a Biomek FxP Liquid Handler (Beckman Coulter) of similar liquid handling platform.

43. Prepare the PCR2 master mix and aliquot:

PCR2 BARCODING with Illumina compatible primers	1 Reaction	Storage	Cat. No.
FastStart High Fidelity 10X Reaction Buffer	1 µL	-20 °C	FastStart High Fidelity PCR System REF:04738292001
MgCl2 (25 mM)	1.8 µL	-20 °C	
DMSO	0.5 µL	-20 °C	
dNTP Mix (10 mM)	0.2 µL	-20 °C	
FastStart High Fidelity Enzyme (5U/µL)	0.1 µL	-20 °C	
RT-PCR Grade Water	1.90 µL	-20 °C	UltraPure DNAse/RNAse-Free Distilled Water, (Life Technologies, #10977035)
Single-direction barcodes (2 µM, Fluidigm)	2.0 µL	-20 °C	Access Array™ Barcode Library for Illumina® Sequencers-384, Single Direction, Fluidigm (Cat. No. 100-4876)
PCR1 barcoding aliquot	2.5 µL	-20 °C	
**TOTAL**	10 µL		

44. Incubate in a thermocycler and run the following PCR program:

PCR2 PROGRAM		
Temperature	Time (min:sec)	Cycles
95 C	10:00	1
95 C	00:15	10
60 C	00:30	
72 C	01:00	
72 C	03:00	1
4 C	HOLD	

45. Pool amplicons from each barcoded library using a liquid handling platform and use Ampure XP beads to clean-up pooled libraries (0.8:1 beads to cDNA ratio). Quantify

libraries using Qubit (ThermoFisher; Cat No. 32854) and check library size distribution and specific amplification of targeted amplicons on D1000 TapeStation or similar capillary array (Figure MS3). Note: barcodes and adaptors add 103 bp extra to the original PCR product.

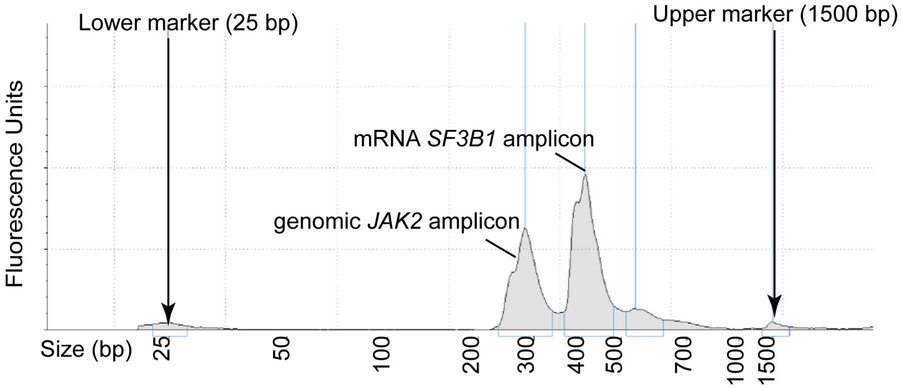

Figure MS3. Representative distributions of targeted amplicon libraries from genomic *JAK2* and mRNA *SF3B1* amplicons in a multiplexed reaction.

46. Libraries are ready for sequencing using custom sequencing primers targeted to CS1/CS2 tags (500 nM of CS1-seq and CS2-seq primers in a total volume of 700 µL for R1 and R2; 500 nM of CS1rc-seq and CS2rc-seq primers in a total volume of 700 µL for Index Read when using the MiSeq platform, Illumina). Note: CS1/CS2 and CS1rc/CS2rc sequencing primers contain LNA modifications (see Key Resources), as compared to CS1/CS2 tags used for PCR1 target-specific primers.

3’-TARGET-seq protocol in 384 well plates

Materials

- 96-well PCR plates (Thermo Fisher Scientific, AB-0900)
- 384 well PCR plates (FrameStar, 4titude, 4ti-0384/C)
- 384 well microplate (Corning® , CLS3702-100EA)
- RNase Free Microfuge Tubes (Invitrogen, AM12400)
- V-shaped 96 well plate (AXYGEN, P-96-450V-C, 500 uL 96 well "V" bottom clear; Ref. 391-02-501)
- PCR film (Thermo Fisher Scientific, MicroAMp Clear Adhesive Film, Cat. No. 4306311)
- Aluminium Sealing Film (StarLab, E2796-0792)
- 96-well magnetic stand (Invitrogen, AM10027)
- Protease (Qiagen, Cat#19155)
- Triton X-100 (Sigma-Aldrich, Cat#T8787)
- RNase inhibitor (TAKARA, Cat#2313A)
- dNTPs (Life Technologies, Cat#R0192)
- UltraPure DNAse/RNAse-Free Distilled Water (Life Technologies, Cat#10977035)
- EB Buffer (Qiagen, Cat No./ID: 19086)
- RNAse-free TE buffer (Invitrogen, Cat#AM9849)
- Ethanol
- ERCC aliquot (Ambion, Cat#4456740)
- SMARTScribe enzyme (Clontech - Cat. No. 639537).
- SeqAMP enzyme (Clontech, Cat#638509).
- RT-PCR Grade Water (Life Technologies, Cat#AM9935).
- Ampure XP beads (Beckman Coulter, Cat#A63881)
- Pre-amplification primers: oligodT-ISPCR barcoded primers, mRNA target-specific primers, TSO-LNA; gDNA target-specific primers, cDNA target-specific primers and ISPCR primers. Keep in dedicated “pre-amplification” area only. Custom primers from Biomers.net (oligodT-ISPCR barcoded primers, mRNA/gDNA/cDNA target-specific primers, ISPCR primers; HPLC purification) and Qiagen (TSO-LNA; RNAse free HPLC purification).
- PCR1 primers: CS1/CS2-target specific primers for gDNA and cDNA PCR1 barcoding (custom primers from Invitrogen; desalted).
- High Sensitivity NGS Fragment Analysis Kit (1bp - 6,000 bp; Agilent; Cat# DNF-474) or similar kit for capillary system (High Sensitivity D5000 ScreenTape System; Agilent, Cat# 5067- 5592 and Cat# 5067- 5593; or Agilent High Sensitivity DNA Kit to use with Agilent 2100 Bioanalyzer System, Cat#5067-4627, Cat#5067-4626).
- P5_index primer (custom oligonucleotide from Biomers.net; HPLC purified, contains PTO modifications; See Key Resources)
- Sequencing primers for targeted genotyping libraries: CS1-seq, CS2-seq, CS1rc-seq, CS2rc-seq (custom LNA primers from Qiagen; HPLC purification; See Key Resources).
- Sequencing primer: P5_SEQ (custom oligonucleotide from Biomers.net; PAGE purified, contains PTO modifications; See Key Resources).
- Nextera XT Kit Library Preparation Kit (Cat. No.15032354, Illumina) including i7 indexes (Illumina, Cat#FC-131-1001; alternatively, custom i7 primers can be used)

- KAPA 2G Robust HS PCR Kit (Sigma Aldrich,Cat#KK5517)
- FastStart High Fidelity PCR System (Roche, REF:04738292001)
- Access Array™ Barcode Library for Illumina® Sequencers-384, Single Direction (Fluidigm, Cat#100-4876).
- Qubit (ThermoFisher, Cat. No. 32854)

Sorting and lysis – Timing: variable

1. First, prepare a lysis buffer+oligodT stock plate. Sufficient lysis buffer (without barcoded oligo-dT) should be calculated to account for the required number of cells for each experiment plus 15% dead volume, and aliquoted into a 96-well PCR plate (Thermo Scientific #AB-0900) in a clean environment dedicated to 'preamplification' work only. Keep the lysis buffer on ice/in a cold rack. Lysis buffer should be prepared fresh on the day of sorting, maximum a few hours before use.

2. Aliquot each barcoded oligodT-ISPCR primer in each well of the same 96-well PCR plate (containing lysis buffer), using a liquid handling platform. The amount of barcoded-oligodT in each well is calculated as follows: (number of cells to be processed/96)*0.575 µL; i.e. if processing a total of 3000 cells, aliquot (3000/96)*0.575 µL of barcoded oligodT following the layout below (Figure MS4):

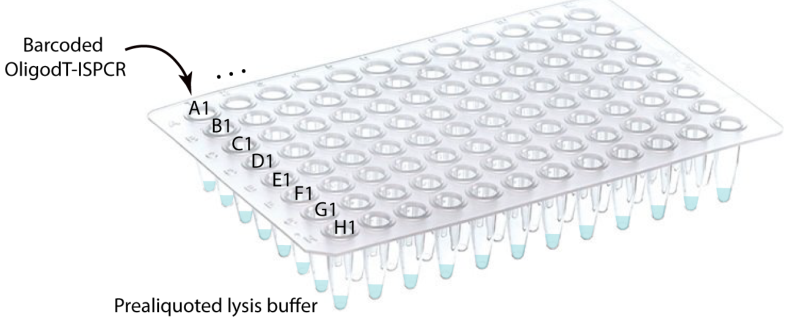

Figure MS4. Schematic representation of lysis buffer+barcoded oligodT stock plate preparation.

Lysis buffer	1 cell	Storage	Cat. No./Supplier
Triton 0.4%	0.95 µL	-20 °C	Sigma-Aldrich # T8787, resuspend in RNAse free water
RNAse Inhibitor	0.05 µL	-20 °C	TAKARA #2313A
dNTPs (10 mM)	0.5 µL	-20 °C	Life Technologies #R0192
Protease (1.09 AU/mL in water)	0.05 µL	+4 °C	Qiagen #19155; resuspend in UltraPure DNAse/RNAse-Free Distilled Water
ERCC RNA spike-in mix (1:4e5)	0.02 µL	-80 °C (single use aliquot)	Ambion #4456740
**TOTAL**	**1.57 µL**		
Oligo-dT-ISPCR (10 µM) – barcoded, well-specific	0.5 µL	-20 °C	Custom HPLC primers, Biomers.net
**TOTAL**	**2.07 µL**		

3. Aliquot the mixture of lysis buffer+barcoded oligodT into each well of a 384 well-plate (FrameStar) following the layout below using a Biomek FxP Liquid Handler

(Beckman Coulter) of similar liquid handling platform. Each barcode will be aliquoted four times per plate, once in each quadrant (Figure MS5). Cover the plates with a PCR film and keep them on ice/in the fridge until use. Alternatively, sets of 384 barcoded oligodTs might be used.

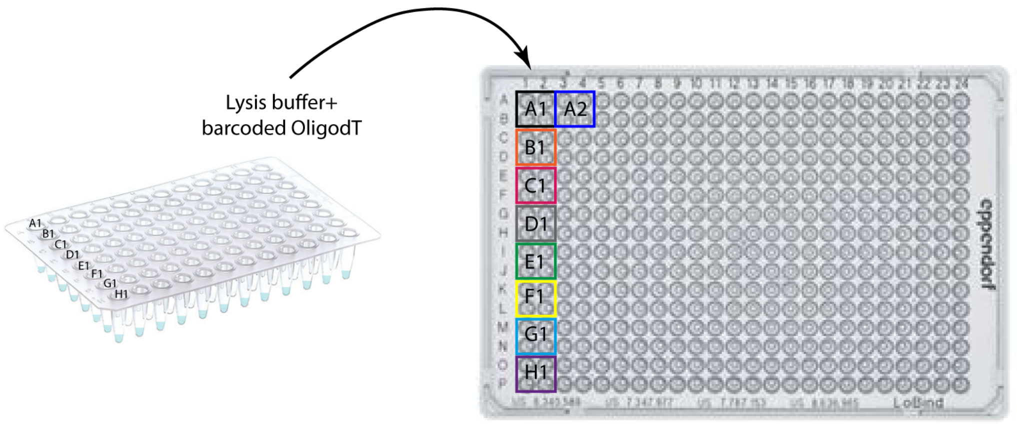

Figure MS5. Schematic representation of lysis buffer+barcoded oligodT aliquoting into 384 well plates.

4. Prepare the sorter for single-cell sorting. Use single cell purity mode and keep the event rate low (less than 1000/s).

5. Perform an alignment test sort using a 384 well plate (Corning® 384 well microplate, CLS3702-100EA) and sort one fluorescent bead per well. Check under a fluorescent microscope that there is only one bead in each well and that the position of the bead is centered at the very bottom of the plate. If correct, alignment checks are now complete.

6. Check sorter alignment: use a 384-well PCR plate (FrameStar, same model as the one in which single cells will be sorted) covered with a PCR film, and sort 50 cells in the four corners of the plate (positions 1-A, 1-P, 24-A and 24-P). Droplets should be positioned in the centre of the wells if the sorter is correctly aligned; if not, make necessary adjustments until drops are falling perfectly into each well. After this initial check, remove the PCR film and sort 50 cells into the same four corners of the 384 plate: drops should now be deposited at the very bottom of each well with no traces of liquid been left in the sides of each well.

7. Sort cells directly into a 384 well PCR plate (FrameStar) containing lysis buffer+barcoded oligodT-ISPCR, cover the plate with an aluminium PCR film (StarLab), spin down the plate and incubate 5 minutes at room temperature to allow for protease digestion. If sorting time is longer than 10 minutes, there is no need to incubate the plate further.

8. Put the plate directly into dry ice and store at -80 °C up to 1-2 months. (Processing plates after 3 months of -80 °C storage has shown decreased yield and/or signs of RNA degradation).

Heat inactivation, cDNA synthesis and amplification (RTPCR) – Timing: 6.5 hours; 1.5 hours hands-on time

9. Transport the plate(s) and TSO-LNA aliquot from -80 °C storage on dry ice to a ‘pre-amplification’ dedicated workspace/clean room.

10. Thaw the 5X Buffer, RT-PCR Grade Water and any mRNA targeting primers that you might add to the mix. These can be thawed at room temperature. Aliquot them into an RNase free tube to prepare a master mix for the retrotranscription (RT) step as per the table below. RNase inhibitor, TSO-LNA and SMARTScribe enzyme will be added to the mix during heat inactivation step.

RT	1 cell	Storage	Cat. No.
**Buffer 5X**	1.00 µL	-20 °C	Clontech - Cat. No. 639537 (delivered with enzyme)
**RNase Inhibitor** (wait until the 72C step to add it)	0.125 µL	-20 °C	TAKARA - 2313A
**TSO-LNA (100 uM) -** wait until 72C step to add it	0.05 µL	-80 °C	Custom TSO-LNA oligo from Exiqon-Qiagen (same as Picelli et al., 2013); avoid freeze/thaw cycles
**RT-PCR Grade Water**	Variable	-20 °C	Life Tech - AM9935
**mRNA primers** (0.0175 µL of each primer from a 200 µM stock)	Variable	-20 °C	Custom HPLC purified primers from biomers.net; resuspend in RNAse Free TE/water
**SMARTScribe enzyme-** wait until 72C step to add it	0.5 µL	-20 °C	Clontech - Cat. No. 639537
**TOTAL**	2.80 µL		
**TOTAL (cumulative)**	4.87 µL		

11. Incubate the sample plate 15 minutes at 72 °C in a thermocycler (no RT mix has been added at this point). This step will inactivate the protease included in the lysis buffer so it doesn’t interfere with any subsequent enzymatic steps.

12. During the heat inactivation, add the RNAse Inhibitor, TSO-LNA and RT enzyme to the RT master mix on ice/cold block. Vortex and spin down.

13. Once the heat inactivation step is finished, take the plate out of the thermocycler, spin down and place into ice/cold rack. Aliquot 2.8 µL of RT mix into each well and carefully seal the plate with a PCR film (MicroAMp Clear Adhesive Film, Cat. No. 4306311). Note: it is essential that this step is performed within 5-7 minutes to avoid RNA degradation.

14. Spin down and run the following program in a thermocycler:

Temperature	Time	Cycles
42 C	90 min	1
50 C	2 min	10 cycles
42 C	2 min	
70 C	15 min	1
4 C	HOLD	-

15. Fifteen minutes before the RT program finishes, start thawing reagents to prepare the PCR master mix.

PCR	1 cell	Storage	Cat. No.
**2X Buffer**	6.25 µL	-20 °C	Clontech Cat#638509 (delivered with enzyme)
**ISPCR (10 µM)**	0.0625 µL	-20 °C	Custom HPLC oligo from biomers.net (same as Picelli et al., 2013)
**RT-PCR Water**	Variable	-20 °C	Life Technologies #AM9935
**SeqAMP Enzyme -** wait until RT is about to finish to add	0.25 µL	-20 °C	Clontech Cat#638509
**cDNA primers -** (0.0175 µL from each primer from 20 µM stock)	Variable	-20 °C	Custom HPLC purified primers from biomers.net; resuspend in RNAse Free TE/water
**Genomic primers** (0.05 µL from each primer from a 200 µM stock)	Variable	-20 °C	
**TOTAL**	7.50 µL		
**TOTAL (cumulative)**	12.37 µL		

16. Once the RT program is finished, spin down the plate and add PCR master mix on ice/cold rack. Spin down the plate at 1000 g for 15 seconds. Take outside of the clean room workspace, place in a thermocycler and run the following program:

Temperature	Time	Cycles
98 C	3 min	1
98 C	00:15	24 cycles (single HSPCs)
67 C	00:20	
72 C	6 min	
72 C	5 min	1
4 C	HOLD	1

Pooling and bead clean-up – Timing: 45 minutes

17. Pool 1 µL of amplified cDNA+amplicon mix from each uniquely-barcoded well of the 384 well-plate into an eppendorf tube using a liquid handler platform. Four pools should be made from each 384-well plate, corresponding to 96 cells of each quadrant of uniquely-barcoded wells. Once pooled, perform bead purification. Aliquot 80 µL of pooled cDNA into a V-shaped 96 well plate (Cat. No. P-96-450V-C, Axygen) and aliquot 48 µL pre-warmed Ampure XP beads (Beckman Coulter;

Cat. No. A63881) into each well (0.6:1 beads to cDNA ratio). Incubate for 5 minutes at room temperature. Note that whilst polyadenylated cDNA has been uniquely-barcoded, amplicons corresponding to each single cell don’t contain unique barcodes and therefore precaution should be taken to avoid cross-contamination between wells at this stage.

18. Incubate mixture into a 96-well magnetic stand for 2 minutes. Once the liquid is clear of beads, remove the liquid.

19. Wash the beads twice with 80 % EtOH (freshly prepared, dilute EtOH in PCR grade water). Add 100 µL of ethanol to each well, incubate for 30 seconds and remove. Repeat once more (2 times in total) and use P10 tips to remove any remaining ethanol.

20. Leave the beads to air-dry for 3 minutes. Be careful not to overdry the beads at this point or it will be difficult to resuspend them. Resuspend the beads in 80 µL and repeat the bead purification step: add 48 µL of beads to the cleaned product, wash twice with ethanol and remove any remaning ethanol using P10 tips.

21. Resuspend the beads into 21 µL of EB Buffer (Qiagen Cat No./ID: 19086) with the plate off the magnet. Incubate for 30 seconds and put the plate back onto the magnet.

22. Incubate on the magnet until the liquid is clear of beads, then transfer 20 µL of purified cDNA library to a new plate for -20 °C storage or further processing.

23. Check cDNA traces quality and size distributions using Bioanalyzer (Agilent), Fragment Analyzer Automated CE System (Advanced Analytical) or similar capillary arrays (Figure MS6). If primer dimers are detected at this stage (100-300 bp peaks), libraries should be re-purified with Ampure XP beads.

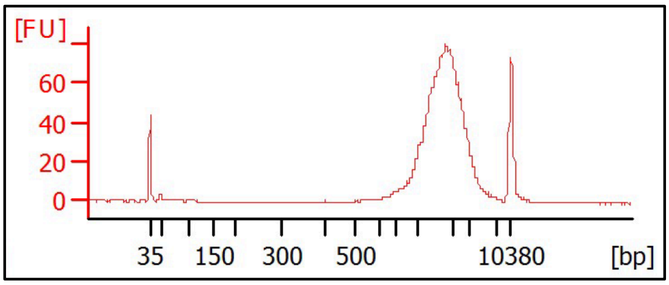

Figure MS6. Representative cDNA traces of pooled and bead-purified amplified cDNA libraries from 96 HSPCs.

Whole transcriptome library preparation – Timing: 45 minutes

24. Library preparation is performed using a commercially available Nextera XT Kit (FC-131-1096, Illumina) with modifications in the indexing PCR step. First, prepare one tube for each bead-purified cDNA pool and add 10 µL of Tagmentation Buffer into each tube.

25. Add 1 ng of purified cDNA from each pool, up to a total volume of 5 µL and 5 µL of Amplicon Tagmentation Mix (ATM). Incubate 6 minutes at 55 C (total volume 20 µL).

Reagents	1 reaction (µL)
Tagmentation Buffer	10
Bead purified cDNA (0.2 ng/uL)	5
ATM (Amplicon Tagment Mix)	5
**TOTAL**	20

26. Once the incubation is finished, add 5 µL of NT buffer to neutralize the tagmentation reaction.

27. Prepare PCR master mix as outlined below. i7 index primers are commercially available (Illumina, Cat#FC-131-1001); P5_index primer is a custom indexing primer (see Key Resources).

Reagents	1 reaction (µL)
i7 index (2 µM)	5 µL
P5_index (10 µM)	1 µL
NPM (PCR master mix)	15 µL
Water	4 µL
**TOTAL**	25 µL
**TOTAL (cumulative)**	50 µL

28. Incubate in a thermocycler and run the following PCR program:

Temperature	Time	Cycles
72 C	3 minutes	1
95 C	30 seconds	1
95 C	10 seconds	14 cycles
55 C	30 seconds	
72 C	30 seconds	
72 C	5 minutes	1
4 C	HOLD	1

29. Bead-purify tagmented libraries twice using Ampure XP beads. For the first bead purification step, use 34 µL of beads and 50 µL of library - resuspend in 34 µL of EB buffer (Qiagen) and use the product to perform a second bead purification using 20 µL of beads. Resuspend the final product in a total volume of 20 µL of EB buffer (Qiagen).

30. Run libraries on D5000 TapeStation or similar capillary array. Library fragments should be from 300 bp to 800 bp on average (Figure MS7):

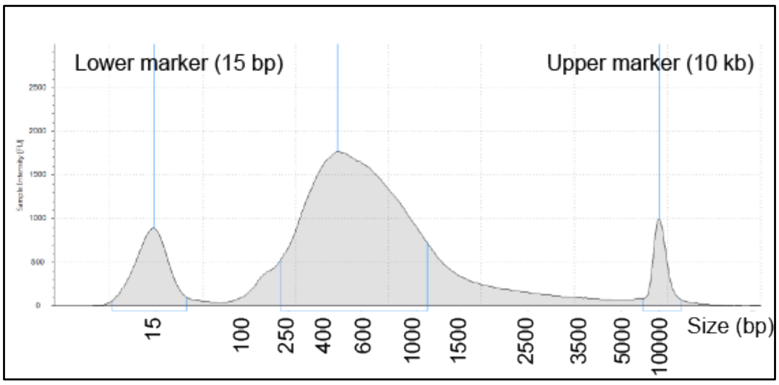

Figure MS7. Representative traces of tagmented, amplified and bead-purified 3'-TARGETseq Nextera XT libraries.

31. Quantify tagmented and barcoded libraries using Qubit (ThermoFisher, Cat. No. 32854) and pool equimolar concentrations of each tagmented library. Quantify the final pool and sequence on a NextSeq/HiSeq platform using custom P5_SEQ sequencing primer for Read1 (See Key Resources). Index read and Read2 use standard sequencing primers provided within the commercially-available sequencing cartridge. If using the NextSeq platform, load a 3 pM library diluted in 1.3 mL of HT1 Buffer (Illumina) and 900 nM of P5_SEQ primer in a total volume of 3 mL of HT1 buffer.

Single cell genotyping library preparation for NGS – Timing: 5 hours – 2 hours hands on time

32. Take one aliquot of the unpurified cDNA-amplicon mix, dilute 1:2 with PCR Grade water and use as an input for the first barcoding PCR (PCR1). Perform an individual PCR reaction for each sample in a 384 well-plate (FrameStar 384, Cat. No. 4ti-0384/C). During this PCR reaction, target-specific primers attached to universal tags (CS1/CS2 adaptors) will be added to each amplicon from each sample, in order to prepare a targeted sequencing library. Targets with similar amplification efficiencies might be amplified simultaneously in the same reaction for the same single cell. Note: while oligodT-primed mRNA molecules carry a cell-specific barcode, gDNA and cDNA pre-amplified amplicons will not have a cell-specific barcode and, therefore, amplicons corresponding to each cell should be kept in individual wells of the 384 well-plate, taking precautions to avoid cross-well contamination.

33. Prepare PCR Mix and aliquot in the 384 well-plate using a Biomek FxP Liquid Handler (Beckman Coulter) of similar liquid handling platform:

PCR1 BARCODING with target-specific primers	1 Reaction	Storage	Cat. No.
KAPA 2G Ready Mix	3.125 µL	-20 °C	KAPA 2G Robust HS PCR Kit #KK5517
Primer F1+R1 (20 uM)	0.375 µL	-20 °C	Custom primers (Invitrogen) cartridge purification, resuspend in TE
Primer F2+R2 (20 uM)	0.375 µL	-20 °C	
Primer F3+R3 (20 uM)	0.375 µL	-20 °C	
Primer FX+RX…	…	…	

RT-PCR Grade Water	Variable	-20 °C	UltraPure DNAse/RNAse-Free Distilled Water, (Life Technologies, #10977035)
cDNA aliquot	1.5 μL	-20 °C	
**TOTAL**	6.25 μL		

34. Incubate in a thermocycler and run the following PCR program:

**PCR1 PROGRAM**		
Temperature	Time (min:sec)	Cycles
95 C	03:00	1
95 C	00:15	20
60 C	00:20	
72 C	01:00	
72 C	05:00	1
4 C	HOLD	

35. Use 2.5 μL of PCR1 product as an input for the next reaction (PCR2). During this step, sample-specific barcodes are attached to previously tagged amplicons using the Access Array™ Barcode Library for Illumina® Sequencers (384, Single Direction, Fluidigm). Barcode each sample in individual reactions.

36. Aliquot the barcodes (Access Array™ Barcode Library for Illumina® Sequencers) into a 384 well plate, and aliquot the PCR1 product into the same plate using a liquid handling platform.

37. Prepare the PCR master mix and aliquot.

**PCR2 BARCODING with Illumina compatible primers**	**1 Reaction**	**Storage**	**Cat. No.**
FastStart High Fidelity 10X Reaction Buffer	1 μL	-20 °C	FastStart High Fidelity PCR System REF:04738292001
MgCl2 (25 mM)	1.8 μL	-20 °C	
DMSO	0.5 μL	-20 °C	
dNTP Mix (10 mM)	0.2 μL	-20 °C	
FastStart High Fidelity Enzyme (5U/μL)	0.1 μL	-20 °C	
RT-PCR Grade Water	1.90 μL	-20 °C	UltraPure DNAse/RNAse-Free Distilled Water, (Life Technologies, #10977035)

Single-direction barcodes (2 uM, Fluidigm)	2.0 µL	-20 °C	Access Array™ Barcode Library for Illumina® Sequencers-384, Single Direction, Fluidigm (Cat. No. 100-4876)
PCR1 barcoding aliquot	2.5 µL	-20 °C	
**TOTAL**	10 µL		

38. Incubate in a thermocycler and run the following PCR program:

**PCR2 PROGRAM**		
Temperature	Time (min:sec)	Cycles
95 C	10:00	1
95 C	00:15	10
60 C	00:30	
72 C	01:00	
72 C	03:00	1
4 C	HOLD	

39. Pool amplicons from each barcoded library using a liquid handling platform and use Ampure XP beads to clean-up pooled libraries (0.8:1 beads to cDNA ratio). Quantify libraries using Qubit (ThermoFisher; Cat No. 32854) and check library size distribution and specific amplification of targeted amplicons on D1000 TapeStation or similar capillary array (Figure MS8). Note: barcodes and adaptors add 103 bp extra to the original PCR product.

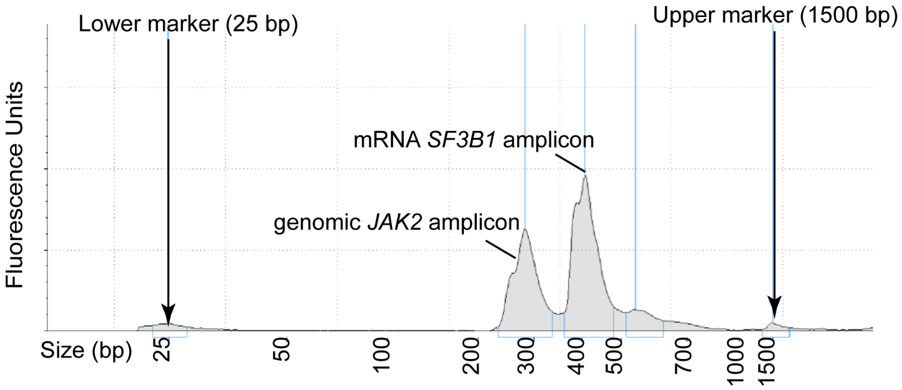

Figure MS8. Representative distributions of targeted amplicon libraries from genomic *JAK2* and mRNA *SF3B1* amplicons in a multiplexed reaction.

40. Libraries are ready for sequencing using custom sequencing primers targeted to CS1/CS2 tags (500 nM of CS1-seq and CS2-seq primers in a total volume of 700 µL for R1 and R2; 500 nM of CS1rc-seq and CS2rc-seq primers in a total volume of 700 µL for Index Read when using the MiSeq platform, Illumina). Note: CS1/CS2 and CS1rc/CS2rc sequencing primers contain LNA modifications (see Key Resources), as compared to CS1/CS2 tags used for PCR1 target-specific primers.

Primer design and validation technical note.

Pre-amplification (RTPCR) primer design

Primers should be designed taking into account the following considerations:

- Design genomic primers binding to at least one intronic region so they are compatible with parallel cDNA amplification.
- Primers for gDNA amplification should be checked for specificity against genomic and transcriptome references (so they are compatible with parallel cDNA amplification) using Primer BLAST (https://www.ncbi.nlm.nih.gov/tools/primer-blast/) or similar tools.
- Design mRNA/cDNA primers ideally in the exon before and the exon after your mutation/region of interest. An example of *JAK2* primer design can be found below (Figure MS9).

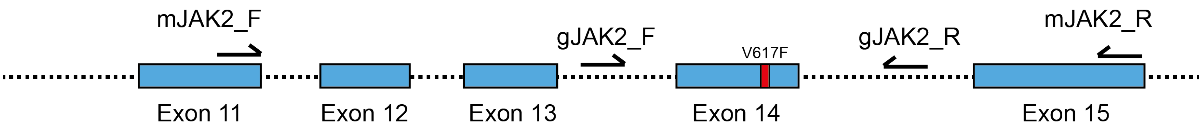

Figure MS9. Schematic representation of mRNA and gDNA *JAK2* primer design.

- Primers for mRNA/cDNA amplification should be checked for specificity against transcriptome references using Primer BLAST or similar tools.
- Design amplicons ideally ranging from 250 bp to 700 bp long. We have tested amplicons up to 1 kb, which worked optimally. In the rare event in which exons are longer than 1 kb, the preferred option is to design a unique primer pair spanning the mutation of interest.
- In the event that mutations of interest are in terminal exons or 3'-UTR regions, design two forward primers (mRNA and gDNA specific) and one unique reverse primer, which will amplify both types of amplicons. An example of *ASXL1* primer design can be found below (Figure MS10).

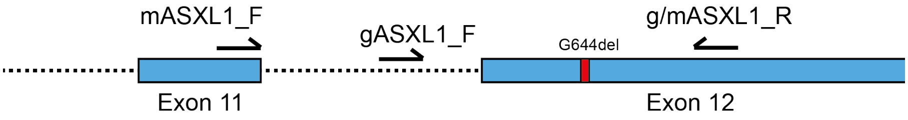

Figure MS10. Schematic representation of mRNA and gDNA *ASXL1* primer design.

cDNA primers used in the PCR step contain the same primer sequence used for mRNA primers in the RT step, but are attached to ISPCR adaptors (ISPCR adaptor sequence: 5'- AAGCAGTGGTATCAACGCAGAGT-3') in the 5'-end of each primer. This increases amplification efficiency of cDNA targets. Importantly, in the specific case of terminal exons where a common primer is used to amplify both cDNA and gDNA molecules, cDNA primers used in the PCR step should not be attached to ISPCR adaptors, as this will create concatemers that will disrupt the successful generation of cDNA libraries.

Pre-amplification primer validation

Primers used for gDNA and mRNA/cDNA pre-amplification should be validated for specificity using bulk gDNA and bulk cDNA, respectively. If primers are not specific or they present low amplification efficiencies, they should be redesigned.

Primer multiplexing strategies for pre-amplification should be validated for the generation of excessive primer dimers and concatemers in a minimum of 8 single cell samples. Examples of a good and bad library (primers generating concatemers) are shown below (Figure MS11). Figure MS11a represents a good quality cDNA library; Figure MS11b represents a good quality library despite high primer dimer concentration and Figure MS11c represents a bad quality cDNA library in which primers are interfering with cDNA amplification.

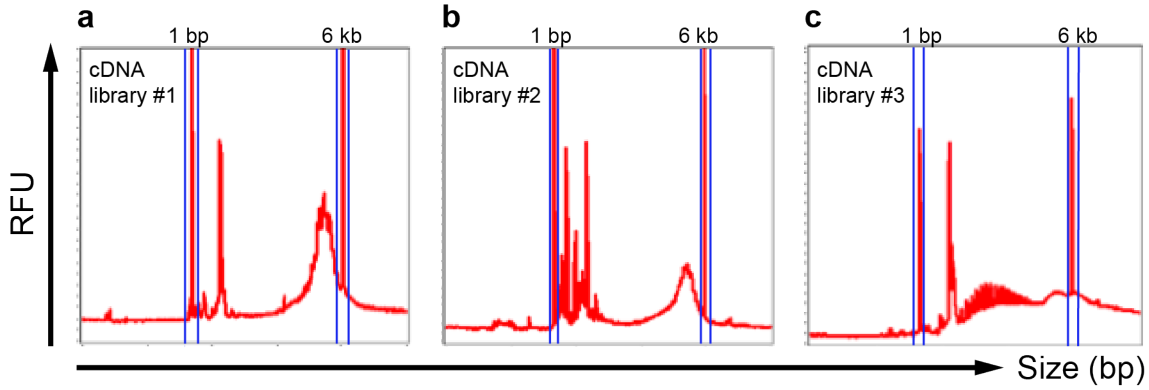

Figure MS11. Representative cDNA libraries from single HSPCs produced using different primer multiplex combinations.

When primer combinations generate concatemers or greatly decrease cDNA yield, such as the case presented in Figure MS11c, each primer pair should be tested individually in single cells and those pairs originating concatemers should be redesigned. Alternatively, when mRNA primer pairs generate concatemers, mRNA primer concentration might be decreased, down to a minimum 35 nM in the RT mix. gDNA primer concentration should not be decreased.

Custom barcoding primer design (PCR1 barcoding primers)

Specific primers for gDNA and cDNA used during PCR1-barcoding should be designed nested from the original RT+PCR amplicon (pre-amplification primers) to increase specific amplification and PCR efficiency. Specificity should be checked against transcriptome references for both types of molecules, and they should be validated using bulk genomic DNA and cDNA, respectively. Primers are tagged to CS1/CS2 universal adaptors in the 5'end (Forward adaptor, CS1: ACACTGACGACATGGTTCTACA; Reverse adaptor, CS2: TACGGTAGCAGAGACTTGGTCT), which will be used to add cell-specific barcodes during PCR2 step.

PCR1 primers should be different for each type of molecule (gDNA or cDNA), so that independent mutational readouts can be obtained from each, bioinformatically extracting reads matching each primer sequence. In the specific case of terminal exons, whilst one unique reverse primer was used during RT-PCR steps, two reverse primers should be

used for PCR1 barcoding, and therefore gDNA and mRNA amplicons should be barcoded in different reactions in such case.

When sequencing using a sequencing platform with 300 cycles configuration (150 bp R1 and 150 bp R2), primers should be designed taking into account the relative distance of the mutation to start of the primer, so that the mutation is well covered during sequencing. Sequencing configurations with shorter reads are not recommended.

Custom barcoding primer validation

Primers used for gDNA and cDNA PCR1-barcoding should be validated for specificity and amplification efficiency in pre-amplified single cell samples. To do that, PCR1 should be performed individually for each target using 35 cycles of PCR amplification, and specific amplification should be checked on a Fragment Analyzer platform (Agilent) or similar capillary array (Figure MS12). Amplification efficiency might be derived from the quantification of specific product obtained in each case. Alternatively, specificity and amplification efficiency might be assessed with qPCR. If primers are not specific, they should be redesigned.

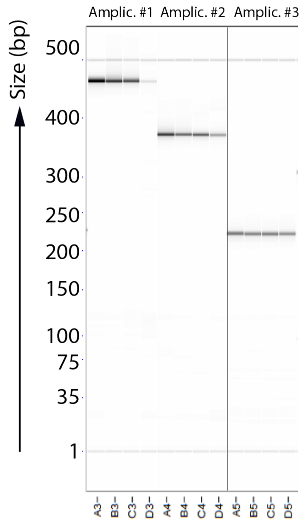

Figure MS12. Representative example of Fragment Analyzer results for three different PCR1 primer pairs.

Example of primers used for *JAK2*-V617F amplification

Primer Name	Primer sequence	Step	Type	Purification
mJAK2_F	TAAATGCTGTCCCCCAAAGC	RT	mRNA	HPLC
mJAK2_R	CCATGCCAACTGTTTAGCAAC	RT	mRNA	HPLC
gJAK2_F	ccaaagcacattgtatcctcatct	PCR	gDNA	HPLC
gJAK2_R	cactgacacctagctgtgatcct	PCR	gDNA	HPLC
ISPCR_mJAK2_F	AAGCAGTGGTATCAACGCAGAGTTAAATGCTGTCCCCCAAAGC	PCR	cDNA	HPLC
ISPCR_mJAK2_R	AAGCAGTGGTATCAACGCAGAGTCCATGCCAACTGTTTAGCAAC	PCR	cDNA	HPLC
mJAK2_PCR1_F	ACACTGACGACATGGTTCTACATCTGGATAAAGCACACAGAAACT	PCR1	cDNA	Desalted
mJAK2_PCR1_R	TACGGTAGCAGAGACTTGGTCTTCCAAATTTTACAAACTCCTGAACC	PCR1	cDNA	Desalted
gJAK2_PCR1_F	ACACTGACGACATGGTTCTACAttatggacaacagtcaaacaacaa	PCR1	gDNA	Desalted
gJAK2_PCR1_R	TACGGTAGCAGAGACTTGGTCTaaaggcattagaaagcctgtagt	PCR1	gDNA	Desalted

ISPCR adaptor is labelled in orange; CS1/CS2 adaptors are labelled in blue
