# Supplementary material for: Unravelling Intratumoral Heterogeneity through High-Sensitivity Single-Cell Mutational Analysis and Parallel RNA Sequencing
Source: Mol Cell. 2019 Mar 21;73(6):1292–1305.e8. doi: 10.1016/j.molcel.2019.01.009 (PMC6436961; doi:10.1016/j.molcel.2019.01.009)
Supplement: Document S2. Article plus Supplemental Information [file mmc8.pdf]

Molecular Cell

Unravelling Intratumoral Heterogeneity through High-Sensitivity Single-Cell Mutational Analysis and Parallel RNA Sequencing

Graphical Abstract

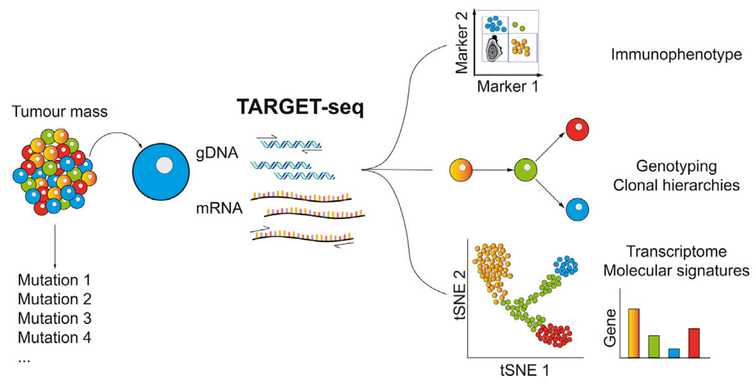

Highlights

- Conventional scRNA-seq protocols do not allow reliable mutational analysis
- TARGET-seq combines high-sensitivity genomic DNA and cDNA genotyping with scRNA-seq
- TARGET-seq resolves the distinct transcriptional signatures of tumor genetic subclones
- Non-mutant cells from patients show aberrant, inflammation-associated gene expression

Authors

Alba Rodriguez-Meira, Gemma Buck, Sally-Ann Clark, ..., Sten Eirik W. Jacobsen, Supat Thongjuea, Adam J. Mead

Correspondence

adam.mead@imm.ox.ac.uk

In Brief

Rodriguez-Meira et al. developed TARGET-seq, a method for high-sensitivity mutational analysis and parallel RNA sequencing from the same single cell. Applied to 4,559 single cells, TARGET-seq unraveled transcriptional and genetic tumor heterogeneity in myeloproliferative neoplasm (MPN) stem and progenitor cells. TARGET-seq is a powerful tool for resolving the molecular signatures of genetically distinct tumor subclones.

Rodriguez-Meira et al., 2019, Molecular Cell *73*, 1292–1305
March 21, 2019 © 2019 The Author(s). Published by Elsevier Inc.
https://doi.org/10.1016/j.molcel.2019.01.009

Molecular Cell
Technology

Unravelling Intratumoral Heterogeneity through High-Sensitivity Single-Cell Mutational Analysis and Parallel RNA Sequencing

Alba Rodriguez-Meira,[1,2] Gemma Buck,[1,2] Sally-Ann Clark,[3] Benjamin J. Povinelli,[1,2] Veronica Alcolea,[1,2] Eleni Louka,[1,2] Simon McGowan,[4] Angela Hamblin,[5] Nikolaos Sousos,[1,2] Nikolaos Barkas,[1,2] Alice Giustacchini,[1,2] Bethan Psaila,[1,2,5] Sten Eirik W. Jacobsen,[1,2,6,7,8] Supat Thongjuea,[2,4] and Adam J. Mead[1,2,5,9,*]
[1]Haematopoietic Stem Cell Biology Laboratory, Medical Research Council Weatherall Institute of Molecular Medicine, University of Oxford, Oxford OX3 9DS, UK
[2]Medical Research Council Molecular Haematology Unit, Weatherall Institute of Molecular Medicine, University of Oxford, Oxford OX3 9DS, UK
[3]Flow Cytometry Facility, Medical Research Council, Weatherall Institute of Molecular Medicine, University of Oxford, Oxford OX3 9DS, UK
[4]Medical Research Council Centre for Computational Biology, Weatherall Institute of Molecular Medicine, University of Oxford, Oxford OX3 9DS, UK
[5]National Institute for Health Research Biomedical Research Centre, University of Oxford, Oxford, UK
[6]Department of Cell and Molecular Biology, Wallenberg Institute for Regenerative Medicine, Karolinska Institutet, Stockholm, Sweden
[7]Karolinska University Hospital, Stockholm, Sweden
[8]Department of Medicine Huddinge, Center for Hematology and Regenerative Medicine, Karolinska Institutet, Stockholm, Sweden
[9]Lead Contact
*Correspondence: adam.mead@imm.ox.ac.uk
https://doi.org/10.1016/j.molcel.2019.01.009

SUMMARY

Single-cell RNA sequencing (scRNA-seq) has emerged as a powerful tool for resolving transcriptional heterogeneity. However, its application to studying cancerous tissues is currently hampered by the lack of coverage across key mutation hotspots in the vast majority of cells; this lack of coverage prevents the correlation of genetic and transcriptional readouts from the same single cell. To overcome this, we developed TARGET-seq, a method for the high-sensitivity detection of multiple mutations within single cells from both genomic and coding DNA, in parallel with unbiased whole-transcriptome analysis. Applying TARGET-seq to 4,559 single cells, we demonstrate how this technique uniquely resolves transcriptional and genetic tumor heterogeneity in myeloproliferative neoplasms (MPN) stem and progenitor cells, providing insights into deregulated pathways of mutant and non-mutant cells. TARGET-seq is a powerful tool for resolving the molecular signatures of genetically distinct subclones of cancer cells.

INTRODUCTION

Resolving intratumoral heterogeneity (ITH) is critical for our understanding of tumor evolution and resistance to therapies; this understanding, in turn, is required for the development of effective cancer treatments and biomarkers for precision medicine (McGranahan and Swanton, 2017). The best-characterized source of ITH has been at the genetic level; this heterogeneity has been identified through advances in next-generation sequencing (NGS) techniques at the bulk and single-cell levels (Vogelstein et al., 2013). However, certain factors beyond somatic mutations contribute to ITH. For example, some tumors are hierarchically organized and contain cancer stem cells (CSCs), which propagate disease relapse. The genetic events underlying tumor evolution originate in CSCs, which in some tumors are rare within the total tumor bulk population (Clevers, 2011; Magee et al., 2012; Woll et al., 2014). Furthermore, the CSCs' normal cellular counterparts, which lack genetic mutations, can be difficult to distinguish from malignant cells because they might share phenotypic features, but these cells can nevertheless be informative for disease biology (Giustacchini et al., 2017). Consequently, resolving ITH requires methods that allow these multiple layers of heterogeneity to be teased apart.

A potentially powerful approach for gaining a better understanding of the functional consequences of ITH is to link genetic ITH with the transcriptional signatures of distinct subpopulations of tumor cells. A number of studies have begun to apply single-cell RNA sequencing (scRNA-seq) to characterize different malignancies, demonstrating the power of scRNA-seq to identify the different cell types that are encompassed within a tumor, including cells with "stemness" signatures and characterization of developmental hierarchies of tumor cells (Patel et al., 2014; Tirosh et al., 2016a, 2016b; Venteicher et al., 2017). However, although scRNA-seq approaches can readily resolve such transcriptional heterogeneity, current techniques do not allow parallel mutational analysis because of a lack of coverage across mutation hotspots (Kiselev et al., 2017; Patel et al., 2014; Tirosh et al., 2016b). This integration of mutational and transcriptional information is crucial for linking genetic evolution events to the cell of origin; this is of considerable

 © 2019 The Author(s). Published by Elsevier Inc.
This is an open access article under the CC BY license (http://creativecommons.org/licenses/by/4.0/).

importance because serial mutation acquisition might occur within distinct and developmentally ordered stem and progenitor cell types, as described in acute leukemia (Jan et al., 2012). Furthermore, mutation analysis is also important for unravelling disrupted gene expression in non-mutant cells; this disruption of gene expression might be cell-extrinsically mediated and of clinical importance (Giustacchini et al., 2017). In order to overcome this current limitation in single-cell genomic techniques, we set out to develop a method that combines full-length scRNA-seq or 3′-end-counting, high-throughput scRNA-seq with high-sensitivity mutation analysis.

DESIGN

The limitation of applying current scRNA-sequencing techniques to the detection of mutations in single cells partly relates to the fact that commonly used ''end-counting'' scRNA-seq techniques only detect the 3′ or 5′ region of transcripts (Hedlund and Deng, 2018). Consequently, most mutations within the body of a gene are not covered by sequencing reads. However, scRNA-seq techniques that amplify full-length transcripts, such as Smart-seq2 (Picelli et al., 2013), also have very poor sensitivity with regard to detecting the expression of most genes in most cells (Figure S1), and this difficulty precludes high-sensitivity mutational analysis. Furthermore, the vast majority of mutations identified in cancer are single-nucleotide variants (SNVs) and small indels (Vogelstein et al., 2013); these might be either heterozygous or associated with loss of heterozygosity (LOH) and have important functional consequences (Kharazi et al., 2011). Therefore, a key challenge in the field is to minimize allelic dropouts (ADOs) in order to ensure the detection of both alleles from a single cell.

It remains unclear whether the high ADO rates and lack of coverage across mutation hotspots in scRNA-seq data is primarily due to technical dropouts related to inefficient reverse transcription (RT) and/or PCR amplification or whether they are the result of true biological heterogeneity in the expression of mutant transcripts across single cells. We therefore first optimized the Smart-seq2 RT and PCR enzymatic conditions (SMART-seq+; Table S1A); this resulted in a significant reduction in dropout rates (Figure S1A), particularly for genes expressed at a low level (Figures S1B and C); a 25% increase in the number of genes detected per cell (Figure S1D); and a reduction in library bias (Figure S1E). However, despite improved sensitivity for the detection of gene expression with SMART-seq+, ADO rates remained exceedingly high for most genes (Figures S1F–H), a fact that currently precludes reliable mutational analysis using scRNA-seq (Povinelli et al., 2018). We therefore concluded that, because of the stochastic nature of gene expression in single cells, improving sensitivity for the analysis of coding DNA (cDNA) alone is unlikely to provide sufficient sensitivity for the detection of most cancer-associated mutations at the single-cell level.

Overcoming this problem requires the detection of mutations from genomic DNA (gDNA) in parallel with cDNA. Techniques for studying gDNA and mRNA from the same single cell have been previously described. However, these techniques either require both types of molecules to be physically separated (Han et al., 2018; Hou et al., 2016; Macaulay et al., 2015), which inevitably results in some loss of genetic material and consequently limits the techniques' sensitivity, or they rely on the parallel amplification of total gDNA and mRNA followed by the masking of coding regions (Dey et al., 2015). These technical constraints restrict the sensitivity of such techniques for the confident detection of specific point mutations. Whole-genome amplification also introduces significant expense to the method and has inherently high ADO and false-positive rates (Hosokawa et al., 2017; Wang et al., 2014). As a result, up to now, these techniques have not been widely used for parallel mutation or scRNA-seq analysis in cancer. Methods that combine targeted single-cell gene expression and mutation analysis have also been reported (Cheow et al., 2016; Wang et al., 2017), but these approaches have the limitation that only the expression of a limited number of pre-selected genes can be analyzed per cell.

Recently, we have described a method for the high-sensitivity detection of BCR-ABL1 (breakpoint cluster region and Abelson murine leukemia viral oncogene homolog 1 fusion protein) transcripts in parallel with scRNA-seq in chronic myeloid leukemia stem cells (Giustacchini et al., 2017). Although this study highlights the power of linking mutation and transcriptome information in single cells, the method is dependent on the expression of the targeted gene and/or allele in all mutated cells. This approach was effective in the specific case of the *BCR-ABL* fusion gene. However, for many autosomal genes, expression is undetectable or highly allelic-biased in the majority of transcriptionally active and highly proliferative K562 cells (Figure S1F) and also in quiescent $Lin^{-}CD34^{+}CD38^{-}$ primary human hematopoietic stem and progenitor cells (HSPCs; Figures S1G and H); this makes this method unsuitable to profile most mutations found in cancer. Moreover, this approach precludes analysis of non-coding mutations with key roles in tumorigenesis (Khurana et al., 2016). We therefore developed a method named TARGET-seq, which dramatically reduces ADO and also enables the efficient detection of non-coding mutations from the same single cell by allowing parallel, targeted mutation analysis of gDNA and cDNA alongside scRNA-seq.

RESULTS

TARGET-Seq Dramatically Increases the Sensitivity of Mutation Detection in Single Cells

In order to improve the detection of specific mRNA and gDNA amplicons, we extensively modified previously published template-switching protocols (Hedlund and Deng, 2018; Picelli et al., 2013; Zheng et al., 2018). To improve the release of gDNA, we modified the lysis procedure to include a mild protease digestion (Figure 1A and Table S1); we subsequently heat-inactivated the protease to avoid inhibition of the RT and PCR steps. Target-specific primers for cDNA and gDNA were added to the RT and PCR-amplification steps (Table S2), which also used modified enzymes (Table S1) that provided more efficient amplification (Figure 1A). We used an aliquot of the pre-amplified gDNA and cDNA libraries for targeted NGS of specific cDNA and gDNA amplicons and another aliquot for whole-transcriptome library preparation. The libraries used for targeted mutation analysis and those used for scRNA-seq were sequenced and analyzed independently.

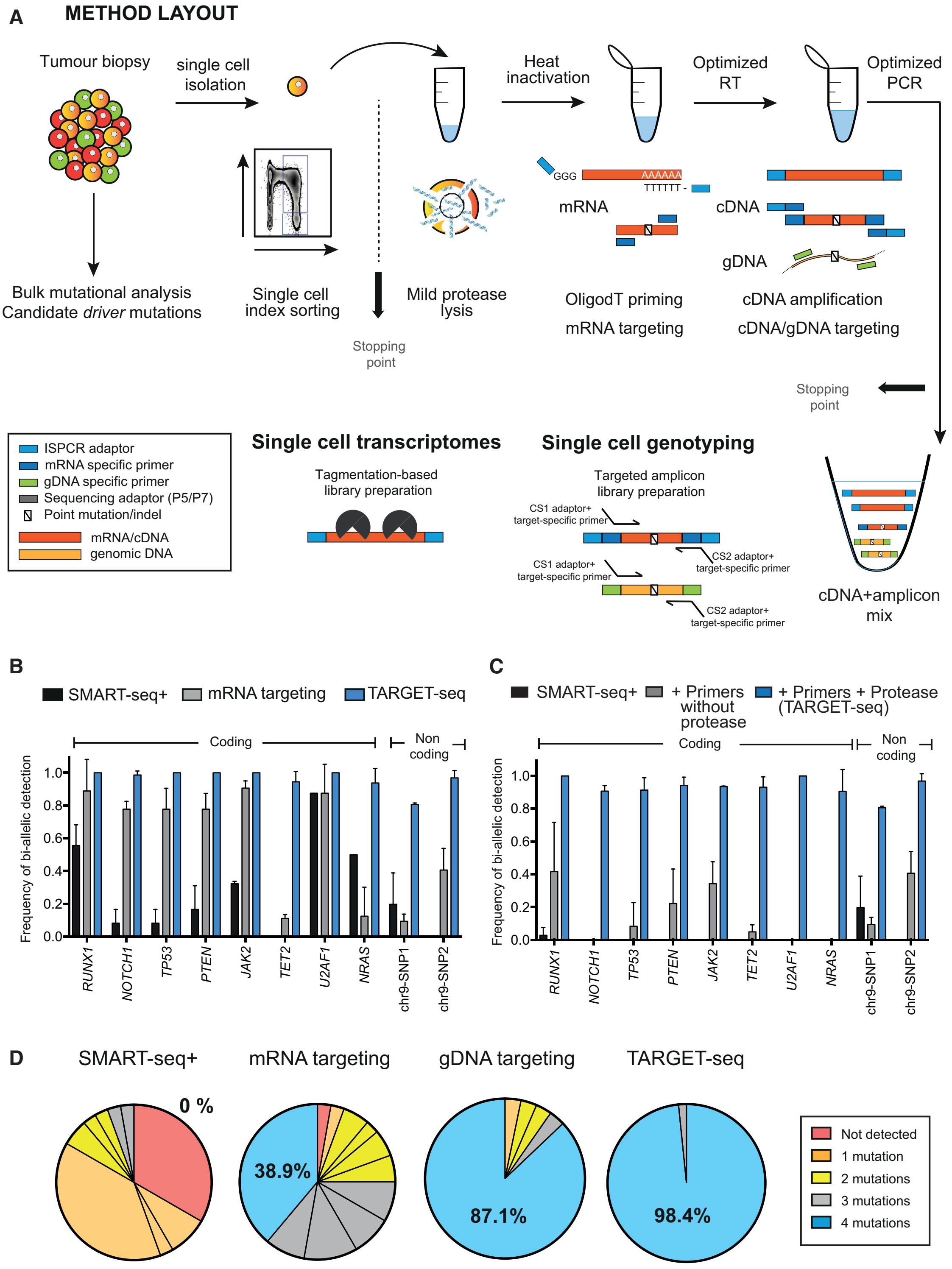

Figure 1. TARGET-Seq: A Method for High-Sensitivity Mutation Detection and Parallel Whole-Transcriptome Analysis from the Same Single Cell

(A) Schematic representation of the method (full details are available in STAR Methods and Supplemental Experimental Procedures). In brief, cells were sorted into plates containing TARGET-seq lysis buffer; after lysis, protease was heat inactivated. RT mix was then added. OligodT-ISPCR primed polyadenylated mRNA and target-specific primers primed mRNA molecules of interest. During subsequent PCR, we used ISPCR adaptors to amplify polyA-cDNA, and we used

(legend continued on next page)

In clonal cell lines, TARGET-seq dramatically improved the detection of ten mutation hotspots, including SNVs and small indels across both coding and non-coding regions (Figure 1B). Notably, gDNA amplicons alone achieved a mean 93% bi-allelic mutation and/or SNV detection (Figure 1C; the variant-calling pipeline and specific examples of variant calling can be found in Figures S2A and S2B, respectively). Importantly, mutational analysis from raw RNA-sequencing reads was impossible in almost all cells because of a lack of coverage (Figure S2C), despite the fact that the mean sequencing depth reached 2.93 million reads/cell.

We next tested whether TARGET-seq would improve the detection of combinations of mutations in single cells. We profiled four different mutations in a clonal T cell leukemia diploid cell line (JURKAT) carrying heterozygous mutations in *NOTCH1*, *RUNX1*, *TP53*, and *PTEN*. When we used SMART-seq+, detection of all of the four mutations within the same single cell was not achieved in any of the cells analyzed. mRNA targeting detected the four mutations in 38.9% of cells, gDNA targeting in 87.1% of cells, and TARGET-seq (combined mRNA+gDNA targeting) in 98.4% of cells (Figure 1D). Therefore, TARGET-seq provides extremely high sensitivity for the detection of multiple mutations in the same single cell, and this high sensitivity is essential for reliable reconstruction of tumor phylogenetic trees.

TARGET-Seq Produces Unbiased Transcriptomic Readouts from Single Cells

To determine whether TARGET-seq introduces a bias in the single-cell whole-transcriptome data, we evaluated its performance in two cell lines (JURKAT and SET2) and in primary human HSPCs. Cells clustered by cell type and not by method (Figures 2A and 2B), and there were no significant differences in the number of genes detected between methods (Figure 2C). The sequencing quality controls (QCs; Figure S3A), numbers of cells passing QC (Figure S3B), and transcript coverage (Figure S3C) were comparable between SMART-seq+ and TARGET-seq, and there were good correlations of gene expression, including for genes selected for targeted amplification (Figures 2D, S3D, and S3E). Similarly, ERCC spike-in controls revealed high correlations between methods (Figures 2E, S3F, and S3G), and cDNA traces were comparable (Figures S3H–J). These results demonstrate that TARGET-seq allows accurate mutation detection with parallel, unbiased, and full-length (Figure S3C) scRNA-seq of the same single cell.

The Stem Cell Compartment of Patients with MPN is Genetically and Transcriptionally Heterogeneous

We next applied TARGET-seq to analyze 458 HSPCs in samples from five patients with myeloproliferative neoplasms (MPN); the samples carried different combinations of *JAK2V617F*, *EZH2*, and *TET2* mutations (Tables 1 and S3). Two normal donors were also included as controls. We isolated $Lin^{-}CD34^{+}$ cells via fluorescence-activated cell sorting (FACS) (Figure S4) and indexed the cells for CD38, CD90, CD45RA, and CD123 to allow assessment of clonal involvement in different stem and progenitor cell compartments (Majeti et al., 2007). All mutations identified in total mononuclear cells were also detected in single cells within the $Lin^{-}CD34^{+}$ compartment with TARGET-seq (Table S3), revealing subclonal mutations with striking inter-patient heterogeneity. This allowed us to determine the mutation acquisition order (Table S3B), which is of importance for MPN biology (Ortmann et al., 2015). For example, in patient SMD32316 (a patient with essential thrombocythemia; Tables 1 andS3), we could determine that a *TET2* mutation was acquired after the *JAK2V617F* mutation, whereas in patient OX2123 (a patient with myelodysplastic syndrome [MDS]/MPN overlap; Tables 1 and S3), a *TET2* mutation was acquired before a *JAK2V617F* mutation. In two patients with a similar *JAK2V617F* variant allele frequency (VAF) in bulk mononuclear cells (MNCs), the low percentage of ADO that was achieved by TARGET-seq analysis of single cells revealed that *JAK2V617F* was heterozygous in most $Lin^{-}CD34^{+}CD38^{-}$ cells in patient IF0602 (a patient who had myelofibrosis [MF] and was receiving treatment with a JAK1/2 inhibitor; Table 1), and there was a normal distribution within the different $Lin^{-}CD34^{+}CD38^{-}$ stem and progenitor fractions (Figure 3A). In contrast, in patient IF0111 (a patient who had polycythemia vera and was receiving interferon; Table 1), a lower fraction of clonally involved $Lin^{-}CD34^{+}CD38^{-}$ cells were homozygous for *JAK2V617F* and predominantly had a $CD90^{+}CD45RA^{+}$ aberrant phenotype (Figure 3B) that has also been reported in other myeloid malignancies (Dimitriou et al., 2016). The ability to reliably distinguish heterozygous versus homozygous *JAK2V617F* mutations is of considerable importance for MPN biology (Li et al., 2014) and also, more broadly, in cancer because a mutant-allele-specific imbalance is common during disease progression (Soh et al., 2009).

TARGET-seq analysis uniquely allowed wild-type (WT) HSPCs to be reliably distinguished from *JAK2V617F* mutant cells in the same samples. The analysis revealed the aberrant expression of biologically relevant genes such as *LEPR* (Jiang et al., 2008) and oncogenes such as *MYCN*, *TP53*, or *PPP2R5A,* as well as biologically relevant pathways, including upregulation of hedgehog (Figure 3D) and Wnt β-catenin (Figure 3F) pathway-associated transcription (Table S4), in heterozygous (Figures 3C and 3D) and homozygous (Figures 3E and 3F) *JAK2V617F*-mutated HSPCs. HSPCs from patient IF0111 also showed dysregulation of interferon-associated gene expression, consistent with the

target-specific cDNA and gDNA primers to amplify amplicons of interest. An aliquot of the resulting cDNA+amplicon mix was used for preparing the genotyping library and another aliquot for preparing the transcriptome library for scRNA-seq.

(B) Frequency with which TARGET-seq detected heterozygous mutations in ten coding and non-coding regions in cell lines; this approach is compared to SMART-seq+ and mRNA targeting approaches (n = 376 cells, 2–3 independent experiments per amplicon; the bar graph represents mean ± SD).

(C) Frequency of detection of heterozygous mutations for the same amplicons as in (B), showing exclusively results from targeted genomic DNA sequencing. The bar graph represents mean ± SD.

(D) Frequency of detection of heterozygous mutations in JURKAT cells with SMART-seq+ (n = 36 cells), mRNA targeting (n = 36 cells), gDNA targeting (n = 62 cells), and TARGET-seq (n = 62 cells) when four different mutations (*RUNX1*, *NOTCH1*, *PTEN*, and *TP53*) in the same single cell were profiled in three independent experiments. Each slice of the pie chart represents a different combination of mutations, and each color represents the number of mutations detected per single cell.

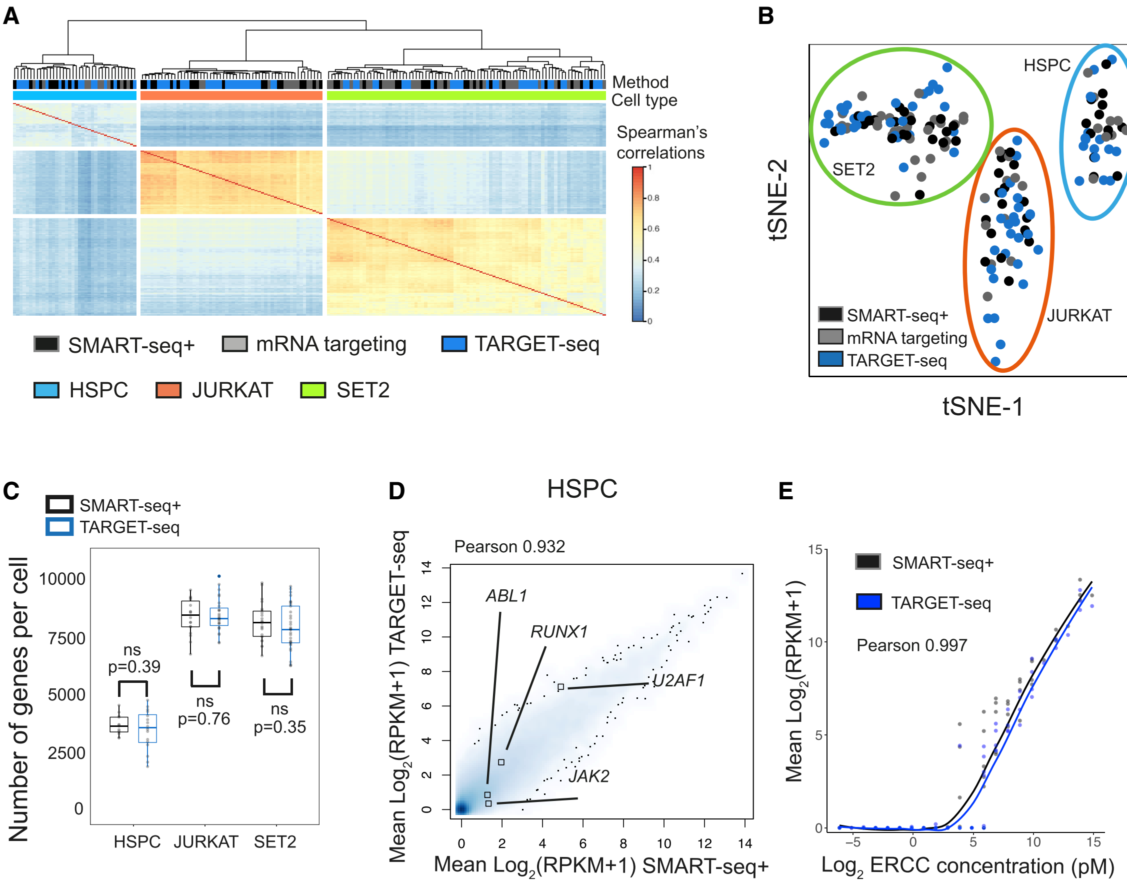

Figure 2. Unbiased Whole-Transcriptome Analysis of Single Cells with TARGET-Seq

(A) Unsupervised hierarchical clustering of Spearman's correlations from 180 single cells (JURKAT, n = 56; SET2, n = 86; and HSPC, n = 38); 4,088 highly variable genes were used. scRNA-seq libraries were generated with SMART-seq+, mRNA targeting, or TARGET-seq as indicated.

(B) tSNE representation of HSPCs, SET2 cells, and JURKAT cells from (A); the same 4,088 highly variable genes as in (A) were used.

(C) Number of detected genes per cell (RPKM $\geq$ 1) in HSPCs, SET2, and JURKAT cell lines from SMART-seq+ or TARGET-seq. "p" indicates the Student's-t-test p value, and "ns" = non-significance. The boxes represent median and quartiles, and the dots represent the value for each individual cell.

(D) Whole-transcriptome Pearson's correlation between SMART-seq+ and TARGET-seq ensembles (mean RPKM values per condition) in HSPCs. The expression values for the genes targeted are highlighted.

(E) Pearson's correlation between mean ERCC spike-in expression values from SMART-seq+ and TARGET-seq in HSPCs per ERCC spike-in concentration.

patient receiving treatment with interferon (Figure 3F and Table 1). The $CD90^+CD45RA^+$ aberrant phenotype was also present at a similar low frequency in an additional patient with a homozygous *JAK2* mutation (Figure 3G; patient OX4739, an MF patient receiving JAK1/2 inhibitor treatment). Cells from patient OX4739 also showed disrupted expression of a number of the same genes identified in patient IF0111 (Table S4E).

Importantly, this analysis allowed us to identify candidate biomarkers for *JAK2V617F* mutations in HSPCs from patients with an isolated *JAK2* mutation (Figure 3H; *RXFP1*, *GAS2*, and *WDR86*). Interestingly, *VWF*, a marker of platelet-biased stem cells (Sanjuan-Pla et al., 2013), was specifically upregulated in *JAK2V617F* mutant cells from patients IF0602 and OX4739, whose disease was characterized by abnormal megakaryocytic differentiation and MF, but it was not upregulated in *JAK2V617F* mutant cells from patient IF0111, who had a polycythemia phenotype (Figure 3I). These data support the notion that transcriptional lineage priming in the HSPC compartment might be linked to the disease phenotype in MPN.

Distinct Genetic Subclones Present Unique Transcriptional Signatures

TARGET-seq also uniquely allowed comparison of WT cells from patients' samples and normal controls. Intriguingly, this analysis established that WT HSPCs from patients with MPN were transcriptionally distinct from normal donor HSPCs (Figure 4A) and showed enrichment of inflammatory pathways associated with tumor necrosis factor α (TNFα) and interferon (IFN) signaling (Figures 3D, 3F, and 4B). These results might indicate the MPN microenvironment's effects on the wild-type cells from the same patient; a similar finding was demonstrated to have clinically predictive value in chronic myeloid leukemia (Giustacchini et al., 2017). Interestingly, WT HSPCs from patient IF0111, who was receiving interferon treatment, also showed strong IFN

Table 1. Summary of Donors in the Study, Mutation Status, and Clinical Characteristics

Sample Code	Mutation(s)	Donor Type	Diagnosis	Treatment	Figures
HD7643	–	normal donor	–	NA	Figures 3C–F, 3H, 3I, 4A–E, and S4A
HD7650	–	normal donor	–	NA	Figures 3C–F, 3H, 3I, and 4A–E
Aph1	–	normal donor	–	NA	Figures 5A–G and 5I–K
HD85	–	normal donor	–	NA	Figures 5A–G and 5I–K
SMD32316	JAK2 p.Val617Phe, TET2 p.Gln958Ter	patient	ET	aspirin	Figures 4A–C and 4E
IF0111	JAK2 p.Val617Phe	patient	PV	pegylated IFN alpha-2a	Figures 3B, 3E, 3F, 3H, 3I, 4A–C, and 4E
OX4739	JAK2 p.Val617Phe	patient	myelofibrosis (PMF)	ruxolitinib (JAK1 and JAK2 inhibitor)	Figures 3G–I, 4C, and 4E
OX2123	JAK2 p.Val617Phe, EZH2 p.Glu249AsnfsTer16, TET2 c.3409+1G>C	patient	MDS/MPN overlap with grade 3 bone marrow fibrosis	none	Figures 4C, 4D and S4B
IF0602	JAK2 p.Val617Phe	patient	myelofibrosis (PMF)	momelotinib (JAK1 and JAK2 inhibitor)	Figures 3A, 3C, 3D, 3H, 3I, 4A–C, and 4E (full length TARGET-seq); and Figures 5A–K (3'-TARGET-seq)
IF0155	JAK2 p.Val617Phe	patient	myelofibrosis (post-ET)	anagrelide	Figures 5A–K
IF0157	JAK2 p.Val617Phe	patient	myelofibrosis (post-PV)	ruxolitinib 10 mg BD (JAK1 and JAK2 inhibitor)	Figures 5A–K
IF0140	JAK2 p.Val617Phe, TET2 p.Ser1612LeufsTer4	patient	myelofibrosis (post-PV)	ruxolitinib 20 mg BD (JAK1 and JAK2 inhibitor)	Figures 5A–C
IF0101	JAK2 p.Val617Phe, CBL p.Cys404Tyr, SRSF2 p.Pro95His	patient	myelofibrosis (PMF)	ruxolitinib 10 mg BD (JAK1 and JAK2 inhibitor)	Figures 5A–C, 6E, 6F, S6, S7C, S7F, S7I, S7L, and S7O
IF0123	JAK2 p.Val617Phe, SF3B1p.Lys666Asn	patient	myelofibrosis (PMF)	ruxolitinib 5 mg BD (JAK1 and JAK2 inhibitor)	Figures 5A–G and S6
IF0138	JAK2 p.Val617Phe, ASXL1 p.Gly646TrpfsTer12, ASXL1 p.Gly644TrpfsTer12	patient	myelofibrosis (post-PV)	hydroxycarbamide	Figures 5A–K, 6C, 6D, S6, S7B, S7E, S7H, S7K, and S7N
IF0137	JAK2 p.Val617Phe, U2AF1 p.Gln157Arg, TET2 p.Ile1105MetfsTer8, ASXL1 p.Gln910AlafsTer13, ASXL1 p.Trp898ArgfsTer5	patient	myelofibrosis (PMF)	none	Figures 5A–G, 6A, 6B, S6, S7A, S7D, S7G, S7J, and S7M

Additional clinical details are shown in Table S3. PMF, primary myelofibrosis; MDS, myelodysplastic syndrome; MPN, myeloproliferative neoplasm; ET, essential thrombocythemia; PV, polycythemia vera.

signaling signatures, thus providing an additional layer of validation for the transcriptional signatures obtained (Figures 3F and 4B).

Using the top 2,000 genes identified by random forest analysis (Figure 4C), we analyzed combinations of mutations and showed striking clustering of HSPCs of the same genotype from multiple different patients. HSPCs carrying mutations in epigenetic modifiers had a highly distinct transcriptomic signature, whereas the signature of cells carrying only heterozygous *JAK2V617F* mutations more closely resembled the transcriptome of WT cells (Figure 4C). *EZH2* mutant cells showed enrichment in pathways such as apoptosis, P53 signaling, hypoxia, and the cell cycle (Figure 4D and Table S4F) previously identified to be correlated with loss of PRC2 function (Xie et al., 2014) and negative enrichment in genes downregulated upon *EZH2* knockdown (Table S4F). *TET2* mutant cells also showed enrichment in HSC-related genes and a negative enrichment in genes downregulated upon *TET2* knockout (Zhang et al., 2016) (Figure 4D and Table S4F). Moreover, *JAK2V617F* cells showed dysregulation of *STAT5A* targets (Figure 4E and Table S4G). Taken together, these data demonstrate that TARGET-seq reveals distinct and biologically relevant molecular signatures of HSPC subclones in MPN and represents a powerful tool for biomarker and therapeutic target discovery.

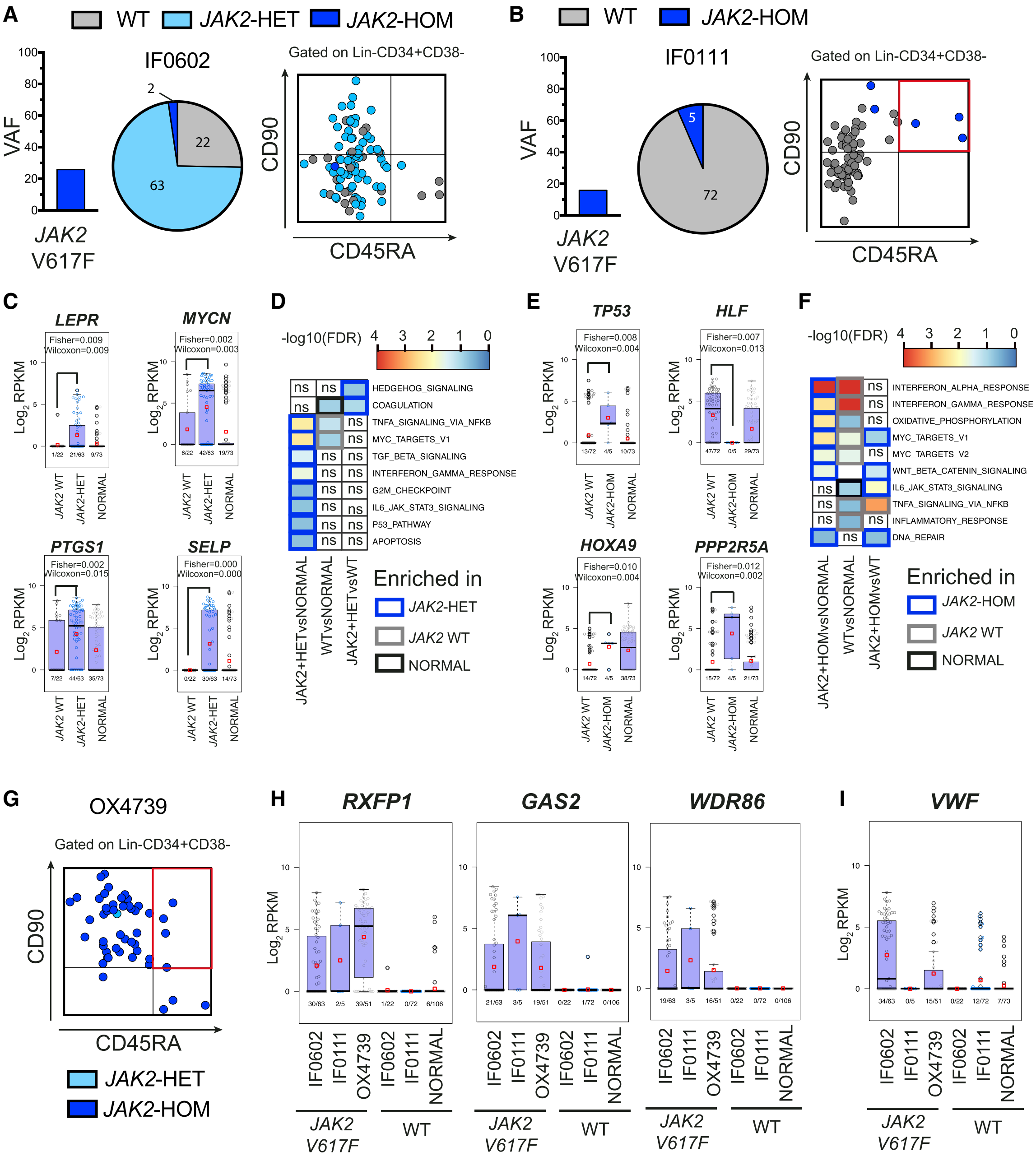

Figure 3. TARGET-Seq Reveals Genetic and Transcriptional Heterogeneity in the Stem-Cell Compartment of Patients with MPN

(A and B) Variant allele frequency of *JAK2V617F* mutation (left), as identified by bulk sequencing of total MNCs; proportion of single cells that carry the mutation (including zygosity) in the Lin-CD34+CD38- compartment (center); and integration of index sorting with mutational information (right) for patients IF0602 (A) and IF0111 (B).

(C–F) Analysis of disrupted gene expression associated with *JAK2V617F* mutation in HSPCs. Beeswarm plots show selected differentially expressed genes between (C) *JAK2* wild-type (WT) and *JAK2V617F*-heterozygous mutant cells from patient IF0602 or (E) *JAK2* WT and *JAK2V617F*-homozygous mutant cells from patient IF0111. Expression values for single cells from two normal donors (NORMAL) are also shown. Each dot represents the expression value for each single cell; red squares represent mean expression values for each group, and boxes represent median and quartiles. Fisher's test and Wilcoxon test p values are shown on the top of each graph; expressing cell frequencies are shown on the bottom of each bar for each group. Table S4A (patient IF0602) and Table S4C (patient IF0111) show all significant, differentially expressed genes. (D) GSEA analysis of *JAK2* WT and *JAK2V617F*-heterozygous mutant cells from patient IF0602 or (F) *JAK2* WT and *JAK2V617F*-homozygous mutant cells from patient IF0111, as well as cells from normal donors (NORMAL). The heatmap represents –log10(FDR q-values) for each comparison, for which a FDR q-value cut-off < 0.25 was used; a white color with "ns" represents non-significance. The borders of each square of the heatmap are colored according to the group in which a particular pathway is enriched. Table S4B (patient IF0602) and Table S4D (patient IF0111) show results for all significant genesets tested.

(G) Integration of index sorting with mutational information for patient OX4739.

(legend continued on next page)

High-Throughput 3′-TARGET-Seq Resolves Complex Clonal Hierarchies in *JAK2* Mutant Myelofibrosis

To increase the throughput of the technique, we adapted TARGET-seq to allow barcoding and pooling of scRNA-seq libraries in a 384-well format in reduced reaction volumes, generating 3′-biased libraries (Table S1C and Figure S5A). Barcodes could be reliably detected (Figure S5B), sequencing quality metrics were in line with other 3′-biased scRNA-seq methods (Paul et al., 2015; Velten et al., 2017) (Figure S5C), and transcript coverage was 3′ biased (Figure S5D). We then analyzed 2,798 cells from a cohort of eight patients with MF and two age-matched normal donors (Tables 1 and S3). TARGET-seq genotyping provided very low dropout rates, in stark contrast to cDNA genotyping alone (Figures S6A and S6B). This allowed reconstruction of clonal hierarchies in these patients at unprecedented scale and resolution (Figures S6B and S6C and Table S3). Considerable inter-patient heterogeneity was observed, and there were both linear and branching patterns of clonal evolution (Figure S6C). Spliceosome mutations were an early event in these patients; in contrast, *ASXL1* mutations were acquired late, and there were also multiple *ASXL1* mutations acquired independently in patient IF0137 (Figures S6B and S6C and Table S3).

T-SNE analysis using 3,286 highly variable genes showed distinct clusters of MF HSPCs according to their genotype (Figure 5A). HSPCs carrying mutations in spliceosome components or epigenetic modifiers in addition to *JAK2* clustered separately from WT HSPCs, including WT cells from the same patients, and were also distinct from cells carrying a *JAK2* mutation alone. TARGET-seq allowed the identification of specific gene expression associated with certain genetic subclones of HSPCs. For example, cells carrying mutations exclusively in *JAK2* specifically upregulated *B4GALT1* (Figure 5B), which is associated with acquisition of drug resistance in leukemia (Zhou et al., 2013), and cells with mutations in epigenetic modifiers specifically upregulated *PITX1*, which has been previously implicated in leukemogenesis (Nagel et al., 2011). *ZFP36* (also known as *TTP*), which modulates the interferon-induced inflammatory response (Sauer et al., 2006), was upregulated in cells carrying mutations in spliceosome components. Cells carrying mutations in spliceosome and epigenetic genes upregulated *PHB*, a proposed therapeutic target in leukemia (Pomares et al., 2016). MF HSPCs also showed more transcriptional diversity, including within genetically defined subclones, than WT counterparts (Figure 5C), suggesting that this transcriptional heterogeneity is not driven by genetic heterogeneity alone (Figure 5C). Normal donor HSPCs also clustered separately from WT HSPCs from MF patients (Figure 5D), an observation similar to that made by full-length TARGET-seq. Differences between normal donor and MF WT HSPCs included dysregulation of specific genes and gene signatures associated with inflammation, as well as TNFα and TGFβ signaling (Figures 5E and 5F and Table S5). Furthermore, a number of oncogenes and tumor suppressors were aberrantly expressed in WT HSPCs from MF patients (Figure 5G), raising the possibility that these cells might be more susceptible to malignant transformation and the development of secondary hematopoietic malignancy.

Specific analysis that compared only *JAK2* mutant and WT cells and used the top 2,000 genes identified by random forest analysis showed specific clustering of WT, *JAK2V617F*-heterozygous, and *JAK2V617F*-homozygous cells (Figure 5H). *JAK2*V617F-heterozygous cells showed enrichment in inflammation-related signatures such as TNFα, TGF β, and IFN signaling; the G2M checkpoint; and the P53 pathway (Figure 5I), further validating the pathways previously identified by full-length TARGET-seq in specific patients (Figure 3). *JAK2*V617F-homozygous mutant cells showed enrichment in WNT β-catenin, hedgehog signaling, and apoptosis, as well as in inflammation-related signatures (Figure 5I). The distinct clustering we observed was driven by a number of the same genes identified by full-length TARGET-seq, e.g., *GAS2* and *RXFP1* (Figure 5J and Table S5); we also identified a number of additional genes (*STAT1*, *CD69*, and *NFKBIZ* [Figure 5J and Table S5]), some of which were specifically upregulated in *JAK2*-homozygous but not *JAK2*-heterozygous mutant cells (*IL8* and *CLEC7A* [Figure 5K]).

Transcriptional Differences between Genetic Subclones within Individual Patients Are Identified with TARGET-Seq

Finally, we explored whether distinct genetic subclones of HSPCs in individual patients could be identified with TARGET-seq. We analyzed three patients with complex clonal hierarchies (at least three genetic subclones [Figure S6]): patients IF0137 (Figures 6A and 6B), IF0138 (Figures 6C and 6D), and IF0101 (Figures 6E and 6F). Each genetic subclone clustered separately (Figures 6A, 6C, and 6E) and showed transcriptional differences driven by pro-apoptotic genes (*MCL1* [Figure 6B and Table S6]), JAK2-STAT signaling (*STAT2* [Figure 6D and Table S6]), chemokines (*CXCL2* [Figure 6D and Table S6), and genes previously implicated in leukemogenesis (*PHB*, *BCL11A*, and *STAG2* [Figures 6B and 6F and Table S6) or drug resistance (*GSTK1* [Figure 6F and Table S6).

We then explored whether the same genetic subclones could have been identified by common dimensionality reduction or clustering methods. Dimensionality reduction using highly variable genes (Figures S7A–C) did not identify distinct clustering patterns associated with genetic subclones in patients IF0137, IF0138, or IF0101 either when we regressed out the effect of the cell-cycle phase (Figures S7D–F) or when we specifically modeled zero inflation (Figures S7G–I) (Pierson and Yau, 2015). Furthermore, genetic subclones could not be identified with a

(H) Beeswarm plots of selected genes identified as biomarkers of *JAK2* mutant cells independently of the patient analyzed. Expression values across HSPCs from patients IF0602, IF0111, OX4739 (*JAK2* WT and *JAK2V617F* mutant cells shown separately), and two normal donors (NORMAL) are shown; expression frequencies are provided at the bottom of each graph for each group.

(I) A Beeswarm plot of *VWF* expression values across HSPCs for the same patients and normal donors as in (H). Each dot represents the expression value for each single cell; red squares represent mean expression values for each group, and boxes represent the median and quartiles. Fisher's test and Wilcoxon test p values are shown on the top of each graph; expressing cell frequencies are shown on the bottom of each bar for each group.

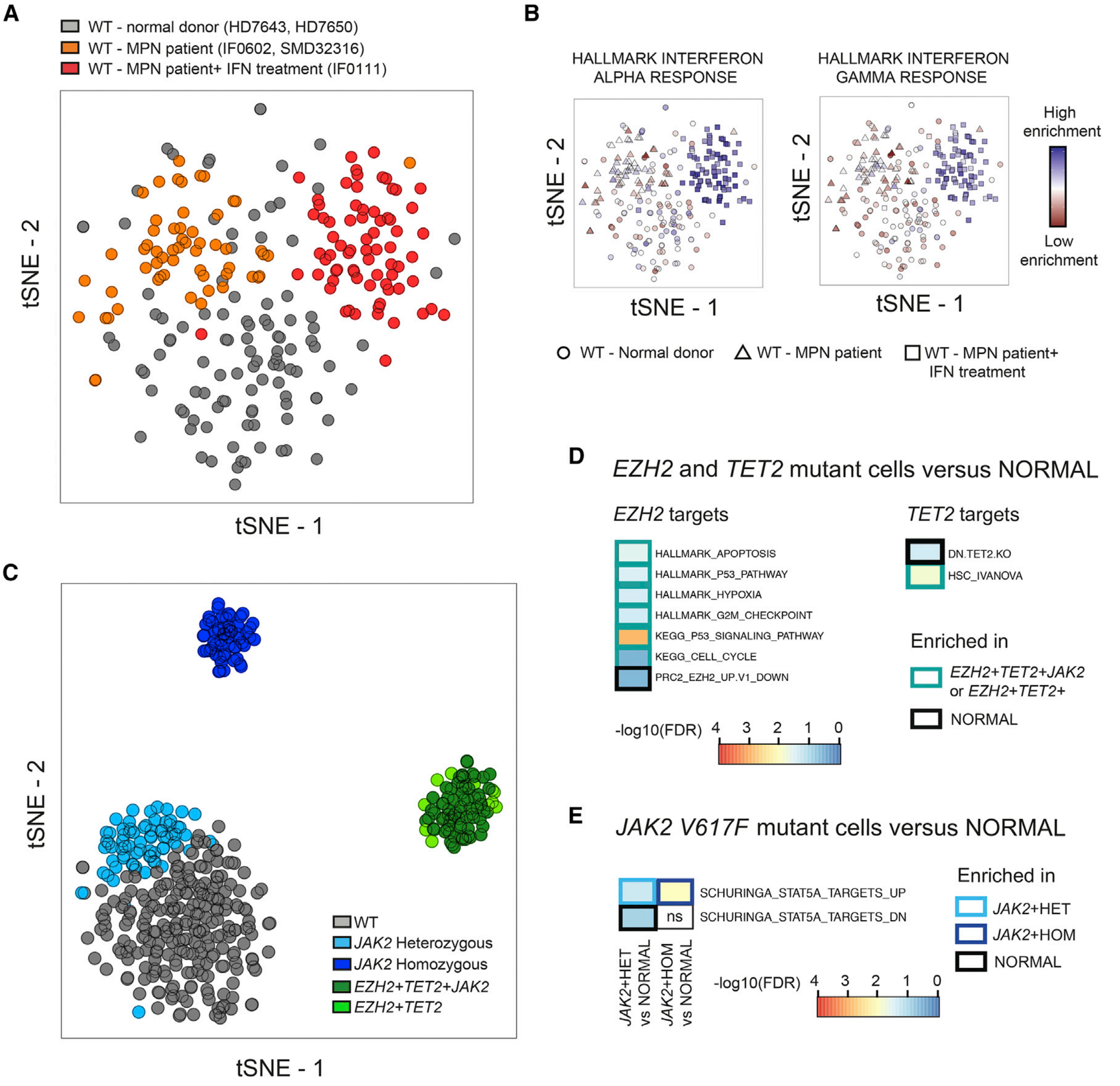

Figure 4. TARGET-Seq Reveals Distinct Transcriptional Signatures Associated with the Presence or Absence of Somatic Mutations in Single HSPCs

(A) tSNE representation of 236 wild-type (WT) HSPCs from the three samples (from patients IF0602, SMD32316, and IF0111) in which WT cells are present, and cells from two normal donors (donors HD7650 and HD7643); 5,365 highly variable genes were used. Cells from normal donors are colored in gray, and cells from patients with MPN are colored in orange (patients SMD32316 and IF0602) or red (patient IF0111; patient treated with interferon).

(B) Enrichment of IFN-α (left) or IFN-γ (right) signaling gene signatures as a projection of ssGSEA results at the same tSNE coordinates from the cells of the specific donors or patients shown in (A). Each shape represents a group of donors.

(C) tSNE representation of 448 HSPCs from five patients and two normal controls; the top 2,000 genes as measured by the Gini index from the random forest analysis were used. Only genotypes present in at least five cells were analyzed. The gene expression matrix was batch- and donor-corrected, and genotypes were preserved.

(D and E) Enrichment of *EZH2*-related pathways, *TET2*-related pathways (D), or the JAK/STAT pathway (E) in cells carrying mutations in these genes compared to (n = 106) cells from two normal donors. The heatmap represents –log10(FDR q-values) for each comparison, using a FDR q-value cut-off < 0.25. A complete list of all significant genesets tested can be found in Tables S4F and S4G, and a summary list of all genesets can be found in Table S4H.

recently published single-cell K-means clustering method (SC3) (Kiselev et al., 2017) previously reported to specifically distinguish genetically distinct subclones of cells (Figures S7J–L); they also could not be identified with the KNN-based clustering implemented in the PAGODA2 package (Figures S7M–O). Distinct genetic subclones from the same patient were, however, robustly identified by dimensionality reduction when we used genes that were differentially expressed between different genetic subclones, the identification of which was made possible by TARGET-seq (Figure 6).

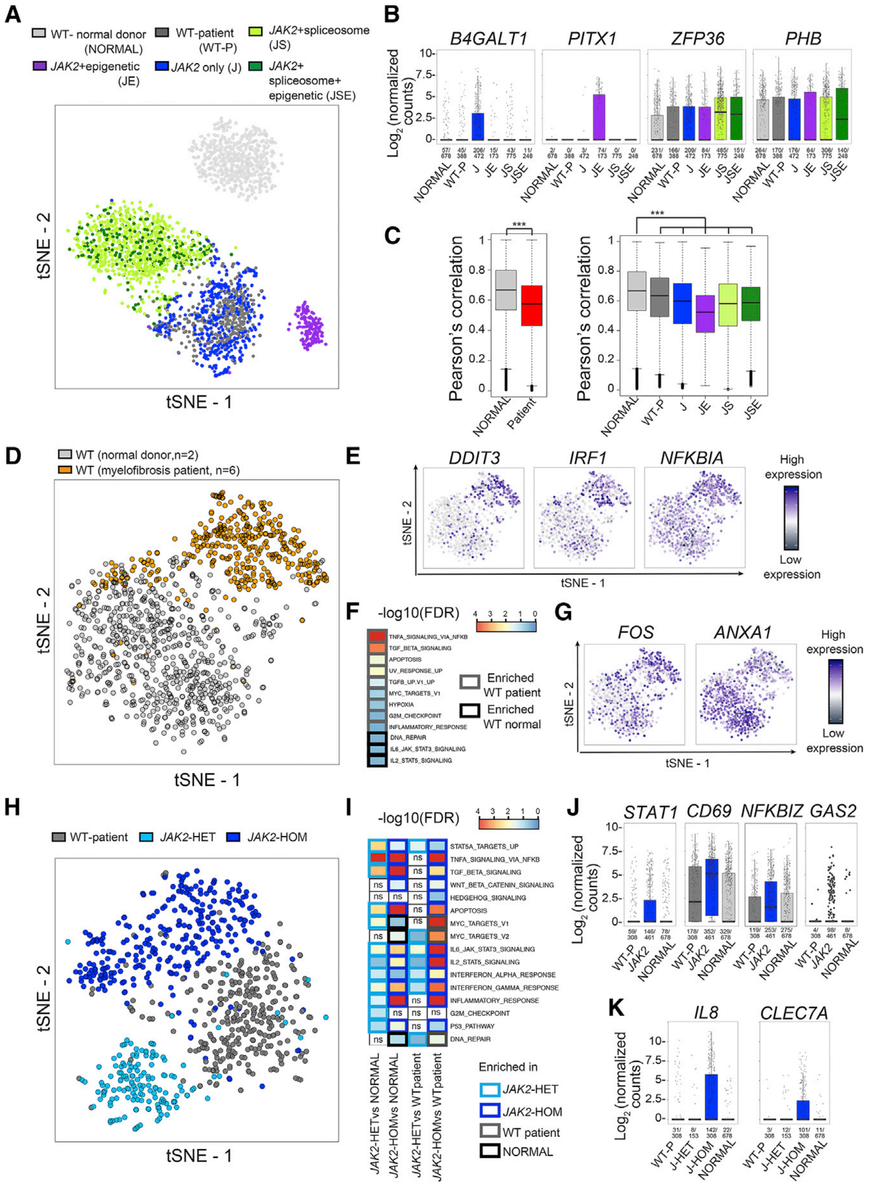

Figure 5. High-Throughput TARGET-Seq Identifies Molecular Signatures of Genetic Subclones in HSPCs from *JAK2-V617F* Mutant Myelofibrosis

(A) tSNE representation of 2,734 HSPCs from eight patients and two age-matched normal donors; the samples were processed with 3′-TARGET-seq, and 3,286 highly variable genes were used for the analysis. Cells from age-matched normal donors are colored in light gray (NORMAL). Wild-type (WT) cells from patients with MF are colored in dark gray (“WT-P”). Cells carrying mutations exclusively in *JAK2* are colored in blue (“J”); those carrying mutations in *JAK2* and epigenetic modifiers (*TET2* and *ASXL1*) are colored in purple (“JE”); those carrying mutations in *JAK2* and spliceosome components (*SF3B1*, *SRSF2*, and *U2AF1*) are colored in light green (“JS”); and those carrying mutations in *JAK2*, spliceosome components, and epigenetic modifiers are colored in dark green (“JSE”). The gene expression matrix was batch- and donor-corrected, and genotypes were preserved.

(B) Boxplots of representative differentially expressed genes from *JAK2* only (*B4GALT1*), *JAK2*+epigenetic (*PITX1*), *JAK2*+spliceosome (*ZFP36*), or *JAK2*+spliceosome+epigenetic (*PHB* and *ZFP36*) genetic subclones. Each dot represents the expression value for each single cell; boxes represent median and quartiles, and the central line represents the median for each group. Expression frequencies are shown on the bottom of each bar for each group.

(C) Boxplot of overall Pearson’s correlation of cells from normal donors and cells from MF-patient samples; the cells are grouped per donor type (normal donor or patient sample; left panel) or by the genotype groups presented in (A) (right panel). A Kolmogorov-Smirnov test provided the significance level for each comparison (***; p value < 0.001).

(D) tSNE representation of 1,066 WT cells from six patients and two normal donors; 3,436 highly variable genes were used. The gene expression matrix was batch-corrected, and the donor effect was preserved.

(E) tSNE projection (from the same cells as in [D]) representing relative gene expression levels from selected differentially expressed inflammation-associated genes in WT cells from patients and normal donors.

(F) Enrichment of selected pathways in the same WT cells from the same samples as in (D) and (E) from normal donors and patients. A complete list of all significant genesets tested can be found in Table S5A.

(G) tSNE projection representing relative gene expression levels from selected differentially expressed oncogenes (*FOS*) and tumor suppressors (*ANXA1*) between the same WT cells from patients and normal donors as in (D).

(H) tSNE representation of 769 WT and *JAK2*-only mutant HSPCs from four patients with MF (patients IF0138, IF0155, IF0157, and IF0602); we used the top 2,000 genes as identified by the Gini index from random forest analysis.

(I) Enrichment of selected HALLMARK and STAT5A pathways from the same cells as in (H), as well as cells from normal donors (NORMAL). A complete list of all significant genesets tested can be found in Tables S5B and S5C, and specific comparisons for subclones within patients can be found in Table S5D.

(J and K) Analysis of disrupted gene expression associated with *JAK2V617F* mutation in HSPCs. Boxplots show selected differentially expressed genes specifically upregulated in *JAK2* mutant cells independently of zygosity (J) or exclusively in *JAK2*-homozygous cells (K). Each dot represents the expression value for each single cell; boxes represent median and quartiles, and the central line represents the median for each group. Expressing-cell frequencies are shown on the bottom of each bar for each group. A complete list of all significant differentially expressed genes and associated p values can be found in Table S5E. The heatmaps are colored according to –log10(FDR q-values) for each comparison, for which an FDR q-value cut-off < 0.25 was used. The borders of each square of the heatmap are colored according to the group in which a particular pathway is enriched; a white color with “ns” represents non-significance.

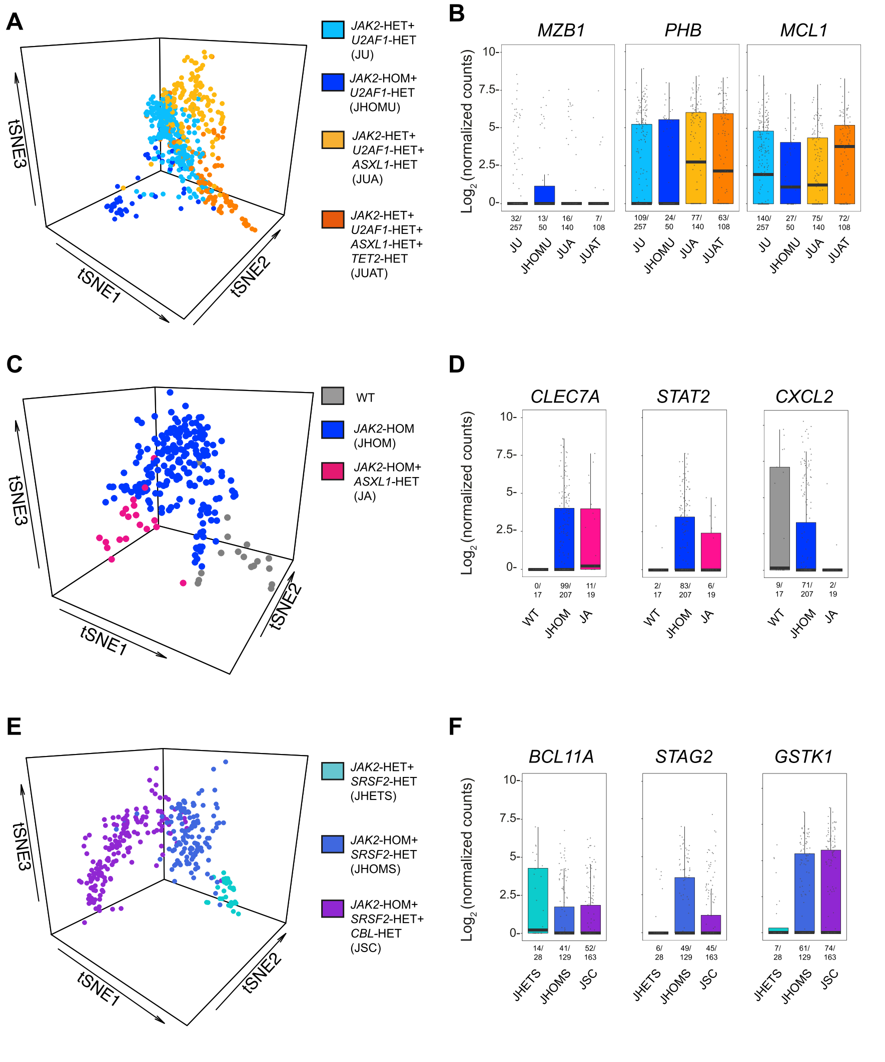

Figure 6. TARGET-Seq Resolves Genetic and Transcriptional Heterogeneity of HSPCs within Individual Myelofibrosis Patients

(A and B) Distinct transcriptional signatures of genetic subclones identified by TARGET-seq in patient IF0137. (A) tSNE representation of 555 cells; 633 differentially expressed genes identified with ANOVA were used and (B) boxplots of selected differentially expressed genes between each genetic subclone from the same cells as in (A). Genetic subclones carrying *JAK2*, *U2AF1*, and *ASXL1* (p897/p910) mutations from patient IF0137 are labeled JAK2-*HET*+U2AF1-*HET*+ASXL1-*HET* and were analyzed together as indicated. Each genetic subclone is colored and labeled according to the legend provided in (A).

(C and D) Distinct transcriptional signatures of genetic subclones from patient IF0138. (C) tSNE representation of 243 cells; 418 differentially expressed genes identified with ANOVA were used. (D) Boxplots of selected differentially expressed genes between distinct genetic subclones. Each genetic subclone is colored according to the legend provided in (C).

(E and F) Distinct transcriptional signatures of genetic subclones from patient IF0101. (E) tSNE representation of 320 cells; 500 differentially expressed genes identified with ANOVA were used. (F) Boxplots of selected differentially expressed genes between distinct genetic subclones. Each genetic subclone is colored according to the legend provided in (E). Each dot represents the expression value for each single cell; boxes represent median and quartiles, and the central line represents the median for each group. Expressing cell frequencies are shown on the bottom of each bar for each group. The list of differentially expressed genes identified in each patient and associated p values for each comparison can be found in Table S6. Only genetic subclones representing at least 5% of the total cells for each patient are included in the analysis.

DISCUSSION

With the advent of molecularly targeted therapy in cancer (Longo, 2017), clinical remissions and clonal responses can be readily achieved in many patients. However, relapse frequently occurs, and it is often associated with evidence of clonal evolution, most likely reflecting ITH already present at diagnosis (Smith et al., 2017) and a differential response to the targeted therapy in distinct tumor subclones. Therefore, it is crucial to resolve the clonal heterogeneity of tumors and dissect the transcriptional heterogeneity associated with the responsive and resistant subclones of cancer cells. Although scRNA-seq offers great potential to resolve the transcriptomic signatures of tumor subclones, up to now it has not been possible to correlate scRNA-seq data with mutation analysis because of the lack of coverage for small indels or point mutations in the scRNA-seq reads, although large chromosomal aberrations can be detected (Tirosh et al., 2016a). For example, in a recent study of gliomas, from 22 mutations analyzed, reads spanning the position of the mutations were detected in 0.4% to 8.7% of the cells (Tirosh et al., 2016b). Although methods for the parallel sequencing of the whole-transcriptome and whole-genome of single cells have previously been reported, these methods are not well suited for high-sensitivity mutation detection because of high ADO rates (Dey et al., 2015; Macaulay et al., 2015). Furthermore, these approaches are relatively costly because of the requirement for whole-genome amplification. Consequently, up to now, such techniques have not been widely used for the analysis of cancerous tissues.

We herein report a single-cell RNA sequencing and genotyping method that provides a simple, easily implementable, and customizable protocol for high-sensitivity mutation detection with parallel, unbiased whole-transcriptome analysis. TARGET-seq has clear advantages above other available scRNA-seq methodologies and provides improved complexity of scRNA-seq libraries and a dramatically improved ability to detect multiple mutations in the same single cell, primarily attributable to the detection of gDNA variants through modified cell lysis and high-sensitivity, targeted amplification. The high sensitivity for bi-allelic detection of mutations provided by our technique is also of considerable importance as loss of heterozygosity of

a number of different mutations is an important driver of disease phenotype as well as therapy response (Kharazi et al., 2011). This is also demonstrated in our analysis of patients with MPN; this analysis shows clear transcriptional differences between *JAK2*-heterozygous and homozygous HSPCs in multiple patients. TARGET-seq also allowed analysis of the order of acquisition of mutations, which is of importance in cancer biology (Ortmann et al., 2015). Moreover, TARGET-seq has the advantage of combining scRNA-seq data and mutational analysis with index sorting, allowing cells to be traced back to canonical stem and progenitor cell hierarchies. This revealed an aberrant HSPC phenotype associated with the presence of a *JAK2*-homozygous mutation in patients with MPN. Furthermore, the reliable identification of WT cells by TARGET-seq allows analysis of aberrant gene expression in normal tissue-residing cells; such aberrant expression might reflect cell-extrinsic phenomena. Such microenvironmental factors might underlie many aspects of tumor biology and therapy response.

TARGET-seq is adapted to allow both full-length and 3′-biased scRNA-seq approaches. The throughput of the full-length technique would typically enable the preparation of approximately 400 cells per week and thousands of cells within a few months; this amount is in line with the numbers of cells analyzed in published scRNA-seq tumor datasets (Giustacchini et al., 2017; Tirosh et al., 2016a, 2016b). This version of the protocol generates scRNA-seq libraries of high complexity and sensitivity for detecting low-level expressed genes. Moreover, it allows analysis of alternative splicing patterns; this is of importance in cancer biology (David and Manley, 2010), as well as in many other diseases (Cooper et al., 2009), particularly because components of the spliceosome machinery are recurrently mutated in cancer (Kandoth et al., 2013).

Higher-throughput scRNA-seq techniques are available (Macosko et al., 2015; Zheng et al., 2017); these typically provide shallow coverage of only the 3′ or 5′ region of transcripts and lower molecular capture rates but enable the analysis of larger numbers of cells. Therefore, we also developed 3′-biased TARGET-seq to allow higher-throughput analysis. 3′-TARGET-seq is associated with shallower coverage than full-length TARGET-seq, reducing sequencing costs, but it retains high-sensitivity mutation analysis at the single-cell level. 3′-TARGET-seq is mostly automated, and the process would typically allow 1,000 cells to be processed per week and tens of thousands to be processed within a few months, considerably increasing the throughput of the technique. In a cohort of patients with MF, this approach revealed complex clonal hierarchies and marked inter-patient variability that was not apparent from bulk genetic analysis. This allowed distinct transcriptional signatures of specific genetic subclones and non-clonally involved WT HSPCs to be characterized, which was not possible with other computational approaches.

In summary, TARGET-seq is a powerful tool for resolving both genetic and transcriptional intratumoral heterogeneity. TARGET-seq also uniquely allows the identification of specific molecular signatures within genetically distinct subclones of tumor cells. We expect that this will pave the way for the application of scRNA sequencing for the definitive analysis of intratumoral heterogeneity and the identification and characterization of therapy-resistant tumor subclones.

Limitations

A potential limitation of TARGET-seq is that this approach does not support mutation discovery and relies on the analysis of known driver mutations or mutations previously identified by other discovery-type methods. However, because the lysate is initially frozen and stored, this will routinely allow for mutational analysis of the same sample before the subsequent processing of single cells. Up to now, we have multiplexed primers to detect a total of 12 different mutations per single cell. Although this will be adequate for analyzing key driver mutations in many tumors, for more genetically complex malignancies, a more complex multiplexing strategy might be required. For very genetically complex tumors where potentially hundreds of different mutations need to be tracked, a whole-genome and whole-transcriptome approach might be more appropriate (Dey et al., 2015; Macaulay et al., 2015), albeit at the cost of reduced sensitivity for the detection of those mutations (Hosokawa et al., 2017; Wang et al., 2014). In the current study, we have applied this technique to analyze hematopoietic tumors; however, this method could be broadly applied to the analysis of a range of cancers and is a powerful tool for linking transcriptional signatures with genetic tumor heterogeneity.

STAR★METHODS

Detailed methods are provided in the online version of this paper and include the following:

- KEY RESOURCES TABLE
- CONTACT FOR REAGENT AND RESOURCE SHARING
- METHOD DETAILS
  - Cell Lines
  - Banking and Processing of Human Samples
  - Bulk Sequencing of Mononuclear Cells
  - Fluorescent Activated Cell Sorting (FACS) Staining and Single-Cell Isolation
  - cDNA Synthesis (RT-PCR)
  - Targeted NGS Single-Cell Genotyping
  - Nextera XT Library Preparation for Full-Length Whole-Transcriptome Sequencing
  - Nextera XT Library Preparation for 3′-Biased Whole-Transcriptome Sequencing
  - Single Cell Full-Length RNA-Sequencing Data Pre-Processing
  - Single Cell 3′-Biased RNA-Sequencing Data Pre-Processing
  - Whole-Transcriptome Variant Calling from Single Cells
  - Mutational Analysis from RNA-Sequencing Reads
  - Dropout Frequency and Library Bias Calculation
  - Transcript Coverage
  - Differential Expression Analysis
  - Identification of Highly Variable Genes
  - Single Cell Clustering and Dimensionality Reduction
  - Cell to Cell Correlation Measurements
  - Batch Correction
  - Cell Cycle Phase Assignment and Correction
  - Random Forest Analysis
  - GeneSet Enrichment Analysis

- QUANTIFICATION AND STATISTICAL ANALYSIS
  - Computational Reconstruction of Clonal Hierarchies
  - Code Availability
- DATA AND SOFTWARE AVAILABILITY
- ADDITIONAL RESOURCES

SUPPLEMENTAL INFORMATION

Supplemental information includes seven figures, Supplemental Material and Methods, and six tables and can be found with this article online at https://doi.org/10.1016/j.molcel.2019.01.009.

ACKNOWLEDGMENTS

We thank all the patients who kindly donated samples and the staff at the National Cancer Research Network (NCRN); Dr. Deena Iskander and the MDSBio study for samples; and Dr. Nguyen Tran for laboratory management. This work was funded by a Medical Research Council (MRC) Senior Clinical Fellowship (MR/L006340/1) to A.J.M., a Cancer Research UK (CRUK) DPhil Prize Studentship (C5255/A20936) to A.R-M., and the MRC Molecular Haematology Unit (MHU) core award to A.J.M. and S.E.W.J. (MC_UU_12009/5). The authors acknowledge the contributions of Dr. Neil Ashley at the MRC Weatherall Institute of Molecular Medicine (WIMM) Single Cell Facility and MRC-funded Oxford Consortium for Single-Cell Biology (MR/M00919X/1). They also acknowledge the National Institute for Health Research (NIHR) Oxford Biomedical Research Centre (BRC); the WIMM Flow Cytometry Facility, supported by the MRC Human Immunology Unit (HIU); the MRC MHU (MC_UU_12009); the NIHR Oxford BRC and John Fell Fund (131/030 and 101/517), the Edward Penley Abraham Cephalosporin Trust Fund (CF182 and CF170), and the WIMM Strategic Alliance (awards G0902418 and MC_UU_12025). The views expressed are those of the author(s) and not necessarily those of the NHS, the NIHR, the Department of Health, or the NIH.

AUTHOR CONTRIBUTIONS

A.R.-M. designed, performed, and analyzed experiments, performed bioinformatic analyses, and contributed to writing the manuscript. G.B. developed method automation protocols. G.B., S.A.C., B.J.P., V.A.D., E.L., and N.S. performed experiments. B.J.P. analyzed data. E.L., B.P., N.S., and A.H. processed clinical samples and provided clinical information. S.M. and N.B. provided bioinformatic pipelines. A.G. provided protocols and technical input. S.E.W.J. provided input in experimental design, analysis, and writing the manuscript. S.T. designed and supervised bioinformatic analyses. A.J.M. conceived and supervised the project, designed and analyzed experiments, and wrote the manuscript. All authors read and approved the submitted manuscript.

DECLARATION OF INTERESTS

The authors declare no competing interests.

Received: April 13, 2018
Revised: November 7, 2018
Accepted: January 7, 2019
Published: February 11, 2019

REFERENCES

Cheow, L.F., Courtois, E.T., Tan, Y., Viswanathan, R., Xing, Q., Tan, R.Z., Tan, D.S., Robson, P., Loh, Y.H., Quake, S.R., and Burkholder, W.F. (2016). Single-cell multimodal profiling reveals cellular epigenetic heterogeneity. Nat. Methods *13*, 833–836.

Clevers, H. (2011). The cancer stem cell: premises, promises and challenges. Nat. Med. *17*, 313–319.

Cooper, T.A., Wan, L., and Dreyfuss, G. (2009). RNA and disease. Cell *136*, 777–793.

David, C.J., and Manley, J.L. (2010). Alternative pre-mRNA splicing regulation in cancer: pathways and programs unhinged. Genes Dev. *24*, 2343–2364.

Dey, S.S., Kester, L., Spanjaard, B., Bienko, M., and van Oudenaarden, A. (2015). Integrated genome and transcriptome sequencing of the same cell. Nat. Biotechnol. *33*, 285–289.

Dimitriou, M., Woll, P.S., Mortera-Blanco, T., Karimi, M., Wedge, D.C., Doolittle, H., Douagi, I., Papaemmanuil, E., Jacobsen, S.E.W., and Hellström-Lindberg, E. (2016). Perturbed hematopoietic stem and progenitor cell hierarchy in myelodysplastic syndromes patients with monosomy 7 as the sole cytogenetic abnormality. Oncotarget *7*, 72685–72698.

Dobin, A., Davis, C.A., Schlesinger, F., Drenkow, J., Zaleski, C., Jha, S., Batut, P., Chaisson, M., and Gingeras, T.R. (2013). STAR: Ultrafast universal RNA-seq aligner. Bioinformatics *29*, 15–21.

Giustacchini, A., Thongjuea, S., Barkas, N., Woll, P.S., Povinelli, B.J., Booth, C.A.G., Sopp, P., Norfo, R., Rodriguez-Meira, A., Ashley, N., et al. (2017). Single-cell transcriptomics uncovers distinct molecular signatures of stem cells in chronic myeloid leukemia. Nat. Med. *23*, 692–702.

Hamblin, A., Burns, A., Tham, C., Clifford, R., Robbe, P., Timbs, A., Mason, J., Dreau, H., Weller, A., Jithesh, P., et al. (2014). Development and evaluation of the clinical utility of a next generation sequencing (NGS) tool for myeloid disorders. Blood *124*, 2373.

Han, K.Y., Kim, K.T., Joung, J.G., Son, D.S., Kim, Y.J., Jo, A., Jeon, H.J., Moon, H.S., Yoo, C.E., Chung, W., et al. (2018). SIDR: simultaneous isolation and parallel sequencing of genomic DNA and total RNA from single cells. Genome Res. *28*, 75–87.

Hedlund, E., and Deng, Q. (2018). Single-cell RNA sequencing: Technical advancements and biological applications. Mol. Aspects Med. *59*, 36–46.

Hosokawa, M., Nishikawa, Y., Kogawa, M., and Takeyama, H. (2017). Massively parallel whole genome amplification for single-cell sequencing using droplet microfluidics. Sci. Rep. *7*, 5199.

Hou, Y., Guo, H., Cao, C., Li, X., Hu, B., Zhu, P., Wu, X., Wen, L., Tang, F., Huang, Y., and Peng, J. (2016). Single-cell triple omics sequencing reveals genetic, epigenetic, and transcriptomic heterogeneity in hepatocellular carcinomas. Cell Res. *26*, 304–319.

Jahn, K., Kuipers, J., and Beerenwinkel, N. (2016). Tree inference for single-cell data. Genome Biol. *17*, 86.

Jan, M., Snyder, T.M., Corces-Zimmerman, M.R., Vyas, P., Weissman, I.L., Quake, S.R., and Majeti, R. (2012). Clonal evolution of preleukemic hematopoietic stem cells precedes human acute myeloid leukemia. Sci. Transl. Med. *4*, 149ra118.

Jiang, L., Li, Z., and Rui, L. (2008). Leptin stimulates both JAK2-dependent and JAK2-independent signaling pathways. J. Biol. Chem. *283*, 28066–28073.

Kandoth, C., McLellan, M.D., Vandin, F., Ye, K., Niu, B., Lu, C., Xie, M., Zhang, Q., McMichael, J.F., Wyczalkowski, M.A., et al. (2013). Mutational landscape and significance across 12 major cancer types. Nature *502*, 333–339.

Kharazi, S., Mead, A.J., Mansour, A., Hultquist, A., Böiers, C., Luc, S., Buza-Vidas, N., Ma, Z., Ferry, H., Atkinson, D., et al. (2011). Impact of gene dosage, loss of wild-type allele, and FLT3 ligand on Flt3-ITD-induced myeloproliferation. Blood *118*, 3613–3621.

Khurana, E., Fu, Y., Chakravarty, D., Demichelis, F., Rubin, M.A., and Gerstein, M. (2016). Role of non-coding sequence variants in cancer. Nat. Rev. Genet. *17*, 93–108.

Kiselev, V.Y., Kirschner, K., Schaub, M.T., Andrews, T., Yiu, A., Chandra, T., Natarajan, K.N., Reik, W., Barahona, M., Green, A.R., and Hemberg, M. (2017). SC3: consensus clustering of single-cell RNA-seq data. Nat. Methods *14*, 483–486.

Li, H., Handsaker, B., Wysoker, A., Fennell, T., Ruan, J., Homer, N., Marth, G., Abecasis, G., and Durbin, R.; 1000 Genome Project Data Processing Subgroup. (2009). The Sequence Alignment/Map format and SAMtools. Bioinformatics *25*, 2078–2079.

Li, J., Kent, D.G., Godfrey, A.L., Manning, H., Nangalia, J., Aziz, A., Chen, E., Saeb-Parsy, K., Fink, J., Sneade, R., et al. (2014). JAK2V617F homozygosity

drives a phenotypic switch in myeloproliferative neoplasms, but is insufficient to sustain disease. Blood *123*, 3139–3151.

Liao, Y., Smyth, G.K., and Shi, W. (2014). featureCounts: An efficient general purpose program for assigning sequence reads to genomic features. Bioinformatics *30*, 923–930.

Longo, D.L. (2017). Imatinib changed everything. N. Engl. J. Med. *376*, 982–983.

Macaulay, I.C., Haerty, W., Kumar, P., Li, Y.I., Hu, T.X., Teng, M.J., Goolam, M., Saurat, N., Coupland, P., Shirley, L.M., et al. (2015). G&T-seq: parallel sequencing of single-cell genomes and transcriptomes. Nat. Methods *12*, 519–522.

Macosko, E.Z., Basu, A., Satija, R., Nemesh, J., Shekhar, K., Goldman, M., Tirosh, I., Bialas, A.R., Kamitaki, N., Martersteck, E.M., et al. (2015). Highly parallel genome-wide expression profiling of individual cells using nanoliter droplets. Cell *161*, 1202–1214.

Magee, J.A., Piskounova, E., and Morrison, S.J. (2012). Cancer stem cells: impact, heterogeneity, and uncertainty. Cancer Cell *21*, 283–296.

Majeti, R., Park, C.Y., and Weissman, I.L. (2007). Identification of a hierarchy of multipotent hematopoietic progenitors in human cord blood. Cell Stem Cell *1*, 635–645.

McGranahan, N., and Swanton, C. (2017). Clonal heterogeneity and tumor evolution: Past, present, and the future. Cell *168*, 613–628.

Nagel, S., Venturini, L., Przybylski, G.K., Grabarczyk, P., Schneider, B., Meyer, C., Kaufmann, M., Schmidt, C.A., Scherr, M., Drexler, H.G., and Macleod, R.A. (2011). Activation of Paired-homeobox gene PITX1 by del(5)(q31) in T-cell acute lymphoblastic leukemia. Leuk. Lymphoma *52*, 1348–1359.

Ortmann, C.A., Kent, D.G., Nangalia, J., Silber, Y., Wedge, D.C., Grinfeld, J., Baxter, E.J., Massie, C.E., Papaemmanuil, E., Menon, S., et al. (2015). Effect of mutation order on myeloproliferative neoplasms. N. Engl. J. Med. *372*, 601–612.

Patel, A.P., Tirosh, I., Trombetta, J.J., Shalek, A.K., Gillespie, S.M., Wakimoto, H., Cahill, D.P., Nahed, B.V., Curry, W.T., Martuza, R.L., et al. (2014). Single-cell RNA-seq highlights intratumoral heterogeneity in primary glioblastoma. Science *344*, 1396–1401.

Paul, F., Arkin, Y., Giladi, A., Jaitin, D.A., Kenigsberg, E., Keren-Shaul, H., Winter, D., Lara-Astiaso, D., Gury, M., Weiner, A., et al. (2015). Transcriptional heterogeneity and lineage commitment in myeloid progenitors. Cell *163*, 1663–1677.

Picelli, S., Björklund, A.K., Faridani, O.R., Sagasser, S., Winberg, G., and Sandberg, R. (2013). Smart-seq2 for sensitive full-length transcriptome profiling in single cells. Nat. Methods *10*, 1096–1098.

Pierson, E., and Yau, C. (2015). ZIFA: Dimensionality reduction for zero-inflated single-cell gene expression analysis. Genome Biol. *16*, 241.

Pomares, H., Palmeri, C.M., Iglesias-Serret, D., Moncunill-Massaguer, C., Saura-Esteller, J., Núñez-Vázquez, S., Gamundi, E., Arnan, M., Preciado, S., Albericio, F., et al. (2016). Targeting prohibitins induces apoptosis in acute myeloid leukemia cells. Oncotarget *7*, 64987–65000.

Povinelli, B.J., Rodriguez-Meira, A., and Mead, A.J. (2018). Single cell analysis of normal and leukemic hematopoiesis. Mol. Aspects Med. *59*, 85–94.

Sanjuan-Pla, A., Macaulay, I.C., Jensen, C.T., Woll, P.S., Luis, T.C., Mead, A., Moore, S., Carella, C., Matsuoka, S., Bouriez Jones, T., et al. (2013). Platelet-biased stem cells reside at the apex of the haematopoietic stem-cell hierarchy. Nature *502*, 232–236.

Sauer, I., Schaljo, B., Vogl, C., Gattermeier, I., Kolbe, T., Müller, M., Blackshear, P.J., and Kovarik, P. (2006). Interferons limit inflammatory responses by induction of tristetraprolin. Blood *107*, 4790–4797.

Sims, D., Sudbery, I., Ilott, N.E., Heger, A., and Ponting, C.P. (2014). Sequencing depth and coverage: key considerations in genomic analyses. Nat. Rev. Genet. *15*, 121–132.

Smith, C.C., Paguirigan, A., Jeschke, G.R., Lin, K.C., Massi, E., Tarver, T., Chin, C.S., Asthana, S., Olshen, A., Travers, K.J., et al. (2017). Heterogeneous resistance to quizartinib in acute myeloid leukemia revealed by single-cell analysis. Blood *130*, 48–58.

Soh, J., Okumura, N., Lockwood, W.W., Yamamoto, H., Shigematsu, H., Zhang, W., Chari, R., Shames, D.S., Tang, X., MacAulay, C., et al. (2009). Oncogene mutations, copy number gains and mutant allele specific imbalance (MASI) frequently occur together in tumor cells. PLoS ONE *4*, e7464.

Tirosh, I., Izar, B., Prakadan, S.M., Wadsworth, M.H., 2nd, Treacy, D., Trombetta, J.J., Rotem, A., Rodman, C., Lian, C., Murphy, G., et al. (2016a). Dissecting the multicellular ecosystem of metastatic melanoma by single-cell RNA-seq. Science *352*, 189–196.

Tirosh, I., Venteicher, A.S., Hebert, C., Escalante, L.E., Patel, A.P., Yizhak, K., Fisher, J.M., Rodman, C., Mount, C., Filbin, M.G., et al. (2016b). Single-cell RNA-seq supports a developmental hierarchy in human oligodendroglioma. Nature *539*, 309–313.

Velten, L., Haas, S.F., Raffel, S., Blaszkiewicz, S., Islam, S., Hennig, B.P., Hirche, C., Lutz, C., Buss, E.C., Nowak, D., et al. (2017). Human haematopoietic stem cell lineage commitment is a continuous process. Nat. Cell Biol. *19*, 271–281.

Venteicher, A.S., Tirosh, I., Hebert, C., Yizhak, K., Neftel, C., Filbin, M.G., Hovestadt, V., Escalante, L.E., Shaw, M.L., Rodman, C., et al. (2017). Decoupling genetics, lineages, and microenvironment in IDH-mutant gliomas by single-cell RNA-seq. Science *355*, eaai8478.

Vogelstein, B., Papadopoulos, N., Velculescu, V.E., Zhou, S., Diaz, L.A., Jr., and Kinzler, K.W. (2013). Cancer genome landscapes. Science *339*, 1546–1558.

Wang, L., Wang, S., and Li, W. (2012). RSeQC: quality control of RNA-seq experiments. Bioinformatics *28*, 2184–2185.

Wang, Y., Waters, J., Leung, M.L., Unruh, A., Roh, W., Shi, X., Chen, K., Scheet, P., Vattathil, S., Liang, H., et al. (2014). Clonal evolution in breast cancer revealed by single nucleus genome sequencing. Nature *512*, 155–160.

Wang, L., Fan, J., Francis, J.M., Georghiou, G., Hergert, S., Li, S., Gambe, R., Zhou, C.W., Yang, C., Xiao, S., et al. (2017). Integrated single-cell genetic and transcriptional analysis suggests novel drivers of chronic lymphocytic leukemia. Genome Res. *27*, 1300–1311.

Wilm, A., Aw, P.P., Bertrand, D., Yeo, G.H., Ong, S.H., Wong, C.H., Khor, C.C., Petric, R., Hibberd, M.L., and Nagarajan, N. (2012). LoFreq: a sequence-quality aware, ultra-sensitive variant caller for uncovering cell-population heterogeneity from high-throughput sequencing datasets. Nucleic Acids Res. *40*, 11189–11201.

Woll, P.S., Kjällquist, U., Chowdhury, O., Doolittle, H., Wedge, D.C., Thongjuea, S., Erlandsson, R., Ngara, M., Anderson, K., Deng, Q., et al. (2014). Myelodysplastic syndromes are propagated by rare and distinct human cancer stem cells in vivo. Cancer Cell *25*, 794–808.

Xie, H., Xu, J., Hsu, J.H., Nguyen, M., Fujiwara, Y., Peng, C., and Orkin, S.H. (2014). Polycomb repressive complex 2 regulates normal hematopoietic stem cell function in a developmental-stage-specific manner. Cell Stem Cell *14*, 68–80.

Zhang, X., Su, J., Jeong, M., Ko, M., Huang, Y., Park, H.J., Guzman, A., Lei, Y., Huang, Y.-H., Rao, A., et al. (2016). DNMT3A and TET2 compete and cooperate to repress lineage-specific transcription factors in hematopoietic stem cells. Nat. Genet. *48*, 1014–1023.

Zheng, G.X., Terry, J.M., Belgrader, P., Ryvkin, P., Bent, Z.W., Wilson, R., Ziraldo, S.B., Wheeler, T.D., McDermott, G.P., Zhu, J., et al. (2017). Massively parallel digital transcriptional profiling of single cells. Nat. Commun. *8*, 14049.

Zheng, S., Papalexi, E., Butler, A., Stephenson, W., and Satija, R. (2018). Molecular transitions in early progenitors during human cord blood hematopoiesis. Mol. Syst. Biol. *14*, e8041.

Zhou, H., Ma, H., Wei, W., Ji, D., Song, X., Sun, J., Zhang, J., and Jia, L. (2013). B4GALT family mediates the multidrug resistance of human leukemia cells by regulating the hedgehog pathway and the expression of p-glycoprotein and multidrug resistance-associated protein 1. Cell Death Dis. *4*, e654.

STAR★METHODS

KEY RESOURCES TABLE

REAGENT or RESOURCE	SOURCE	IDENTIFIER
Antibodies		
CD8-FITC (Lineage)	BioLegend	Clone: RPA-T8<br>Cat#: 301006<br>RRID: AB_314124
CD20-FITC (Lineage)	BioLegend	Clone: 2H7<br>Cat#: 302304<br>RRID: AB_314252
CD66b-FITC (Lineage)	BioLegend	Clone: G10F5<br>Cat#: 305104<br>RRID: AB_314496
CD10-FITC (Lineage)	BioLegend	Clone: HI10a<br>Cat#: 312208<br>RRID: AB_314919
CD127-FITC (Lineage)	eBioscience	Clone eBioRDR5; Cat#: 11-1278-42<br>RRID: AB_1907343
Human Hematopoietic Lineage Cocktail – FITC (Lineage)	eBioscience	Cat# 22-7778-72; RRID: AB_1311229
CD123-PECy7	BioLegend	Clone: 6H6<br>Cat#: 306010<br>RRID: AB_493576
CD38-PETxRed	Invitrogen	Clone: HIT2<br>Cat#: MHCD3817<br>RRID: AB_10392545
CD90-BV421	BioLegend	Clone: 5E10<br>Cat#: 328122<br>RRID: AB_2561420
CD45RA-PE	eBioscience	Clone: HI100<br>Cat#: 12-0458-41<br>RRID: AB_10717397
CD34-APC-eF780	eBioscience	Clone: 4H11<br>Cat#: 47-0349-42<br>RRID: AB_2573956
CD34-PerCP/Cy5.5	BioLegend	Clone: 562<br>Cat# 343611, RRID:AB_2566787
CD90-PE	BioLegend	Clone: 5E10<br>Cat# 328109<br>RRID: AB_893442
CD45RA-FITC	Invitrogen	Clone: MEM56<br>Cat# MHCD45RA01<br>RRID: AB_10373858
CD2-PE/Cy5 (Lineage)	BioLegend	Clone: RPA-2.10<br>Cat# 300209<br>RRID:AB_314033
CD3-PE/Cy5 (Lineage)	BioLegend	Clone: HIT3a<br>Cat# 300310<br>RRID: AB_314046
CD4-PE/Cy5 (Lineage)	BioLegend	Clone: RPA-T4<br>Cat# 300510<br>RRID: AB_314078
CD7-PE/Cy5 (Lineage)	BioLegend	Clone: 6B7<br>Cat# 343110<br>RRID: AB_2075096

(*Continued on next page*)

Continued

REAGENT or RESOURCE	SOURCE	IDENTIFIER
CD8-PE/Cy5 (Lineage)	BioLegend	Clone: RPA-T8<br>Cat# 301010<br>RRID: AB_314128
CD10-PE/Cy5 (Lineage)	BioLegend	Clone: HI10a<br>Cat# 312206<br>RRID: AB_314917
CD11b-PE/Cy5 (Lineage)	BioLegend	Clone: ICRF44<br>Cat# 301308<br>RRID: AB_314160
CD14-PE/Cy5 (Lineage)	Invitrogen	Clone: 61D3<br>Cat# 15-0149-41<br>RRID: AB_2573057
CD19-PE/Cy5 (Lineage)	BioLegend	Clone: HIB19<br>Cat# AB_314240<br>RRID: 302210
CD20-PE/Cy5 (Lineage)	BioLegend	Clone: 2H7<br>Cat# AB_314256<br>RRID: 302308
CD56-PE/Cy5 (Lineage)	BD Biosciences	Clone: B159<br>Cat# 555517<br>RRID: AB_395907
CD235a,b-PE/Cy5 (Lineage)	BioLegend	Clone: HIR2<br>Cat# 306606<br>RRID: AB_314624
Biological Samples		
Healthy Donors (HD7643; HD7650; Aph1; HD85) and MPN patient samples (OX2123; IF0602; IF0111; SMD32316; OX4739; IF0101; IF0123; IF0137; IF0138; IF0140; IF0155; IF0157; See Table 1 and Table S3)	INForMeD Study (REC:199833, University of Oxford)	https://www.hra.nhs.uk/planning-and-improving-research/application-summaries/research-summaries/the-informed-study/
Chemicals, Peptides, and Recombinant Proteins		
Protease	QIAGEN	Cat# 19155
RNase Inhibitor	Takara (Clontech)	Cat# 2313A
SMARTScribe	Takara (Clontech)	Cat# 639537
SeqAMP	Takara (Clontech)	Cat# 638509
Critical Commercial Assays		
Nextera XT DNA Library Preparation Kit	Illumina	Cat# FC-131-1096
Nextera XT Index Kit v2 Set A	Illumina	Cat# FC-131-2001
Nextera XT Index Kit v2 Set C	Illumina	Cat# FC-131-2003
KAPA 2G Robust HS PCR Kit	Kapa Biosystems	Cat# KK5517
FastStart High Fidelity PCR System, dNTPack - Sigma-Aldrich	Roche	Cat# 04-738-292 001
Access Array™ Barcode Library for Illumina® Sequencers-384, Single Direction	Fluidigm	Cat# 100-4876
Deposited Data		
Single-cell RNA sequencing	this paper	GEO: GSE105454
Targeted genotyping sequencing (validation; Figure 1)	this paper	SRA: PRJNA503734
Targeted genotyping sequencing (patients processed using full-length TARGET-seq; Figures 3–4)	this paper	SRA: PRJNA503736
Targeted genotyping sequencing (patients processed using 3′-TARGET-seq; Figures 5 and 6; Figures S6 and S7)	this paper	SRA: PRJNA503628
Experimental Models: Cell Lines		
K562	ATCC	RRID:CVCL_0004
MOLT4	ATCC	RRID:CVCL_0013

(*Continued on next page*)

Continued

REAGENT or RESOURCE	SOURCE	IDENTIFIER
NALM6	DSMZ	RRID:CVCL_0092
SET2	Laboratory of Dr. Jacqueline Boultwood	RRID:CVCL_2187
JURKAT	ATCC	RRID:CVCL_0367
Oligonucleotides		
OligodT-ISPCR (HPLC purification): aagcagtggtatcaacgcagagtacttttttttttttttttttttttttttttttvn	Picelli et al., 2013	N/A
TSO-LNA (RNase Free HPLC purification): AAGCAGTGGTATCAACGCAGAGTACATrGrG+G	Picelli et al., 2013	N/A
ISPCR (HPLC purification): AAGCAGTGGTATCAACGCAGAGT	Picelli et al., 2013	N/A
P5_index (HPLC purification): AATGATACGGCGACCACCGAGATCTACACGCCTGTC CGCGGAAGCAGTGGTATCAACGCAGAGT*T*G	this paper; adapted from Zheng et al., 2018	N/A
P5_SEQ (PAGE purification): GCCTGTCCGCGGAAGCAGTGG TATCAACGCAGAGTTGC*T	this paper; adapted from Zheng et al., 2018	N/A
CS1-seq sequencing primer (HPLC purification): A+CA+CTG+ACGACATGGTTCTACA	N/A	N/A
CS2-seq sequencing primer (HPLC purification): T+AC+GGT+AGCAGAGACTTGGTCT	N/A	N/A
CS1rc-seq sequencing primer (HPLC purification): T+GT+AG+AACCATGTCGTCAGTGT	N/A	N/A
CS2rc-seq sequencing primer (HPLC purification): A+GAC+CA+AGTCTCTGCTACCGTA	N/A	N/A
See Table S2 for pre-amplification, barcoding PCR1 target-specific primer sequences and barcoded oligodT-ISPCR primers	this paper and adaptor from Zheng et al., 2018	N/A
Software and Algorithms		
bcl2fastq (version 2.20)	Illumina	RRID:SCR_015058
STAR (version 2.4.2a)	Dobin et al., 2013	https://github.com/alexdobin/STAR RRID: SCR_015899
TrimGalore (version 0.4.1)	Felix Krueger, The Babraham Institute	https://www.bioinformatics.babraham.ac.uk/projects/trim_galore/
FeatureCounts (version 1.4.5-p1)	Liao et al., 2014	http://subread.sourceforge.net/ RRID: SCR_012919
Samtools (version 1.1)	Li et al., 2009	http://samtools.sourceforge.net/ RRID:SCR_002105
R (version 3.4.3)	CRAN	RRID:SCR_001905
Flowjo	Tree Star	RRID:SCR_008520
Gene set enrichment analysis (GSEA)	Broad Institute	RRID:SCR_003199
MSigDB	Broad Institute	RRID:SCR_003199
Graphpad Prism (version 7)	Graphpad	RRID:SCR_002798
Other		
Full-length TARGET-seq, 3′TARGETseq detailed protocols and primer design and validation technical note	This Paper	Methods S1

CONTACT FOR REAGENT AND RESOURCE SHARING

Further information and requests for resources and reagents should be directed to and will be fulfilled by the Lead Contact, Adam Mead (adam.mead@imm.ox.ac.uk).

METHOD DETAILS

Cell Lines

K562, MOLT4 and JURKAT cells were obtained from the American Type Culture Collection (ATCC). NALM6 cells were obtained from the German Collection of Microorganisms and Cell Cultures (DSMZ). SET2 cells were kindly provided by Dr. Jacqueline Boultwood and Dr. Andrea Pellagatti (Radcliffe Department of Medicine, University of Oxford). All cell lines were maintained in culture in RPMI-1640 supplemented with 10% Fetal Calf Serum (FCS) and antibiotics. Cell lines were authenticated by targeted sequencing of known mutations.

Banking and Processing of Human Samples

Patients and normal donors provided written informed consent in accordance with the Declaration of Helsinki for sample collection and use in research under the INForMeD Study (REC:199833, University of Oxford). Cryopreserved peripheral blood and bone marrow mononuclear cells (MNCs) were thawed and processed for flow cytometry analysis as previously described (Woll et al., 2014). Briefly, cryopreserved cells were thawed and 1 mL of FCS was immediately added to each sample. Samples were further diluted with 8mL IMDM (Iscove's Modified Dulbecco's Medium) supplemented with 20% FCS and 10% DNase I (Merck). Samples were spun down for 10 min at 350 g, washed and spun down again for 10 min at 350 g. A summary of patients and normal donors' samples used for analysis can be found in Table 1 and Table S3.

Bulk Sequencing of Mononuclear Cells

Bulk genomic DNA from patient samples' mononuclear cells was isolated using DNeasy Blood & Tissue Kit (QIAGEN) as per manufacturer's instructions. Targeted sequencing was performed using a TruSeq Custom Amplicon panel (Illumina) consisting of 341 amplicons (~56 kb) designed around exons of 32 genes frequently mutated in myeloid malignancies (Hamblin et al., 2014). Library preparation was performed as per manufacturer's instructions using 50-250 ng genomic DNA.

Targets were chosen based on the genes/exons most frequently mutated and/or likely to alter clinical practice (diagnostic, prognostic, predictive or monitoring capacity) across a range of myeloid malignancies (e.g., MDS/AML/ MPN), and can be found in the table below:

Gene	Exons Covered	Gene	Exons Covered
ASXL1	12	*KRAS*	2, 3
ATRX	8, 9, 10, 17-31	*MPL*	10
CBL	8-9	*NPM1*	11
CBLB	9-10	*NRAS*	2,3
CBLC	9-10	*PDGFRA*	12, 14, 18
CEBPA	1	*PHF6*	2-10
CSF3R	14-17	*PTEN*	5-7
DNMT3A	23	*RUNX1*	3-8
ETV6	1-8	*SETBP1*	4
EZH2	2-20	*SF3B1*	14,15
FLT3	14, 15, 20	*SRSF2*	1
HRAS	2,3	*TET2*	3-11
IDH1	4	*TP53*	4-9
IDH2	4	*U2AF1*	2, 6
JAK2	12,14	*WT1*	7, 9
KIT	2, 8-11, 13, 17	*ZRSR2*	1-11

Alignment and variant calling were performed in Basespace (utilizing BWA and GATK/Somatic variant caller; Illumina, or SVC) while filtering and annotation were performed using Variant Studio (Illumina).

Every variant was individually assessed against COSMIC, dbSNP, gnomAD and published literature for frequency in the germline and acquired state and whether any data (*in* vitro or *in vivo*) suggests its likely pathogenicity. Variants with a population frequency of > 1% were considered polymorphisms. Variants with a population frequency of < 1% but with ethnicity bias and a variant allele frequency close to 50% were also considered polymorphisms.

Any variant passing these criteria and a variant allele frequency cut-off of 5% of the reads (point mutations) or 2% of reads (insertions/deletions longer than 5 bp) was reported as mutated in Table S3 and analyzed for each patient.

Fluorescent Activated Cell Sorting (FACS) Staining and Single-Cell Isolation

Single cell FACS-sorting was performed as previously described (Giustacchini et al., 2017), using BD Aria III or BD Fusion I instruments (Becton Dickinson) for 96-well plate experiments and SH800S (SONY) for 384-well plate experiments. Full details are provided in Supplemental Experimental Procedures. Experiments involving isolation of human hematopoietic stem and progenitor cells (HSPCs) included single color stained controls (CompBeads, BD Biosciences) and Fluorescence Minus One controls (FMOs). Lineage-CD34+ cells were sorted and indexed for CD38, CD90, CD45RA and CD123 markers, which allowed us to record the fluorescence levels of each marker for each single cell. For samples processed using full-length TARGET-seq in 96 well-plates (Table S3), HSPCs were stained with the following the antibody cocktail: Lineage-FITC, CD34-APC-e780, CD38-PE-TxRed, CD90-BV421, CD45RA-PE and CD123-PECy7. For samples processed using 3′-TARGET-seq in 384-well plates (Table S3), HSPCs were stained with the following antibody cocktail: Lineage-PE/Cy5, CD34-PerCp/Cy5.5, CD38-PE-TxRed, CD90-PE, CD45RA-FITC, CD123-PECy7. The full list of antibodies used for HSPCs immunophenotyping and isolation can be found in Key Resources; 7- aminoactinomycin D (7-AAD) was used for dead cell exclusion. Briefly, single cells directly sorted into 96-well PCR plates containing 4.1-4.2 μL of lysis buffer or into 384-well plates containing 2.07 μL of lysis buffer . K562 cells were sorted into the lysis buffer described in Table S1A. JURKAT, MOLT4, NALM6, SET2 and HSPCs (processed using full length TARGET-seq) were sorted into lysis buffers described in Table S1B. HSPCs processed using 3′-TARGET-seq were sorted into the lysis buffer described in Table S1C, using the barcoded oligodT-ISPCR primers listed in Table S2C (adapted from (Zheng et al., 2018)). Flow cytometry profiles of the HSPC compartment (Figure S4) were analyzed using FlowJo software (version 10.1).

cDNA Synthesis (RT-PCR)

For K562 cells, RT and PCR steps were performed as described in Table S1A, using 18 cycles of PCR amplification. For JURKAT, MOLT4, NALM6, SET2 cells and HSPCs (full length TARGET-seq), RT and PCR steps were performed as described in Table S1B, using 20 cycles of PCR amplification for cell lines and 22 cycles of amplification for HSPCs. For HSPCs processed using 3′-TARGET-seq, RT and PCR steps were performed as described in Table S1C, using 24 cycles of PCR amplification. The sequences of the primers used in the RT and PCR steps, for whole transcriptome and targeted retrotranscription and cDNA amplification, can be found in Table S2A and Key Resources Table. Primers were designed to amplify amplicons 250-700 bp long and specificity was checked against RefSeq and human genome assembly databases using PrimerBlast. mRNA and cDNA primers were designed to amplify coding regions whereas gDNA primers were designed to bind at least to one intronic region. More information regarding primer design and validation can be found in the Supplemental Experimental Procedures "Technical Note: Primer Design and Validation." After PCR, an aliquot of the cDNA-amplicon mix was used for whole transcriptome library preparation and another aliquot, for single-cell genotyping library preparation. For full length TARGET-seq, 15 μL from a total of 25 μL of cDNA-amplicon mix were diluted with 11 μL of water, purified using 16 μL of Ampure XP Beads (0.6:1 beads to cDNA ratio; Beckman Coulter), and resuspended in a final volume of 8 μL of EB buffer (QIAGEN). For high throughput 3′-TARGET-seq, 1 μL from each quadrant of a 384-well plate was pooled to generate a cDNA pool of barcoded libraries; each cDNA pool was purified twice using Ampure XP beads (0.6:1 beads to cDNA ratio). The quality of cDNA traces was checked using a High Sensitivity DNA Kit in a Bioanalyzer instrument (Agilent Technologies). The remaining of the cDNA-amplicon mix was used for subsequent single-cell genotyping or stored at −20 C.

Targeted NGS Single-Cell Genotyping

After RT-PCR, 1.5 μL aliquot from each single cell derived cDNA+amplicon mix was used as input to generate a targeted and Illumina-compatible library for single cell genotyping. The preparation of single cell genotyping libraries involves 2 PCR steps (See Supplemental Experimental Procedures). In the first PCR step, target specific primers (Table S2B) attached to universal CS1 / CS2 adaptors (Figure 1, Forward adaptor, CS1: ACACTGACGACATGGTTCTACA; Reverse adaptor, CS2: TACGGTAGCAGAGACTTGGTCT) are used to amplify the target regions of interest. Target-specific primers were designed to specifically amplify cDNA or gDNA, amplifying annotated coding regions in the case of cDNA amplicons and at least one intronic region in the case of genomic DNA amplicons. In the second PCR step (See Detailed Protocol), Illumina compatible adaptors (PE1/PE2) containing 10 bp single-direction indexes (Access Array Barcode Library for Illumina® Sequencers-384, Single Direction, Fluidigm) are attached to pre-amplified amplicons from the first PCR through CS1/CS2 regions, to generate single-cell barcoded libraries. Amplicons were pooled using a Mosquito HTS liquid handling platform (TTP Labtech) and pooled amplicons were purified with Ampure XP beads (0.8:1 ratio beads to product; Beckman Coulter). Purified pools were quantified using Quant-iT Picogreen (Thermo Fisher Scientific) and each pool was diluted to a final concentration of 4 nM. Pools were further diluted to 10 pM in HT1 buffer prior sequencing.

Up to 384 single cells were sequenced on a MiSeq (Illumina) instrument, with the following sequencing configuration: 151 bp R1, 10 bp index read, 151 bp R2. We used custom sequencing primers for Read1 and Read 2 (500 nM CS1-seq and 500 nM CS2-seq; See Key Resources) and Index Read (500 nM CS1rc-seq and 500 nM CS2rc-seq; See Key Resources) diluted in 700 μL of HT1 buffer. Reads were aligned to GRCh37/hg19 using STAR with default settings (version 2.4.2a) and cDNA/gDNA amplicons were separated into different bam files using a custom pipeline, extracting reads matching the different primer sequences used for targeted PCR barcoding. This allowed us to obtain independent mutational information from cDNA and gDNA. Variant calling was performed using mpileup (samtools version 1.1, options–minBQ 30,–count-orphans,–ignore overlaps) and results were summarized with a custom pipeline (https://github.com/albarmeira/TARGET-seq; Figure S2A). Thresholds for the detection of each amplicon were set based on non-template controls and thresholds for mutation calling were based on WT controls and customized for each amplicon

(1.5%–4% of the reads, representative examples can be found in Figure S2B). Both non-template and WT controls were routinely processed in parallel to test samples. Importantly, none of the tested mutations were detected in any control cells (n = 874) or blanks (n = 114) in any of the experiments using the mutational pipeline and cut-offs described, implying that the false positive rate of variant calling is effectively zero. For experiments involving isolation of HSPCs, QC genotyping was performed as follows: single cells where one of the targeted amplified genes tested failed to be detected by either gDNA or mRNA were excluded from analysis. Cells for which cDNA/gDNA mutation analysis showed discrepant readouts were considered heterozygous if one of the molecules (cDNA or gDNA) gave a heterozygous readout. When one of the molecules gave a homozygous readout and the other gave a WT readout, cells were also considered heterozygous, although this was a rare event occurring in 0.18% of the amplicons. We considered a cell homozygous when only the mutant allele was detected at the genomic DNA level and we considered a cell WT when only the WT allele was detected at the genomic DNA level. We excluded cells in which only the WT or mutant allele were detected at the mRNA level, but the same gene was not detected at the gDNA level, a rare event occurring in 0.57% of amplicons. Specifically for *JAK2* mutation, where we carried out extensive analysis of the data for zygosity, we included an additional "not determined" category for cells with mRNA and gDNA *JAK2* amplicons in which allele frequency was 0.03 < AF < 0.1 for gDNA (full-length TARGET-seq dataset), 0.04 < AF < 0.1 for gDNA (3′-TARGET-seq dataset) and 0.03 < AF < 0.1 for mRNA (3′-TARGET-seq dataset). Not determined amplicons were excluded from analysis: 36 of 3900 amplicons detected for gJAK2 and 51 out of 1295 amplicons detected for mJAK2. We required a minimum coverage of 30 reads per amplicon to obtain mutational readouts; the mean coverage per amplicon is 2641 reads.

Nextera XT Library Preparation for Full-Length Whole-Transcriptome Sequencing

Bead-purified cDNA libraries were used for tagmentation with Nextera XT DNA Kit (Illumina) using one fourth of the original volume as previously described (Giustacchini et al., 2017). 4nM libraries were diluted to 1.8 pM in HT1 buffer and sequenced on a NextSeq instrument with 75 bp single-end reads using a NextSeq 500/550 High Output v2 kit (Illumina). HSPCs were sequenced to a mean sequencing depth of 2.4 M reads.

Nextera XT Library Preparation for 3′-Biased Whole-Transcriptome Sequencing

Bead-purified and pooled cDNA libraries were used for tagmentation-based library preparation with Nextera XT DNA Kit (Illumina) using a custom PCR amplification strategy. Briefly, 1 ng of each barcoded cDNA pool was tagmented as per manufacturer's instructions. Subsequently, reaction was stopped and PCR was performed as per manufacturer's instructions, with the exception of P5 adaptor, for which 200 nM of a custom P5 adaptor was used (P5_index; See Key Resources). Each indexed pool was bead purified twice with Ampure XP beads (0.7:1 beads to cDNA ratio). 4nM libraries were diluted to 3 pM in a total volume of 1.3 mL of HT1 buffer and were sequenced on a NextSeq instrument, using a NextSeq 500/550 High Output v2 kit (Illumina) with a custom sequencing primer for read1 (P5_SEQ, 900 nM in a total volume of 3 mL of HT1 buffer; See Key Resources) and the following sequencing configuration: 20 bp R1; 8 bp index read; 64 bp R2. HSPCs were sequenced to a mean sequencing depth of 152,552 reads.

Single Cell Full-Length RNA-Sequencing Data Pre-Processing

RNA-sequencing reads were trimmed for Nextera adaptors with TrimGalore (version 0.4.1) and aligned to the human genome (hg19) using STAR with default settings (version 2.4.2a). RefSeq gene model was used as the reference for gene expression quantification. Counts for each RefSeq gene were obtained with FeatureCounts (version 1.4.5-p1; options–primary) and were normalized to reads per kilobase per million mapped reads (RPKM). Genes with RPKM values less than 1 were considered non-detected (Giustacchini et al., 2017) and expression values for these genes were converted to zero. We further normalized RPKM expression values into the log2 scale. QC filtering was performed using the following parameters: percentage of reads mapping in exons > 50%, percentage of mapped reads > 50% and number of detected genes per cell (RPKM > = 1) > 6000 for JURKAT and SET2 cells, > 5000 for K562 cells and > 1500 for primary HSPCs. For cell lines, we excluded 8 cells after applying these QC filters (5.3%) and for HSPCs, 33 cells (6.1%).

Single Cell 3′-Biased RNA-Sequencing Data Pre-Processing

FASTQ files were generated using bcl2fastq (version 2.20) with default parameters and the following read configuration: Y12N*, I8, Y64N*, in which read1 corresponds to an 8bp cell-specific barcode, index read corresponds to i7 index from each cDNA pool and read2 corresponds to cDNA sequence. Demultiplexed FASTQ files were trimmed for polyA tails using TrimGalore (version 0.4.1); files from different lanes were merged together using samtools (version 1.1) and aligned to the human genome using STAR (version 2.4.2a). RefSeq gene model was used as the reference for gene expression quantification. Counts for each RefSeq gene were obtained with FeatureCounts (version 1.4.5-p1; options–primary). Counts were normalized as follows: counts for each single cell were divided by the total library size for that cell and multiplied by the mean library size of all cells processed (68,412). Genes with normalized count values less than 1 were considered non-detected and expression values for these genes were converted to zero. We further normalized counts into the log2 scale. QC filtering was performed using the following parameters: library size > 2000 reads; percentage of reads mapping to the mitochondrial chromosome < 10%; percentage of ERCC < 50% and number of detected genes per cell (normalized counts > = 1) > 500. We retained 2851 cells after applying these QC filters (81.6%).

Whole-Transcriptome Variant Calling from Single Cells

Bam files from 48 single K562 cells (Figure S1F) or 38 single HSPCs (Figure S1H) were merged using samtools to computationally create a single cell ensemble. LoFreq software (Wilm et al., 2012) was used for variant calling in the single cell ensemble. Heterozygous regions across the transcriptome (AF > 0.05 of the minor allele, Allele Frequency) were used for variant calling in each individual cell, requiring a minimum coverage of 10 reads and minimum base quality of 30. A SNV was considered heterozygous if 0.05 < AF < 0.95 and homozygous if AF < 0.05 or AF < 0.95.

Mutational Analysis from RNA-Sequencing Reads

Variant calling from raw RNA-sequencing reads was performed using mpileup (samtools version 1.1, options–minBQ 30,–count-orphans,–ignore overlaps) and results were summarized with a custom script (https://github.com/albarmeira/TARGET-seq). Thresholds for the detection of amplicons were set at 30 reads per position (Figure S2C), in line with variant calling guidelines (Sims et al., 2014).

Dropout Frequency and Library Bias Calculation

The frequency of dropout for a given gene was calculated as the percentage of cells from a specific condition (SMART-seq2 or SMART-seq+) in which the gene is not detected (RPKM < 1), as compared to the average expression of that gene in K562 bulk samples (6 replicates of 100 cells each; 3 replicates per chemistry). Library bias was calculated as the ratio between the mean RPKM of the top 10% expressed genes in the library and the mean RPKM of all genes.

Transcript Coverage

Normalized transcript coverage was calculated using "geneBody_coverage.py" script from RSeQC package (Wang et al., 2012), using a list of 4040 housekeeping genes obtained from http://rseqc.sourceforge.net/.

Differential Expression Analysis

Differentially expressed genes were identified using a combination of non-parametric Wilcoxon test, to compare the expression values for each group, and Fisher's exact test, to compare the frequency of expression for each group, as previously described (Giustacchini et al., 2017). We used log2(RPKM) and log2(normalized counts) matrices, including genes expressed in at least two cells (when analyzing less than 200 cells; Table S4) or in at least five cells (when analyzing over 200 cells; Tables S5, and S6). P values were combined using Fisher's method and adjusted p values were derived using Benjamini & Hochberg procedure. Significant genes were selected on the basis of adjusted *P* value < 0.1 and absolute log2(fold change)>0.5. Differentially expressed genes in between several distinct genetic subclones (Figure 6, and Table S6) were identified using the "genefilter" package in R with analysis of variance (p value < 0.05). Beeswarm plots from selected genes were generated using "beeswarm" package in R and boxplots from selected genes were generated using "ggplot2" package in R.

Identification of Highly Variable Genes

We identified variable genes above technical noise by fitting a lowess model of the log2(mean expression level) and coefficient of variation for each gene. We selected genes with a coefficient of variation above the fitted model and log2(mean expression) > = 0.

Single Cell Clustering and Dimensionality Reduction

T-distributed stochastic neighbor embedding (tSNE) was performed using 'Rtsne' package, the implementation of the method in R, with "perplexity" = 15 for Figures 4A and 4B "perplexity=20" for Figures 2B and 4C. For the analysis of 3′-TARGET-seq, similarly to other high-throughput 3′-biased techniques, we first computed a PCA reduction using 50 dimensions, and then used the top thirty (Figures 5A, 5D, 5E, and 5G), top twenty (Figure 5H) or top five dimensions (Figures 6A, 6C, 6E, and S7A–F) with higher variance to generate the tSNE plots in Figures 5, 6, and S7, using "perplexity=20" for Figure 5H, "perplexity=25" for Figures 6A, 6C, 6E, and S7A–F, and "perplexity=30" for Figures 5A, 5D, 5E, and 5G. The number of genes used for each analysis is specified in the legend for each figure. Zero Inflated Factor Analysis (ZIFA) (Pierson and Yau, 2015) was used to assess transcriptional heterogeneity associated with the subclonal composition of patients IF0137, IF0138 and IF0101 (Figures S7G–I), performed using highly variable genes with default parameters. SC3 software (Kiselev et al., 2017) was used to analyze the subclonal composition of patients IF0137, IF0138 and IF0101, using default parameters and k = 4 for patient IF0137 (as there are four genetically-distinct subclones; Figure S7J) or k = 3 for patients IF0138 and IF0101 (as there are three genetically-distinct subclones; Figures S7K and S7L) with default parameters. K-Nearest Neighbors clustering integrated in the PAGODA2 package (https://github.com/hms-dbmi/pagoda2) was used to analyze the subclonal composition of patients IF0137, IF0138 and IF0101 (Figures S7M–O). We calculated a PCA reduction of the batch-corrected gene expression matrix using 50 principal components and 3000 overdispersed genes, computed nearest neighbors using "cosine" distance (k = 15) and identified clusters using "multilevel community" method. We then plotted the tSNE graphs presented in Figures S7M–O with "perplexity=25." We observed that transcriptional heterogeneity between genetic subclones within individual patients was better captured with higher-dimensionality representations, and we therefore represent three tSNE dimensions in Figures 6 and S7.

Cell to Cell Correlation Measurements

Pearson's correlation between single cells for each genetic subgroup was calculated using the log2(normalized counts), including genes expressed in at least five cells (Figure 5C).

Batch Correction

Batch correction was performed using "limma" package in R (Figures 4, 5, 6, and S7). Gene expression matrix was batch and donor corrected in Figures 4C, 5A, and 5H, while preserving genotypes. Gene expression matrix was batch corrected in Figures 5D, 5E, and 5G, while preserving donor effect. Gene expression matrix was batch corrected in Figures S7A and S7D and plate corrected in Figures S7C and S7F. We used batchNorm function from PAGODA2 package (method = "glm") to perform batch correction in Figures S7M and S7O.

Cell Cycle Phase Assignment and Correction

An S-phase and G2M-phase cell cycle score was calculated as the mean expression value of a set of S-phase and G2M-phase genes (Tirosh et al., 2016a) for each cell. S-phase and G2M-phase scores were used to fit a linear model on the normalized and logged gene expression matrices using "limma" package in R, in order to remove the effect of cell cycle. Cell-cycle corrected matrices were used as an input for the analysis presented in Figures 5A, 5H, and S7D–F.

Random Forest Analysis

Random forest analysis was performed using 'randomForest' package in R (ntree = 2000), trained on the genotypes of single cells. Only genotypes with at least five cells were included in this analysis. Expression matrix was batch and donor-corrected, and genotypes were preserved. The top 2000 genes identified by the random forest analysis (MeanDecreaseGini > 0.041 in Figure 4C; MeanDecreaseGini > 0.045 in Figure 5H) were used for the tSNE representation in Figures 4C and 5H (perplexity = 20). Clustering of cells was stable when selecting from 500 to 5000 top genes from the random forest analysis.

GeneSet Enrichment Analysis

GSEA was performed using GSEA software (http://software.broadinstitute.org/gsea) with default parameters and 1000 permutations on the phenotype. Gene sets used for the analysis were downloaded from MSigDB or relevant studies (Table S4H). Single Sample GSEA (ssGSEA) was performed using ssGSEA Projection Module (https://genepattern.broadinstitute.org) with default settings and combine mode 'combine.off'. A projection of ssGSEA results is shown in Figure 4B.

QUANTIFICATION AND STATISTICAL ANALYSIS

Unpaired Student t test with Welch's correction was used for the comparisons in Figures S1A, S1B, S1D, S1E, 2C, and S3A. Kolmogorov-Smirnov test was used for the comparison of Pearson's correlations distributions in Figure 5C.

Computational Reconstruction of Clonal Hierarchies

Phylogenetic tree reconstruction for patients with more than one driver mutation was performed using SCITE (Jahn et al., 2016) with default parameters and "-r 1 -l 900000 -fd 0.001 -ad 0.01 0.01 -cc 0." We accounted for Loss of Heterozygosity in *JAK2* by introducing the mutational status of each *JAK2* allele as separate components of the mutational matrix.

Code Availability

R, Perl and Python scripts used for the analysis are available upon request or accessible at https://github.com/albarmeira/TARGET-seq. Genotyping pipeline used for analysis of targeted-sequencing data generated by TARGET-seq (SCpipeline) can be downloaded from https://github.com/albarmeira/TARGET-seq.

DATA AND SOFTWARE AVAILABILITY

Single cell RNA-sequencing data is available at GEO: GSE105454. Single cell targeted sequencing data is available at the NCBI's SRA data repository with project number SRA: PRJNA503734 (validation experiments), SRA: PRJNA503736 (full-length TARGETseq patients' dataset) and SRA: PRJNA503628 (3′-TARGETseq patients' dataset).

ADDITIONAL RESOURCES

Detailed protocols and primer design technical note: a detailed full-length TARGET-seq, 3′-TARGET-seq protocol and a Technical Note describing primer design and validation is provided as Supplemental Experimental Procedures.

Molecular Cell, Volume *73*

Supplemental Information

Unravelling Intratumoral Heterogeneity through High-Sensitivity Single-Cell Mutational Analysis and Parallel RNA Sequencing

Alba Rodriguez-Meira, Gemma Buck, Sally-Ann Clark, Benjamin J. Povinelli, Veronica Alcolea, Eleni Louka, Simon McGowan, Angela Hamblin, Nikolaos Sousos, Nikolaos Barkas, Alice Giustacchini, Bethan Psaila, Sten Eirik W. Jacobsen, Supat Thongjuea, and Adam J. Mead

Figure S1

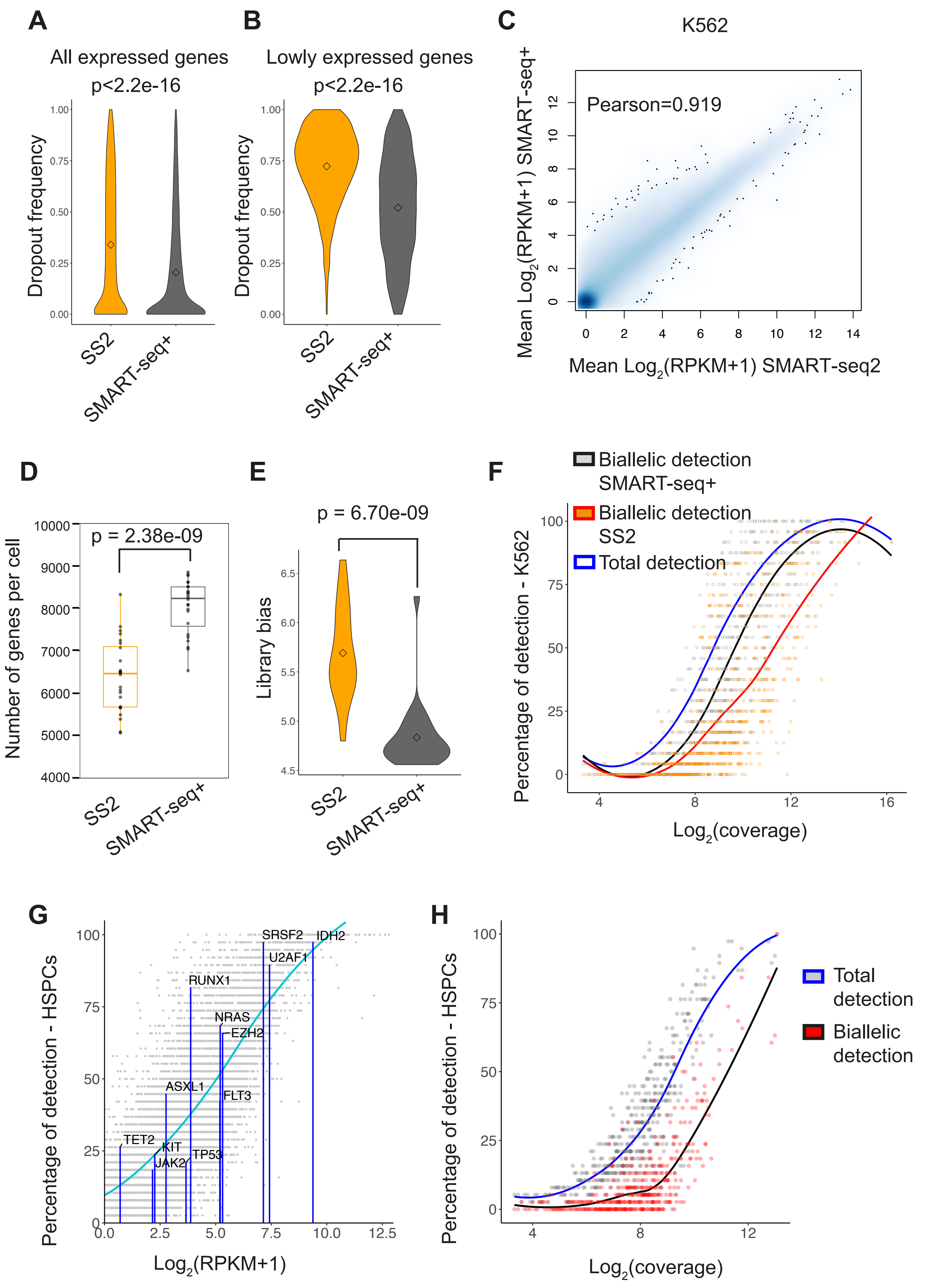

A
All expressed genes
p<2.2e-16
Dropout frequency
SS2
SMART-seq+
B
Lowly expressed genes
p<2.2e-16
C
K562
Pearson=0.919
Mean Log2(RPKM+1) SMART-seq+
Mean Log2(RPKM+1) SMART-seq2
D
p = 2.38e-09
Number of genes per cell
E
p = 6.70e-09
Library bias
F
Biallelic detection SMART-seq+
Biallelic detection SS2
Total detection
Percentage of detection - K562
Log2(coverage)
G
Percentage of detection - HSPCs
Log2(RPKM+1)
SRSF2
IDH2
U2AF1
RUNX1
NRAS
EZH2
ASXL1
FLT3
TET2
KIT
JAK2
TP53
H
Total detection
Biallelic detection

Figure S1, related to Figure 1. Single-cell RNA-sequencing is associated with high levels of allelic dropout. (a) Comparison of dropout frequency between SMART-seq2 (SS2) and SMART-seq+ methods (n=48 single K562 cells; 24 cells per chemistry from three independent experiments) for all genes expressed in K562 bulk samples (RPKM>1; 9096 genes). P-value from two-tailed unpaired Student's t-test is shown on the top of the graph. Points represent the mean for each group. **(b)** Comparison of dropout frequency for lowly expressed genes (2>RPKM>1; 1347 genes) between SMART-seq2 (SS2) and SMART-seq+ methods for the same cells as in (a). P-value from two-tailed unpaired Student's t-test is shown on the top of the graph. Points represent the mean for each group. **(c)** Pearson's correlation between mean log2(RPKM+1) values for SMART-seq2 and SMART-seq+ chemistries (n=48). **(d)** Number of detected genes per cell in K562 cells processed using Smart-seq2 or optimized SMART-seq+ chemistry for the same cells as in (a-c). P-value from two-tailed unpaired Student's t-test is shown on the top of the graph. Boxes represent median and quartiles and points represent the value for each single cell. **(e)** Library bias per chemistry, calculated as the ratio between the mean RPKM values of the top 10% expressed genes and the mean RPKM for all genes expressed in the library, using the same 48 cells as in (a-d). P-value from two-tailed unpaired Student's t-test is shown on the top of the graph. Points represent the mean for each group. **(f)** Percentage of total (dark blue line) or bi-allelic detection in heterozygous SNVs for Smart-seq2 (orange dots and red line) or optimized SMART-seq+ (grey dots and black line) chemistries (n=48 single K562 cells). Lines represent the mean percentage of detection (y-axis) with respect to log2(coverage; x-axis) and points represent individual SNVs. **(g)** Total percentage of detection of selected myeloid genes in Lin-CD34+CD38- hematopoietic stem/progenitor cells (HSPC; n=38; y-axis) with respect to the average level of expression for each gene (log2(RPKM+1); x-axis). Blue bars represent detection of specific gene transcripts that are frequently mutated in myeloid malignancies. The light blue line represents the average percentage of detection for a certain expression value (number of cells that express that gene divided by the total number of cells), and each grey dot represents an individual transcript. **(h)** Total versus bi-allelic percentage of detection of heterozygous SNVs in the same single cells as in (g) with respect to the total number of reads spanning that position (log2(coverage); x-axis). The blue line and grey points represent the total percentage of detection for a certain heterozygous position. The black line and red points indicate the detection of both alleles (at least 5% of reads mapping to either of the alleles).

Figure S2

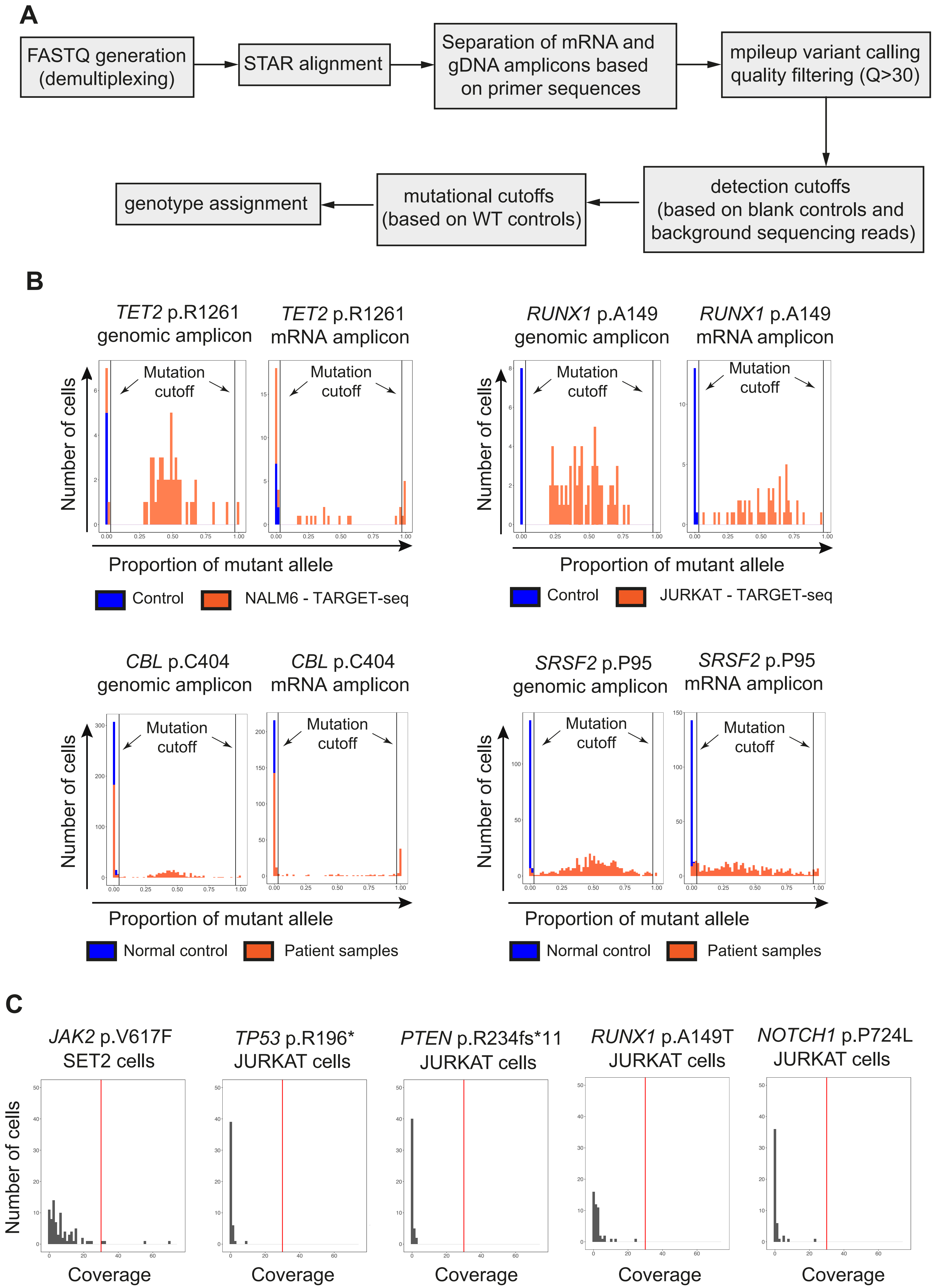

A
FASTQ generation (demultiplexing)
STAR alignment
Separation of mRNA and gDNA amplicons based on primer sequences
mpileup variant calling quality filtering (Q>30)
detection cutoffs (based on blank controls and background sequencing reads)
mutational cutoffs (based on WT controls)
genotype assignment
B
TET2 p.R1261 genomic amplicon
TET2 p.R1261 mRNA amplicon
RUNX1 p.A149 genomic amplicon
RUNX1 p.A149 mRNA amplicon
Mutation cutoff
Number of cells
Proportion of mutant allele
Control
NALM6 - TARGET-seq
JURKAT - TARGET-seq
CBL p.C404 genomic amplicon
CBL p.C404 mRNA amplicon
SRSF2 p.P95 genomic amplicon
SRSF2 p.P95 mRNA amplicon
Normal control
Patient samples
C
JAK2 p.V617F SET2 cells
TP53 p.R196* JURKAT cells
PTEN p.R234fs*11 JURKAT cells
RUNX1 p.A149T JURKAT cells
NOTCH1 p.P724L JURKAT cells
Coverage

Figure S2, related to Figure 1. Targeted pre-amplification and sequencing of mRNA and gDNA amplicons dramatically increases the sensitivity of mutation detection. (a) Schematic representation of the pipeline used for variant calling of targeted next generation sequencing. **(b)** Representative examples of variant allele frequencies and mutational cutoffs for gDNA and cDNA amplicons in *TET2* p.R1261 mutation in NALM6 cell line, *RUNX1* p.A149T mutation in JURKAT cell line, and *CBL* p.C404 and *SRSF2* p.P95 mutations in patient samples and normal donors. Black lines represent mutation cut-offs for each amplicon. **(c)** RNA-sequencing coverage of *JAK2* mutation in SET2 cells and *TP53, NOTCH1, RUNX1* and *PTEN* mutations in JURKAT cells. The y-axis represents the number of cells against their coverage for each mutation in the x-axis. Red line represents a coverage threshold of 30, used as minimum coverage for targeted sequencing experiments.

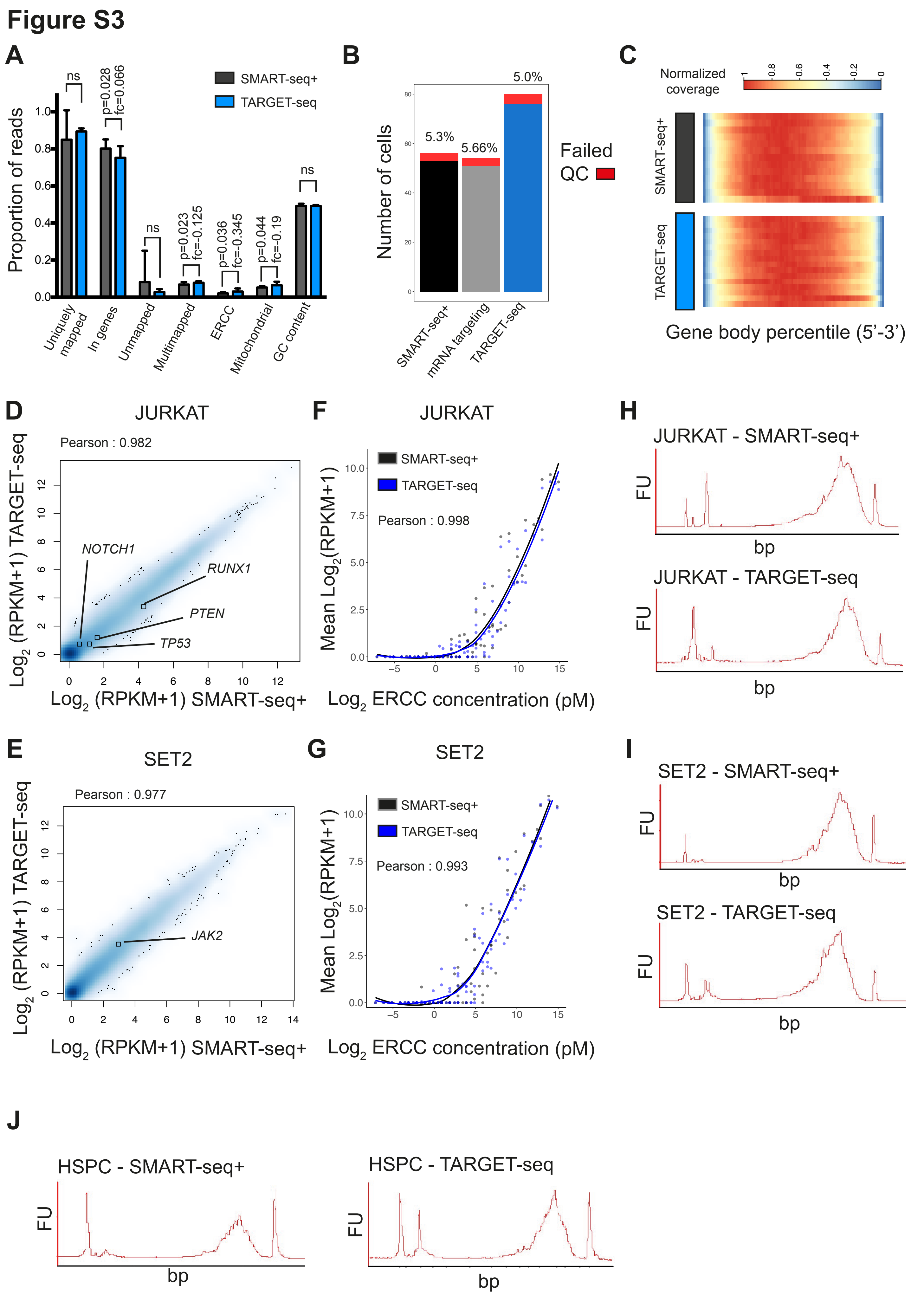

Figure S3
A
B
C
D
E
F
G
H
I
J
SMART-seq+
TARGET-seq
Proportion of reads
ns
p=0.028 fc=0.066
p=0.023 fc=-0.125
p=0.036 fc=-0.345
p=0.044 fc=-0.19
Uniquely mapped
In genes
Unmapped
Multimapped
ERCC
Mitochondrial
GC content
Number of cells
5.3%
5.66%
5.0%
Failed QC
mRNA targeting
Normalized coverage
Gene body percentile (5'-3')
JURKAT
Pearson : 0.982
NOTCH1
RUNX1
PTEN
TP53
Log2 (RPKM+1) TARGET-seq
Log2 (RPKM+1) SMART-seq+
Pearson : 0.998
Mean Log2(RPKM+1)
Log2 ERCC concentration (pM)
JURKAT - SMART-seq+
JURKAT - TARGET-seq
FU
bp
SET2
Pearson : 0.977
JAK2
Pearson : 0.993
SET2 - SMART-seq+
SET2 - TARGET-seq
HSPC - SMART-seq+
HSPC - TARGET-seq

Figure S3, related to Figure 2. Unbiased whole transcriptome analysis of single cells using TARGET-seq. **(a)** Sequencing statistics of single cell libraries from HSPCs processed using SMART-seq+ or TARGET-seq. The bar graph represents the proportion of reads for each sequencing statistic and condition, and error bars represent standard deviation of the mean. P-values from two-tailed Student's t-test and fold change values for each sequencing statistic are shown on the top of each pair of bars. **(b)** Number of cells passing or failing QC (Quality Control) per method. The percentage of cells failing QC for each method is shown on the top of each bar. **(c)** Normalized transcript coverage from single HSPCs processed using SMART-seq+ or TARGET-seq methods, using 4040 housekeeping genes. **(d,e)** Whole transcriptome Pearson's correlation between SMART-seq+ and TARGET-seq ensembles (mean RPKM values per condition) in JURKAT **(d)** and SET2 cells **(e)**. The expression values for the genes targeted are highlighted in each cell type. **(f,g)** Pearson's correlation between mean ERCC spike-in expression values from SMART-seq+ and TARGET-seq in JURKAT cells **(f)** and SET2 cells **(g)** per each ERCC spike-in concentration. **(h-j)** Bioanalyzer traces of representative cDNA libraries synthetized using SMART-seq+ or TARGET-seq in JURKAT **(h)**, SET2 **(i)** or HSPCs **(j)**.

Figure S4

A HD7643

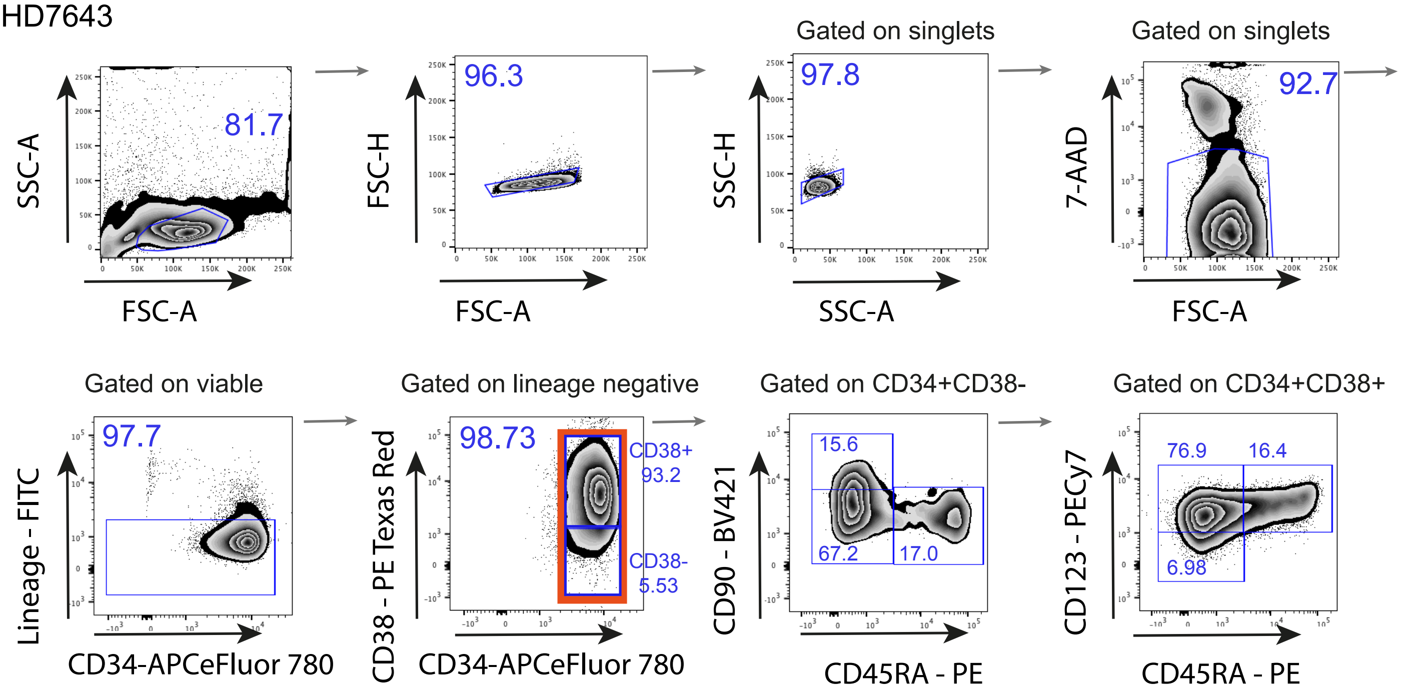

B OX2123

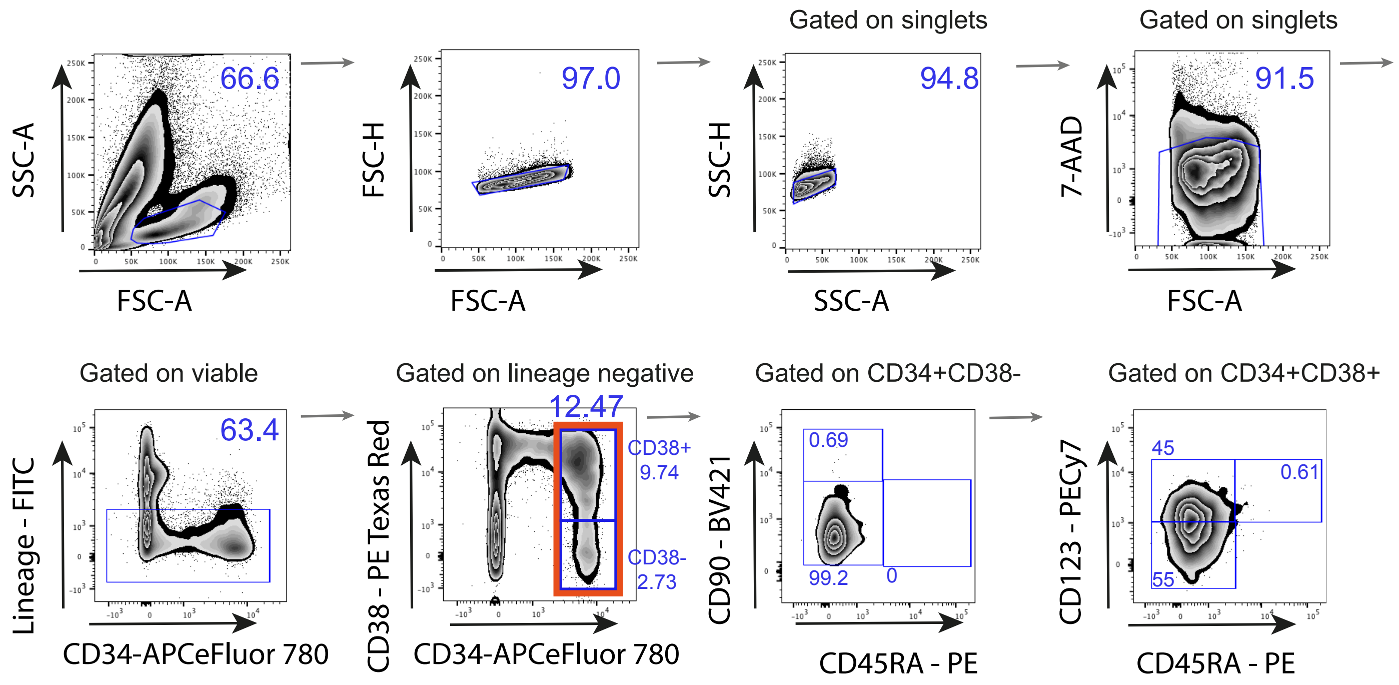

Figure S4, related to Figures 3 and 4. **Schematic representation of gating and sorting strategy. (a-b)** Schematic representation of gating and sorting strategy for a CD34+ selected healthy donor sample (a; HD7643) or patient sample (b; OX2123). Orange square represents sorting gate. Numbers represent percentage of gated cells. Antibodies used for HSPC isolation are listed in Key Resources Table and STAR Methods.

Figure S5

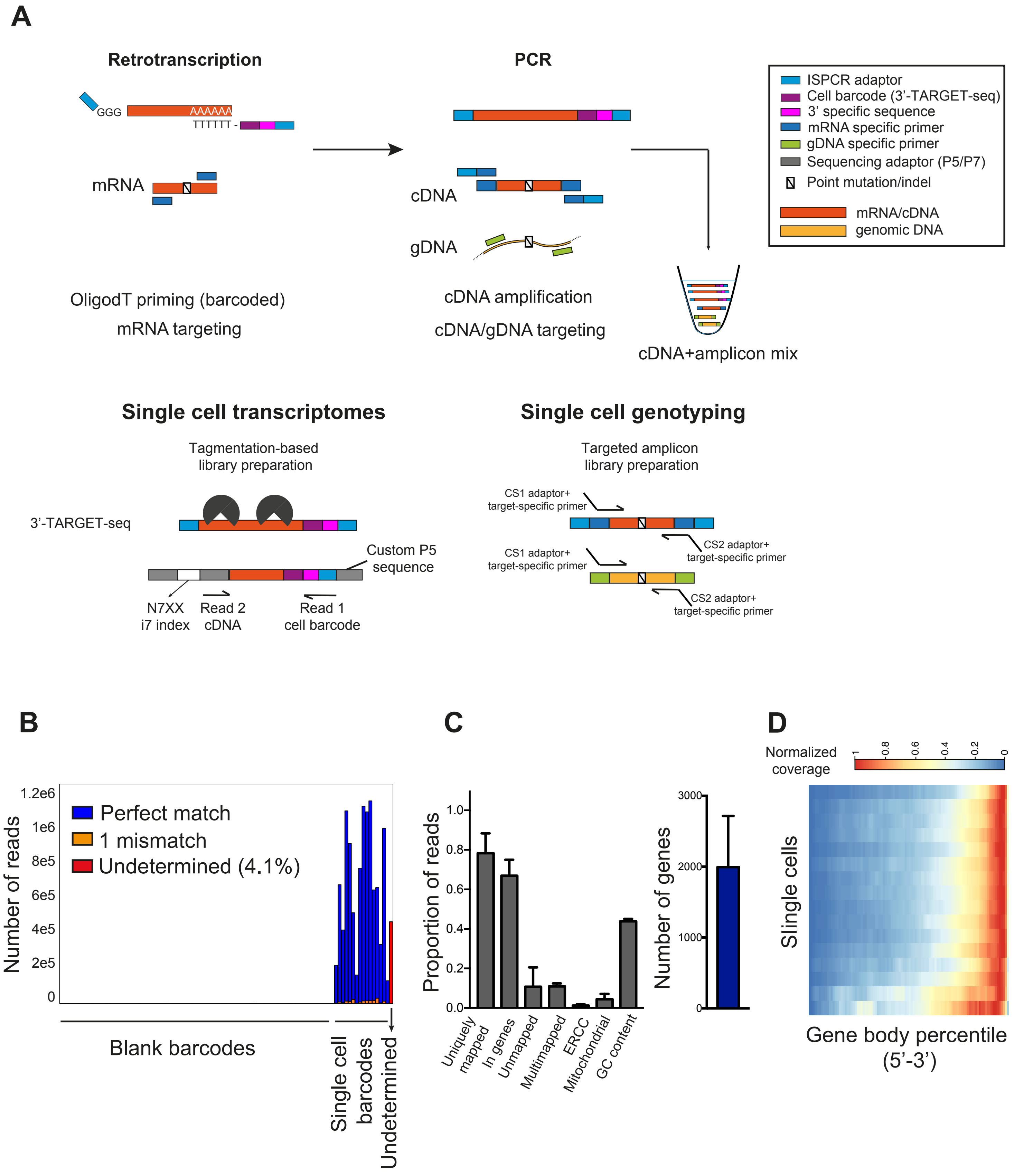
A
Retrotranscription
GGG
AAAAAA
TTTTTT -
mRNA
OligodT priming (barcoded)
mRNA targeting
PCR
cDNA
gDNA
cDNA amplification
cDNA/gDNA targeting
cDNA+amplicon mix
ISPCR adaptor
Cell barcode (3'-TARGET-seq)
3' specific sequence
mRNA specific primer
gDNA specific primer
Sequencing adaptor (P5/P7)
Point mutation/indel
mRNA/cDNA
genomic DNA
Single cell transcriptomes
Tagmentation-based library preparation
3'-TARGET-seq
Custom P5 sequence
N7XX i7 index
Read 2 cDNA
Read 1 cell barcode
Single cell genotyping
Targeted amplicon library preparation
CS1 adaptor+ target-specific primer
CS2 adaptor+ target-specific primer
CS1 adaptor+ target-specific primer
CS2 adaptor+ target-specific primer
B
Number of reads
1.2e6
1e6
8e5
6e5
4e5
2e5
0
Perfect match
1 mismatch
Undetermined (4.1%)
Blank barcodes
Single cell barcodes
Undetermined
C
Proportion of reads
1.0
0.8
0.6
0.4
0.2
0.0
Uniquely mapped
In genes
Unmapped
Multimapped
ERCC
Mitochondrial
GC content
Number of genes
3000
2000
1000
0
D
Normalized coverage
1
0.8
0.6
0.4
0.2
0
Slingle cells
Gene body percentile (5'-3')

Figure S5, related to Figures 5 and 6. Validation of high throughput 3'-TARGET-seq. (a) Schematic representation of 3'-TARGET-seq method. Briefly, a barcoded oligodT-ISPCR primer was used to prime polyadenylated mRNA molecules from each single cell; a 3'-specific sequence is also added to preferentially enrich for fragments containing the 3'-end of the molecule in tagmentation-based library preparation and 3'-biased sequencing. **(b)** Detection of cellular barcodes using 3'-TARGETseq in 16 HSPCs. Blue bars represent total number of reads mapping to cellular barcodes used for cDNA synthesis of HSPCs (16 barcodes); blank barcodes represent those not used for cDNA synthesis (80 barcodes); red bar represents the total number of reads from cell barcodes that do not match any of the 96 available cell barcodes. **(c)** Sequencing statistics of 3'-TARGET-seq libraries from the same 16 HSPCs as in (b) and number of genes detected per cell. Bars represents the proportion of reads for each sequencing statistic (left panel) or number of genes detected per cell (normalized counts≥1, right panel); error bars represent standard deviation of the mean. "GC" refers to guanine-cytosine content. **(d)** Normalized transcript coverage across 4040 housekeeping genes for 16 single HSPCs processed using 3'-TARGET-seq, showing expected 3' bias.

Figure S6

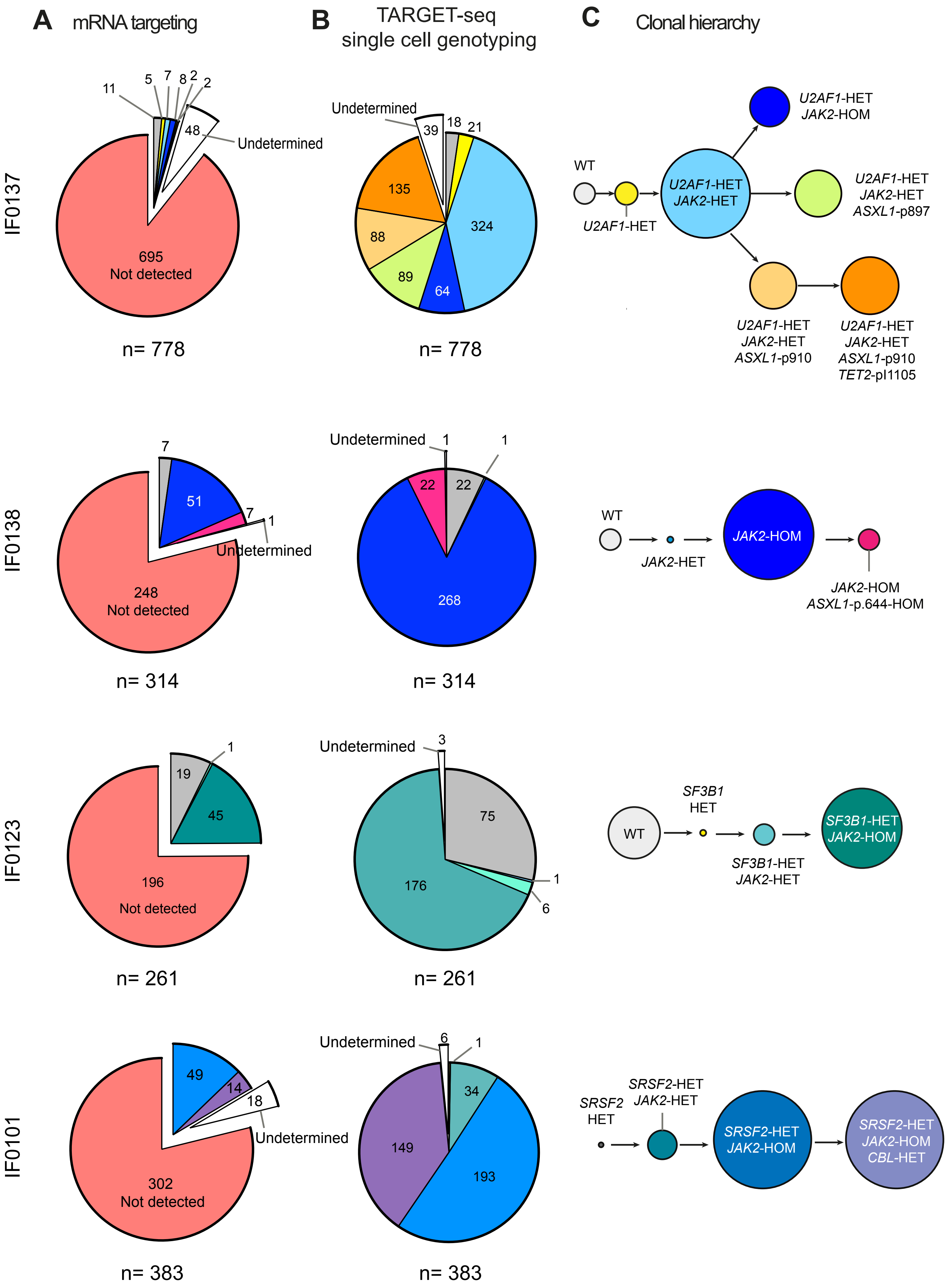
A
mRNA targeting
B
TARGET-seq
single cell genotyping
C
Clonal hierarchy
IF0137
11
5
7
8
2
2
48
Undetermined
695
Not detected
n= 778
Undetermined
39
18
21
135
88
89
64
324
n= 778
WT
U2AF1-HET
U2AF1-HET
JAK2-HET
U2AF1-HET
JAK2-HOM
U2AF1-HET
JAK2-HET
ASXL1-p897
U2AF1-HET
JAK2-HET
ASXL1-p910
U2AF1-HET
JAK2-HET
ASXL1-p910
TET2-pI1105
IF0138
7
51
7
1
Undetermined
248
Not detected
n= 314
Undetermined
1
1
22
22
268
n= 314
WT
JAK2-HET
JAK2-HOM
JAK2-HOM
ASXL1-p.644-HOM
IF0123
19
1
45
196
Not detected
n= 261
Undetermined
3
75
176
1
6
n= 261
WT
SF3B1
HET
SF3B1-HET
JAK2-HET
SF3B1-HET
JAK2-HOM
IF0101
49
14
18
Undetermined
302
Not detected
n= 383
Undetermined
6
1
34
149
193
n= 383
SRSF2
HET
SRSF2-HET
JAK2-HET
SRSF2-HET
JAK2-HOM
SRSF2-HET
JAK2-HOM
CBL-HET

Figure S6, related to Figures 5 and 6. TARGET-seq reveals genetic subclones in the HSPC compartment from myelofibrosis patients that could not be inferred through bulk sequencing or by mRNA targeting alone. (a-b) Subclonal composition of indicated patients' HSPC compartment identified by single cell genotyping using (a) mutational information from targeted mRNA amplicons (mRNA targeting) or (b) TARGET-seq. Total number of cells identified per subclone is shown in each slice of the pie chart, and the total number of cells passing QC genotyping for each patient is shown below each chart. "Undetermined" cells (those not fitting in the clonal hierarchy determined by SCITE) are coloured in white; "ND" (Not Detected; coloured in red) represents cells in which at least one of the amplicons was not detected. Each patient is labelled according to the code provided in Table S3. **(c)** Clonal hierarchies identified by SCITE for each patient. Each subclone in (a-b) is color-coded according to the clonal tree presented in (c). A full list of genetic subclones identified for all patients can be found in Table S3b. The size of circles in the clonal tree represents the relative fraction of detected cells according to (b).

Figure S7

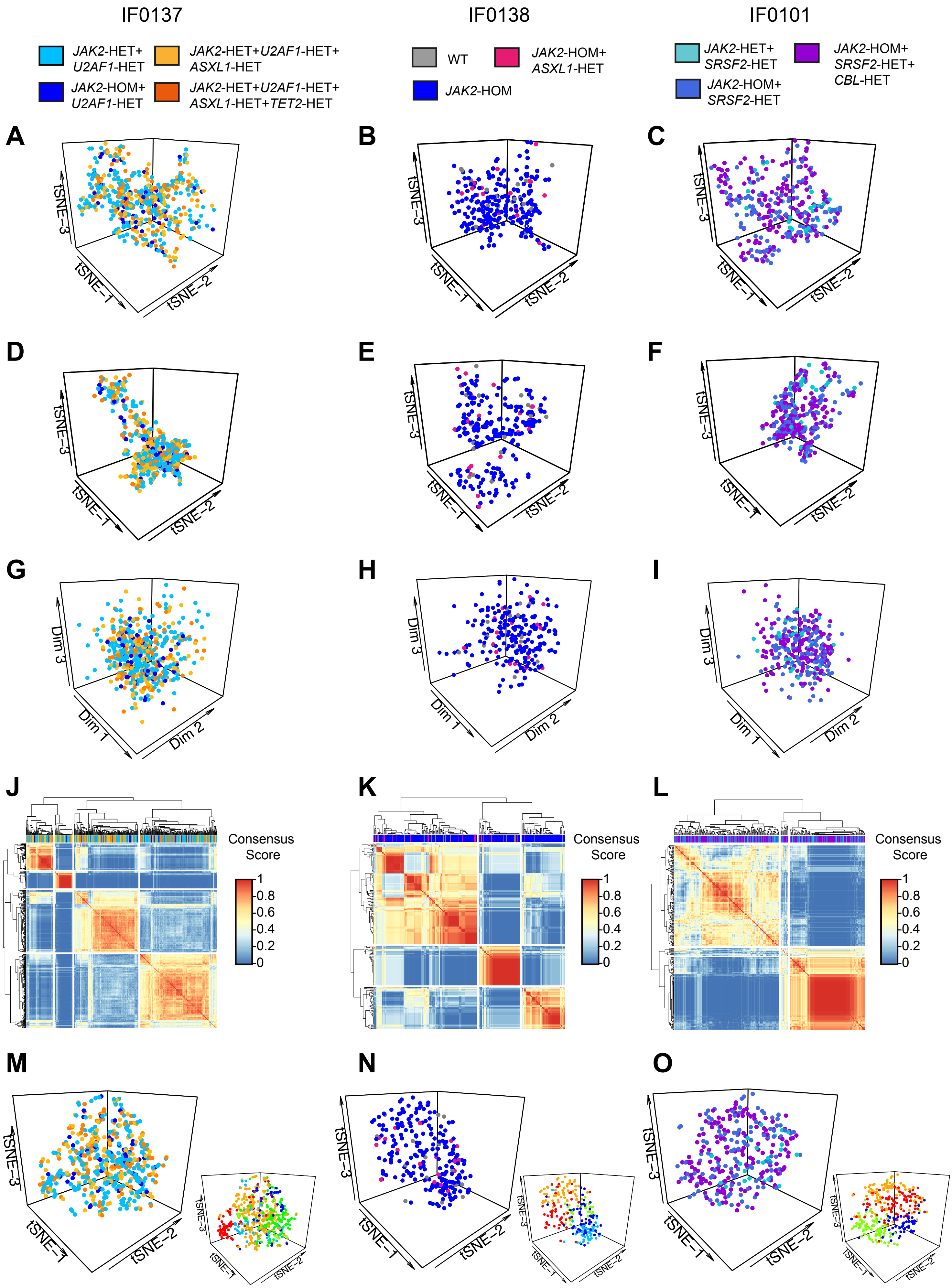
IF0137
IF0138
IF0101
JAK2-HET+ U2AF1-HET
JAK2-HET+U2AF1-HET+ ASXL1-HET
JAK2-HOM+ U2AF1-HET
JAK2-HET+U2AF1-HET+ ASXL1-HET+TET2-HET
WT
JAK2-HOM+ ASXL1-HET
JAK2-HOM
JAK2-HET+ SRSF2-HET
JAK2-HOM+ SRSF2-HET+ CBL-HET
JAK2-HOM+ SRSF2-HET
A
B
C
D
E
F
G
H
I
J
K
L
M
N
O
tSNE-1
tSNE-2
tSNE-3
Dim 1
Dim 2
Dim 3
Consensus Score
1
0.8
0.6
0.4
0.2
0

Figure S7, related to Figure 6. **Computational analysis of scRNA-seq data does not distinguish genetically distinct subclones of HSPCs within individual myelofibrosis patients**. **(a-c)** tSNE representation of 555 cells from patient IF0137 (a), 243 cells from patient IF0138 (b) and 320 cells from patient IF0101 (c) using 3031 (a), 2605 (b) and 3023 (c) highly variable genes. Gene expression matrices were batch corrected and genotypes were preserved. Cells are colored according to each genotype group for each patient. **(d-f)** tSNE representation from the same cells and patients as in (a-c), using highly variable genes and regressing out the effect of the cell cycle. Gene expression matrices were batch corrected and genotypes were preserved. **(g-i)** ZIFA dimensionality reduction from the same single cells and patients as in (a-c) using 3031 (g), 2605 (h) or 3023 (i) highly variable genes. **(j-l)** SC3 K-means clustering clustering from the same single cells and patients as in (a-c) using k=4 for patient IF0137 (j), k=3 for patient IF0138 (k) and k=3 for patient IF0101 (l). Heatmaps are coloured according to the consensus score computed by SC3. **(m-o)** tSNE representation of clusters identified by PAGODA2 from the same single cells and patients as in (a-c), coloured by genotype (left panel) or by clusters identified by PAGODA2 (right panel). We identified 7 clusters in patient IF0137 (m), 5 clusters in patient IF0138 (n) and 4 clusters in patient IF0101 (o). Gene expression matrices were batch corrected using the batchNorm function (method='glm').

SUPPLEMENTAL EXPERIMENTAL PROCEDURES

Detailed step by step TARGET-seq protocols and primer design and validation technical note, related to Figure 1 and Figure S5.

Full-length TARGET-seq protocol in 96 well plates

Materials

- 96-well PCR plates (Thermo Fisher Scientific, AB-0900)
- 384 well PCR plates (FrameStar, 4titude, 4ti-0384/C)
- Corning® 96 Well TC-Treated Microplates (Cat. No. CLS3595-50EA)
- RNase Free Microfuge Tubes (Invitrogen, AM12400)
- V-shaped 96 well plate (AXYGEN, P-96-450V-C, 500 uL 96 well "V" bottom clear; Ref. 391-02-501)
- PCR film (Thermo Fisher Scientific, MicroAMp Clear Adhesive Film, Cat. No. 4306311)
- Aluminium Sealing Film (StarLab, E2796-0792)
- 96-well magnetic stand (Invitrogen, AM10027)
- Protease (Qiagen, Cat#19155)
- Triton X-100 (Sigma-Aldrich, Cat#T8787)
- RNase inhibitor (TAKARA, Cat#2313A)
- dNTPs (Life Technologies, Cat#R0192)
- UltraPure DNAse/RNAse-Free Distilled Water (Life Technologies, Cat#10977035)
- EB Buffer (Qiagen, Cat No./ID: 19086)
- Ethanol
- RNAse-free TE buffer (Invitrogen, Cat#AM9849)
- ERCC aliquot (Ambion, Cat#4456740)
- SMARTScribe enzyme (Clontech - Cat. No. 639537).
- SeqAMP enzyme (Clontech, Cat#638509).
- RT-PCR Grade Water (Life Technologies, Cat#AM9935).
- Ampure XP beads (Beckman Coulter, Cat#A63881)
- Pre-amplification primers: oligodT-ISPCR primer, mRNA target-specific primers, TSO-LNA; gDNA target-specific primers, cDNA target-specific primers and ISPCR primers. Keep in dedicated "pre-amplification" area only. Custom primers from Biomers.net (oligodT-ISPCR, mRNA/gDNA/cDNA target-specific primers, ISPCR primers; HPLC purification) and Qiagen (TSO-LNA; RNAse free HPLC purification).
- High Sensitivity NGS Fragment Analysis Kit (1bp - 6,000 bp; Agilent; Cat# DNF-474) or similar kit for capillary system (High Sensitivity D5000 ScreenTape System; Agilent, Cat# 5067- 5592 and Cat# 5067- 5593; or Agilent High Sensitivity DNA Kit to use with Agilent 2100 Bioanalyzer System, Cat#5067-4627 and Cat#5067-4626).
- PCR1 primers: CS1/CS2-target specific primers for gDNA and cDNA PCR1 barcoding (custom primers from Invitrogen; desalted).
- Sequencing primers for targeted genotyping libraries: CS1-seq, CS2-seq, CS1rc-seq, CS2rc-seq (custom LNA primers from Qiagen; HPLC purification).

- Nextera XT Kit Library Preparation Kit (Illumina , Cat#15032354) including i7 indexes and i5 indexes (Nextera XT Index Kit, Illumina , Cat#FC-131-1001).
- KAPA 2G Robust HS PCR Kit (Sigma Aldrich,Cat#KK5517)
- FastStart High Fidelity PCR System (Roche, REF:04738292001)
- Access Array™ Barcode Library for Illumina® Sequencers-384, Single Direction (Fluidigm, Cat#100-4876).
- Qubit (ThermoFisher, Cat. No. 32854)

Sorting and lysis – Timing: variable

1. First, prepare sufficient lysis buffer for the required number of cells for each experiment, plus 10% dead volume. Aliquot the lysis buffer (containing oligodT-ISPCR primer) into each well of a 96-well PCR plate (Thermo Scientific #AB-0900) in a clean environment dedicated to 'pre-amplification' work only. Cover with a PCR film and keep on ice/in the fridge until use. Lysis buffer should be prepared fresh on the day of sorting, maximum a few hours before use.

Lysis	1 cell	Storage	Cat. No./Supplier
Triton 0.4%	1.9 µL	-20 °C	Sigma-Aldrich # T8787, resuspended in DNAse/RNAse-Free water
RNAse Inhibitor	0.1 µL	-20 °C	TAKARA #2313A
dNTPs (10 mM)	1 µL	-20 °C	Life Technologies #R0192
Oligo-dT-ISPCR (10 µM)	1 µL	-20 °C	Biomers (custom oligo, HPLC purified)
Protease (1.09 AU/mL in water)	0.1 µL	+4 °C	Qiagen #19155; resuspend in UltraPure DNAse/RNAse-Free Distilled Water (Life Tech, #10977035)
ERCC RNA spike-in mix (1:2e6)	0.1 µL	-80 °C(single use aliquot)	Ambion #4456740
**TOTAL**	4.2 µL		

2. Prepare the sorter for single-cell sorting. Use single cell purity mode and keep the event rate low (less than 1000/s).

3. Check sorter alignment: use a 96 well tissue culture flat-bottom plate (Corning) and sort one fluorescent bead per well. Check under a fluorescent microscope that there is only one bead per well and that the position of the bead is centered. Note: don't add any media into the plate so that the bead stays in the place it was deposited by the sorter.

4. Use a 96-well PCR plate (Thermo Scientific #AB-0900, same model as the one in which cells will be sorted) covered with a PCR film, and sort 50 cells in positions 1A, 1H, 12A and 12H. Droplets should be positioned in the centre of the wells if the sorter is correctly aligned; if not, make necessary adjustments until drops are falling perfectly into each well.

5. After this initial check, remove the PCR film from the 96 PCR plate and sort 50 cells into columns 1 and 12 (wells 1A, 1B, 1C, 1D, 1E, 1F, 1G, 1H and 12A, 12B, 12C, 12D, 12E, 12F, 12G, 12H): drops should now be deposited at the very bottom of each well with no traces of liquid been left in the sides of each well. If correct, alignment checks are now complete.

6. Perform a purity sort of desired populations.

7. Sort cells directly into a 96 well PCR plate (Thermo Scientific #AB-0900) containing the lysis buffer, cover the plate with an aluminium PCR film (StarLab), spin down the plate and incubate 5 minutes at room temperature to allow for protease digestion. If sorting time is longer than 10 minutes, there is no need to incubate the plate further.

8. Put the plate directly into dry ice and store at -80 °C up to 1-2 months. (Processing plates after 3 months of -80 °C storage has shown decreased yield and/or signs of RNA degradation).

Heat inactivation, cDNA synthesis and amplification (RTPCR) – Timing: 6.5 hours; 1.5 hours hands-on time

9. Transport the plate(s) and TSO-LNA aliquot from -80 C storage on dry ice to a 'pre-amplification' dedicated workspace/clean room.

10. Thaw the 5X Buffer, RT-PCR Grade Water and any mRNA targeting primers that you might add to the mix. These can be thawed at room temperature. Aliquot them into an RNase free tube to prepare a master mix for the retrotranscription (RT) step as per the table below. RNase inhibitor, TSO-LNA and SMARTScribe enzyme will be added to the mix during heat inactivation step.

RT	1 cell (µL)	Storage	Cat. No.
**Buffer 5X**	2.00 µL	-20 °C	Clontech - Cat. No. 639537 (delivered with enzyme)
**RNase Inhibitor** (wait until the 72C step to add it)	0.25 µL	-20 °C	TAKARA – Cat#2313A
**TSO-LNA (100 µM)** (wait until 72C step to add it)	0.10 µL	-80 °C (aliquot into single use aliquots to avoid freeze/thaw cycles)	Custom TSO-LNA oligo from Exiqon-Qiagen (same as Picelli et al., 2013)
**RT-PCR Grade Water**	Variable	-20 °C	Life Tech - AM9935
**mRNA primers** (0.035 µL of each primer from a 200 uM stock)	Variable	-20 °C	Custom HPLC purified primers from biomers.net; resuspend in RNAse Free TE/water
**SMARTScribe** (wait until 72C step to add it)	1.00 µL	-20 °C	Clontech - Cat. No. 639537

**TOTAL**	5.60 µL		
**TOTAL (cumulative)**	9.80 µL		

11. Incubate the sample plate 15 minutes at 72 °C in a thermocycler (no RT mix has been added at this point). This step will inactivate the protease included in the lysis buffer so it doesn't interfere with any subsequent enzymatic steps.

12. During the heat inactivation time, add the RNAse Inhibitor, TSO-LNA and RT enzyme to the RT master mix on ice/cold block. Vortex and spin down.

13. Once the heat inactivation step is finished, take the plate out of the thermocycler, spin down and place into ice/cold rack. Aliquot 5.6 µL of RT mix into each well and carefully seal the plate with a PCR film (MicroAMp Clear Adhesive Film, Cat. No. 4306311). Note: it is essential that this step is performed within 5-7 minutes to avoid RNA degradation.

14. Spin down and run the following program in a thermocycler:

Temperature	Time	Cycles
42 C	90 min	1
50 C	2 min	10 cycles
42 C	2 min	
70 C	15 min	1
4 C	HOLD	-

15. Fifteen minutes before the RT program finishes, start thawing reagents to prepare the PCR master mix.

PCR	1 cell (µL)	Storage	Cat. No.
**2X Buffer**	12.50 µL	-20 °C	638509 - Clontech (delivered with enzyme
**ISPCR (10 µM)**	0.125 µL	-20 °C	Custom HPLC oligo from biomers.net (same as Picelli et al., 2013)
**RT-PCR Water**	Variable	-20 °C	Life Tech - AM9935
**SeqAMP Enzyme -** wait until RT is about to finish to add	0.50 µL	-20 °C	638509 - Clontech
**cDNA primers -** (0.035 µL from each primer from 20 µM stock)	Variable	-20 °C	Custom HPLC purified primers from biomers.net; resuspend in RNAse Free TE/water
**Genomic primers** (0.1 µL from each primer from a 200 µM stock)	Variable	-20 °C	
**TOTAL**	15.00 µL		
**TOTAL (cumulative)**	24.80 µL		

16. Once the RT program is finished, spin down the plate and add PCR master mix on ice/cold rack. Spin down the plate at 1000 g for 15 seconds. Take outside of the clean room workspace, place in a thermocycler and run the following program:

Temperature	Time	Cycles
98 C	3 min	
98 C	00:15	22 cycles (single HSPCs)
67 C	00:20	
72 C	6 min	
72 C	5 min	
4 C	HOLD	

Bead clean-up – Timing: 45 minutes

17. Add 16 µL of beads (Ampure XP Beads, Beckman Coulter, Cat. No. 391-02-501) into a V-shaped 96 well plate (AXYGEN, P-96-450V-C, 500 uL 96 well "V" bottom clear; Ref. 391-02-501).

18. Aliquot 11 µL of clean water (PCR grade) into the same V-shaped 96 well plate.

19. Aliquot 14 µL of each cDNA+amplicon mix into each well of the same plate and pipette up and down to mix the cDNA+amplicon mix with beads (0.6:1 beads to cDNA ratio). Incubate for 5 minutes at room temperature.

20. Incubate mixture on a 96-well magnetic stand for 2 minutes. Once the liquid is clear of beads, remove the liquid.

21. Wash the beads twice with 80 % EtOH (freshly prepared, dilute EtOH in PCR grade water). Add 100 µL of ethanol to each well, incubate for 30 seconds and remove. Repeat once more (2 times in total) and use P10 tips to remove any remaining ethanol.

22. Leave the beads to air-dry for 3 minutes. Be careful not to overdry the beads at this point or it will be difficult to resuspend them.

23. Resuspend the beads into 8 µL of EB Buffer (Qiagen Cat No./ID: 19086) with the plate off the magnet. Incubate for 30 seconds and put the plate back onto the magnet. Incubate into the magnet until the liquid is clear, then transfer 7.5 µL of purified cDNA library to a new plate for -20 C storage or further processing.

24. Check cDNA traces quality and size distributions using Bioanalyzer (Agilent), Fragment Analyzer Automated CE System (Advanced Analytical) or similar capillary system. Representative good quality cDNA traces are shown below (Figure MS1).

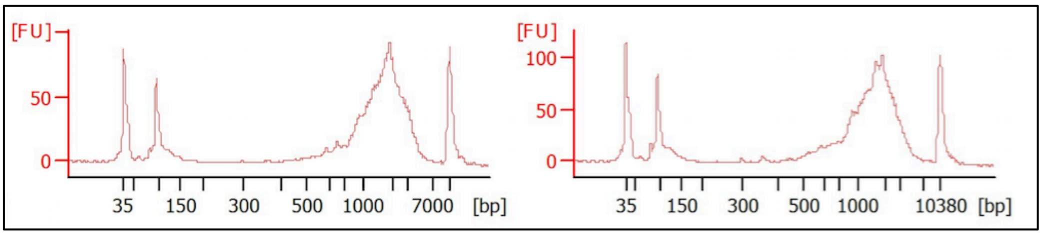

Figure MS1. Representative bead-purified cDNA traces from single HSPCs synthesized using full-length TARGET-seq in 96-well plates.

Whole transcriptome library preparation – Timing: 2 hours – 1.5 hours hands on time

25. Library preparation is performed using a commercially available Nextera XT Kit (FC-131-1096, Illumina) and commercially available i5 and i7 indeces (Nextera XT Index Kit, FC-131-1001, Illumina) using one fourth of the recommended volume. First, add 2.5 µL of Tagmentation Buffer into the required number of wells in a 96-well or 384-well plate (one well will be used for each cell).

26. Add 700 pg of bead-purified cDNA from each pool in a total volume of 1.25 µL and 1.25 µL of Amplicon Tagmentation Mix (ATM). Incubate 6 minutes at 55 C (total volume 5 µL).

Reagents	1 reaction (µL)
Tagmentation Buffer	2.5 µL
Bead purified cDNA (560 pg/µL)	1.25 µL
ATM (Amplicon Tagment Mix)	1.25 µL
**TOTAL**	5 µL

27. Once the incubation is finished, add 1.25 µL of NT (Neutralization) buffer to neutralize the tagmentation reaction.

28. Prepare PCR master mix as outlined below.

Reagents	1 reaction (µL)
i7 index (2 µM)	1.25 µL
i5 index (2 µM)	1.25 µL
NPM (PCR master mix)	3.75 µL
**TOTAL**	6.25 µL
**TOTAL (cumulative)**	12.5 µL

29. Incubate in a thermocycler and run the following PCR program:

Temperature	Time	Cycles
72 C	3 minutes	1
95 C	30 seconds	1
95 C	10 seconds	14 cycles

55 C	30 seconds	
72 C	30 seconds	
72 C	5 minutes	1
4 C	HOLD	1

30. Bead-purify barcoded and tagmented Nextera XT libraries using Ampure XP beads. First, dilute the product 1:1 with 12.5 μL of PCR-grade water. Aliquot each barcoded and tagmented library into a V-shaped 96 well plate (Cat. No. P-96-450V-C, Axygen) and aliquot 16 μL of pre-warmed (room temperature) Ampure XP beads (Beckman Coulter; Cat. No. A63881) into each well (0.6:1 beads to cDNA ratio). Incubate for 5 minutes at room temperature.

31. Incubate mixture into a 96-well magnetic stand for 2 minutes. Once the liquid is clear of beads, remove the liquid.

32. Wash the beads twice with 80 % EtOH (freshly prepared, dilute EtOH in PCR grade water). Add 100 μL of ethanol to each well, incubate for 30 seconds and remove. Repeat once more (2 times in total) and use P10 tips to remove any remaining ethanol.

33. Leave the beads to air-dry for 3 minutes. Be careful not to overdry the beads at this point or it will be difficult to resuspend them.

34. Resuspend the beads into 21 μL of EB Buffer (Qiagen Cat No./ID: 19086) with the plate off the magnet. Incubate for 30 seconds and put the plate back onto the magnet.

35. Incubate on the magnet until the liquid is clear of beads, then transfer 20 μL of purified tagmented/barcoded library to a new plate for -20 °C storage or further processing.

36. Run libraries on D5000 TapeStation or similar capillary array. Library fragments should be from 300 bp to 800 bp on average (Figure MS2):

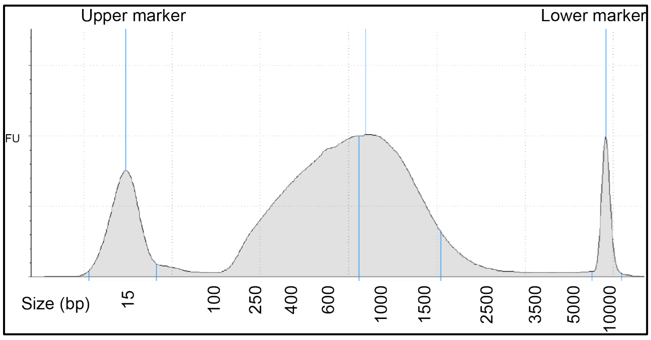

Figure MS2. Representative traces of tagmented, amplified and bead-purified full-length Nextera XT libraries.

37. Quantify tagmented and barcoded libraries using Qubit (ThermoFisher, Cat. No. 32854) and pool equimolar concentrations of each library. Quantify the final pool and sequence on a NextSeq/HiSeq platform.

Single cell genotyping library preparation for NGS – Timing: 5 hours – 2 hours hands on time

38. Take one aliquot of the unpurified cDNA+amplicon mix, dilute 1:2 with PCR Grade water and use as an input for the first barcoding PCR (PCR1). Perform an individual PCR reaction for each sample in a 384 well-plate (FrameStar 384, Cat. No. 4ti-0384/C). During this PCR reaction, target-specific primers attached to universal tags (CS1/CS2 adaptors) will be added to each amplicon from each sample, in order to prepare a targeted sequencing library. Targets with similar amplification efficiencies might be amplified simultaneously in the same reaction for the same single cell. Note: gDNA and cDNA pre-amplified amplicons don't have a cell-specific barcode at this stage; therefore, amplicons corresponding to each cell should be kept in individual wells of the 384 well-plate, taking precautions to avoid cross-well contamination.

39. Prepare PCR1 Mix and aliquot in the 384 well-plate using a Biomek FxP Liquid Handler (Beckman Coulter) of similar liquid handling platform:

**PCR1 BARCODING with target-specific primers**	**1 Reaction**	**Storage**	**Cat. No.**
KAPA 2G Ready Mix	3.125 µL	-20 °C	KAPA 2G Robust HS PCR Kit #KK5517
Primer F1+R1 (20 µM)	0.375 µL	-20 °C	Custom primers (Invitrogen) desalted, resuspend in TE
Primer F2+R2 (20 µM)	0.375 µL	-20 °C	
Primer F3+R3 (20 µM)	0.375 µL	-20 °C	
Primer FX+RX…	…	…	
RT-PCR Grade Water	Variable	-20 °C	UltraPure DNAse/RNAse-Free Distilled Water, (Life Technologies, #10977035)
cDNA aliquot	1.5 µL	-20 °C	
**TOTAL**	6.25 µL		

40. Incubate in a thermocycler and run the following PCR program:

**PCR1 PROGRAM**		
Temperature	Time (min:sec)	Cycles
95 C	03:00	1
95 C	00:15	20
60 C	00:20	
72 C	01:00	
72 C	05:00	1
4 C	HOLD	

41. Use 2.5 µL of PCR1 product as an input for the next reaction (PCR2). During this step, sample-specific barcodes are attached to previously tagged amplicons using the

Access Array™ Barcode Library for Illumina® Sequencers (384, Single Direction, Fluidigm). Barcode each sample in individual reactions.

42. Aliquot the barcodes (Access Array™ Barcode Library for Illumina® Sequencers) into a 384 well plate, and aliquot the PCR1 product into the same plate using a Biomek FxP Liquid Handler (Beckman Coulter) of similar liquid handling platform.

43. Prepare the PCR2 master mix and aliquot:

**PCR2 BARCODING with Illumina compatible primers**	**1 Reaction**	**Storage**	**Cat. No.**
FastStart High Fidelity 10X Reaction Buffer	1 µL	-20 °C	FastStart High Fidelity PCR System REF:04738292001
MgCl2 (25 mM)	1.8 µL	-20 °C	
DMSO	0.5 µL	-20 °C	
dNTP Mix (10 mM)	0.2 µL	-20 °C	
FastStart High Fidelity Enzyme (5U/µL)	0.1 µL	-20 °C	
RT-PCR Grade Water	1.90 µL	-20 °C	UltraPure DNAse/RNAse-Free Distilled Water, (Life Technologies, #10977035)
Single-direction barcodes (2 µM, Fluidigm)	2.0 µL	-20 °C	Access Array™ Barcode Library for Illumina® Sequencers-384, Single Direction, Fluidigm (Cat. No. 100-4876)
PCR1 barcoding aliquot	2.5 µL	-20 °C	
**TOTAL**	10 µL		

44. Incubate in a thermocycler and run the following PCR program:

**PCR2 PROGRAM**		
Temperature	Time (min:sec)	Cycles
95 C	10:00	1
95 C	00:15	10
60 C	00:30	
72 C	01:00	
72 C	03:00	1
4 C	HOLD	

45. Pool amplicons from each barcoded library using a liquid handling platform and use Ampure XP beads to clean-up pooled libraries (0.8:1 beads to cDNA ratio). Quantify

libraries using Qubit (ThermoFisher; Cat No. 32854) and check library size distribution and specific amplification of targeted amplicons on D1000 TapeStation or similar capillary array (Figure MS3). Note: barcodes and adaptors add 103 bp extra to the original PCR product.

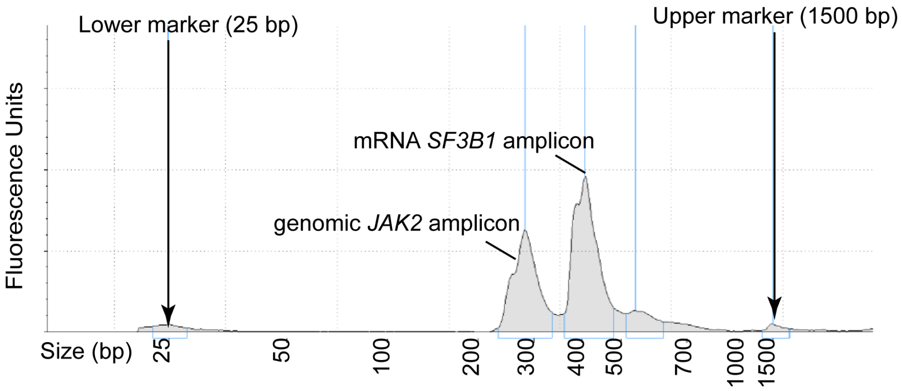

Figure MS3. Representative distributions of targeted amplicon libraries from genomic *JAK2* and mRNA *SF3B1* amplicons in a multiplexed reaction.

46. Libraries are ready for sequencing using custom sequencing primers targeted to CS1/CS2 tags (500 nM of CS1-seq and CS2-seq primers in a total volume of 700 µL for R1 and R2; 500 nM of CS1rc-seq and CS2rc-seq primers in a total volume of 700 µL for Index Read when using the MiSeq platform, Illumina). Note: CS1/CS2 and CS1rc/CS2rc sequencing primers contain LNA modifications (see Key Resources), as compared to CS1/CS2 tags used for PCR1 target-specific primers.

3’-TARGET-seq protocol in 384 well plates

Materials

- 96-well PCR plates (Thermo Fisher Scientific, AB-0900)
- 384 well PCR plates (FrameStar, 4titude, 4ti-0384/C)
- 384 well microplate (Corning® , CLS3702-100EA)
- RNase Free Microfuge Tubes (Invitrogen, AM12400)
- V-shaped 96 well plate (AXYGEN, P-96-450V-C, 500 uL 96 well "V" bottom clear; Ref. 391-02-501)
- PCR film (Thermo Fisher Scientific, MicroAMp Clear Adhesive Film, Cat. No. 4306311)
- Aluminium Sealing Film (StarLab, E2796-0792)
- 96-well magnetic stand (Invitrogen, AM10027)
- Protease (Qiagen, Cat#19155)
- Triton X-100 (Sigma-Aldrich, Cat#T8787)
- RNase inhibitor (TAKARA, Cat#2313A)
- dNTPs (Life Technologies, Cat#R0192)
- UltraPure DNAse/RNAse-Free Distilled Water (Life Technologies, Cat#10977035)
- EB Buffer (Qiagen, Cat No./ID: 19086)
- RNAse-free TE buffer (Invitrogen, Cat#AM9849)
- Ethanol
- ERCC aliquot (Ambion, Cat#4456740)
- SMARTScribe enzyme (Clontech - Cat. No. 639537).
- SeqAMP enzyme (Clontech, Cat#638509).
- RT-PCR Grade Water (Life Technologies, Cat#AM9935).
- Ampure XP beads (Beckman Coulter, Cat#A63881)
- Pre-amplification primers: oligodT-ISPCR barcoded primers, mRNA target-specific primers, TSO-LNA; gDNA target-specific primers, cDNA target-specific primers and ISPCR primers. Keep in dedicated “pre-amplification” area only. Custom primers from Biomers.net (oligodT-ISPCR barcoded primers, mRNA/gDNA/cDNA target-specific primers, ISPCR primers; HPLC purification) and Qiagen (TSO-LNA; RNAse free HPLC purification).
- PCR1 primers: CS1/CS2-target specific primers for gDNA and cDNA PCR1 barcoding (custom primers from Invitrogen; desalted).
- High Sensitivity NGS Fragment Analysis Kit (1bp - 6,000 bp; Agilent; Cat# DNF-474) or similar kit for capillary system (High Sensitivity D5000 ScreenTape System; Agilent, Cat# 5067- 5592 and Cat# 5067- 5593; or Agilent High Sensitivity DNA Kit to use with Agilent 2100 Bioanalyzer System, Cat#5067-4627, Cat#5067-4626).
- P5_index primer (custom oligonucleotide from Biomers.net; HPLC purified, contains PTO modifications; See Key Resources)
- Sequencing primers for targeted genotyping libraries: CS1-seq, CS2-seq, CS1rc-seq, CS2rc-seq (custom LNA primers from Qiagen; HPLC purification; See Key Resources).
- Sequencing primer: P5_SEQ (custom oligonucleotide from Biomers.net; PAGE purified, contains PTO modifications; See Key Resources).
- Nextera XT Kit Library Preparation Kit (Cat. No.15032354, Illumina) including i7 indexes (Illumina, Cat#FC-131-1001; alternatively, custom i7 primers can be used)

- KAPA 2G Robust HS PCR Kit (Sigma Aldrich,Cat#KK5517)
- FastStart High Fidelity PCR System (Roche, REF:04738292001)
- Access Array™ Barcode Library for Illumina® Sequencers-384, Single Direction (Fluidigm, Cat#100-4876).
- Qubit (ThermoFisher, Cat. No. 32854)

Sorting and lysis – Timing: variable

1. First, prepare a lysis buffer+oligodT stock plate. Sufficient lysis buffer (without barcoded oligo-dT) should be calculated to account for the required number of cells for each experiment plus 15% dead volume, and aliquoted into a 96-well PCR plate (Thermo Scientific #AB-0900) in a clean environment dedicated to 'preamplification' work only. Keep the lysis buffer on ice/in a cold rack. Lysis buffer should be prepared fresh on the day of sorting, maximum a few hours before use.

2. Aliquot each barcoded oligodT-ISPCR primer in each well of the same 96-well PCR plate (containing lysis buffer), using a liquid handling platform. The amount of barcoded-oligodT in each well is calculated as follows: (number of cells to be processed/96)*0.575 µL; i.e. if processing a total of 3000 cells, aliquot (3000/96)*0.575 µL of barcoded oligodT following the layout below (Figure MS4):

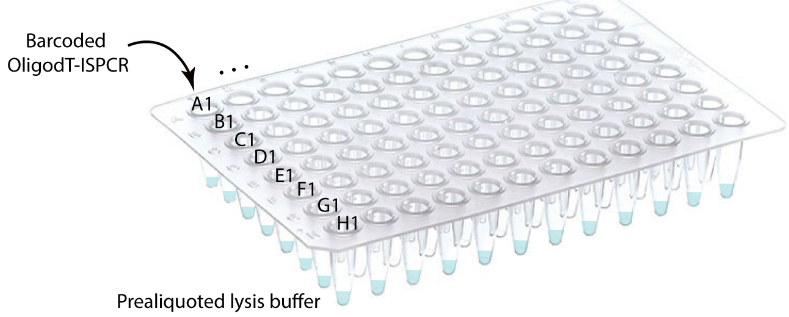

Figure MS4. Schematic representation of lysis buffer+barcoded oligodT stock plate preparation.

Lysis buffer	1 cell	Storage	Cat. No./Supplier
Triton 0.4%	0.95 µL	-20 °C	Sigma-Aldrich # T8787, resuspend in RNAse free water
RNAse Inhibitor	0.05 µL	-20 °C	TAKARA #2313A
dNTPs (10 mM)	0.5 µL	-20 °C	Life Technologies #R0192
Protease (1.09 AU/mL in water)	0.05 µL	+4 °C	Qiagen #19155; resuspend in UltraPure DNAse/RNAse-Free Distilled Water
ERCC RNA spike-in mix (1:4e5)	0.02 µL	-80 °C (single use aliquot)	Ambion #4456740
**TOTAL**	**1.57 µL**		
Oligo-dT-ISPCR (10 µM) – barcoded, well-specific	0.5 µL	-20 °C	Custom HPLC primers, Biomers.net
**TOTAL**	**2.07 µL**		

3. Aliquot the mixture of lysis buffer+barcoded oligodT into each well of a 384 well-plate (FrameStar) following the layout below using a Biomek FxP Liquid Handler

(Beckman Coulter) of similar liquid handling platform. Each barcode will be aliquoted four times per plate, once in each quadrant (Figure MS5). Cover the plates with a PCR film and keep them on ice/in the fridge until use. Alternatively, sets of 384 barcoded oligodTs might be used.

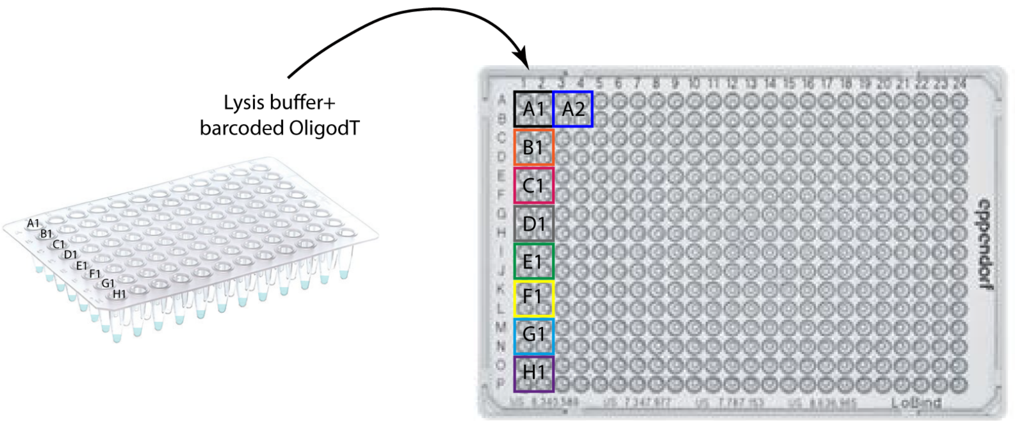

Figure MS5. Schematic representation of lysis buffer+barcoded oligodT aliquoting into 384 well plates.

4. Prepare the sorter for single-cell sorting. Use single cell purity mode and keep the event rate low (less than 1000/s).

5. Perform an alignment test sort using a 384 well plate (Corning® 384 well microplate, CLS3702-100EA) and sort one fluorescent bead per well. Check under a fluorescent microscope that there is only one bead in each well and that the position of the bead is centered at the very bottom of the plate. If correct, alignment checks are now complete.

6. Check sorter alignment: use a 384-well PCR plate (FrameStar, same model as the one in which single cells will be sorted) covered with a PCR film, and sort 50 cells in the four corners of the plate (positions 1-A, 1-P, 24-A and 24-P). Droplets should be positioned in the centre of the wells if the sorter is correctly aligned; if not, make necessary adjustments until drops are falling perfectly into each well. After this initial check, remove the PCR film and sort 50 cells into the same four corners of the 384 plate: drops should now be deposited at the very bottom of each well with no traces of liquid been left in the sides of each well.

7. Sort cells directly into a 384 well PCR plate (FrameStar) containing lysis buffer+barcoded oligodT-ISPCR, cover the plate with an aluminium PCR film (StarLab), spin down the plate and incubate 5 minutes at room temperature to allow for protease digestion. If sorting time is longer than 10 minutes, there is no need to incubate the plate further.

8. Put the plate directly into dry ice and store at -80 °C up to 1-2 months. (Processing plates after 3 months of -80 °C storage has shown decreased yield and/or signs of RNA degradation).

Heat inactivation, cDNA synthesis and amplification (RTPCR) – Timing: 6.5 hours; 1.5 hours hands-on time

9. Transport the plate(s) and TSO-LNA aliquot from -80 °C storage on dry ice to a ‘pre-amplification’ dedicated workspace/clean room.

10. Thaw the 5X Buffer, RT-PCR Grade Water and any mRNA targeting primers that you might add to the mix. These can be thawed at room temperature. Aliquot them into an RNase free tube to prepare a master mix for the retrotranscription (RT) step as per the table below. RNase inhibitor, TSO-LNA and SMARTScribe enzyme will be added to the mix during heat inactivation step.

RT	1 cell	Storage	Cat. No.
**Buffer 5X**	1.00 µL	-20 °C	Clontech - Cat. No. 639537 (delivered with enzyme)
**RNase Inhibitor** (wait until the 72C step to add it)	0.125 µL	-20 °C	TAKARA - 2313A
**TSO-LNA (100 uM) -** wait until 72C step to add it	0.05 µL	-80 °C	Custom TSO-LNA oligo from Exiqon-Qiagen (same as Picelli et al., 2013); avoid freeze/thaw cycles
**RT-PCR Grade Water**	Variable	-20 °C	Life Tech - AM9935
**mRNA primers** (0.0175 µL of each primer from a 200 µM stock)	Variable	-20 °C	Custom HPLC purified primers from biomers.net; resuspend in RNAse Free TE/water
**SMARTScribe enzyme-** wait until 72C step to add it	0.5 µL	-20 °C	Clontech - Cat. No. 639537
**TOTAL**	2.80 µL		
**TOTAL (cumulative)**	4.87 µL		

11. Incubate the sample plate 15 minutes at 72 °C in a thermocycler (no RT mix has been added at this point). This step will inactivate the protease included in the lysis buffer so it doesn’t interfere with any subsequent enzymatic steps.

12. During the heat inactivation, add the RNAse Inhibitor, TSO-LNA and RT enzyme to the RT master mix on ice/cold block. Vortex and spin down.

13. Once the heat inactivation step is finished, take the plate out of the thermocycler, spin down and place into ice/cold rack. Aliquot 2.8 µL of RT mix into each well and carefully seal the plate with a PCR film (MicroAMp Clear Adhesive Film, Cat. No. 4306311). Note: it is essential that this step is performed within 5-7 minutes to avoid RNA degradation.

14. Spin down and run the following program in a thermocycler:

Temperature	Time	Cycles
42 C	90 min	1
50 C	2 min	10 cycles
42 C	2 min	
70 C	15 min	1
4 C	HOLD	-

15. Fifteen minutes before the RT program finishes, start thawing reagents to prepare the PCR master mix.

PCR	1 cell	Storage	Cat. No.
**2X Buffer**	6.25 µL	-20 °C	Clontech Cat#638509 (delivered with enzyme)
**ISPCR (10 µM)**	0.0625 µL	-20 °C	Custom HPLC oligo from biomers.net (same as Picelli et al., 2013)
**RT-PCR Water**	Variable	-20 °C	Life Technologies #AM9935
**SeqAMP Enzyme -** wait until RT is about to finish to add	0.25 µL	-20 °C	Clontech Cat#638509
**cDNA primers -** (0.0175 µL from each primer from 20 µM stock)	Variable	-20 °C	Custom HPLC purified primers from biomers.net; resuspend in RNAse Free TE/water
**Genomic primers** (0.05 µL from each primer from a 200 µM stock)	Variable	-20 °C	
**TOTAL**	7.50 µL		
**TOTAL (cumulative)**	12.37 µL		

16. Once the RT program is finished, spin down the plate and add PCR master mix on ice/cold rack. Spin down the plate at 1000 g for 15 seconds. Take outside of the clean room workspace, place in a thermocycler and run the following program:

Temperature	Time	Cycles
98 C	3 min	1
98 C	00:15	24 cycles (single HSPCs)
67 C	00:20	
72 C	6 min	
72 C	5 min	1
4 C	HOLD	1

Pooling and bead clean-up – Timing: 45 minutes

17. Pool 1 µL of amplified cDNA+amplicon mix from each uniquely-barcoded well of the 384 well-plate into an eppendorf tube using a liquid handler platform. Four pools should be made from each 384-well plate, corresponding to 96 cells of each quadrant of uniquely-barcoded wells. Once pooled, perform bead purification. Aliquot 80 µL of pooled cDNA into a V-shaped 96 well plate (Cat. No. P-96-450V-C, Axygen) and aliquot 48 µL pre-warmed Ampure XP beads (Beckman Coulter;

Cat. No. A63881) into each well (0.6:1 beads to cDNA ratio). Incubate for 5 minutes at room temperature. Note that whilst polyadenylated cDNA has been uniquely-barcoded, amplicons corresponding to each single cell don't contain unique barcodes and therefore precaution should be taken to avoid cross-contamination between wells at this stage.

18. Incubate mixture into a 96-well magnetic stand for 2 minutes. Once the liquid is clear of beads, remove the liquid.

19. Wash the beads twice with 80 % EtOH (freshly prepared, dilute EtOH in PCR grade water). Add 100 µL of ethanol to each well, incubate for 30 seconds and remove. Repeat once more (2 times in total) and use P10 tips to remove any remaining ethanol.

20. Leave the beads to air-dry for 3 minutes. Be careful not to overdry the beads at this point or it will be difficult to resuspend them. Resuspend the beads in 80 µL and repeat the bead purification step: add 48 µL of beads to the cleaned product, wash twice with ethanol and remove any remaning ethanol using P10 tips.

21. Resuspend the beads into 21 µL of EB Buffer (Qiagen Cat No./ID: 19086) with the plate off the magnet. Incubate for 30 seconds and put the plate back onto the magnet.

22. Incubate on the magnet until the liquid is clear of beads, then transfer 20 µL of purified cDNA library to a new plate for -20 °C storage or further processing.

23. Check cDNA traces quality and size distributions using Bioanalyzer (Agilent), Fragment Analyzer Automated CE System (Advanced Analytical) or similar capillary arrays (Figure MS6). If primer dimers are detected at this stage (100-300 bp peaks), libraries should be re-purified with Ampure XP beads.

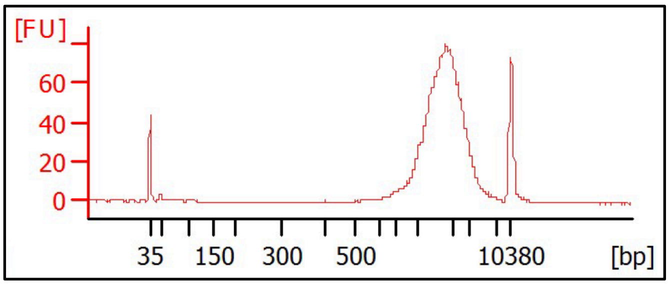

Figure MS6. Representative cDNA traces of pooled and bead-purified amplified cDNA libraries from 96 HSPCs.

Whole transcriptome library preparation – Timing: 45 minutes

24. Library preparation is performed using a commercially available Nextera XT Kit (FC-131-1096, Illumina) with modifications in the indexing PCR step. First, prepare one tube for each bead-purified cDNA pool and add 10 µL of Tagmentation Buffer into each tube.

25. Add 1 ng of purified cDNA from each pool, up to a total volume of 5 μL and 5 μL of Amplicon Tagmentation Mix (ATM). Incubate 6 minutes at 55 C (total volume 20 μL).

Reagents	1 reaction (μL)
Tagmentation Buffer	10
Bead purified cDNA (0.2 ng/uL)	5
ATM (Amplicon Tagment Mix)	5
**TOTAL**	20

26. Once the incubation is finished, add 5 μL of NT buffer to neutralize the tagmentation reaction.

27. Prepare PCR master mix as outlined below. i7 index primers are commercially available (Illumina, Cat#FC-131-1001); P5_index primer is a custom indexing primer (see Key Resources).

Reagents	1 reaction (μL)
i7 index (2 μM)	5 μL
P5_index (10 μM)	1 μL
NPM (PCR master mix)	15 μL
Water	4 μL
**TOTAL**	25 μL
**TOTAL (cumulative)**	50 μL

28. Incubate in a thermocycler and run the following PCR program:

Temperature	Time	Cycles
72 C	3 minutes	1
95 C	30 seconds	1
95 C	10 seconds	14 cycles
55 C	30 seconds	
72 C	30 seconds	
72 C	5 minutes	1
4 C	HOLD	1

29. Bead-purify tagmented libraries twice using Ampure XP beads. For the first bead purification step, use 34 μL of beads and 50 μL of library - resuspend in 34 μL of EB buffer (Qiagen) and use the product to perform a second bead purification using 20 μL of beads. Resuspend the final product in a total volume of 20 μL of EB buffer (Qiagen).

30. Run libraries on D5000 TapeStation or similar capillary array. Library fragments should be from 300 bp to 800 bp on average (Figure MS7):

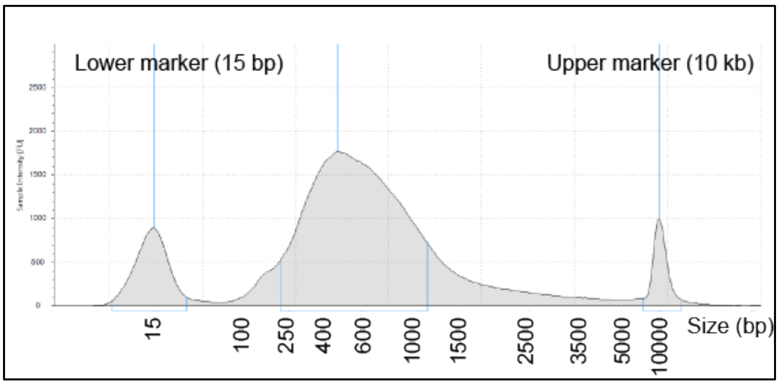

Figure MS7. Representative traces of tagmented, amplified and bead-purified 3'-TARGETseq Nextera XT libraries.

31. Quantify tagmented and barcoded libraries using Qubit (ThermoFisher, Cat. No. 32854) and pool equimolar concentrations of each tagmented library. Quantify the final pool and sequence on a NextSeq/HiSeq platform using custom P5_SEQ sequencing primer for Read1 (See Key Resources). Index read and Read2 use standard sequencing primers provided within the commercially-available sequencing cartridge. If using the NextSeq platform, load a 3 pM library diluted in 1.3 mL of HT1 Buffer (Illumina) and 900 nM of P5_SEQ primer in a total volume of 3 mL of HT1 buffer.

Single cell genotyping library preparation for NGS – Timing: 5 hours – 2 hours hands on time

32. Take one aliquot of the unpurified cDNA-amplicon mix, dilute 1:2 with PCR Grade water and use as an input for the first barcoding PCR (PCR1). Perform an individual PCR reaction for each sample in a 384 well-plate (FrameStar 384, Cat. No. 4ti-0384/C). During this PCR reaction, target-specific primers attached to universal tags (CS1/CS2 adaptors) will be added to each amplicon from each sample, in order to prepare a targeted sequencing library. Targets with similar amplification efficiencies might be amplified simultaneously in the same reaction for the same single cell. Note: while oligodT-primed mRNA molecules carry a cell-specific barcode, gDNA and cDNA pre-amplified amplicons will not have a cell-specific barcode and, therefore, amplicons corresponding to each cell should be kept in individual wells of the 384 well-plate, taking precautions to avoid cross-well contamination.

33. Prepare PCR Mix and aliquot in the 384 well-plate using a Biomek FxP Liquid Handler (Beckman Coulter) of similar liquid handling platform:

**PCR1 BARCODING with target-specific primers**	**1 Reaction**	**Storage**	**Cat. No.**
KAPA 2G Ready Mix	3.125 µL	-20 °C	KAPA 2G Robust HS PCR Kit #KK5517
Primer F1+R1 (20 uM)	0.375 µL	-20 °C	Custom primers (Invitrogen) cartridge purification, resuspend in TE
Primer F2+R2 (20 uM)	0.375 µL	-20 °C	
Primer F3+R3 (20 uM)	0.375 µL	-20 °C	
Primer FX+RX…	…	…	

RT-PCR Grade Water	Variable	-20 °C	UltraPure DNAse/RNAse-Free Distilled Water, (Life Technologies, #10977035)
cDNA aliquot	1.5 μL	-20 °C	
**TOTAL**	6.25 μL		

34. Incubate in a thermocycler and run the following PCR program:

**PCR1 PROGRAM**		
Temperature	Time (min:sec)	Cycles
95 C	03:00	1
95 C	00:15	20
60 C	00:20	
72 C	01:00	
72 C	05:00	1
4 C	HOLD	

35. Use 2.5 μL of PCR1 product as an input for the next reaction (PCR2). During this step, sample-specific barcodes are attached to previously tagged amplicons using the Access Array™ Barcode Library for Illumina® Sequencers (384, Single Direction, Fluidigm). Barcode each sample in individual reactions.

36. Aliquot the barcodes (Access Array™ Barcode Library for Illumina® Sequencers) into a 384 well plate, and aliquot the PCR1 product into the same plate using a liquid handling platform.

37. Prepare the PCR master mix and aliquot.

**PCR2 BARCODING with Illumina compatible primers**	**1 Reaction**	**Storage**	**Cat. No.**
FastStart High Fidelity 10X Reaction Buffer	1 μL	-20 °C	FastStart High Fidelity PCR System REF:04738292001
MgCl2 (25 mM)	1.8 μL	-20 °C	
DMSO	0.5 μL	-20 °C	
dNTP Mix (10 mM)	0.2 μL	-20 °C	
FastStart High Fidelity Enzyme (5U/μL)	0.1 μL	-20 °C	
RT-PCR Grade Water	1.90 μL	-20 °C	UltraPure DNAse/RNAse-Free Distilled Water, (Life Technologies, #10977035)

Single-direction barcodes (2 uM, Fluidigm)	2.0 µL	-20 °C	Access Array™ Barcode Library for Illumina® Sequencers-384, Single Direction, Fluidigm (Cat. No. 100-4876)
PCR1 barcoding aliquot	2.5 µL	-20 °C	
**TOTAL**	10 µL		

38. Incubate in a thermocycler and run the following PCR program:

**PCR2 PROGRAM**		
Temperature	Time (min:sec)	Cycles
95 C	10:00	1
95 C	00:15	10
60 C	00:30	
72 C	01:00	
72 C	03:00	1
4 C	HOLD	

39. Pool amplicons from each barcoded library using a liquid handling platform and use Ampure XP beads to clean-up pooled libraries (0.8:1 beads to cDNA ratio). Quantify libraries using Qubit (ThermoFisher; Cat No. 32854) and check library size distribution and specific amplification of targeted amplicons on D1000 TapeStation or similar capillary array (Figure MS8). Note: barcodes and adaptors add 103 bp extra to the original PCR product.

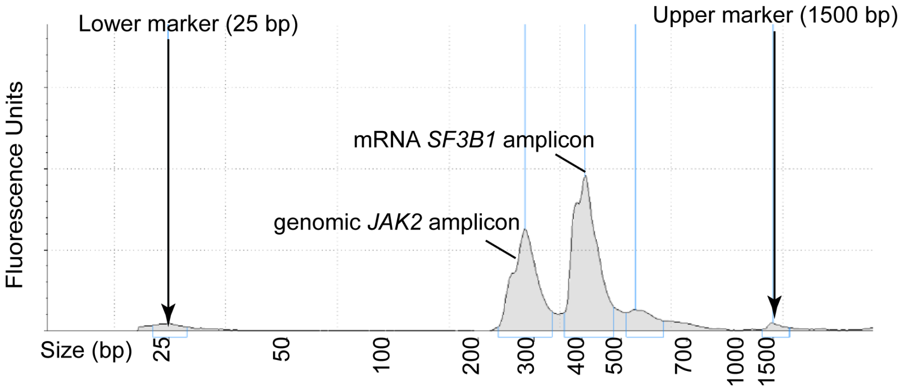

Figure MS8. Representative distributions of targeted amplicon libraries from genomic *JAK2* and mRNA *SF3B1* amplicons in a multiplexed reaction.

40. Libraries are ready for sequencing using custom sequencing primers targeted to CS1/CS2 tags (500 nM of CS1-seq and CS2-seq primers in a total volume of 700 µL for R1 and R2; 500 nM of CS1rc-seq and CS2rc-seq primers in a total volume of 700 µL for Index Read when using the MiSeq platform, Illumina). Note: CS1/CS2 and CS1rc/CS2rc sequencing primers contain LNA modifications (see Key Resources), as compared to CS1/CS2 tags used for PCR1 target-specific primers.

<u>Primer design and validation technical note.</u>

Pre-amplification (RTPCR) primer design

Primers should be designed taking into account the following considerations:

- Design genomic primers binding to at least one intronic region so they are compatible with parallel cDNA amplification.
- Primers for gDNA amplification should be checked for specificity against genomic and transcriptome references (so they are compatible with parallel cDNA amplification) using Primer BLAST (https://www.ncbi.nlm.nih.gov/tools/primer-blast/) or similar tools.
- Design mRNA/cDNA primers ideally in the exon before and the exon after your mutation/region of interest. An example of *JAK2* primer design can be found below (Figure MS9).

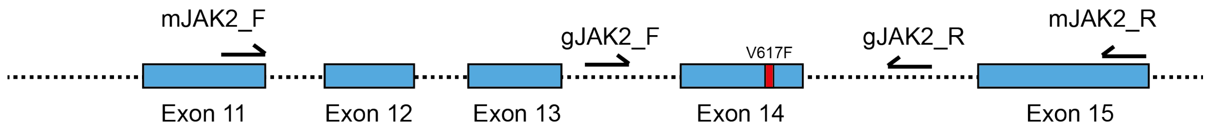

Figure MS9. Schematic representation of mRNA and gDNA *JAK2* primer design.

- Primers for mRNA/cDNA amplification should be checked for specificity against transcriptome references using Primer BLAST or similar tools.
- Design amplicons ideally ranging from 250 bp to 700 bp long. We have tested amplicons up to 1 kb, which worked optimally. In the rare event in which exons are longer than 1 kb, the preferred option is to design a unique primer pair spanning the mutation of interest.
- In the event that mutations of interest are in terminal exons or 3'-UTR regions, design two forward primers (mRNA and gDNA specific) and one unique reverse primer, which will amplify both types of amplicons. An example of *ASXL1* primer design can be found below (Figure MS10).

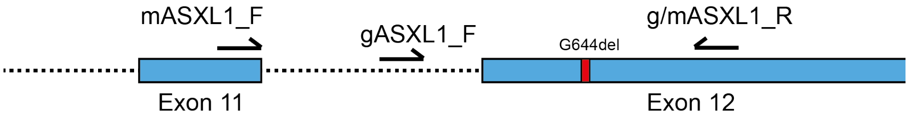

Figure MS10. Schematic representation of mRNA and gDNA *ASXL1* primer design.

cDNA primers used in the PCR step contain the same primer sequence used for mRNA primers in the RT step, but are attached to ISPCR adaptors (ISPCR adaptor sequence: 5'- AAGCAGTGGTATCAACGCAGAGT-3') in the 5'-end of each primer. This increases amplification efficiency of cDNA targets. Importantly, in the specific case of terminal exons where a common primer is used to amplify both cDNA and gDNA molecules, cDNA primers used in the PCR step should not be attached to ISPCR adaptors, as this will create concatemers that will disrupt the successful generation of cDNA libraries.

Pre-amplification primer validation

Primers used for gDNA and mRNA/cDNA pre-amplification should be validated for specificity using bulk gDNA and bulk cDNA, respectively. If primers are not specific or they present low amplification efficiencies, they should be redesigned.

Primer multiplexing strategies for pre-amplification should be validated for the generation of excessive primer dimers and concatemers in a minimum of 8 single cell samples. Examples of a good and bad library (primers generating concatemers) are shown below (Figure MS11). Figure MS11a represents a good quality cDNA library; Figure MS11b represents a good quality library despite high primer dimer concentration and Figure MS11c represents a bad quality cDNA library in which primers are interfering with cDNA amplification.

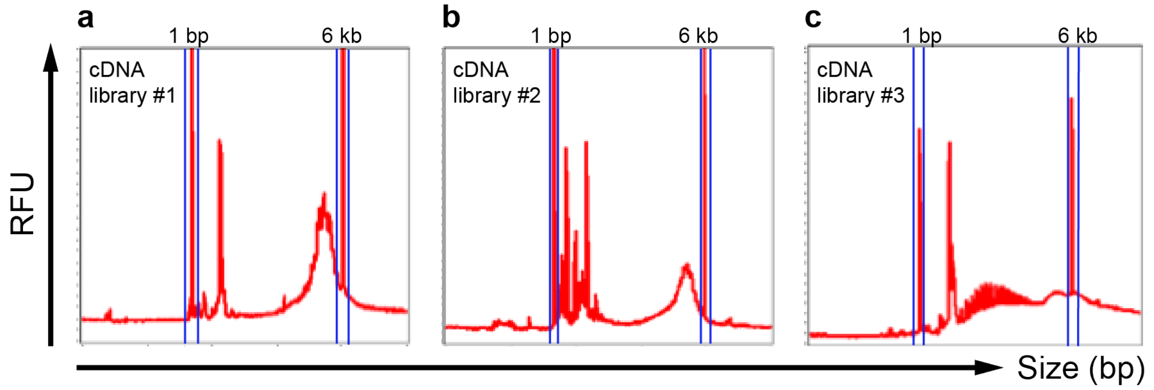

Figure MS11. Representative cDNA libraries from single HSPCs produced using different primer multiplex combinations.

When primer combinations generate concatemers or greatly decrease cDNA yield, such as the case presented in Figure MS11c, each primer pair should be tested individually in single cells and those pairs originating concatemers should be redesigned. Alternatively, when mRNA primer pairs generate concatemers, mRNA primer concentration might be decreased, down to a minimum 35 nM in the RT mix. gDNA primer concentration should not be decreased.

Custom barcoding primer design (PCR1 barcoding primers)

Specific primers for gDNA and cDNA used during PCR1-barcoding should be designed nested from the original RT+PCR amplicon (pre-amplification primers) to increase specific amplification and PCR efficiency. Specificity should be checked against transcriptome references for both types of molecules, and they should be validated using bulk genomic DNA and cDNA, respectively. Primers are tagged to CS1/CS2 universal adaptors in the 5'end (Forward adaptor, CS1: ACACTGACGACATGGTTCTACA; Reverse adaptor, CS2: TACGGTAGCAGAGACTTGGTCT), which will be used to add cell-specific barcodes during PCR2 step.

PCR1 primers should be different for each type of molecule (gDNA or cDNA), so that independent mutational readouts can be obtained from each, bioinformatically extracting reads matching each primer sequence. In the specific case of terminal exons, whilst one unique reverse primer was used during RT-PCR steps, two reverse primers should be

used for PCR1 barcoding, and therefore gDNA and mRNA amplicons should be barcoded in different reactions in such case.

When sequencing using a sequencing platform with 300 cycles configuration (150 bp R1 and 150 bp R2), primers should be designed taking into account the relative distance of the mutation to start of the primer, so that the mutation is well covered during sequencing. Sequencing configurations with shorter reads are not recommended.

Custom barcoding primer validation

Primers used for gDNA and cDNA PCR1-barcoding should be validated for specificity and amplification efficiency in pre-amplified single cell samples. To do that, PCR1 should be performed individually for each target using 35 cycles of PCR amplification, and specific amplification should be checked on a Fragment Analyzer platform (Agilent) or similar capillary array (Figure MS12). Amplification efficiency might be derived from the quantification of specific product obtained in each case. Alternatively, specificity and amplification efficiency might be assessed with qPCR. If primers are not specific, they should be redesigned.

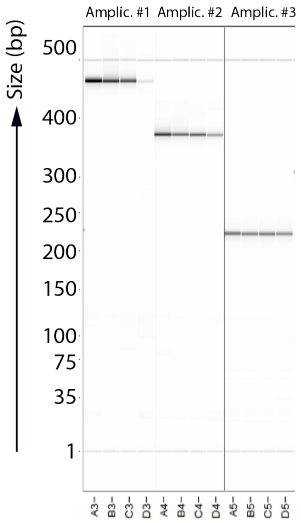

Figure MS12. Representative example of Fragment Analyzer results for three different PCR1 primer pairs.

Example of primers used for *JAK2*-V617F amplification

Primer Name	Primer sequence	Step	Type	Purification
mJAK2_F	TAAATGCTGTCCCCCAAAGC	RT	mRNA	HPLC
mJAK2_R	CCATGCCAACTGTTTAGCAAC	RT	mRNA	HPLC
gJAK2_F	ccaaagcacattgtatcctcatct	PCR	gDNA	HPLC
gJAK2_R	cactgacacctagctgtgatcct	PCR	gDNA	HPLC
ISPCR_mJAK2_F	AAGCAGTGGTATCAACGCAGAGTTAAATGCTGTCCCCCAAAGC	PCR	cDNA	HPLC
ISPCR_mJAK2_R	AAGCAGTGGTATCAACGCAGAGTCCATGCCAACTGTTTAGCAAC	PCR	cDNA	HPLC
mJAK2_PCR1_F	ACACTGACGACATGGTTCTACATCTGGATAAAGCACACAGAAACT	PCR1	cDNA	Desalted
mJAK2_PCR1_R	TACGGTAGCAGAGACTTGGTCTTCCAAATTTTACAAACTCCTGAACC	PCR1	cDNA	Desalted
gJAK2_PCR1_F	ACACTGACGACATGGTTCTACAttatggacaacagtcaaacaacaa	PCR1	gDNA	Desalted
gJAK2_PCR1_R	TACGGTAGCAGAGACTTGGTCTaaaggcattagaaagcctgtagt	PCR1	gDNA	Desalted

ISPCR adaptor is labelled in orange; CS1/CS2 adaptors are labelled in blue
